# Gold-catalyzed bicyclic annulations of 4-methoxy-1,2-dienyl-5-ynes with isoxazoles to form indolizine derivatives *via* an Au-π-allene intermediate†
†Electronic supplementary information (ESI) available. CCDC 1894125–1894129 and 1913325. For ESI and crystallographic data in CIF or other electronic format see DOI: 10.1039/c9sc00735k


**DOI:** 10.1039/c9sc00735k

**Published:** 2019-05-22

**Authors:** Antony Sekar Kulandai Raj, Kuo-Chen Tan, Liang-Yu Chen, Mu-Jeng Cheng, Rai-Shung Liu

**Affiliations:** a Frontier Research Centers on Fundamental and Applied Science of Matters , Department of Chemistry , National Tsing-Hua University , Hsinchu , Taiwan , Republic of China . Email: rsliu@mx.nthu.edu.tw; b Department of Chemistry , National Cheng Kung University , Tainan 701 , Taiwan . Email: mjcheng@mail.ncku.tw

## Abstract

Gold-catalyzed bicyclic annulations of 4-methoxy-1,2-dienyl-5-ynes with isoxazoles afford indolizine derivatives with a structural rearrangement. The mechanism of these new annulations does not involve α-imino gold carbenes generated from gold π-alkyne intermediates. We postulate alkyne attack on gold π-allenes, yielding vinyl gold carbenes. These newly generated carbenes react with isoxazole derivatives to yield Z-3-imino-2-en-1-als, further enabling sequential cyclizations to deliver indolizine derivatives in two distinct classes.

## Abstract 2

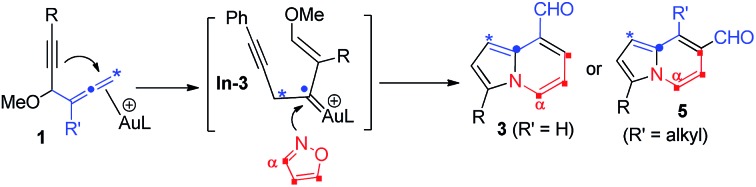
Gold-catalyzed bicyclic annulations of 4-methoxy-1,2-dienyl-5-ynes with isoxazoles afford indolizine derivatives; the reaction mechanism involves alkyne attack on a gold π-allene to yield a vinyl gold carbene.

## Introduction

The advent of gold catalysis has greatly promoted the synthetic utility of alkynes. Apart from the functionalizations of alkynes with O, N and C based nucleophiles, gold catalysts also accelerate the development of new alkyne annulations^1^ with π-bond motifs. Isoxazoles are readily available aromatic heterocycles; interest in their gold-catalyzed alkyne annulations^2^,^3^ is rapidly growing because of the easy generation of α-imino gold carbenes (eqn (1)). Ye and coworkers reported the first [3 + 2]-annulations of ynamides with isoxazoles to deliver pyrrole derivatives *via* α-imino gold carbenes **In-1** (eqn (1)).3a–c The use of electron-deficient alkynes also afforded pyrrole products with similar carbene intermediates.3*d* We employed 1,4-diyn-3-ols to seek other azacycles,^4^ but still producing pyrrole derivatives *via* a 1,2-alkyne migration to α-imino gold carbenes (eqn (2)). Despite intensive efforts, the strong preference toward pyrrole products limits the utility of these isoxazole/alkyne annulations. Similar π-alkyne routes were observed for the anthranil/alkyne annulations, yielding indole derivatives.^5^ We sought to achieve the synthesis of other azacyclic compounds beyond pyrrole or indole derivatives; generation of intermediates other than α-imino gold carbenes is a viable route. This work reports gold-catalyzed bicyclic annulations of 4-methoxy-1-allenyl-5-ynes with isoxazoles to form 8- and 7-formylindolizines **3** and **5**; the structural rearrangement of products is noted here (eqn (3)). We postulate an atypical mechanism for these bicyclic annulations *via* a 1,4-alkyne migration, activated by a gold π-allene intermediate; the resulting vinyl gold carbene **In-3** is trapped by an isoxazole to enable initial sequential cyclizations before delivering indolizine products. This new annulation rationalizes the carbon source of indolizines **3** and **5** from the two reactants well.

Previous work: gold carbene *via* π-alkyne intermediates
1

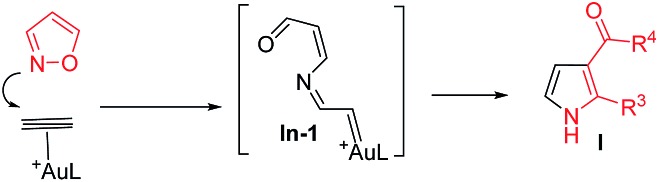




One example:
2

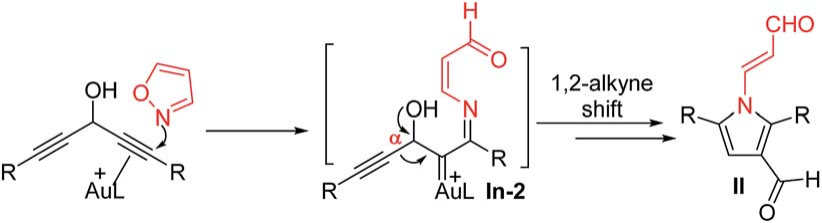




This work: vinyl gold carbene *via* π-alkyne intermediates
3

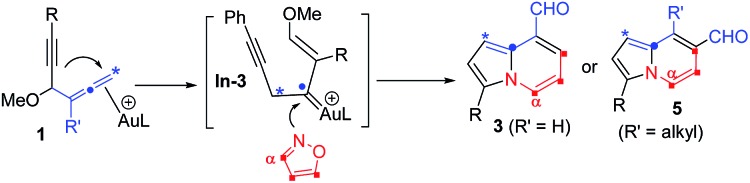




**Scheme 1:**
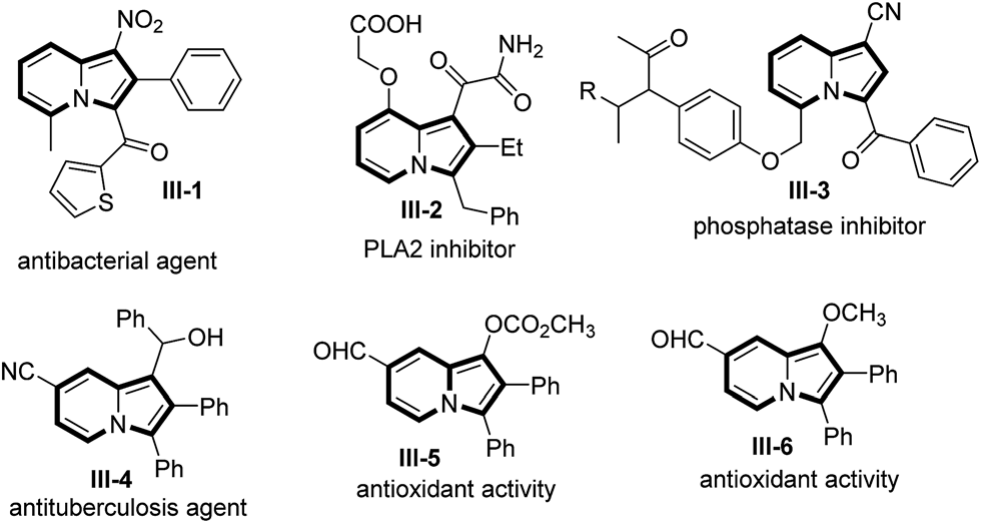
Representative bioactive molecules.

Indolizine frameworks are present in the core structures of natural products including (–)-swainsonine, (+)-castanospermine, lamellarins and camptothecin.^6^,^7^ Synthetic indolizine derivatives, such as compounds **III-1–III-4**, are demonstrated to be antibacterial reagents, PLA2 inhibitors, phosphatase inhibitors and antituberculosis agents^8^ whereas species **III-5** and **III-6** show antioxidant activity.^9^ Indolizine species **III-5** and **III-6** structurally match with our resulting products **5** bearing a C(7)-aldehyde (Scheme 1).
4

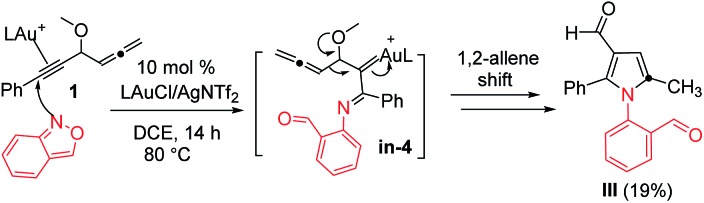




## Results and discussion

Our initial target focused on the reactions of 4-methoxy-1,2-dienyl-5-ynes **1a** with anthranil using gold catalysts; the reactions gave pyrrole derivatives **III** again (eqn (4)).^10^ A mechanistic analysis indicates a typical route of the alkyne activation, involving a 1,2-allene migration to the gold carbene center. We switch our attention to isoxazole derivatives. Table 1 shows the optimizations of a new bicyclic annulation of 4-methoxy-1,2-dienyl-5-yne **1a** with isoxazole **2a** using various gold catalysts. Our initial tests with IPrAuCl/AgNTf_2_ (10 mol%) in DCE at 25 °C (27 h) led to a high recovery of the starting alkyne **1a** (entry 1). IPrAuCl/AgNTf_2_ (10 mol%) in DCE at 45 °C (48 h) gave unreacted **1a** with a 28% recovery (entry 2). To our pleasure, the reaction in a hot DCE solution (65 °C, 14 h) afforded an indolizine derivative **3a** bearing a C(8)-aldehyde group; the yield was 88% (entry 3). Under these optimized conditions, P(*t*-Bu)_2_(*o*-biphenyl) AuCl/AgNTf_2_ was less efficient to yield product **3a** and unreacted **1a** in 62% and 21%, respectively (entry 4). Other gold phosphines such as LAuCl/AgNTf_2_ (L = PPh_3_, P(OPh)_3_) were catalytically inactive (entries 5 and 6). Alternations of silver salts as in IPrAuCl/AgX (X = SbF_6_ and OTf) rendered the reactions less efficient, giving compounds in 61% and 0% yields respectively; the reactions were only compatible with non-coordinating anions (entries 7 and 8). IPrAuCl or AgNTf_2_ alone (10 mol%) was entirely inactive (entries 9 and 10). IPrAuCl/AgNTf_2_ became inefficient in THF, MeCN and toluene (entries 11–13). The structure of compound **3a** was inferred from X-ray diffraction studies of its related compounds **3c** and **3d**,^11^ as depicted in Table 2, and further verified with ^1^H NOE spectra.

**Table 1:** Bicyclic annulations with various gold catalysts^*a*^

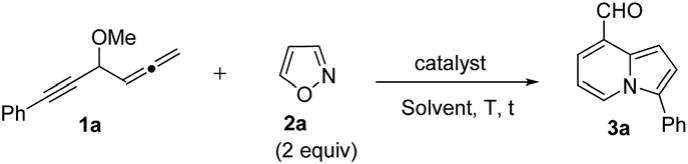						
Entry	Catalyst (mol%)	*T* [°C]	*t* [h]	Solvent	Yield^*b*^ [%]	
					**1a**	**3a**
1	IPrAuCl/AgNTf_2_ (10)^*c*^	25	27	DCE	75	Trace
2	IPrAuCl/AgNTf_2_ (10)	45	48	DCE	28	Trace
3	IPrAuCl/AgNTf_2_ (10)	65	14	DCE	—	88
4	LAuCl/AgNTf_2_ (10)^*d*^	65	27	DCE	21	62
5	PPh_3_AuCl/AgNTf_2_ (10)	65	35	DCE	94	—
6	P(OPh)_3_AuCl/AgNTf_2_ (10)	65	32	DCE	95	—
7	IPrAuCl/AgSbF_6_ (10)	65	24	DCE	24	61
8	IPrAuCl/AgOTf (10)	65	22	DCE	—	—
9	IPrAuCl (10)	65	13	DCE	85	—
10	AgNTf_2_ (10)	65	30	DCE	76	—
11	IPrAuCl/AgNTf_2_ (10)	65	25	THF	—	—
12	IPrAuCl/AgNTf_2_ (10)	80	21	MeCN	87	—
13	IPrAuCl/AgNTf_2_ (10)	100	21	Toluene	—	Trace

^*a*^[**1a**] = 0.15 M.

^*b*^Product yields are reported after separation from a silica column.

^*c*^IPr = 1,3-bis(diisopropylphenyl)imidazole-2-ylidene.

^*d*^L = P(*t*-Bu)_2_(*o*-biphenyl), DCE = 1,2-dichloroethane, DCM = dichloromethane, THF = tetrahydrofuran, MeCN = acetonitrile, Tf = trifluoromethanesulfonyl.

**Table 2:** Formation of 8-formylindolizines^*a*^

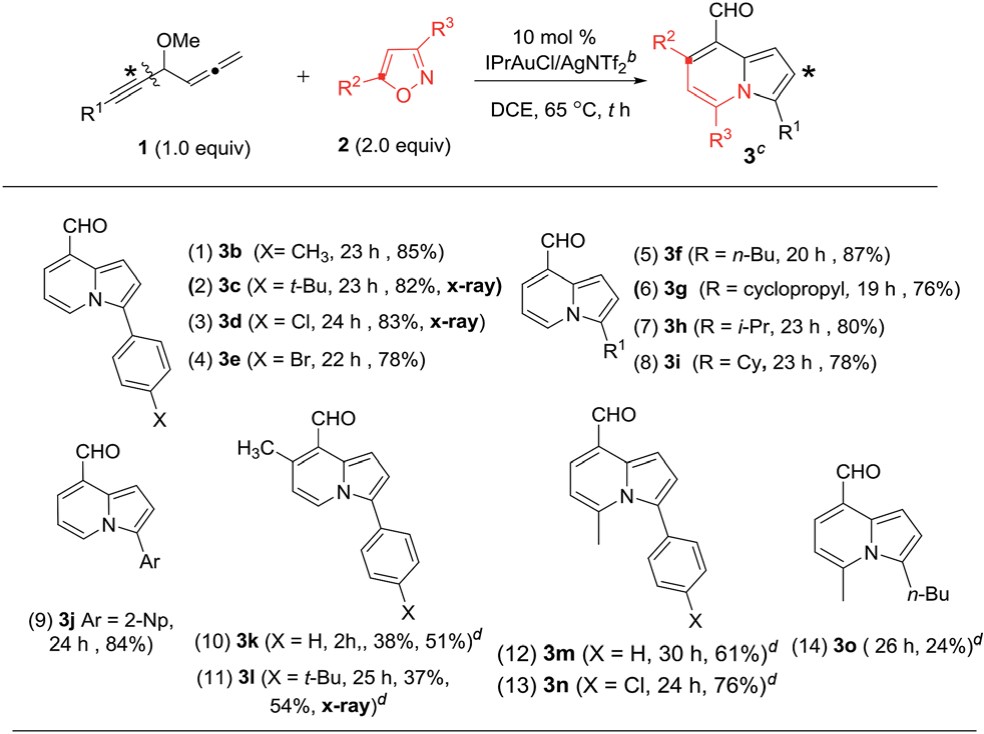

^*a*^[**1**] = 0.15 M.

^*b*^IPr = 1,3-bis(diisopropylphenyl)imidazole-2-ylidene.

^*c*^Product yields are reported after separation from a silica column.

^*d*^These data correspond to 3 equiv. of isoxazole, Tf = trifluoromethanesulfonyl.

We assessed the generality of these bicyclic annulations with various 4-methoxy-1,2-dienyl-5-ynes and substituted isoxazoles; the results are depicted in Table 2. We tested these annulations first on 4-phenylethynyl allene substrates **1b–1e** (X = Me, *tert*-butyl, Cl and Br), smoothly affording 8-formylindolizine derivatives **3b–3e** in good yields (78–85%, entries 1–4); X-ray diffraction revealed that products **3c** and **3d** bear an aldehyde at their C(8)-carbons. The reactions were further compatible with alkylethynyl allenes **1f–1i** (R = *n*-butyl, cyclopropyl, isopropyl and cyclohexyl), yielding desired indolizines **3f–3i** in 76–87% (entries 5–8). For 2-napthylethynyl allene **1j**, its corresponding indolizine **3j** was obtained in 84% yield (entry 9). We performed the reaction on 5-methylisoxazole **2b** (R^2^ = Me), yielding 7-methyl-8-formylindolizines **3k** and **3l** in 38% and 37% yields, respectively(entries 10 and 11); the yields of the two products were increased to 51% and 54% using a high loading of isoxazole **2b** (3 equiv.). The molecular structure of indolizine **3l** was confirmed with X-ray diffraction.^11^ For 3-methylisoxazole **2c** (R^3^ = Me), its corresponding indolizines **3m** and **3n** were obtained in 61% and 76% yields respectively (entries 12 and 13); the proposed structure of **3m** was verified by ^1^H NOE spectra. We tested the reactions on an alkyl-substituted allene substrate with **2c** rendered desired **3o** with 24% yield (entry 14). Structural analysis of these indolizine products supports a 1,4-migration of the alkynyl moiety to the C(1)-allene carbon.

As depicted in Table 3, 3-disubstituted allene derivatives **4** gave distinct 7-formylindolizines **5** under the same conditions. We assessed the scope of this new annulation using various allenylynes bearing R^1^ and R^2^ substituents. Entries 1–3 show the applicability of this catalysis to various phenylethynyl allenes **4a–4c** (X = H, Cl and Br), rendering the desired products **5a–5c** in 69–76% yields (entries 1–3); the molecular structure of the chloro derivative **5b** was determined with X-ray diffraction.^11^ For 2-napthylethynyl allene **4d**, its corresponding product **5d** was obtained in 71% yield (entry 4). The reaction was extensible to substrate **4e** bearing 3-methylallene (R^2^ = Me), yielding compound **5e** in 39% yield (entry 5). We tested the reactions on all alkyl-substituted 1,2-dienyl-5-allenes **4f–4j** (R^1^, R^2^ = alkyl), delivering the desired 7-formylindolizines **5f–5j** in satisfactory yields (76–81%, entries 6–10). The proposed structure of compound **5j** was confirmed with X-ray diffraction study.^11^

**Table 3:** Formation of 7-formylindolizines^*a*^

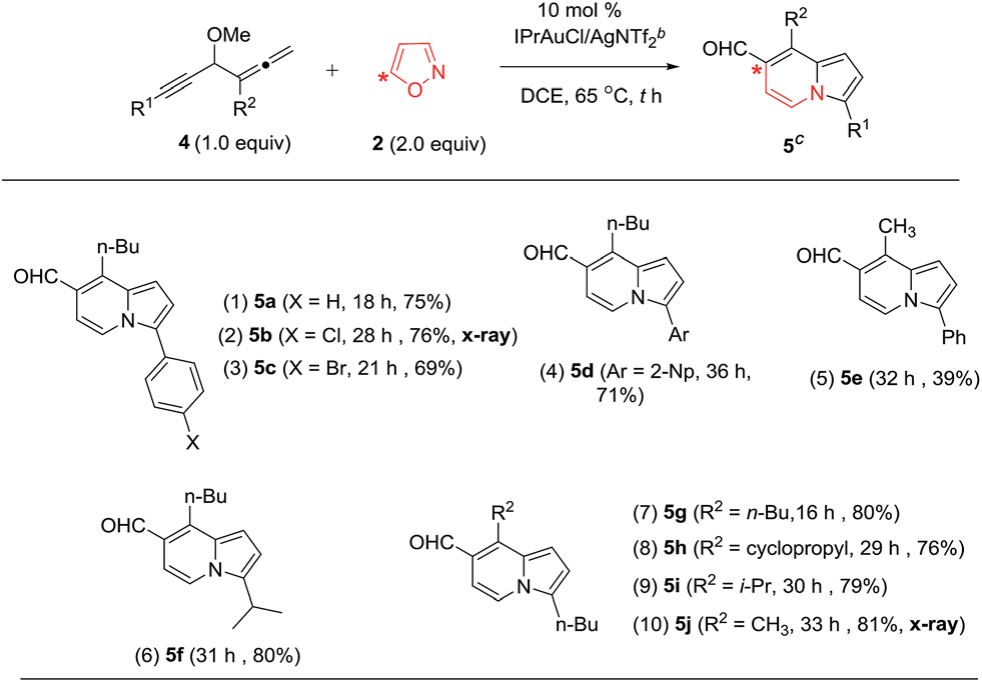

^*a*^[**4**] = 0.15 M.

^*b*^IPr = 1,3-bis(diisopropylphenyl)imidazole-2-ylidene.

^*c*^Product yields are reported after separation from a silica column.

To test the electronic effect of allenyl substituents, we prepared an allenyl ester **6** that reacted with 5-arylisoxazoles **2d** (Ar = Ph) and **2e** (Ar = 4-ClPh) to yield indolizine derivatives **7a** and **7b** (eqn (5)). The X-ray diffraction results of compound **7b** confirmed its structure with no 1,4-alkyne shift; the formation of these two products arose from gold π-alkyne intermediates as described before (eqn (4)). The change of chemoselectivity is attributed to a weak coordination between gold and an allenyl ester.
5

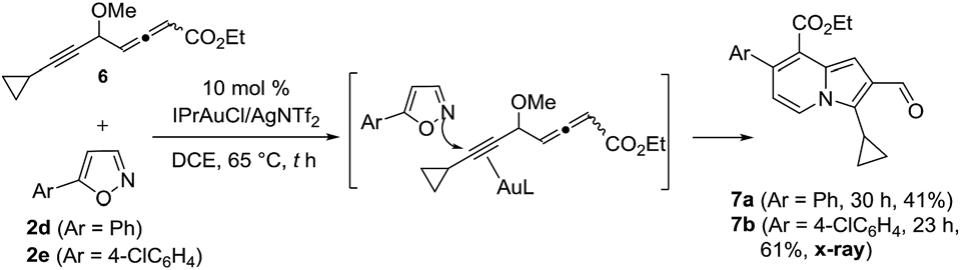




We performed a series of experiments to elucidate the mechanisms of formation of 8- and 7-formylindolizines **3** and **5**. We prepared ^13^C-enriched **1a** and **4e**; each contained 10% ^13^C content in the CH–OMe carbon. Their resulting products ^13^C-**3a** and ^13^C-**5e** were found to have the enrichment at the aldehyde carbons (eqn (6) and (7)). We prepared d_2_-**1a** bearing =CD_2_ at the allene C(1)-carbon; its resulting indolizine d_2_-**3a** comprised equal deuterium content (*X* = *Y* = 0.72 D) at the two pyrrolyl carbons. We also performed a crossover experiment involving d_2_-**1a** and d_0_-**1b**; this mixture only produced d_2_-**3a** and d_0_-**3b** according the mass analysis. The entire 1,2-dienyl-5-yne skeleton **1** remained completely on the resulting indolizine molecule.
6

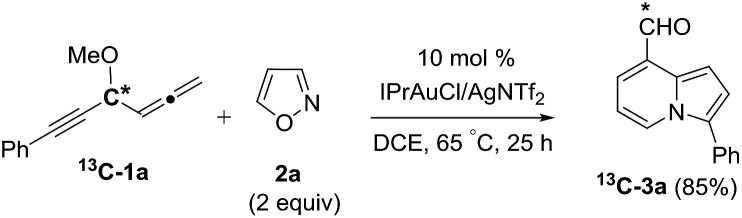



7

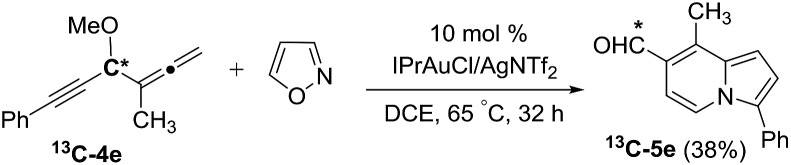



8

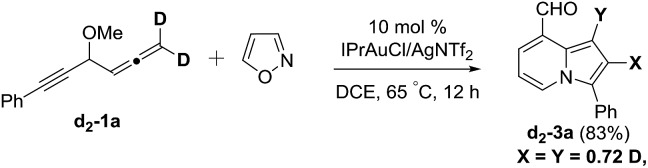



9

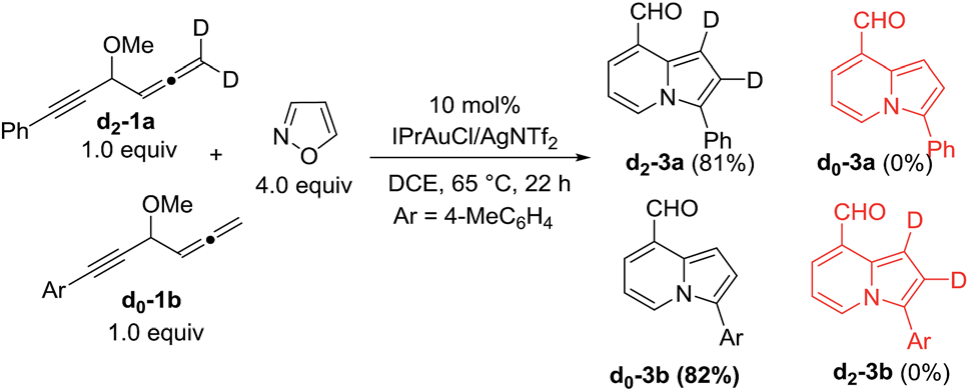




**Scheme 2:**
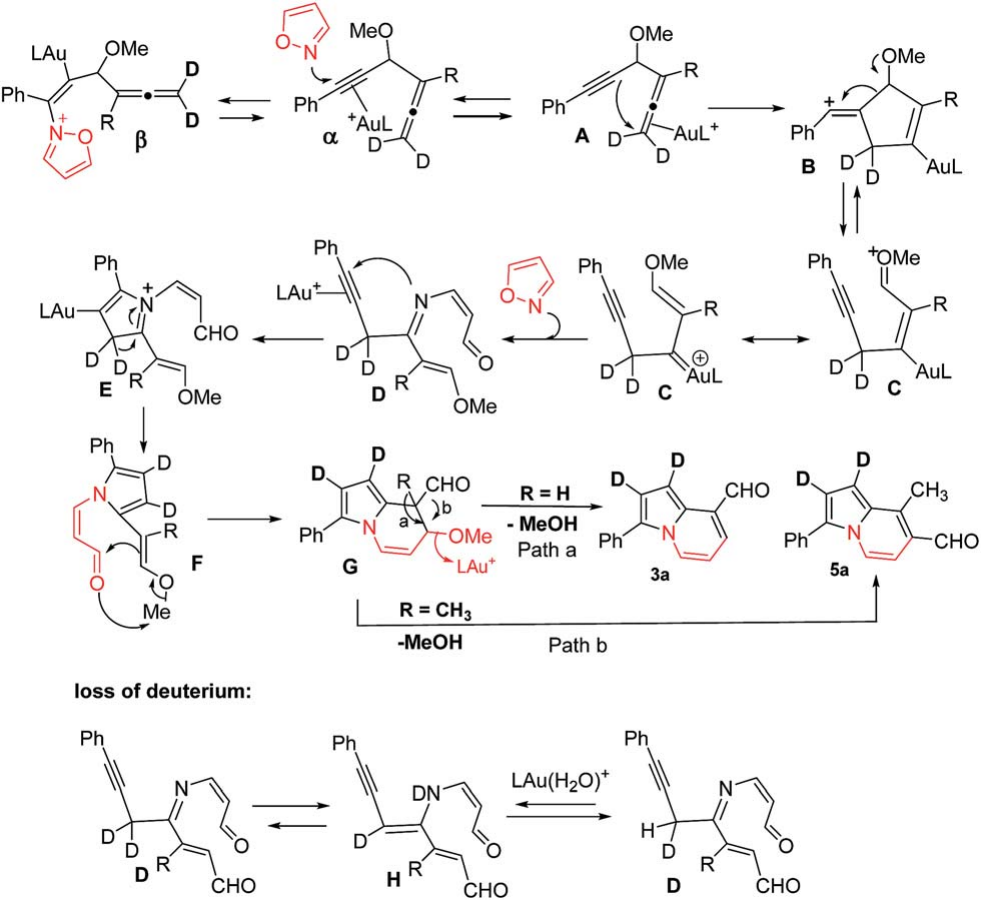
A proposed mechanism.

According the structural analysis of the resulting indolizines **3** and **5**, we postulate a mechanism involving an allene-activation route. This mechanism rationalizes the deuterium and crossover experiments well (eqn (8) and (9)). We use d_2_-**1a** (R = H) as a tool to verify the mechanism. In the N-attack of isoxazole **2a** with Au-π-alkyne α, the resulting intermediate β has a highly aromatic isoxazole ring that is difficult to cleave. We postulate an alternative path involving nucleophilic attack of an alkyne at its tethered Au-π-allene **A** to form vinyl cation **B**. An alkyne as a nucleophile to attack an electrophilic Au-π-allene is noted in gold catalysis.^12^ We conceive that this vinyl cation induces a subsequent C–C bond cleavage of species **B** to form phenylalkyne species **C** bearing an allyl cation **C**, as stabilized by the gold and methoxy group. This species has a resonance form of vinyl gold carbene that reacts smoothly with isoxazole to yield a 3-imino-2-en-1-al **D** with *Z*-configuration.^13^ An amination on the alkyne of species **D** is expected to form an azacyclic intermediate **E** which leads to the desired pyrrole intermediate **F**. For mono-substituted allenes **1** (R = H), a further carbonyl–ene reaction of species **F** yields pyrrole-fused six-membered species **G**, which loses MeOH to yield 8-formyl indolizine **3a**. In the case of a 3,3-disubstituted allene **4** (R = alkyl), a 1,2-formyl shift to the neighboring carbocation occurs preferentially to give 7-formyl indolizine derivative **5a** (Scheme 2).

This postulated mechanism rationalizes a small loss of deuterium content of the indolizine product d_2_-**3a** (*X* = *Y* = 0.72 D), as depicted in eqn (8). In the hot DCE solution (65 °C 12 h), an imine–enamine tautomerization, as shown by species **D** and **H**, results in a deuterium loss of species **D** because of an exchange with residual water. In this mechanism, a major concern is the cleavage of the sigma C–C bond of species **B** to yield vinyl gold carbene **C**.

**Fig. 1:**
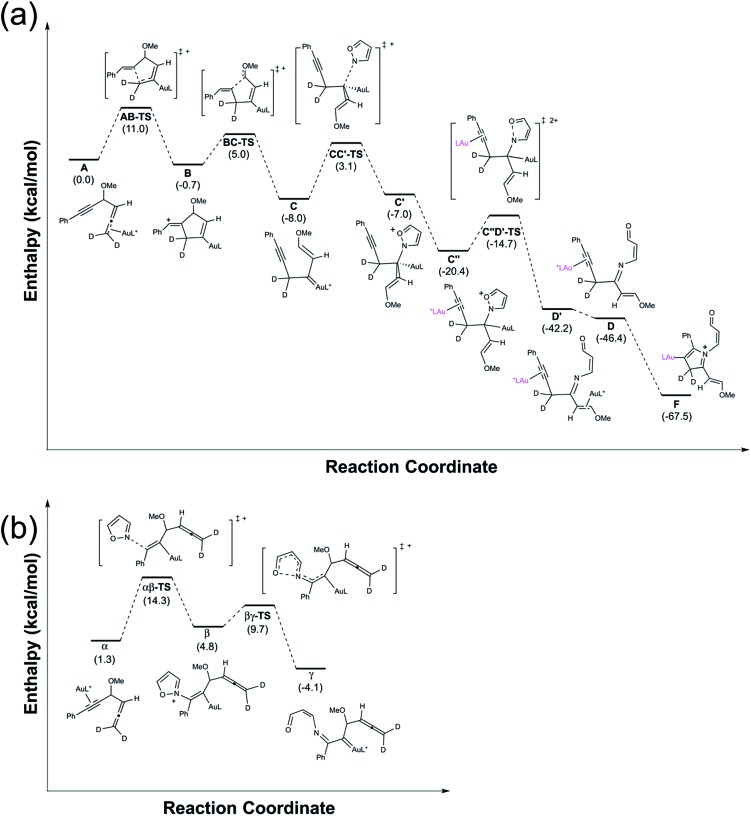
The enthalpic energy profile calculated using density functional theory.

Calculations with density functional theory (B3LYP) were performed to support our proposed mechanism. Attention was paid to the transformations of the gold π-allene intermediate **A** (Fig. 1) to gold pyrrolium (**F**), since the last few steps are well known in organic reactions. 1,4-Alkyne migration of **A** to form **C** is a stepwise process: transformation **A** → **B** occurs with Δ*H*^‡^/Δ*H* = 11.0/–0.7 kcal mol; cleavage of the C–C bond of species **B** results in the formation of intermediate **C** with Δ*H*^‡^/Δ*H* = 5.7/–7.3 kcal mol^–1^. Species **C** is subsequently attacked by an isoxazole to generate **C′** with Δ*H*^‡^/Δ*H* = 11.1/1.0 kcal mol^–1^. Next, the ligation of another IPrAu^+^ to species **C′** is expected to form a digold species **C′′** with Δ*H* = –13.4 kcal mol; this process is accompanied by a N–O cleavage of the isoxazole moiety of species **C′′** to generate **D′** with Δ*H*^‡^/Δ*H* = 5.7/–21.8 kcal mol^–1^. Finally, a release of IPrAu^+^ from species **D′** eventually yields a gold-π-alkyne **D** with Δ*H* = –4.2 kcal mol; an intramolecular cyclization of species **D** generates gold-containing pyrrolium species **F** with no kinetic barrier and Δ*H* = –21.1 kcal mol^–1^. In this **D** → **F** step, the electronic barrier is 0.01 kcal mol^–1^, which disappears after correction for zero-point energy. Overall, all the kinetic barriers are less than 11.1 kcal mol^–1^ with all the steps being thermodynamically downhill except the step **C** → **C′** (Δ*H* = +1.0 kcal mol^–1^). The entire reaction (**A** → **F**) releases an enthalpy –67.5 kcal mol^–1^. Our calculations thus show that the entire process is kinetically facile and thermodynamically favorable, verifying the proposed mechanism.

We also perform the calculation on a competitive reaction involving gold π-alkyne intermediates α, which has energy 1.3 kcal mol^–1^ greater than that of the gold π-allene (**A**). The attack of an isoxazole on π-alkyne α generated alkenylgold species β with Δ*H*^‡^/Δ*H* = 13.0/3.5 kcal mol^–1^. This was followed by a ring-opening reaction to form α-imino gold carbene γ with Δ*H*^‡^/Δ*H* = 4.9/–8.9 kcal mol^–1^. Notably, the barrier for formation and the energy state of intermediate β are greater than those of all intermediates in the π-allene route. We conclude that this π-alkyne route is unlikely to play an important role in the reaction.

## Conclusions

In summary, we report new gold-catalyzed bicyclic annulations between 4-methoxy-1,2-dienyl-5-ynes and isoxazoles to form 7- and 8-formyl indolizine derivatives.^13^ This reaction process does not follow a typical π-alkyne route; α-imino gold carbenes^14^,^15^ do not form here. Instead, the mechanism involves π-allene intermediates to induce a 1,4-alkyne shift, yielding a vinyl gold carbene **C** that is trapped with an isoxazole to generate an α-imino-2-en-1-al. Gold-catalyzed sequential cyclizations of this imine intermediate enable the construction of an indolizine skeleton. This mechanism rationalizes the isotope labeling and crossover experiments well. New versions for these gold-catalyzed annulations will be helpful for the design of new catalysis.

## Conflicts of interest

There are no conflicts of interest to declare.

## Supplementary Material

Supplementary information

Crystal structure data
