# Supplementary material for: Gold-catalyzed bicyclic annulations of 4-methoxy-1,2-dienyl-5-ynes with isoxazoles to form indolizine derivatives via an Au-π-allene intermediate
Source: Chem Sci. 2019 May 22;10(26):6437–42. doi: 10.1039/c9sc00735k (PMC6610539; doi:10.1039/c9sc00735k)
Supplement: Supplementary file 1 [file SC-010-C9SC00735K-s001.pdf]

Electronic Supplementary Material (ESI) for Chemical Science.
This journal is © The Royal Society of Chemistry 2019

# Supporting Information

## Gold-catalyzed Bicyclic Annulations of 4-Methoxy-1,2-dienyl-5-ynes with Isoxazoles to Form Indolizine Derivatives *via* Au-π-Allene Intermediate

Antony Sekar Kulandai Raj,[a] Kuo-Chen Tan,[a] Liang-Yu Chen,[b] Mu-Jeng Cheng*[b] and Rai-Shung Liu*[a]

[a]Frontier Research Centers on Fundamental and Applied Science of Matters and Department of Chemistry, National Tsing-Hua University, Hsinchu, Taiwan, ROC-----------------------------------
Email: rsliu@mx.nthu.edu.tw
[b]Deparment of Chemistry, National Cheng Kung University, Tainan 701, Taiwan,
Email: mjcheng@mail.ncku.edu.tw

## Contents:

**(1) Representative Synthetic Procedures: ----------------------------------------------------------2**

**(2) Standard procedures for catalytic operations: ------------------------------------------------4**

**(3) Mechanistic investigation: ---------------------------------------------------------------------------5**

**(4) Computational details: --------------------------------------------------------------------------------7**

**(5) Spectral data for key compounds: -----------------------------------------------------------------8**

**(6) X-ray crystallographic data of 3c, 3d, 3l, 5b, 5j & 7b: ---------------------------------------23**

**(7) Cartesian Coordinates for Optimized Structures: ------------------------------------------ 81**

**(8) $^1$H, $^{13}$C and NOE of key compounds --------------------------------------------------------151**

## (1) Representative Synthetic Procedures:

### (a) General procedure:

Unless otherwise noted, all the reactions for the preparation of the substrates were performed in oven-dried glassware under nitrogen atmosphere with freshly distilled solvents. The catalytic reactions were performed under Nitrogen atmosphere. DCE, DCM and Ether were distilled from $CaH_2$ under nitrogen. THF were distilled from Na metal under nitrogen. All other commercial reagents were used without further purification, unless otherwise indicated. $^1$H NMR and $^{13}$C NMR spectra were recorded on a Bruker 400 MHz, Varian 400 MHz and 600 MHz spectrometers using chloroform-*d* ($CDCl_3$) and *d*-Acetone as the internal standards.

### (b) Preparation of 4-Methoxy-1,2-dienyl-5-ynes.

All 4-Methoxy-1,2-dienyl-5-ynes **1a, 1b, 1c, 1d, 1e, 1f, 1g, 1h, 1k, $^{13}$C-1a, 6** and 3-disustituted-4-Methoxy-1,2-dienyl-5-ynes **4e** were prepared from the reported procedure in the literature. [S1]

### (c) Preparation of (3-methoxyhexa-4,5-dien-1-yn-1-yl)benzene ($d_2$-1a).

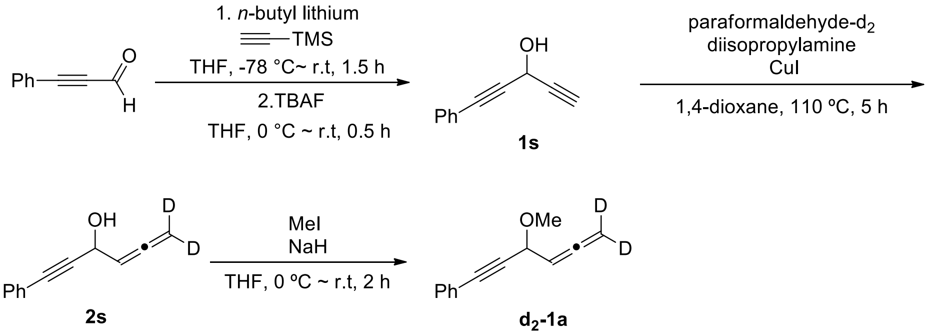


Compound **1s** was prepared from 3-phenylpropiolaldehyde according to a literature procedure. [S2]

To a dried flask was added CuI (0.3 g, 1.6 mmol), paraformaldehyde-$d_2$ (0.82 g, 25.6 mmol) and 1,4-dioxane (10 mL). The resulting mixture was stirred at room temperature before subsequent addition of $iPr_2NH$ (2.3 g, 22.4 mmol), **1s** (2.5 g, 16 mmol) and 1,4-dioxane (5 mL). The mixture was stirred at 110 °C without protection of an inert atmosphere. After 5 h, the crude reaction mixture was cooled to room temperature, filtered through a celite bed ($Et_2O$), and concentrated in vacuo. The residue was purified by column chromatography ($SiO_2$, eluent: EtOAc/hexane) to afford 1-phenylhexa-4,5-dien-1-yn-3-ol (**2s**) as brown oil (1.9 g, 11.2 mmol, 72%).

The compound **2s** (1.9 g, 11.03 mmol) was added to a stirred solution of NaH (0.318 g, 13.24 mmol) in THF (30 mL) at 0 °C, and the resulting mixture was stirred at 0 °C for 30 min. MeI (3.13 g, 22.1 mmol) was added, and the resulting mixture was stirred at rt for 2 h. The reaction was quenched with water and extracted with $Et_2O$. The organic layer was washed with water and brine, dried over MgSO4, and concentrated. The residue was purified by silica gel chromatography (SiO2, eluent: EtOAc/hexane) to afford (3-methoxyhexa-4,5-dien-1-yn-1-yl)benzene (**$d_2$-1a**) as brown oil (1.29 g, 6.95 mmol, 63%).

**(d) Preparation of (3-methoxy-4-methylhexa-4,5-dien-1-yn-1-yl)benzene ($^{13}$C-4e).**

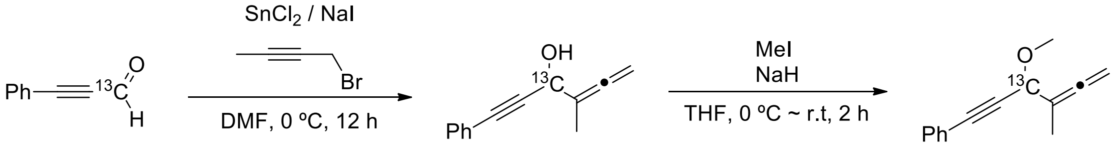


3s 4s 13C-4e

**3s** was prepared according to literature procedure[S1] and **4s** was prepared form the corresponding **3s** according to literature procedure. [S3]

The compound **4s** (2 g, 10.8mmol) was added to a stirred solution of NaH (0.310 g, 13.0 mmol) in THF (30 mL) at 0 °C and the resulting mixture was stirred at 0 °C for 30 min. MeI (3.06 g, 21.6 mmol) was added and the resulting mixture was stirred at rt for 2 h. The reaction was quenched with water and extracted with $Et_2O$. The organic layer was washed with water and brine, dried over MgSO4 and concentrated. The residue was purified by silica gel chromatography (SiO2, eluent: EtOAc/hexane) to afford (3-methoxy-4-methylhexa-4,5-dien-1-yn-1-yl)benzene (**$^{13}$C-4e**) as yellow oil (1.61 g, 8.12 mmol, 75%).

**(e) Preparation of isoxazoles.**

3-methyl isoxazole **2c** was prepared according to literature procedure.[S4] and Isoxazoles (**2d-2e**) were prepared according to literature procedure.[S5]


[S1] : H-C. Hsieh, K-C. Tan, A.S.K. Raj and R-S. Liu, *Chem. Commun.,* 2019, **55**, 1979.

[S2] : R. D. Kardile, B. S. Kale, P. Sharma and R.-S. Liu, *Org.Lett.,* 2018, **20**, 3806.

[S3] : D. Xu, Z. Li and S. Ma, *Tetrahedron Asymmetry* 2003, **14**, 3657.

[S4] : A. G. Griesbeck, M. Franke, J. Neudörfl and H. Kotaka, *Beilstein J. Org. Chem.*, 2011, **7**, 127.

[S5] : Y. He, Y.-Y. Xie, Y.-C. Wang, X.-M. Bin, D.-C. Hu, H.-S. Wang and Y.-M. Pan, *RSC. Adv.*, 2016, **6**, 58988.

## (2) Standard procedures for catalytic operations.

### (a) Typical procedure for the synthesis of 3-phenylindolizine-8-carbaldehyde (3a):

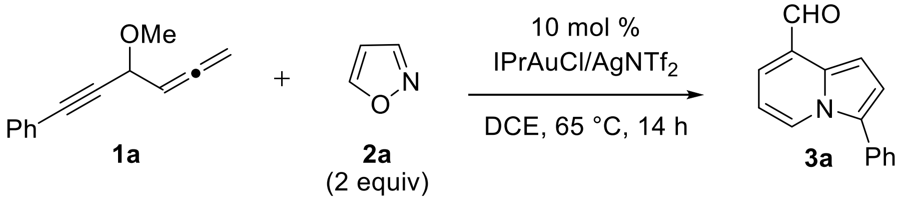


A suspension of IPrAuCl (33.7 mg, 0.054 mmol) and $AgNTf_2$ (21 mg, 0.054 mmol) in dry DCE (1 mL) was fitted with $N_2$ balloon and the mixture was stirred at 25 °C for 5 min. To this solution was added DCE (1 mL) solution of (3-methoxyhexa-4,5-dien-1-yn-1-yl)benzene **1a** (100 mg, 0.54 mmol) and isoxazole **2a** (75 mg, 1.08 mmol) at 25 °C. The resulting mixture was stirred for 14 h at 65 °C. The solution was filtered over a short celite bed and evaporated under reduced pressure. The residue was purified on a silica gel column using ethyl acetate/hexane (10:90) as eluent to give compound **3a** as yellow solid (106 mg, 0.48 mmol, 88%).

### (b) Typical procedure for the synthesis of 8-butyl-3-phenylindolizine-7-carbaldehyde (5a):

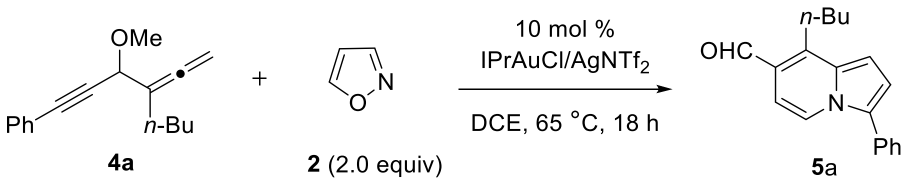


A suspension of IPrAuCl (25.8 mg, 0.042 mmol) and $AgNTf_2$ (16.1 mg, 0.042 mmol) in dry DCE (1 mL) was fitted with $N_2$ balloon and the mixture was stirred at 25 °C for 5 min. To this solution was added DCE (1 mL) solution of (3-methoxy-4-vinylideneoct-1-yn-1-yl)benzene **4a** (100 mg, 0.416 mmol) and isoxazole **2a** (57.4 mg, 0.832 mmol) at 25 °C. The resulting mixture was stirred for 18 h at 65 °C. The solution was filtered over a short celite bed and evaporated under reduced pressure. The residue was purified on a silica gel column using ethyl acetate/hexane (10:90) as eluent to give compound **5a** as yellow oil (87 mg, 0.313 mmol, 75%).

## (3) Mechanistic Investigation

### (a) Typical procedure for the synthesis of 3-phenylindolizine-8-carbaldehyde ($^{13}$C-3a):

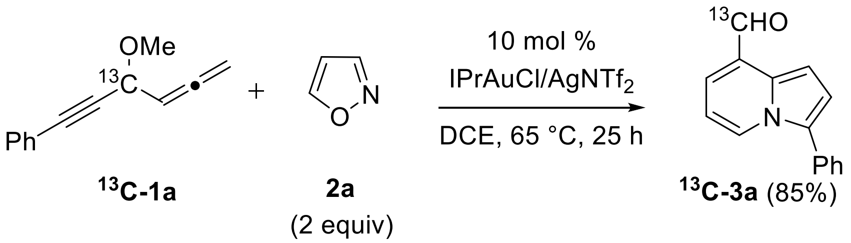


Experimental procedure according to standard procedure for catalytic opreration (**2-a**). Yields are reported after purification from silica column.

### (b) Typical procedure for the synthesis of 8-methyl-3-phenylindolizine-7-carbaldehyde ($^{13}$C-5e):

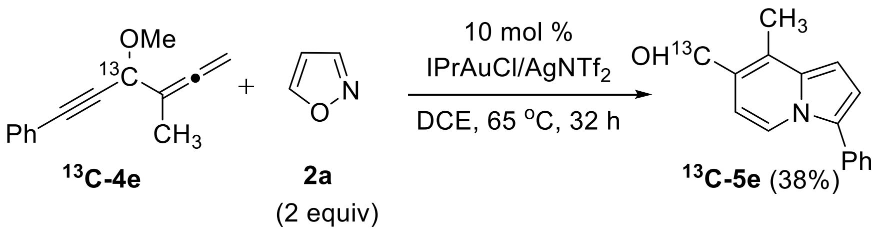


Experimental procedure according to standard procedure for catalytic opreration (**2-a**). Yields are reported after purification from silica column.

**(c) Typical procedure for the synthesis of 3-phenylindolizine-8-carbaldehyde ($d_2$-3a):**

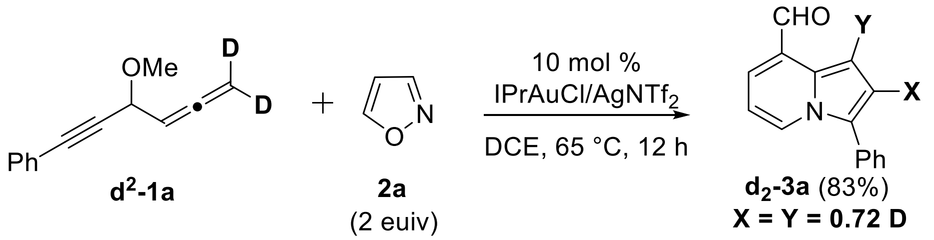


Experimental procedure according to standard procedure for catalytic opreration (**2-a**). Yields are reported after purification from silica column.

**(d) Cross-Over experiment**

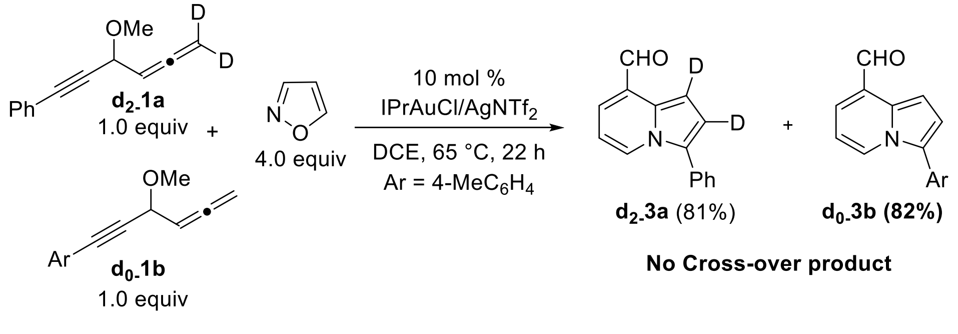


Experimental procedure according to standard procedure for catalytic opreration (**2-a**). Yields are reported after purification from silica column.

## (4) Computational details:

The B3LYP functional [S6-S8] combined with the LACVP** basis set (LanL2DZ for Au and 6-31G** for the others) was used for the geometry optimizations and zero-point vibrational energy (ZPVE). To obtain more accurate electronic energies, single point energy calculations using a larger basis set (LACV3P++**, LanL2TZ for Au, and 6-311++G** for the others) were performed on the B3LYP/LACVP** optimized structures. All calculations were performed under the influence of implicit solvent, which was simulated using the Poisson-Boltzmann self-consistent polarizable continuum method implemented in Jaguar to represent dichloroethane (dielectric constant = 10.65 and effective radius = 2.51 Å).[S9-S10] Enthalpies were calculated as

$$H_{298K} = E_{elec} + ZPVE + \sum_{v} \frac{hv}{e^{hv/kT} - 1} + 4kT$$

with T = 298K.


[S6] A. D. Becke, *Phys. Rev. A* 1988, **38**, 3098-3100.

[S7] A. D. Becke, *J. Chem. Phys.*, 1993, **98**, 5648-5652.

[S8] C. T. Lee, W. T. Yang, R. G. Parr, *Phys. Rev. B* 1988, **37**, 785-789.

[S9] D. J. Tannor, B. Marten, R. Murphy, R. A. Friesner, D. Sitkoff, A. Nicholls, M. Ringnalda, W. A. Goddard, B. Honig, *J. Am. Chem. Soc.*, 1994, **116**, 11875-11882.

[S10] B. Marten, K. Kim, C. Cortis, R. A. Friesner, R. B. Murphy, M. N. Ringnalda, D. Sitkoff, B. Honig, *J. Phys. Chem.*, 1996, **100**, 11775-11788.

## (5) Spectral data for compounds:

**(3-methoxyhexa-4,5-dien-1-yn-1-yl)cyclohexane (1i):**

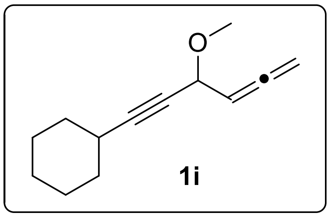


Yellow oil; $^1$H NMR (400 MHz, $CDCl_3$): δ 5.26 ~ 5.21 (m, 1H), 4.88 ~ 4.86 (m, 2H), 4.55 ~ 4.53 (m, 1H), 3.34 (s, 3H), 2.42 ~ 2.38 (m, 1H), 1.78 ~ 1.74 (m, 2H), 1.70 ~ 1.66 (m, 2H), 1.50 ~ 1.39 (m, 3H), 1.34 ~ 1.23 (m, 3H); $^{13}$C NMR (100 MHz, $CDCl_3$): δ 208.8, 91.6, 90.8, 77.3, 76.8, 69.4, 55.1, 32.5, 28.9, 25.8, 24.7; FI-MS calcd for $C_{13}H_{18}O[M^+]$: 190.1358; found : 190.1352.

**2-(3-methoxyhexa-4,5-dien-1-yn-1-yl)naphthalene (1j):**

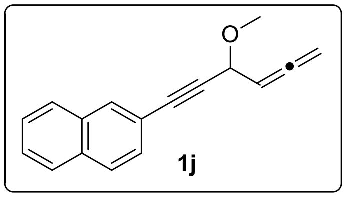


Brown oil; $^1$H NMR (400 MHz, $CDCl_3$): δ 7.97 (s, 1H), 7.80 ~ 7.75 (m, 3H), 7.49 ~ 7.45 (m, 3H), 5.39 (q, $J$ = 6.8 Hz, 1H), 4.97 (dd, $J$ = 6.8, 2.0 Hz, 2H), 4.85 ~ 4.82 (m, 1H), 3.48 (s, 3H); $^{13}$C NMR (100 MHz, $CDCl_3$): δ 209.0, 132.9, 132.8, 131.8, 128.4, 127.9, 127.7, 126.7, 126.5, 119.7, 90.4, 87.0, 86.3, 77.7, 69.8, 55.5, one CH carbon merged with other peaks; FI-MS calcd for $C_{17}H_{14}O[M^+]$: 234.1045; found : 234.1039.

**(3-methoxy-4-vinylideneoct-1-yn-1-yl)benzene (4a):**

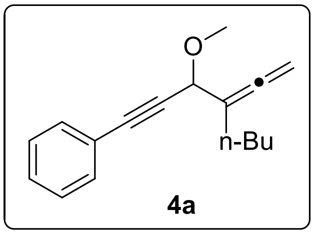


Yellow oil; $^1$H NMR (600 MHz, $CDCl_3$): δ 7.44 ~ 7.43 (m, 2H), 7.29 ~ 7.27 (m, 3H), 4.89 ~ 4.88 (m, 2H), 4.73 (s, 1H), 3.40 (s, 3H), 2.18 ~ 2.09 (m, 2H), 1.50 ~ 1.46 (m, 2H), 1.40 ~ 1.35 (m, 2H), 0.90 (t, $J$ = 7.2 Hz, 3H); $^{13}$C NMR (150 MHz, $CDCl_3$): δ 206.9, 131.8, 128.4, 128.2, 122.6, 102.6, 86.7, 86.1, 77.6, 73.1, 55.5, 29.7, 27.0, 22.4, 13.9; FI-MS calcd for $C_{17}H_{20}O[M^+]$: 240.1514; found : 240.1509.

**1-chloro-4-(3-methoxy-4-vinylideneoct-1-yn-1-yl)benzene (4b):**

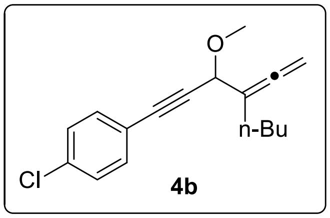


Brown oil; $^{1}$H NMR (400 MHz, $CDCl_3$): δ 7.37 ~ 7.34 (m, 2H), 7.27 ~ 7.25 (m, 2H), 4.89 ~ 4.87 (m, 2H), 4.71 (t, *J* = 1.6 Hz, 1H), 3.39 (s, 3H), 2.19 ~ 2.04 (m, 2H), 1.50 ~ 1.43 (m, 2H), 1.40 ~ 1.30 (m, 2H), 0.89 (t, *J* = 7.2 Hz, 3H); $^{13}$C NMR (100 MHz, $CDCl_3$): δ 206.8, 134.5, 132.9, 128.6, 121.1, 102.4, 87.1, 85.5, 77.7, 73.0, 55.6, 29.7, 26.9, 22.4, 13.9; FI-MS calcd for $C_{17}H_{19}ClO[M^+]$: 274.1124; found : 274.1119.

**1-bromo-4-(3-methoxy-4-vinylideneoct-1-yn-1-yl)benzene (4c):**

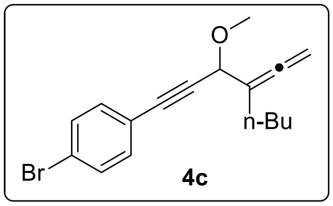


Brown oil; $^{1}$H NMR (400 MHz, $CDCl_3$): δ 7.45 ~ 7.40 (m, 2H), 7.30 ~ 7.28 (m, 2H), 4.89 ~ 4.87 (m, 2H), 4.70 (s, 1H), 3.38 (s, 3H), 2.18 ~ 2.03 (m, 2H), 1.63 ~ 1.43 (m, 2H), 1.33 ~ 1.26 (m, 2H), 0.89 (t, *J* = 7.2 Hz, 3H); $^{13}$C NMR (100 MHz, $CDCl_3$): δ 206.9, 133.2, 131.5, 131.2, 122.7, 121.6, 102.4, 87.3, 85.6, 77.7, 73.0, 55.6, 29.7, 27.0, 22.4, 13.9; FI-MS calcd for $C_{17}H_{19}BrO[M^+]$: 318.0619; found : 318.0614.

**2-(3-methoxy-4-vinylideneoct-1-yn-1-yl)naphthalene (4d):**

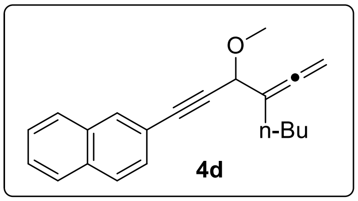


Brown oil; $^{1}$H NMR (400 MHz, $CDCl_3$): δ 7.97 (s, 1H), 7.81 ~ 7.75 (m, 3H),7.49 ~ 7.46 (m, 3H), 4.93 ~ 4.91 (m, 2H), 4.79 (t, *J* = 1.6 Hz, 1H), 3.44 (s, 3H), 2.26 ~ 2.09 (m, 2H), 1.55 ~ 1.47 (m, 2H), 1.44 ~ 1.35 (m, 2H), 0.93 (t, *J* = 7.2 Hz, 3H); $^{13}$C NMR (100 MHz, $CDCl_3$): δ 206.9, 132.9, 131.7, 128.5, 127.9, 127.7, 126.7, 126.5, 119.9, 102.6, 86.9, 86.4, 77.7, 73.2, 55.6, 29.7, 27.0, 22.4, 13.9, One quaternary carbon and one CH carbon merged with other peaks; FI-MS calcd for $C_{21}H_{22}O[M^+]$: 290.1671; found : 290.1665.

**5-methoxy-2-methyl-6-vinylidenedec-3-yne (4f):**

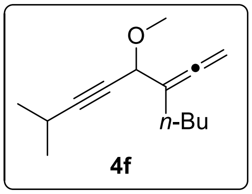


Brown oil; $^{1}H$ NMR (400 MHz, $CDCl_3$): δ 4.83 ~ 4.80 (m, 2H), 4.47 (q, $J$ = 1.6 Hz, 1H), 3.29 (s, 3H), 2.62 ~ 2.55 (m, 1H), 2.14 ~ 1.94 (m, 2H), 1.47 ~1.43 (m, 2H), 1.42 ~ 1.40 (m, 2H), 1.40 (d, $J$ = 7.2 Hz, 6H), 0.88 (t, $J$ = 7.2 Hz, 3H); $^{13}C$ NMR (100 MHz, $CDCl_3$): δ 206.7, 102.8, 92.9, 77.3, 76.0, 72.8, 55.3, 29.7, 26.7, 22.9, 22.4, 20.6, 13.9; FI-MS calcd for $C_{14}H_{22}O[M^+]$: 206.1671; found : 206.1665.

**7-methoxy-8-vinylidenedodec-5-yne (4g):**

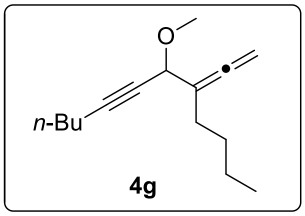


Yellow oil; $^{1}H$ NMR (400 MHz, $CDCl_3$): δ 4.83 ~ 4.80 (m, 2H), 4.47 (t, $J$ = 1.6 Hz, 1H), 3.30 (s, 3H), 2.24 ~ 2.20 (m, 2H), 2.13 ~ 1.95 (m, 2H), 1.52 ~ 1.28 (m, 8H), 0.90 ~ 0.86 (m, 6H); $^{13}C$ NMR (100 MHz, $CDCl_3$): δ 206.6, 102.8, 87.4, 77.3, 72.9, 55.3, 30.6, 29.6, 26.7, 22.4, 21.8, 18.4, 13.9, 13.5, One quaternary carbon merged with other peaks; FI-MS calcd for $C_{15}H_{24}O[M^+]$: 220.1827; found : 220.1822.

**(4-methoxydeca-1,2-dien-5-yn-3-yl)cyclopropane (4h):**

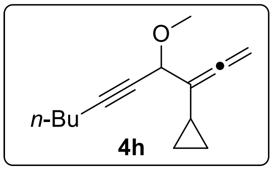


Yellow oil; $^{1}H$ NMR (400 MHz, $CDCl_3$): δ 4.85 (t, $J$ = 1.8 Hz, 2H), 4.58 ~ 4.56 (m, 1H), 3.34 (s, 3H), 2.25 ~ 2.21 (m, 2H), 1.49 ~ 1.44 (m, 2H), 1.43 ~ 1.37 (m, 2H), 1.33 ~ 1.29 (m, 1H), 0.89 (t, $J$ = 7.2 Hz, 3H), 0.72 ~ 0.67 (m, 2H), 0.47 ~ 0.38 (m, 2H); $^{13}C$ NMR (100 MHz, $CDCl_3$): δ 205.1, 106.4, 87.5, 78.6, 76.9, 72.6, 55.3, 30.6, 21.8, 18.4, 13.5, 8.1, 6.8, 6.7; FI-MS calcd for $C_{14}H_{20}O[M^+]$: 204.1514; found : 204.1509.

**3-isopropyl-4-methoxydeca-1,2-dien-5-yne (4i):**

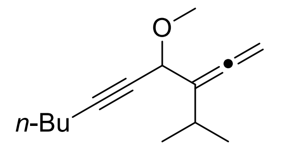

**4i**

Yellow oil; $^{1}$H NMR (400 MHz, $CDCl_3$): δ 4.86 (t, *J* = 1.6 Hz, 2H), 4.54 ~ 4.52 (m, 1H), 3.31 (s, 3H), 2.39 ~ 2.31 (m, 1H), 2.23 ~ 2.19 (m, 2H), 1.49 ~ 1.43 (m, 2H), 1.42 ~ 1.36 (m, 2H), 1.05 (t, *J* = 6.8 Hz, 6H), 0.87 (t, *J* = 7.2 Hz, 3H); $^{13}$C NMR (100 MHz, $CDCl_3$): δ 206.0, 109.2, 87.5, 78.5, 71.9, 55.2, 30.7, 27.0, 22.4, 22.3, 21.9, 18.4, 13.5, One quaternary carbon merged with other peaks; FI-MS calcd for $C_{14}H_{22}O[M^+]$: 206.1671; found : 206.1665.

**4-methoxy-3-methyldeca-1,2-dien-5-yne (4j):**

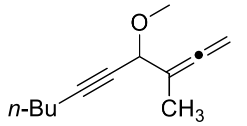

**4j**

Yellow oil; $^{1}$H NMR (400 MHz, $CDCl_3$): δ 4.71 (t, *J* = 2.0 Hz, 2H), 4.42 (d, *J* = 0.8 Hz, 1H), 3.28 (s, 3H), 2.22 ~ 2.18 (m, 2H), 1.70 (t, *J* = 3.2 Hz, 3H), 1.49 ~ 1.44 (m, 2H), 1.42 ~ 1.34 (m, 2H), 0.86 (t, *J* = 7.2 Hz, 3H); $^{13}$C NMR (100 MHz, $CDCl_3$): δ 206.9, 97.5, 87.3, 76.7, 75.5, 73.1, 55.3, 30.6, 21.8, 18.3, 13.6, 13.5, One quaternary carbon merged with other peaks; FI-MS calcd for $C_{12}H_{18}O[M+]$: 178.1358; found : 178.1352.

**3-phenylindolizine-8-carbaldehyde (3a):**

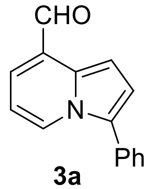

**3a**

Yellow solid; $^{1}$H NMR (600 MHz, $CDCl_3$): δ 10.03 (s, 1H), 8.44 (d, *J* = 7.2 Hz, 1H), 7.53 ~ 7.51 (m, 2H), 7.49 ~ 7.47 (m, 2H), 7.41 (dd, *J* = 4.2, 1.2 Hz, 1H), 7.39 ~ 7.36 (m, 1H), 7.31 (dd, *J* = 6.6, 0.6 Hz, 1H), 6.99 (d, *J* = 4.2 Hz, 1H), 6.64 (t, *J* = 6.6 Hz, 1H); $^{13}$C NMR (150 MHz, $CDCl_3$): δ 190.4, 131.7, 129.5, 129.1, 128.6, 128.4, 128.1, 127.8, 127.7, 126.6, 116.5, 109.2, 102.2; HRMS calcd for $C_{15}H_{11}NO$: 221.0841; found : 221.0845.

**$^{1}$H-NOE map of compound (3a).**

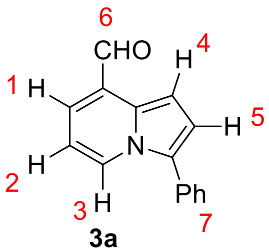


| Irradiation | Enhancement (%) |
|---|---|
| $H^2$ (δ 6.64) | $H^1$ (δ 7.31, 6.66%), $H^3$ (δ 8.44, 8.27%) |
| $H^1$ (δ 7.31) | $H^2$ (δ 6.64, 7.51%), $H^6$ (δ 10.03, 11.68%) |
| $H^4$ (δ 7.41) | $H^5$ (δ 6.99, 2.52%), $H^6$ (δ 10.03, 1.08%) |
| $H^7$ (δ 7.49 ~ 7.47) | $H^3$ (δ 8.44, 1.66%), $H^5$ (δ 6.99, 0.56%) |
| $H^6$ (δ 10.03) | $H^1$ (δ 7.31, 10.61%) |

**3-(*p*-tolyl)indolizine-8-carbaldehyde (3b):**

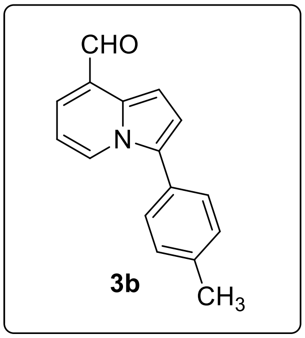


Yellow oil; $^1$H NMR (600 MHz, $CDCl_3$): δ 10.02 (s, 1H), 8.41 (d, $J$ = 7.1 Hz, 1H), 7.41 ~ 7.38 (m, 3H), 7.30 ~ 7.28 (m, 3H), 6.96 (d, $J$ = 3.9 Hz, 1H), 6.62 (t, $J$ = 6.9 Hz, 1H), 2.41 (s, 3H); $^{13}$C NMR (150 MHz, $CDCl_3$): δ 190.5, 137.7, 129.8, 129.4, 128.8, 128.5, 128.2, 127.6, 126.6, 116.2, 109.1, 102.1, 21.3, One quaternary carbon merged with other peaks; HRMS calcd for $C_{16}H_{13}NO$: 235.0997; found : 235.0992.

**3-(4-(*tert*-butyl)phenyl)indolizine-8-carbaldehyde (3c):**

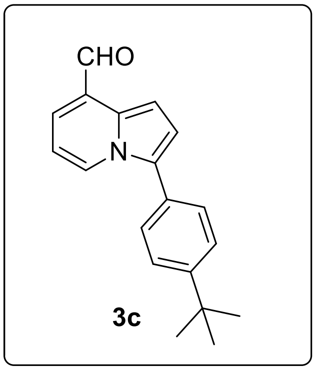


Brown solid; $^1$H NMR (400 MHz, $CDCl_3$): δ 10.02 (s, 1H), 8.45 (d, $J$ = 6.8 Hz, 1H), 7.50 (d, $J$ = 8.4 Hz, 2H), 7.45 (d, $J$ = 8.0 Hz, 2H), 7.40 (d, $J$ = 4.0 Hz, 1H), 7.29 (d, $J$ = 6.8 Hz, 1H), 6.98 (d,

$J$ = 3.6 Hz, 1H), 6.62 (t, $J$ = 6.8 Hz, 1H), 1.37 (s, 9H); $^{13}$C NMR (100 MHz, $CDCl_3$): δ 190.4, 150.9, 129.3, 128.7, 128.3, 127.6, 126.6, 125.9, 116.2, 109.0, 102.1, 34.7, 31.3, One quaternary carbon and CH carbon merged with other peaks; HRMS calcd for $C_{19}H_{19}NO$: 277.1467; found : 277.01472.

**3-(4-chlorophenyl)indolizine-8-carbaldehyde (3d):**

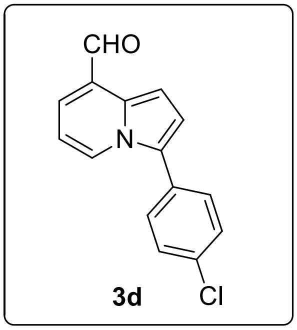


Brown solid; $^1$H NMR (600 MHz, $CDCl_3$): δ 10.03 (s, 1H), 8.37 (d, $J$ = 7.2 Hz, 1H), 7.45 (s, 4H), 7.40 (d, $J$ = 4.2 Hz, 1H), 7.33 (d, $J$ = 6.6 Hz, 1H), 6.97 (d, $J$ = 3.6 Hz, 1H), 6.67 (t, $J$ = 6.6 Hz, 1H); $^{13}$C NMR (150 MHz, $CDCl_3$): δ 190.4, 133.6, 130.2, 129.7, 129.5, 129.3, 128.7, 127.9, 127.7, 125.3, 116.7, 109.5, 102.4; HRMS calcd for $C_{15}H_{10}ClNO$: 255.0451; found : 255.0453.

**3-(4-bromophenyl)indolizine-8-carbaldehyde (3e):**

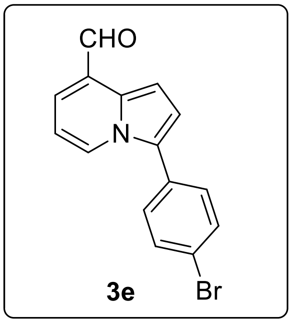


Brown oil; $^1$H NMR (400 MHz, $CDCl_3$): δ 10.02 (s, 1H), 8.44 (d, $J$ = 7.2 Hz, 1H), 7.54 ~ 7.46 (m, 3H), 7.41 ~ 7.35 (m, 2H), 7.31 (d, $J$ = 6.8 Hz, 1H), 6.99 (d, $J$ = 4.0 Hz, 1H), 6.64 (t, $J$ = 6.8 Hz, 1H); $^{13}$C NMR (100 MHz, $CDCl_3$): δ 190.4, 131.7, 129.5, 129.1, 128.5, 128.4, 128.1, 127.8, 127.7, 126.6, 116.4, 109.2, 102.2; HRMS calcd for $C_{15}H_{10}BrNO$: 298.9946; found : 298.9949.

**3-butylindolizine-8-carbaldehyde (3f):**

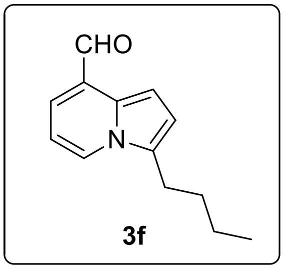

Brown oil; $^{1}$H NMR (600 MHz, $CDCl_3$): δ 9.99 (s, 1H), 7.98 (d, *J* = 7.2 Hz, 1H), 7.25 ~ 7.24 (m, 2H), 6.72 (d, *J* = 4.2 Hz, 1H), 6.66 (t, *J* = 6.6 Hz, 1H), 2.83 (t, *J* = 7.8 Hz, 2H), 1.76 ~ 1.71 (m, 2H), 1.47 ~ 1.41 (m, 2H), 0.96 (t, *J* = 7.2 Hz, 3H); $^{13}$C NMR (150 MHz, $CDCl_3$): δ 190.6, 128.2, 127.4, 127.1, 125.7, 114.1, 108.4, 100.7, 29.5, 25.5, 22.6, 13.9, One quaternary carbon merged with other peaks; HRMS calcd for $C_{13}H_{15}NO$: 201.1154; found : 201.1152.

**3-cyclopropylindolizine-8-carbaldehyde (3g):**

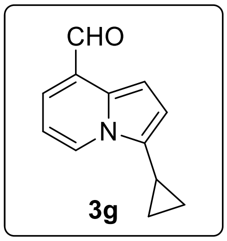

Brown oil; $^{1}$H NMR (400 MHz, $CDCl_3$): δ 9.99 (s, 1H), 8.32 (d, *J* = 7.2 Hz, 1H), 7.29 (d, *J* = 6.8 Hz, 1H), 7.19 (d, *J* = 4.0 Hz, 1H), 6.70 (t, *J* = 6.8 Hz, 1H), 6.66 (d, *J* = 3.6 Hz, 1H), 1.90 ~ 1.84 (m, 1H), 1.03 ~ 0.98 (m, 2H), 0.71 ~ 0.67 (m, 2H); $^{13}$C NMR (100 MHz, $CDCl_3$): δ 190.6, 128.9, 128.1, 127.3, 114.1, 108.5, 100.4, 6.1, 5.5, Two quaternary carbon merged with other peaks; HRMS calcd for $C_{12}H_{11}NO$: 185.0841; found : 185.0821.

**3-isopropylindolizine-8-carbaldehyde (3h):**

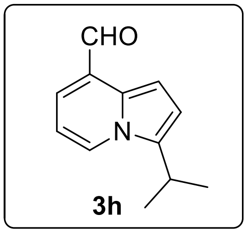

Brown oil; $^{1}$H NMR (400 MHz, $CDCl_3$): δ 9.96 (s, 1H), 7.99 (d, *J* = 7.2 Hz, 1H), 7.24 ~ 7.21 (m, 2H), 6.71 (d, *J* = 3.6 Hz, 1H), 6.64 (t, *J* = 6.7 Hz, 1H), 3.20 ~ 3.14 (m, 1H), 1.33 (d, *J* = 6.8 Hz, 6H); $^{13}$C NMR (100 MHz, $CDCl_3$): δ 190.6, 131.8, 128.2, 127.5, 127.3, 111.8, 108.4, 100.7, 25.2, 21.3, One quaternary carbon merged with other peaks; HRMS calcd for $C_{12}H_{13}NO$: 187.0997; found : 187.0995.

**3-cyclohexylindolizine-8-carbaldehyde (3i):**

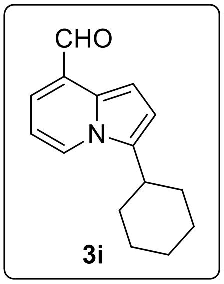


Brown oil; $^1$H NMR (400 MHz, $CDCl_3$): δ 9.99 (s, 1H), 8.04 (d, *J* = 6.8 Hz, 1H), 7.28 ~ 7.24 (m, 2H), 6.72 (d, *J* = 4.0 Hz, 1H), 6.66 (t, *J* = 7.2 Hz, 1H), 2.87 ~ 2.81 (m, 1H), 2.08 ~ 2.03 (m, 2H), 1.90 ~ 1.79 (m, 2H), 1.79 (d, *J* = 1.6 Hz, 1H), 1.49 ~ 1.44 (m, 4H), 1.41 ~ 1.35 (m, 1H); $^{13}$C NMR (100 MHz, $CDCl_3$): δ 190.6, 131.1, 128.3, 127.5, 127.1, 111.9, 108.3, 100.8, 35.2, 31.9, 26.5, 26.2, One quaternary carbon merged with other peaks; HRMS calcd for $C_{15}H_{17}NO$: 227.1310; found : 227.1314.

**3-(naphthalen-2-yl)indolizine-8-carbaldehyde (3j):**

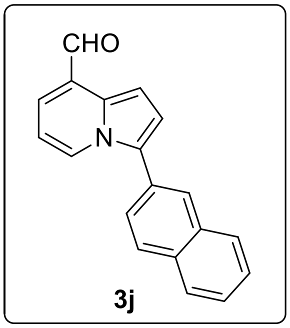


Yellow oil; $^1$H NMR (400 MHz, $CDCl_3$): δ 10.04 (s, 1H), 8.53 (d, *J* = 7.2 Hz,1H), 7.97 (s, 1H), 7.94 (d, *J* = 8.4 Hz, 1H), 7.87 (q, *J* = 3.6 Hz, 2H), 7.63 (dd, *J* = 8.4, 1.6 Hz, 1H), 7.55 ~ 7.48 (m, 2H), 7.46 (d, *J* = 4.0 Hz 1H), 7.33 (d, *J* = 6.4 Hz, 1H), 7.10 (d, *J* = 4.0 Hz, 1H), 6.66 (t, *J* = 7.2 Hz, 1H); $^{13}$C NMR (100 MHz, $CDCl_3$): δ 190.4, 133.6, 132.6, 129.5, 129.0, 128.8, 128.6, 128.1, 127.9, 127.8, 127.7, 127.1, 126.6, 126.5, 126.4, 126.3, 116.9, 109.3, 102.4; HRMS calcd for $C_{19}H_{13}NO$: 271.0997; found : 271.0996.

**7-methyl-3-phenylindolizine-8-carbaldehyde (3k):**

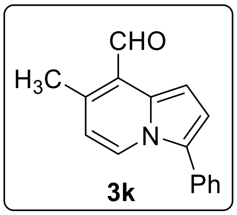

Yellow oil; $^1$H NMR (500 MHz, $CDCl_3$): δ 10.60 (s, 1H), 8.32 (d, *J* = 7.0 Hz, 1H), 7.50 ~ 7.45 (m, 4H), 7.36 ~ 7.34 (m, 2H), 6.93 (d, *J* = 4.0 Hz, 1H), 6.36 (d, *J* = 7.0 Hz, 1H), 2.65 (s, 3H); $^{13}$C NMR (125 MHz, $CDCl_3$): δ 189.0, 138.1, 131.9, 129.6, 129.0, 128.4, 127.7, 127.5, 125.7, 123.1, 116.3, 114.2, 101.2, 17.4; HRMS calcd for $C_{16}H_{13}NO$: 235.0997; found : 235.0999.

**3-(4-(*tert*-butyl)phenyl)-7-methylindolizine-8-carbaldehyde (3l):**

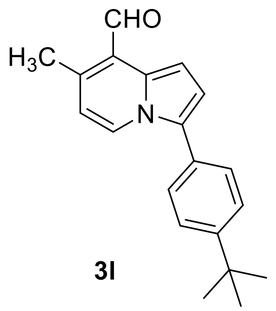

3l

Brown solid; $^1$H NMR (600 MHz, $CDCl_3$): δ 10.6 (s, 1H), 8.32 (d, *J* = 7.3 Hz, 1H), 7.49 ~ 7.47 (m, 2H), 7.44 ~ 7.42 (m, 2H), 7.33 (dd, *J* = 3.8 , 0.5 Hz , 1H), 6.91 (d, *J* = 3.6 Hz, 1H), 6.34 (d, *J* = 7.2 Hz, 1H), 2.65 (s, 3H), 1.36 (s, 9H); $^{13}$C NMR (150 MHz, $CDCl_3$): δ 188.9, 150.6, 137.8, 129.4, 129.3, 128.9, 128.2, 127.8, 125.9, 123.1, 116.1, 113.9, 101.0, 34.7, 31.3, 17.4; HRMS calcd for $C_{20}H_{21}NO$: 291.1623; found : 291.1628.

**5-methyl-3-phenylindolizine-8-carbaldehyde (3m):**

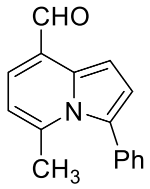

3m

Brown oil; $^1$H NMR (600 MHz, $CDCl_3$): δ 9.99 (s, 1H), 7.48 (d, *J* = 3.6 Hz, 1H), 7.40 ~ 7.35 (m, 5H), 7.26 (d, *J* = 6.6 Hz, 1H), 6.87 (d, *J* = 3.9 Hz, 1H), 6.42 (dd, *J* = 6.8 , 0.8 Hz , 1H), 2.17 (s, 3H); $^{13}$C NMR (150 MHz, $CDCl_3$): δ 190.1, 142.0, 135.2, 131.2, 129.8, 127.9, 127.5, 127.2, 125.7, 119.3, 111.1, 101.7, 23.6, One quaternary carbon merged with other peaks; ESI-MS calcd for $C_{16}H_{14}NO$[M+H]: 236.1075; found : 236.1079.

**$^1$H-NOE map of compound (3m).**

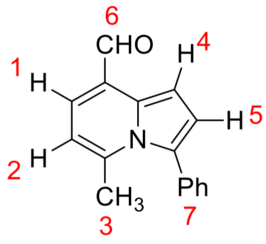


3m

| Irradiation | Enhancement (%) |
|---|---|
| $H^3$ (δ 2.17) | $H^2$ (δ 6.42, 3.53%), $H^7$ (δ 7.40 ~ 7.35, 2.19%), $H^5$ (δ 6.97, 0.76%) |
| $H^2$ (δ 6.42) | $H^3$ (δ 2.17, 5.12%), $H^1$ (δ 7.26, 10.59%) |
| $H^1$ (δ 7.26) | $H^2$ (δ 6.42, 8.82%), $H^6$ (δ 9.99, 12.36%)) |
| $H^6$ (δ 9.99) | $H^1$ (δ 7.26, 12.37%) |

**3-(4-chlorophenyl)-5-methylindolizine-8-carbaldehyde (3n):**

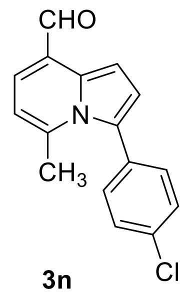


3n

Orange oil; $^1$H NMR (600 MHz, $CDCl_3$): δ 9.99 (s, 1H), 7.48 (d, *J* = 4.0 Hz, 1H), 7.35 ~ 7.31 (m, 4H), 7.27 (d, *J* = 4.0 Hz, 1H), 6.85 (d, *J* = 4.0 Hz, 1H), 6.44 (dd, *J* = 6.8, 0.8 Hz, 1H), 2.19 (s, 3H); $^{13}$C NMR (150 MHz, $CDCl_3$): δ 190.0, 141.7, 134.1, 133.8, 132.2, 130.3, 129.7, 127.5, 126.0, 125.9, 119.6, 111.4, 101.9, 23.7; ESI-MS calcd. for $C_{16}H_{13}ClNO$[M+H]: 270.0686; found: 270.0684.

**3-butyl-5-methylindolizine-8-carbaldehyde (3o):**

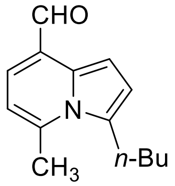


3o

Orange oil; $^1$H NMR (600 MHz, $CDCl_3$): δ 9.90 (s, 1H), 7.32 (d, *J* = 4.0 Hz, 1H), 7.11 (d, *J* = 6.8 Hz, 1H), 6.70 (d, *J* = 4.0 Hz, 1H), 6.35 (dd, *J* = 6.8, 0.8 Hz, 1H), 3.20 (t, *J* = 7.4 Hz, 2H), 2.88 (s, 3H), 1.71 ~ 1.66 (m, 2H), 1.46 ~ 1.41 (m, 2H), 0.95 (t, *J* = 7.4 Hz, 3H); $^{13}$C NMR (150 MHz, $CDCl_3$): δ 190.0, 141.6, 129.9, 129.1, 128.5, 125.9, 116.6, 111.0, 101.3, 33.2, 29.6, 22.4, 22.3, 13.9; ESI-MS calcd. for $C_{14}H_{18}NO$[M+H]: 216.1388; found: 216.1382.

**8-butyl-3-phenylindolizine-7-carbaldehyde (5a):**

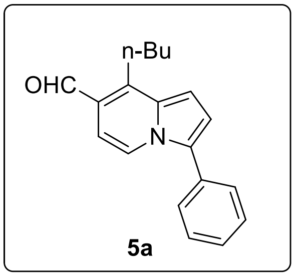


Yellow oil; $^{1}H$ NMR (600 MHz, $CDCl_3$): δ 10.28 (s, 1H), 8.07 (d, *J* = 7.4 Hz, 1H), 7.56 ~ 7.54 (m, 2H), 7.48 (t, *J* = 7.9 Hz, 2H), 7.39 (q, *J* = 7.4 Hz, 1H), 7.02 (d, *J* = 7.4 Hz, 1H), 6.99 (d, *J* = 4.3 Hz, 1H), 6.92 (d, *J* = 4.3 Hz, 1H), 3.23 (t, *J* = 7.9 Hz, 2H), 1.81 ~ 1.76 (m, 2H), 1.52 ~ 1.39 (m, 2H), 0.97 (t, *J* = 7.4 Hz, 3H); $^{13}C$ NMR (150 MHz, $CDCl_3$): δ 187.9, 141.8, 133.0, 131.5, 130.0, 129.1, 128.4, 128.1, 122.1, 120.3, 115.7, 108.0, 105.7, 33.9, 27.0, 23.1, 13.9; HRMS calcd for $C_{19}H_{19}NO$: 277.1467; found : 277.1492.

**8-butyl-3-(4-chlorophenyl)indolizine-7-carbaldehyde (5b):**

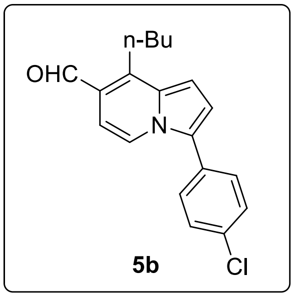


Brown solid; $^{1}H$ NMR (400 MHz, $CDCl_3$): δ 10.27 (s, 1H), 7.99 (d, *J* = 7.6 Hz, 1H), 7.48 ~ 7.43 (m, 4H), 7.03 (d, *J* = 7.6 Hz, 1H), 6.98 (d, *J* = 4.4 Hz, 1H), 6.89 (d, *J* = 4.4 Hz, 1H), 3.21 (t, *J* = 7.6 Hz, 2H), 1.81 ~ 1.73 (m, 2H), 1.52 ~ 1.43 (m, 2H), 0.96 (t, *J* = 7.6 Hz, 3H); $^{13}C$ NMR (100 MHz, $CDCl_3$): δ 187.8, 141.6, 133.8, 133.2, 129.8, 129.4, 129.3, 128.5, 122.2, 119.9, 115.8, 108.2, 105.7, 33.8, 26.9, 23.0, 13.8; HRMS calcd for $C_{19}H_{18}ClNO$: 311.1077; found : 311.1074.

**3-(4-bromophenyl)-8-butylindolizine-7-carbaldehyde (5c):**

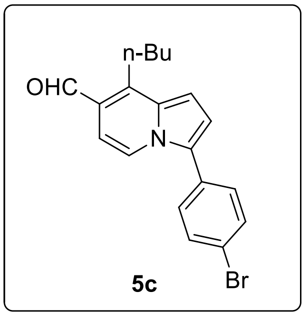

Yellow oil; $^1$H NMR (400 MHz, $CDCl_3$): δ 10.28 (s, 1H), 8.00 (d, *J* = 7.6 Hz, 1H), 7.62 ~ 7.59 (m, 2H), 7.43 ~ 7.39 (m, 2H), 7.04 (d, *J* = 7.6 Hz, 1H), 6.98 (d, *J* = 4.0 Hz, 1H), 6.90 (d, *J* = 4.0 Hz, 1H), 3.22 (t, *J* = 7.6 Hz, 2H), 1.81 ~ 1.73 (m, 2H), 1.51 ~ 1.45 (m, 2H), 0.97 (t, *J* = 7.2 Hz, 3H); $^{13}$C NMR (100 MHz, $CDCl_3$): δ 187.9, 141.7, 133.3, 132.3, 130.3, 129.7, 128.6, 122.3, 121.9, 120.1, 115.8, 108.3, 105.7, 33.8, 26.9, 23.1, 13.8; HRMS calcd for $C_{19}H_{18}BrNO$: 355.0572; found : 355.0578.

**8-butyl-3-(naphthalen-2-yl)indolizine-7-carbaldehyde (5d):**

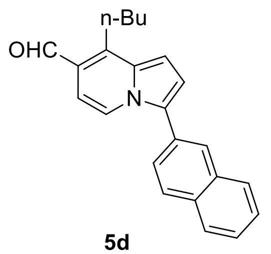


**5d**

Yellow oil; $^1$H NMR (400 MHz, $CDCl_3$): δ 10.29 (s, 1H), 8.17 (d, *J* = 7.2 Hz, 1H), 8.01 (s, 1H), 7.95 (d, *J* = 8.8 Hz, 1H), 7.89 ~ 7.86 (m, 2H), 7.65 (dd, *J* = 8.4, 1.6 Hz, 1H), 7.53 ~ 7.50 (m, 2H), 7.07 ~ 7.03 (m, 3H), 3.26 (t, *J* = 8.0 Hz, 2H), 1.84 ~ 1.76 (m, 2H), 1.58 ~ 1.47 (m, 2H), 0.98 (t, *J* = 7.2 Hz, 3H); $^{13}$C NMR (100 MHz, $CDCl_3$): δ 187.9, 141.8, 133.5, 133.2, 132.8, 130.0, 128.8, 128.0, 127.8, 127.1, 126.7, 126.5, 126.1, 122.3, 120.3, 116.1, 108.2, 105.9, 33.9, 27.0, 23.1, 13.9, One quaternary carbon merged with other peaks; HRMS calcd for $C_{23}H_{21}NO$: 327.1623; found : 327.1624.

**8-methyl-3-phenylindolizine-7-carbaldehyde (5e):**

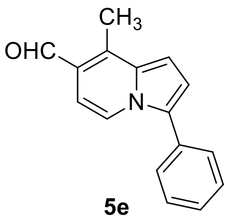


**5e**

Brown oil; $^1$H NMR (400 MHz, $CDCl_3$): δ 10.30 (s, 1H), 8.08 (d, *J* = 7.2 Hz, 1H), 7.56 ~ 7.50 (m, 2H), 7.50 ~ 7.46 (m, 2H), 7.41 ~ 7.36 (m, 1H), 7.01 (d, *J* = 7.2 Hz, 1H), 6.99 (dd, *J* = 4.4, 0.8 Hz, 1H), 6.93 (d, *J* = 4.4 Hz, 1H), 2.79 (s, 3H); $^{13}$C NMR (100 MHz, $CDCl_3$): δ 188.1, 136.4, 133.4, 131.4, 130.1, 129.1, 128.3, 128.1, 122.6, 120.2, 115.7, 107.9, 105.9, 12.7; HRMS calcd for $C_{16}H_{13}NO$: 235.0997; found : 235.0996.

**8-butyl-3-isopropylindolizine-7-carbaldehyde (5f):**

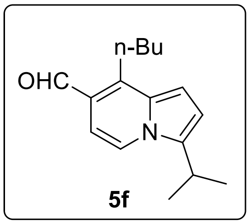

Yellow oil; $^{1}H$ NMR (400 MHz, $CDCl_3$): δ 10.25 (s, 1H), 7.63 (d, *J* = 7.2 Hz, 1H), 7.06 (d, *J* = 7.2 Hz, 1H), 6.88 (d, *J* = 4.4 Hz, 1H), 6.67 (d, *J* = 4.0 Hz, 1H), 3.22 ~ 3.15 (m, 3H), 1.78 ~ 1.70 (m, 2H), 1.49 ~ 1.40 (m, 2H), 1.34 (d, *J* = 6.8 Hz, 6H), 0.94 (t, *J* = 7.2 Hz, 3H); $^{13}C$ NMR (100 MHz, $CDCl_3$): δ 188.0, 141.7, 135.8, 131.8, 120.8, 119.5, 111.2, 107.2, 104.7, 33.9, 26.9, 25.3, 23.0, 21.0, 13.8; HRMS calcd for $C_{16}H_{21}NO$: 243.1623; found : 243.1621.

**3,8-dibutylindolizine-7-carbaldehyde (5g):**

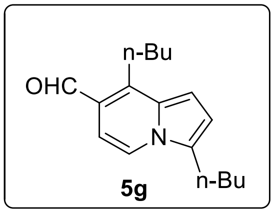

Brown oil; $^{1}H$ NMR (400 MHz, $CDCl_3$): δ 10.26 (s, 1H), 7.59 (d, *J* = 7.2 Hz, 1H), 7.06 (d, *J* = 7.6 Hz, 1H), 6.87 (d, *J* = 4.4 Hz, 1H), 6.66 (d, *J* = 4 Hz, 1H), 3.18 (t, *J* = 8.0 Hz, 2H), 2.80 (t, *J* = 7.6 Hz, 2H), 1.78 ~ 1.64 (m, 4H), 1.49 ~ 1.39 (m, 4H), 0.95 (q, *J* = 6.9 Hz, 6H); $^{13}C$ NMR (100 MHz, $CDCl_3$): δ 188.1, 141.6, 131.8, 129.9, 120.8, 119.5, 113.7, 107.4, 104.6, 33.9, 29.3, 26.9, 25.7, 23.1, 22.6, 13.8; HRMS calcd for $C_{17}H_{23}NO$: 257.1780; found : 257.1785.

**3-butyl-8-cyclopropylindolizine-7-carbaldehyde (5h):**

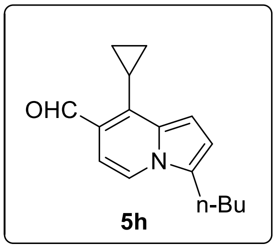

Brown oil; $^{1}H$ NMR (400 MHz, $CDCl_3$): δ 10.69 (s, 1H), 7.61 (d, *J* = 7.2 Hz, 1H), 7.05 (t, *J* = 7.2 Hz, 2H), 6.66 (d, *J* = 4.0 Hz, 1H), 2.80 (t, *J* = 7.6 Hz, 2H), 2.22 ~ 2.15 (m, 1H), 1.75 ~ 1.68 (m, 2H), 1.47 ~ 1.38 (m, 2H), 1.21 ~ 1.17 (m, 2H), 0.96 (t, *J* = 7.3 Hz, 3H), 0.88 ~ 0.84 (m, 2H); $^{13}C$ NMR (100 MHz, $CDCl_3$): δ 189.5, 140.6, 132.1, 129.6, 123.5, 120.0, 113.8, 106.7, 106.0, 29.3, 25.7, 22.5, 13.8, 8.9, 7.2; HRMS calcd for $C_{16}H_{19}NO$: 241.1467; found : 241.1470.

**3-butyl-8-isopropylindolizine-7-carbaldehyde (5i):**

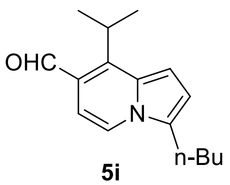


**5i**

Yellow oil; $^{1}H$ NMR (400 MHz, $CDCl_3$): δ 10.45 (s, 1H), 7.59 (d, *J* = 7.6 Hz, 1H), 7.11 (d, *J* = 7.6 Hz, 1H), 6.97 (d, *J* = 4.4 Hz, 1H), 6.66 (d, *J* = 4.0 Hz, 1H), 4.05 (bs, 1H), 2.79 (t, *J* = 7.6 Hz, 2H), 1.76 ~ 1.68 (m, 2H), 1.57 (d, *J* = 7.2 Hz, 6H), 1.48 ~ 1.39 (m, 2H), 0.96 (t, *J* = 7.6 Hz, 3H); $^{13}C$ NMR (100 MHz, $CDCl_3$): δ 188.1, 146.8, 130.0, 129.4, 121.1, 119.5, 113.5, 107.7, 106.4, 29.2, 28.0, 25.8, 23.0, 22.6, 13.8; HRMS calcd for $C_{16}H_{21}NO$: 243.1623; found : 243.1622.

**3-butyl-8-methylindolizine-7-carbaldehyde (5j):**

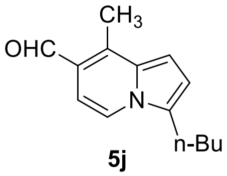


**5j**

Brown solid; $^{1}H$ NMR (400 MHz, $CDCl_3$): δ 10.27 (s, 1H), 7.60 (d, *J* = 7.2 Hz, 1H), 7.04 (d, *J* = 7.6 Hz, 1H), 6.86 (d, *J* = 4.0 Hz, 1H), 6.66 (d, *J* = 4.0 Hz, 1H), 2.81 (t, *J* = 7.2 Hz, 2H), 2.74 (s, 3H), 1.76 ~ 1.67 (m, 2H), 1.47 ~ 1.38 (m, 2H), 0.96 (t, *J* = 7.2 Hz, 3H); $^{13}C$ NMR (100 MHz, $CDCl_3$): δ 188.3, 136.2, 132.1, 130.0, 121.3, 119.4, 113.7, 107.3, 104.9, 29.3, 25.7, 22.5, 13.8, 12.6; HRMS calcd for $C_{14}H_{17}NO$: 215.1310; found : 215.1315.

**ethyl 3-cyclopropyl-8-formyl-7-phenylindolizine-1-carboxylate (7a):**

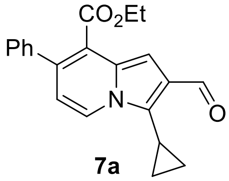


**7a**

Yellow oil; $^{1}H$ NMR (600 MHz, $CDCl_3$): δ 10.42 (s, 1H), 8.22 (dd, *J* = 7.4, 0.9 Hz, 1H), 7.41 ~ 7.34 (m, 5H), 7.01 (d, *J* = 0.7 Hz, 1H), 6.71 (d, *J* =7.4 Hz, 1H), 4.16 (q, *J* = 7.1 Hz, 2H), 2.00 ~ 1.96 (m, 1H), 1.26 ~1.21 (m, 2H), 1.04 (t, *J* = 7.1 Hz, 3H), 0.83 ~ 0.81 (m, 2H); $^{13}C$ NMR (150 MHz, $CDCl_3$): δ 187.5, 166.6, 139.3, 133.1, 131.0, 129.6, 128.4, 128.0, 127.9, 127.1, 124.5,

122.9, 114.9, 99.1, 61.4, 13.7, 6.4, 4.4; ESI-MS calcd. for $C_{21}H_{20}NO_3$[M+H]: 334.1443; found: 334.1451.

**ethyl 7-(4-chlorophenyl)-3-cyclopropyl-8-formylindolizine-1-carboxylate (7b):**

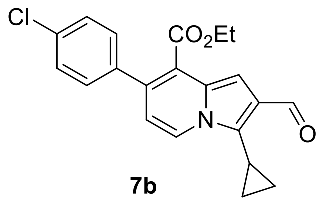


**7b**

Yellow solid; mp: 34.1 ~ 35.3 ºC; $^1$H NMR (600 MHz, $CDCl_3$): δ 10.41 (s, 1H), 8.23 ~ 8.22 (m, 1H), 7.37 (dd, *J* = 8.6 Hz, 2H), 7.28 (d, *J* = 8.6 Hz, 2H), 7.02 (s, 1H), 6.65 (d, *J* =7.4 Hz, 1H), 4.19 (q, *J* = 7.1 Hz, 2H), 2.00 ~ 1.95 (m, 1H), 1.27 ~1.24 (m, 2H), 1.10 (t, *J* = 7.1 Hz, 3H), 0.83 ~ 0.80 (m, 2H); $^{13}$C NMR (150 MHz, $CDCl_3$): δ 187.4, 166.3, 137.8, 134.0, 131.8, 131.1, 129.4, 128.6, 127.2, 124.7, 124.2, 123.0, 114.5, 99.6, 61.6, 13.8, 6.4, 4.5; ESI-MS calcd. for $C_{21}H_{20}NO_3$[M+H]: 368.1054; found: 368.1048.

**(3-methoxyhexa-4,5-dien-1-yn-1-yl)benzene ($d_2$-1a):**

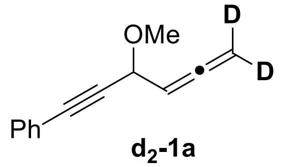


**$d_2$-1a**

Brown oil; $^1$H NMR (400 MHz, $CDCl_3$): δ 7.46 ~ 7.43 (m, 2H), 7.31 ~ 7.27 (m, 3H), 5.36 (d, *J* = 6.8 Hz, 1H), 4.79 (d, *J* = 7.2 Hz, 1H), 3.45 (s, 3H); $^{13}$C NMR (100 MHz, $CDCl_3$): δ 209.0, 131.8, 128.5, 128.2, 122.4, 90.5, 86.7, 85.9, 69.7, 55.5; ESI-MS calcd for $C_{13}H_{11}D_2O$[M+H]: 187.1092; found : 187.1086.

## (6) X-ray crystallographic structure and data for compound 3c, 3d, 3l, 5b, 5j and 7b:

### (A) X-ray crystallographic data of compound (3c).

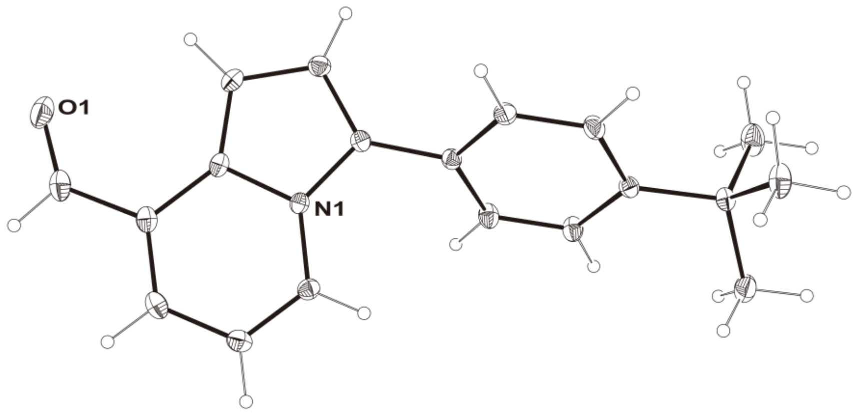


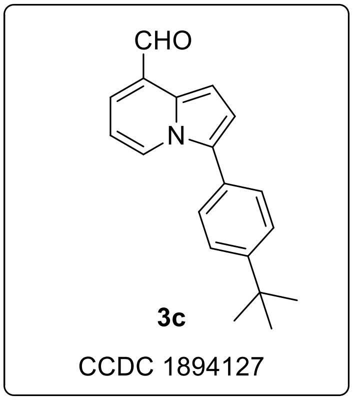


Table S1. Crystal data and structure refinement for d19599.

| | | |
|---|---|---|
| Identification code | d19599 | |
| Empirical formula | C19 H19 N O | |
| Formula weight | 277.35 | |
| Temperature | 200(2) K | |
| Wavelength | 0.71073 Å | |
| Crystal system | Monoclinic | |
| Space group | P 21/n | |
| Unit cell dimensions | a = 9.2570(3) Å | α= 90°. |
| | b = 14.9320(3) Å | β= 109.5890(10)°. |

| | | |
|---|---|---|
| | c = 11.4194(3) Å | $\gamma$ = 90°. |
| Volume | 1487.09(7) Å$^3$ | |
| Z | 4 | |
| Density (calculated) | 1.239 Mg/m$^3$ | |
| Absorption coefficient | 0.076 mm$^{-1}$ | |
| F(000) | 592 | |
| Crystal size | 0.58 x 0.52 x 0.38 mm$^3$ | |
| Theta range for data collection | 2.46 to 25.06°. | |
| Index ranges | -11<=h<=10, -17<=k<=17, -13<=l<=13 | |
| Reflections collected | 16035 | |
| Independent reflections | 2600 [R(int) = 0.0458] | |
| Completeness to theta = 25.06° | 98.5 % | |
| Absorption correction | multi-scan | |
| Max. and min. transmission | 0.9717 and 0.9573 | |
| Refinement method | Full-matrix least-squares on F$^2$ | |
| Data / restraints / parameters | 2600 / 0 / 194 | |
| Goodness-of-fit on F$^2$ | 1.019 | |
| Final R indices [I>2sigma(I)] | R1 = 0.0399, wR2 = 0.0977 | |
| R indices (all data) | R1 = 0.0460, wR2 = 0.1028 | |
| Extinction coefficient | 0.035(10) | |
| Largest diff. peak and hole | 0.215 and -0.182 e.Å$^{-3}$ | |

Table S2. Atomic coordinates ( x $10^4$) and equivalent isotropic displacement parameters ($Å^2$x $10^3$) for d19599. U(eq) is defined as one third of the trace of the orthogonalized $U^{ij}$ tensor.

| | x | y | z | U(eq) |
|---|---|---|---|---|
| C(1) | 5490(2) | 1571(1) | 3302(2) | 42(1) |
| C(2) | 5656(2) | 2528(1) | 3604(1) | 31(1) |
| C(3) | 6160(2) | 2782(1) | 4823(1) | 36(1) |
| C(4) | 6313(2) | 3697(1) | 5159(1) | 35(1) |
| C(5) | 5952(2) | 4331(1) | 4264(1) | 29(1) |
| C(6) | 5265(1) | 3190(1) | 2645(1) | 27(1) |
| C(7) | 4688(2) | 3187(1) | 1371(1) | 31(1) |
| C(8) | 4472(2) | 4082(1) | 974(1) | 31(1) |
| C(9) | 4934(1) | 4638(1) | 1989(1) | 26(1) |
| C(10) | 4911(2) | 5623(1) | 2063(1) | 26(1) |
| C(11) | 6206(2) | 6125(1) | 2689(1) | 33(1) |
| C(12) | 6149(2) | 7051(1) | 2706(1) | 33(1) |
| C(13) | 4805(1) | 7522(1) | 2102(1) | 26(1) |
| C(14) | 4776(2) | 8549(1) | 2073(1) | 28(1) |
| C(15) | 5902(2) | 8940(1) | 3266(2) | 51(1) |
| C(16) | 5237(2) | 8857(1) | 975(2) | 51(1) |
| C(17) | 3188(2) | 8918(1) | 1921(2) | 46(1) |
| C(18) | 3520(2) | 7013(1) | 1475(1) | 34(1) |
| C(19) | 3568(2) | 6087(1) | 1459(1) | 33(1) |
| N(1) | 5438(1) | 4095(1) | 3030(1) | 24(1) |
| O(1) | 5024(1) | 1252(1) | 2273(1) | 53(1) |

Table S3. Bond lengths [Å] and angles [°] for d19599.

| | |
|---|---|
| C(1)-O(1) | 1.205(2) |
| C(1)-C(2) | 1.466(2) |
| C(1)-H(1) | 0.9500 |
| C(2)-C(3) | 1.365(2) |
| C(2)-C(6) | 1.4287(19) |
| C(3)-C(4) | 1.413(2) |
| C(3)-H(3) | 0.9500 |
| C(4)-C(5) | 1.3495(19) |
| C(4)-H(4) | 0.9500 |
| C(5)-N(1) | 1.3738(17) |
| C(5)-H(5) | 0.9500 |
| C(6)-C(7) | 1.371(2) |
| C(6)-N(1) | 1.4133(16) |
| C(7)-C(8) | 1.4045(19) |
| C(7)-H(7) | 0.9500 |
| C(8)-C(9) | 1.3715(19) |
| C(8)-H(8) | 0.9500 |
| C(9)-N(1) | 1.3849(16) |
| C(9)-C(10) | 1.4733(18) |
| C(10)-C(19) | 1.3879(19) |
| C(10)-C(11) | 1.3917(19) |
| C(11)-C(12) | 1.3840(19) |
| C(11)-H(11) | 0.9500 |
| C(12)-C(13) | 1.3940(19) |
| C(12)-H(12) | 0.9500 |
| C(13)-C(18) | 1.3906(19) |
| C(13)-C(14) | 1.5345(18) |
| C(14)-C(16) | 1.524(2) |
| C(14)-C(17) | 1.5242(19) |
| C(14)-C(15) | 1.526(2) |
| C(15)-H(15A) | 0.9800 |
| C(15)-H(15B) | 0.9800 |
| C(15)-H(15C) | 0.9800 |
| C(16)-H(16A) | 0.9800 |

| | |
|---|---|
| C(16)-H(16B) | 0.9800 |
| C(16)-H(16C) | 0.9800 |
| C(17)-H(17A) | 0.9800 |
| C(17)-H(17B) | 0.9800 |
| C(17)-H(17C) | 0.9800 |
| C(18)-C(19) | 1.3844(19) |
| C(18)-H(18) | 0.9500 |
| C(19)-H(19) | 0.9500 |

| | |
|---|---|
| O(1)-C(1)-C(2) | 126.15(15) |
| O(1)-C(1)-H(1) | 116.9 |
| C(2)-C(1)-H(1) | 116.9 |
| C(3)-C(2)-C(6) | 120.05(12) |
| C(3)-C(2)-C(1) | 119.00(13) |
| C(6)-C(2)-C(1) | 120.93(13) |
| C(2)-C(3)-C(4) | 120.97(13) |
| C(2)-C(3)-H(3) | 119.5 |
| C(4)-C(3)-H(3) | 119.5 |
| C(5)-C(4)-C(3) | 119.70(13) |
| C(5)-C(4)-H(4) | 120.1 |
| C(3)-C(4)-H(4) | 120.1 |
| C(4)-C(5)-N(1) | 120.65(12) |
| C(4)-C(5)-H(5) | 119.7 |
| N(1)-C(5)-H(5) | 119.7 |
| C(7)-C(6)-N(1) | 107.20(11) |
| C(7)-C(6)-C(2) | 135.99(12) |
| N(1)-C(6)-C(2) | 116.77(12) |
| C(6)-C(7)-C(8) | 107.58(12) |
| C(6)-C(7)-H(7) | 126.2 |
| C(8)-C(7)-H(7) | 126.2 |
| C(9)-C(8)-C(7) | 109.47(12) |
| C(9)-C(8)-H(8) | 125.3 |
| C(7)-C(8)-H(8) | 125.3 |
| C(8)-C(9)-N(1) | 106.87(11) |
| C(8)-C(9)-C(10) | 130.26(12) |
| N(1)-C(9)-C(10) | 122.86(11) |

| | |
|---|---|
| C(19)-C(10)-C(11) | 117.40(12) |
| C(19)-C(10)-C(9) | 119.82(12) |
| C(11)-C(10)-C(9) | 122.75(12) |
| C(12)-C(11)-C(10) | 121.03(12) |
| C(12)-C(11)-H(11) | 119.5 |
| C(10)-C(11)-H(11) | 119.5 |
| C(11)-C(12)-C(13) | 121.91(12) |
| C(11)-C(12)-H(12) | 119.0 |
| C(13)-C(12)-H(12) | 119.0 |
| C(18)-C(13)-C(12) | 116.56(12) |
| C(18)-C(13)-C(14) | 121.95(11) |
| C(12)-C(13)-C(14) | 121.40(11) |
| C(16)-C(14)-C(17) | 108.82(13) |
| C(16)-C(14)-C(15) | 108.88(14) |
| C(17)-C(14)-C(15) | 107.90(13) |
| C(16)-C(14)-C(13) | 108.16(11) |
| C(17)-C(14)-C(13) | 111.88(11) |
| C(15)-C(14)-C(13) | 111.15(11) |
| C(14)-C(15)-H(15A) | 109.5 |
| C(14)-C(15)-H(15B) | 109.5 |
| H(15A)-C(15)-H(15B) | 109.5 |
| C(14)-C(15)-H(15C) | 109.5 |
| H(15A)-C(15)-H(15C) | 109.5 |
| H(15B)-C(15)-H(15C) | 109.5 |
| C(14)-C(16)-H(16A) | 109.5 |
| C(14)-C(16)-H(16B) | 109.5 |
| H(16A)-C(16)-H(16B) | 109.5 |
| C(14)-C(16)-H(16C) | 109.5 |
| H(16A)-C(16)-H(16C) | 109.5 |
| H(16B)-C(16)-H(16C) | 109.5 |
| C(14)-C(17)-H(17A) | 109.5 |
| C(14)-C(17)-H(17B) | 109.5 |
| H(17A)-C(17)-H(17B) | 109.5 |
| C(14)-C(17)-H(17C) | 109.5 |
| H(17A)-C(17)-H(17C) | 109.5 |
| H(17B)-C(17)-H(17C) | 109.5 |

| | |
|---|---|
| C(19)-C(18)-C(13) | 121.79(13) |
| C(19)-C(18)-H(18) | 119.1 |
| C(13)-C(18)-H(18) | 119.1 |
| C(18)-C(19)-C(10) | 121.31(13) |
| C(18)-C(19)-H(19) | 119.3 |
| C(10)-C(19)-H(19) | 119.3 |
| C(5)-N(1)-C(9) | 129.18(11) |
| C(5)-N(1)-C(6) | 121.85(11) |
| C(9)-N(1)-C(6) | 108.86(11) |

Symmetry transformations used to generate equivalent atoms:

Table S4. Anisotropic displacement parameters ($Å^2 x 10^3$)for d19599. The anisotropic displacement factor exponent takes the form: $-2\pi^2[ h^2a^{*2}U^{11} + ... + 2 h k a^* b^* U^{12} ]$

| | $U^{11}$ | $U^{22}$ | $U^{33}$ | $U^{23}$ | $U^{13}$ | $U^{12}$ |
|---|---|---|---|---|---|---|
| C(1) | 40(1) | 28(1) | 60(1) | 7(1) | 19(1) | 4(1) |
| C(2) | 26(1) | 27(1) | 42(1) | 4(1) | 14(1) | 3(1) |
| C(3) | 34(1) | 35(1) | 42(1) | 12(1) | 14(1) | 7(1) |
| C(4) | 36(1) | 41(1) | 28(1) | 2(1) | 11(1) | 4(1) |
| C(5) | 29(1) | 30(1) | 28(1) | -3(1) | 11(1) | 2(1) |
| C(6) | 23(1) | 22(1) | 37(1) | -3(1) | 13(1) | -1(1) |
| C(7) | 31(1) | 28(1) | 35(1) | -8(1) | 12(1) | -3(1) |
| C(8) | 33(1) | 33(1) | 27(1) | 0(1) | 10(1) | -2(1) |
| C(9) | 25(1) | 27(1) | 28(1) | 2(1) | 11(1) | 0(1) |
| C(10) | 30(1) | 25(1) | 26(1) | 2(1) | 12(1) | 0(1) |
| C(11) | 24(1) | 28(1) | 44(1) | 4(1) | 8(1) | 2(1) |
| C(12) | 24(1) | 27(1) | 44(1) | 2(1) | 7(1) | -3(1) |
| C(13) | 27(1) | 26(1) | 27(1) | 3(1) | 11(1) | 0(1) |
| C(14) | 28(1) | 24(1) | 33(1) | 4(1) | 11(1) | 1(1) |
| C(15) | 57(1) | 29(1) | 55(1) | -5(1) | 1(1) | 2(1) |
| C(16) | 70(1) | 35(1) | 62(1) | 10(1) | 40(1) | 1(1) |
| C(17) | 39(1) | 30(1) | 72(1) | 4(1) | 23(1) | 6(1) |
| C(18) | 28(1) | 29(1) | 37(1) | 4(1) | 2(1) | 3(1) |
| C(19) | 29(1) | 30(1) | 33(1) | 1(1) | 2(1) | -4(1) |
| N(1) | 24(1) | 23(1) | 28(1) | -1(1) | 10(1) | 0(1) |
| O(1) | 60(1) | 29(1) | 68(1) | -7(1) | 19(1) | -2(1) |

Table S5. Hydrogen coordinates ( x $10^4$) and isotropic displacement parameters ($Å^2$x $10^3$) for d19599.

| | x | y | z | U(eq) |
|---|---|---|---|---|
| H(1) | 5776 | 1164 | 3980 | 51 |
| H(3) | 6411 | 2338 | 5455 | 44 |
| H(4) | 6669 | 3865 | 6010 | 42 |
| H(5) | 6053 | 4946 | 4490 | 35 |
| H(7) | 4474 | 2672 | 852 | 37 |
| H(8) | 4069 | 4273 | 133 | 37 |
| H(11) | 7144 | 5828 | 3112 | 39 |
| H(12) | 7053 | 7375 | 3143 | 39 |
| H(15A) | 6950 | 8770 | 3334 | 77 |
| H(15B) | 5666 | 8707 | 3983 | 77 |
| H(15C) | 5813 | 9594 | 3246 | 77 |
| H(16A) | 4486 | 8638 | 200 | 77 |
| H(16B) | 6252 | 8617 | 1059 | 77 |
| H(16C) | 5268 | 9513 | 961 | 77 |
| H(17A) | 3227 | 9574 | 1948 | 69 |
| H(17B) | 2855 | 8695 | 2596 | 69 |
| H(17C) | 2461 | 8723 | 1121 | 69 |
| H(18) | 2584 | 7310 | 1045 | 40 |
| H(19) | 2664 | 5762 | 1026 | 39 |

## (B) X-ray crystallographic data of compound (3d).

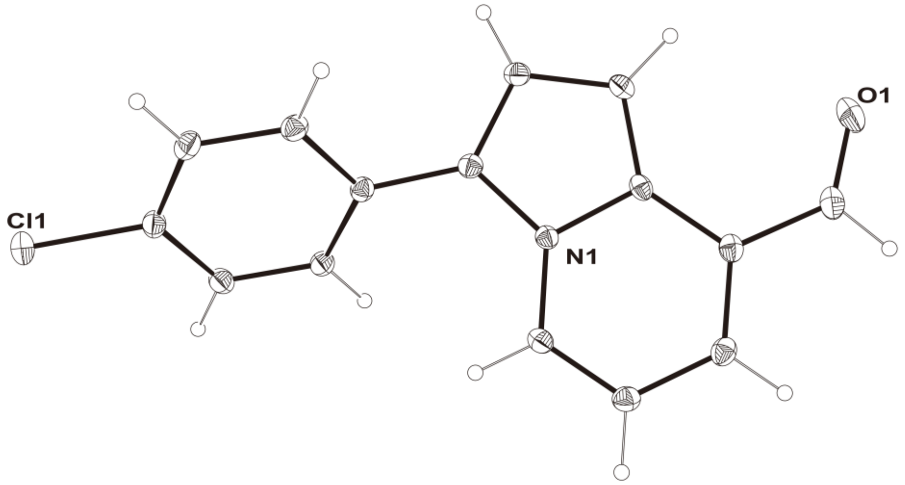


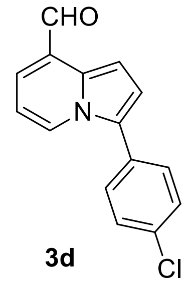


**3d**

CCDC 1894128

Table S6. Crystal data and structure refinement for d19220.

| | | |
|---|---|---|
| Identification code | d19220 | |
| Empirical formula | C15 H10 Cl N O | |
| Formula weight | 255.69 | |
| Temperature | 200(2) K | |
| Wavelength | 0.71073 Å | |
| Crystal system | Monoclinic | |
| Space group | P 21/c | |
| Unit cell dimensions | a = 3.9037(4) Å | $\alpha$= 90°. |

| | | |
|---|---|---|
| | b = 39.676(5) Å | β= 98.326(3)°. |
| | c = 7.6371(9) Å | γ = 90°. |
| Volume | 1170.4(2) $Å^3$ | |
| Z | 4 | |
| Density (calculated) | 1.451 $Mg/m^3$ | |
| Absorption coefficient | 0.311 $mm^{-1}$ | |
| F(000) | 528 | |
| Crystal size | 0.66 x 0.09 x 0.03 $mm^3$ | |
| Theta range for data collection | 2.74 to 25.06°. | |
| Index ranges | -4<=h<=4, -47<=k<=47, -9<=l<=8 | |
| Reflections collected | 15094 | |
| Independent reflections | 2058 [R(int) = 0.0283] | |
| Completeness to theta = 25.06° | 99.0 % | |
| Absorption correction | multi-scan | |
| Max. and min. transmission | 0.9907 and 0.8212 | |
| Refinement method | Full-matrix least-squares on $F^2$ | |
| Data / restraints / parameters | 2058 / 0 / 163 | |
| Goodness-of-fit on $F^2$ | 1.075 | |
| Final R indices [I>2sigma(I)] | R1 = 0.0422, wR2 = 0.1100 | |
| R indices (all data) | R1 = 0.0452, wR2 = 0.1129 | |
| Largest diff. peak and hole | 0.502 and -0.224 e.$Å^{-3}$ | |

Table S7. Atomic coordinates ( x $10^4$) and equivalent isotropic displacement parameters (Å$^2$x $10^3$) for d19220. U(eq) is defined as one third of the trace of the orthogonalized $U^{ij}$ tensor.

| | x | y | z | U(eq) |
|---|---|---|---|---|
| C(1) | 3383(5) | 4488(1) | 3551(2) | 30(1) |
| C(2) | 3168(5) | 4590(1) | 5257(3) | 33(1) |
| C(3) | 4165(5) | 4370(1) | 6641(2) | 30(1) |
| C(4) | 5355(4) | 4047(1) | 6331(2) | 26(1) |
| C(5) | 5586(4) | 3954(1) | 4594(2) | 28(1) |
| C(6) | 4609(5) | 4173(1) | 3199(2) | 31(1) |
| C(7) | 6468(4) | 3827(1) | 7849(2) | 26(1) |
| C(8) | 8546(5) | 3901(1) | 9422(2) | 29(1) |
| C(9) | 8846(5) | 3617(1) | 10520(2) | 29(1) |
| C(10) | 6904(4) | 3365(1) | 9632(2) | 25(1) |
| C(11) | 6050(5) | 3027(1) | 10035(2) | 28(1) |
| C(12) | 7403(5) | 2872(1) | 11730(3) | 38(1) |
| C(13) | 3896(5) | 2843(1) | 8829(2) | 31(1) |
| C(14) | 2488(5) | 2985(1) | 7178(2) | 31(1) |
| C(15) | 3273(4) | 3305(1) | 6786(2) | 28(1) |
| Cl(1) | 2084(2) | 4761(1) | 1793(1) | 47(1) |
| N(1) | 5446(4) | 3494(1) | 7967(2) | 24(1) |
| O(1) | 9268(5) | 3002(1) | 12932(2) | 58(1) |

Table S8. Bond lengths [Å] and angles [°] for d19220.

| | |
|---|---|
| C(1)-C(2) | 1.379(3) |
| C(1)-C(6) | 1.380(3) |
| C(1)-Cl(1) | 1.7432(18) |
| C(2)-C(3) | 1.384(3) |
| C(2)-H(2) | 0.9500 |
| C(3)-C(4) | 1.393(3) |
| C(3)-H(3) | 0.9500 |
| C(4)-C(5) | 1.392(3) |
| C(4)-C(7) | 1.466(2) |
| C(5)-C(6) | 1.385(3) |
| C(5)-H(5) | 0.9500 |
| C(6)-H(6) | 0.9500 |
| C(7)-C(8) | 1.380(3) |
| C(7)-N(1) | 1.386(2) |
| C(8)-C(9) | 1.401(3) |
| C(8)-H(8) | 0.9500 |
| C(9)-C(10) | 1.372(3) |
| C(9)-H(9) | 0.9500 |
| C(10)-N(1) | 1.412(2) |
| C(10)-C(11) | 1.428(3) |
| C(11)-C(13) | 1.363(3) |
| C(11)-C(12) | 1.460(3) |
| C(12)-O(1) | 1.202(3) |
| C(12)-H(12) | 0.9500 |
| C(13)-C(14) | 1.417(3) |
| C(13)-H(13) | 0.9500 |
| C(14)-C(15) | 1.349(3) |
| C(14)-H(14) | 0.9500 |
| C(15)-N(1) | 1.370(2) |
| C(15)-H(15) | 0.9500 |
| | |
| C(2)-C(1)-C(6) | 121.42(17) |
| C(2)-C(1)-Cl(1) | 119.61(14) |
| C(6)-C(1)-Cl(1) | 118.97(15) |

| | |
|---|---|
| C(1)-C(2)-C(3) | 119.10(17) |
| C(1)-C(2)-H(2) | 120.4 |
| C(3)-C(2)-H(2) | 120.4 |
| C(2)-C(3)-C(4) | 121.00(17) |
| C(2)-C(3)-H(3) | 119.5 |
| C(4)-C(3)-H(3) | 119.5 |
| C(5)-C(4)-C(3) | 118.43(16) |
| C(5)-C(4)-C(7) | 122.79(15) |
| C(3)-C(4)-C(7) | 118.71(16) |
| C(6)-C(5)-C(4) | 121.11(16) |
| C(6)-C(5)-H(5) | 119.4 |
| C(4)-C(5)-H(5) | 119.4 |
| C(1)-C(6)-C(5) | 118.93(17) |
| C(1)-C(6)-H(6) | 120.5 |
| C(5)-C(6)-H(6) | 120.5 |
| C(8)-C(7)-N(1) | 106.54(15) |
| C(8)-C(7)-C(4) | 129.13(16) |
| N(1)-C(7)-C(4) | 124.30(15) |
| C(7)-C(8)-C(9) | 109.71(16) |
| C(7)-C(8)-H(8) | 125.1 |
| C(9)-C(8)-H(8) | 125.1 |
| C(10)-C(9)-C(8) | 107.33(16) |
| C(10)-C(9)-H(9) | 126.3 |
| C(8)-C(9)-H(9) | 126.3 |
| C(9)-C(10)-N(1) | 107.61(15) |
| C(9)-C(10)-C(11) | 135.00(16) |
| N(1)-C(10)-C(11) | 117.36(15) |
| C(13)-C(11)-C(10) | 119.73(16) |
| C(13)-C(11)-C(12) | 118.93(17) |
| C(10)-C(11)-C(12) | 121.33(17) |
| O(1)-C(12)-C(11) | 126.81(19) |
| O(1)-C(12)-H(12) | 116.6 |
| C(11)-C(12)-H(12) | 116.6 |
| C(11)-C(13)-C(14) | 120.78(17) |
| C(11)-C(13)-H(13) | 119.6 |
| C(14)-C(13)-H(13) | 119.6 |

| | |
|---|---|
| C(15)-C(14)-C(13) | 119.93(17) |
| C(15)-C(14)-H(14) | 120.0 |
| C(13)-C(14)-H(14) | 120.0 |
| C(14)-C(15)-N(1) | 120.62(16) |
| C(14)-C(15)-H(15) | 119.7 |
| N(1)-C(15)-H(15) | 119.7 |
| C(15)-N(1)-C(7) | 129.56(15) |
| C(15)-N(1)-C(10) | 121.57(15) |
| C(7)-N(1)-C(10) | 108.81(14) |

Symmetry transformations used to generate equivalent atoms:

Table S9. Anisotropic displacement parameters ($Å^2$x $10^3$)for d19220. The anisotropic displacement factor exponent takes the form: $-2\pi^2[ h^2a^{*2}U^{11} + ... + 2 h k a^* b^* U^{12} ]$

| | $U^{11}$ | $U^{22}$ | $U^{33}$ | $U^{23}$ | $U^{13}$ | $U^{12}$ |
|---|---|---|---|---|---|---|
| C(1) | 29(1) | 28(1) | 33(1) | 7(1) | 0(1) | -2(1) |
| C(2) | 35(1) | 23(1) | 40(1) | 1(1) | 5(1) | 3(1) |
| C(3) | 35(1) | 26(1) | 30(1) | -2(1) | 8(1) | 0(1) |
| C(4) | 24(1) | 25(1) | 29(1) | 2(1) | 4(1) | -1(1) |
| C(5) | 30(1) | 24(1) | 29(1) | -1(1) | 5(1) | 3(1) |
| C(6) | 31(1) | 34(1) | 26(1) | 1(1) | 2(1) | -2(1) |
| C(7) | 27(1) | 25(1) | 26(1) | -2(1) | 6(1) | 1(1) |
| C(8) | 33(1) | 28(1) | 26(1) | -4(1) | 4(1) | -3(1) |
| C(9) | 32(1) | 34(1) | 20(1) | -1(1) | 1(1) | 0(1) |
| C(10) | 27(1) | 30(1) | 19(1) | 1(1) | 6(1) | 5(1) |
| C(11) | 31(1) | 29(1) | 26(1) | 2(1) | 8(1) | 5(1) |
| C(12) | 46(1) | 38(1) | 33(1) | 10(1) | 7(1) | 3(1) |
| C(13) | 37(1) | 25(1) | 32(1) | 0(1) | 11(1) | 1(1) |
| C(14) | 33(1) | 29(1) | 30(1) | -5(1) | 5(1) | -2(1) |
| C(15) | 29(1) | 30(1) | 24(1) | -2(1) | 2(1) | 2(1) |
| Cl(1) | 61(1) | 36(1) | 40(1) | 13(1) | -7(1) | 3(1) |
| N(1) | 25(1) | 24(1) | 22(1) | -1(1) | 3(1) | 3(1) |
| O(1) | 71(1) | 65(1) | 33(1) | 15(1) | -9(1) | -8(1) |

Table S10. Hydrogen coordinates ( x $10^4$) and isotropic displacement parameters ($Å^2$x $10^3$) for d19220.

| | x | y | z | U(eq) |
|---|---|---|---|---|
| H(2) | 2346 | 4809 | 5480 | 40 |
| H(3) | 4036 | 4439 | 7820 | 36 |
| H(5) | 6429 | 3736 | 4363 | 33 |
| H(6) | 4779 | 4107 | 2018 | 37 |
| H(8) | 9611 | 4113 | 9716 | 35 |
| H(9) | 10153 | 3601 | 11668 | 35 |
| H(12) | 6741 | 2645 | 11899 | 46 |
| H(13) | 3334 | 2618 | 9099 | 37 |
| H(14) | 992 | 2855 | 6347 | 37 |
| H(15) | 2309 | 3400 | 5682 | 33 |

## (C) X-ray crystallographic data of compound (3l).

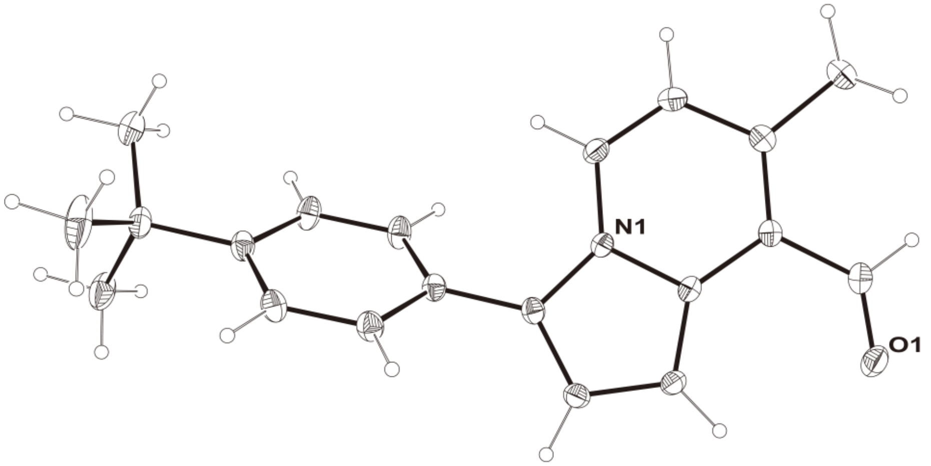


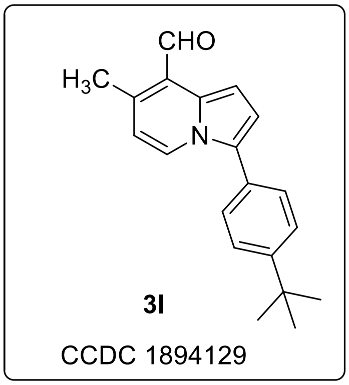


Table S11. Crystal data and structure refinement for d19746.

| | | |
|---|---|---|
| Identification code | d19746 | |
| Empirical formula | C20 H21 N O | |
| Formula weight | 291.38 | |
| Temperature | 200(2) K | |
| Wavelength | 0.71073 Å | |
| Crystal system | Triclinic | |
| Space group | P -1 | |
| Unit cell dimensions | a = 6.7561(3) Å | $\alpha$= 90.518(2)°. |
| | b = 8.5826(5) Å | $\beta$= 90.543(2)°. |
| | c = 14.0541(8) Å | $\gamma$ = 103.168(2)°. |
| Volume | 793.41(7) Å$^3$ | |

| | |
|---|---|
| Z | 2 |
| Density (calculated) | 1.220 $Mg/m^3$ |
| Absorption coefficient | 0.074 $mm^{-1}$ |
| F(000) | 312 |
| Crystal size | 0.75 x 0.72 x 0.23 $mm^3$ |
| Theta range for data collection | 2.44 to 25.06°. |
| Index ranges | $-8 \le h \le 8$, $-10 \le k \le 10$, $-16 \le l \le 16$ |
| Reflections collected | 17180 |
| Independent reflections | 2745 [R(int) = 0.0527] |
| Completeness to theta = 25.06° | 97.7 % |
| Absorption correction | multi-scan |
| Max. and min. transmission | 0.9831 and 0.9463 |
| Refinement method | Full-matrix least-squares on $F^2$ |
| Data / restraints / parameters | 2745 / 0 / 204 |
| Goodness-of-fit on $F^2$ | 1.037 |
| Final R indices [I>2sigma(I)] | R1 = 0.0466, wR2 = 0.1205 |
| R indices (all data) | R1 = 0.0506, wR2 = 0.1247 |
| Extinction coefficient | 0.24(5) |
| Largest diff. peak and hole | 0.201 and -0.180 e.$Å^{-3}$ |

Table S12. Atomic coordinates ( x $10^4$) and equivalent isotropic displacement parameters ($Å^2$x $10^3$) for d19746. U(eq) is defined as one third of the trace of the orthogonalized $U^{ij}$ tensor.

| | x | y | z | U(eq) |
|---|---|---|---|---|
| C(1) | 4951(2) | 12984(2) | 9784(1) | 39(1) |
| C(2) | 5501(2) | 11844(2) | 9114(1) | 30(1) |
| C(3) | 7424(2) | 12123(2) | 8735(1) | 34(1) |
| C(4) | 9089(3) | 13583(2) | 8941(1) | 48(1) |
| C(5) | 7883(2) | 10941(2) | 8110(1) | 39(1) |
| C(6) | 6535(2) | 9567(2) | 7906(1) | 36(1) |
| C(7) | 4024(2) | 10416(2) | 8873(1) | 29(1) |
| C(8) | 2009(2) | 9822(2) | 9072(1) | 33(1) |
| C(9) | 1379(2) | 8333(2) | 8615(1) | 35(1) |
| C(10) | 2990(2) | 7983(2) | 8134(1) | 32(1) |
| C(11) | 3099(2) | 6579(2) | 7546(1) | 33(1) |
| C(12) | 4634(3) | 5754(2) | 7658(1) | 41(1) |
| C(13) | 4686(3) | 4433(2) | 7095(1) | 43(1) |
| C(14) | 3229(2) | 3864(2) | 6399(1) | 37(1) |
| C(15) | 1660(2) | 4664(2) | 6315(1) | 43(1) |
| C(16) | 1604(2) | 5996(2) | 6866(1) | 40(1) |
| C(17) | 3327(3) | 2402(2) | 5782(1) | 42(1) |
| C(18) | 5533(4) | 2309(3) | 5621(2) | 83(1) |
| C(19) | 2342(5) | 2472(3) | 4808(1) | 82(1) |
| C(20) | 2241(4) | 902(2) | 6290(1) | 64(1) |
| N(1) | 4627(2) | 9265(1) | 8285(1) | 29(1) |
| O(1) | 3298(2) | 12855(1) | 10145(1) | 48(1) |

Table S13. Bond lengths [Å] and angles [°] for d19746.

| | |
|---|---|
| C(1)-O(1) | 1.2137(19) |
| C(1)-C(2) | 1.462(2) |
| C(1)-H(1) | 0.9500 |
| C(2)-C(3) | 1.380(2) |
| C(2)-C(7) | 1.429(2) |
| C(3)-C(5) | 1.423(2) |
| C(3)-C(4) | 1.505(2) |
| C(4)-H(4A) | 0.9800 |
| C(4)-H(4B) | 0.9800 |
| C(4)-H(4C) | 0.9800 |
| C(5)-C(6) | 1.342(2) |
| C(5)-H(5) | 0.9500 |
| C(6)-N(1) | 1.3697(18) |
| C(6)-H(6) | 0.9500 |
| C(7)-C(8) | 1.373(2) |
| C(7)-N(1) | 1.4138(17) |
| C(8)-C(9) | 1.400(2) |
| C(8)-H(8) | 0.9500 |
| C(9)-C(10) | 1.375(2) |
| C(9)-H(9) | 0.9500 |
| C(10)-N(1) | 1.3842(18) |
| C(10)-C(11) | 1.4711(19) |
| C(11)-C(16) | 1.388(2) |
| C(11)-C(12) | 1.391(2) |
| C(12)-C(13) | 1.384(2) |
| C(12)-H(12) | 0.9500 |
| C(13)-C(14) | 1.385(2) |
| C(13)-H(13) | 0.9500 |
| C(14)-C(15) | 1.393(2) |
| C(14)-C(17) | 1.533(2) |
| C(15)-C(16) | 1.383(2) |
| C(15)-H(15) | 0.9500 |
| C(16)-H(16) | 0.9500 |
| C(17)-C(20) | 1.517(2) |

| | |
|---|---|
| C(17)-C(19) | 1.524(3) |
| C(17)-C(18) | 1.529(3) |
| C(18)-H(18A) | 0.9800 |
| C(18)-H(18B) | 0.9800 |
| C(18)-H(18C) | 0.9800 |
| C(19)-H(19A) | 0.9800 |
| C(19)-H(19B) | 0.9800 |
| C(19)-H(19C) | 0.9800 |
| C(20)-H(20A) | 0.9800 |
| C(20)-H(20B) | 0.9800 |
| C(20)-H(20C) | 0.9800 |
| | |
| O(1)-C(1)-C(2) | 125.55(14) |
| O(1)-C(1)-H(1) | 117.2 |
| C(2)-C(1)-H(1) | 117.2 |
| C(3)-C(2)-C(7) | 120.42(13) |
| C(3)-C(2)-C(1) | 120.71(13) |
| C(7)-C(2)-C(1) | 118.86(13) |
| C(2)-C(3)-C(5) | 117.93(13) |
| C(2)-C(3)-C(4) | 124.71(14) |
| C(5)-C(3)-C(4) | 117.36(14) |
| C(3)-C(4)-H(4A) | 109.5 |
| C(3)-C(4)-H(4B) | 109.5 |
| H(4A)-C(4)-H(4B) | 109.5 |
| C(3)-C(4)-H(4C) | 109.5 |
| H(4A)-C(4)-H(4C) | 109.5 |
| H(4B)-C(4)-H(4C) | 109.5 |
| C(6)-C(5)-C(3) | 122.28(14) |
| C(6)-C(5)-H(5) | 118.9 |
| C(3)-C(5)-H(5) | 118.9 |
| C(5)-C(6)-N(1) | 120.45(13) |
| C(5)-C(6)-H(6) | 119.8 |
| N(1)-C(6)-H(6) | 119.8 |
| C(8)-C(7)-N(1) | 106.59(12) |
| C(8)-C(7)-C(2) | 135.20(13) |
| N(1)-C(7)-C(2) | 118.21(12) |

| | |
|---|---|
| C(7)-C(8)-C(9) | 108.16(12) |
| C(7)-C(8)-H(8) | 125.9 |
| C(9)-C(8)-H(8) | 125.9 |
| C(10)-C(9)-C(8) | 109.31(13) |
| C(10)-C(9)-H(9) | 125.3 |
| C(8)-C(9)-H(9) | 125.3 |
| C(9)-C(10)-N(1) | 106.63(12) |
| C(9)-C(10)-C(11) | 130.14(13) |
| N(1)-C(10)-C(11) | 123.22(12) |
| C(16)-C(11)-C(12) | 117.23(13) |
| C(16)-C(11)-C(10) | 120.23(13) |
| C(12)-C(11)-C(10) | 122.51(13) |
| C(13)-C(12)-C(11) | 121.01(14) |
| C(13)-C(12)-H(12) | 119.5 |
| C(11)-C(12)-H(12) | 119.5 |
| C(12)-C(13)-C(14) | 122.21(14) |
| C(12)-C(13)-H(13) | 118.9 |
| C(14)-C(13)-H(13) | 118.9 |
| C(13)-C(14)-C(15) | 116.33(13) |
| C(13)-C(14)-C(17) | 121.18(14) |
| C(15)-C(14)-C(17) | 122.46(14) |
| C(16)-C(15)-C(14) | 121.92(14) |
| C(16)-C(15)-H(15) | 119.0 |
| C(14)-C(15)-H(15) | 119.0 |
| C(15)-C(16)-C(11) | 121.23(14) |
| C(15)-C(16)-H(16) | 119.4 |
| C(11)-C(16)-H(16) | 119.4 |
| C(20)-C(17)-C(19) | 109.54(17) |
| C(20)-C(17)-C(18) | 108.64(17) |
| C(19)-C(17)-C(18) | 107.52(18) |
| C(20)-C(17)-C(14) | 108.78(13) |
| C(19)-C(17)-C(14) | 111.55(14) |
| C(18)-C(17)-C(14) | 110.77(14) |
| C(17)-C(18)-H(18A) | 109.5 |
| C(17)-C(18)-H(18B) | 109.5 |
| H(18A)-C(18)-H(18B) | 109.5 |

| | |
|---|---|
| C(17)-C(18)-H(18C) | 109.5 |
| H(18A)-C(18)-H(18C) | 109.5 |
| H(18B)-C(18)-H(18C) | 109.5 |
| C(17)-C(19)-H(19A) | 109.5 |
| C(17)-C(19)-H(19B) | 109.5 |
| H(19A)-C(19)-H(19B) | 109.5 |
| C(17)-C(19)-H(19C) | 109.5 |
| H(19A)-C(19)-H(19C) | 109.5 |
| H(19B)-C(19)-H(19C) | 109.5 |
| C(17)-C(20)-H(20A) | 109.5 |
| C(17)-C(20)-H(20B) | 109.5 |
| H(20A)-C(20)-H(20B) | 109.5 |
| C(17)-C(20)-H(20C) | 109.5 |
| H(20A)-C(20)-H(20C) | 109.5 |
| H(20B)-C(20)-H(20C) | 109.5 |
| C(6)-N(1)-C(10) | 129.94(12) |
| C(6)-N(1)-C(7) | 120.56(12) |
| C(10)-N(1)-C(7) | 109.32(11) |

Symmetry transformations used to generate equivalent atoms:

Table S14. Anisotropic displacement parameters ($Å^2 x 10^3$)for d19746. The anisotropic displacement factor exponent takes the form: $-2\pi^2[ h^2a^{*2}U^{11} + ... + 2 h k a^* b^* U^{12} ]$

| | $U^{11}$ | $U^{22}$ | $U^{33}$ | $U^{23}$ | $U^{13}$ | $U^{12}$ |
|---|---|---|---|---|---|---|
| C(1) | 47(1) | 32(1) | 40(1) | -5(1) | -6(1) | 12(1) |
| C(2) | 36(1) | 28(1) | 28(1) | 2(1) | -4(1) | 9(1) |
| C(3) | 36(1) | 33(1) | 33(1) | 5(1) | -4(1) | 7(1) |
| C(4) | 44(1) | 42(1) | 53(1) | 2(1) | -2(1) | -1(1) |
| C(5) | 31(1) | 46(1) | 39(1) | 2(1) | 6(1) | 8(1) |
| C(6) | 34(1) | 41(1) | 36(1) | -4(1) | 5(1) | 13(1) |
| C(7) | 36(1) | 29(1) | 24(1) | 1(1) | 0(1) | 13(1) |
| C(8) | 33(1) | 35(1) | 34(1) | 0(1) | 6(1) | 11(1) |
| C(9) | 30(1) | 34(1) | 40(1) | 2(1) | 2(1) | 5(1) |
| C(10) | 35(1) | 27(1) | 34(1) | 0(1) | -3(1) | 7(1) |
| C(11) | 40(1) | 29(1) | 31(1) | 0(1) | -1(1) | 8(1) |
| C(12) | 50(1) | 40(1) | 37(1) | -9(1) | -15(1) | 18(1) |
| C(13) | 59(1) | 38(1) | 38(1) | -6(1) | -9(1) | 24(1) |
| C(14) | 52(1) | 29(1) | 28(1) | 0(1) | 0(1) | 6(1) |
| C(15) | 46(1) | 41(1) | 39(1) | -7(1) | -10(1) | 5(1) |
| C(16) | 40(1) | 37(1) | 43(1) | -3(1) | -6(1) | 11(1) |
| C(17) | 61(1) | 30(1) | 32(1) | -4(1) | 4(1) | 6(1) |
| C(18) | 76(2) | 69(1) | 97(2) | -45(1) | 26(1) | 4(1) |
| C(19) | 151(2) | 60(1) | 38(1) | -16(1) | -17(1) | 30(1) |
| C(20) | 104(2) | 33(1) | 51(1) | -4(1) | 24(1) | 6(1) |
| N(1) | 31(1) | 29(1) | 29(1) | -1(1) | 0(1) | 11(1) |
| O(1) | 51(1) | 49(1) | 46(1) | -13(1) | 2(1) | 19(1) |

Table S15. Hydrogen coordinates ( x $10^4$) and isotropic displacement parameters ($Å^2$x $10^3$) for d19746.

| | x | y | z | U(eq) |
|---|---|---|---|---|
| H(1) | 5975 | 13905 | 9950 | 47 |
| H(4A) | 10040 | 13763 | 8410 | 72 |
| H(4B) | 9815 | 13420 | 9525 | 72 |
| H(4C) | 8496 | 14516 | 9022 | 72 |
| H(5) | 9187 | 11132 | 7829 | 46 |
| H(6) | 6908 | 8798 | 7497 | 44 |
| H(8) | 1186 | 10332 | 9455 | 40 |
| H(9) | 47 | 7668 | 8634 | 42 |
| H(12) | 5663 | 6104 | 8129 | 49 |
| H(13) | 5757 | 3898 | 7190 | 52 |
| H(15) | 597 | 4284 | 5865 | 51 |
| H(16) | 521 | 6520 | 6779 | 48 |
| H(18A) | 5564 | 1396 | 5202 | 124 |
| H(18B) | 6176 | 2172 | 6233 | 124 |
| H(18C) | 6272 | 3298 | 5325 | 124 |
| H(19A) | 2981 | 3478 | 4499 | 123 |
| H(19B) | 887 | 2414 | 4884 | 123 |
| H(19C) | 2527 | 1568 | 4414 | 123 |
| H(20A) | 795 | 897 | 6343 | 96 |
| H(20B) | 2834 | 883 | 6928 | 96 |
| H(20C) | 2394 | -44 | 5928 | 96 |

## (D) X-ray crystallographic data of compound (5b).

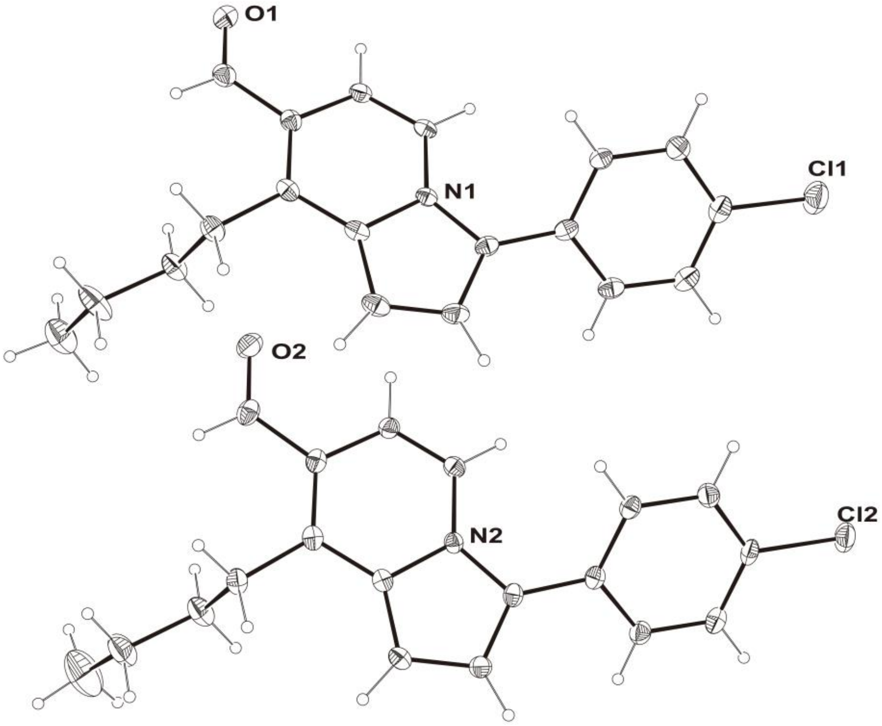


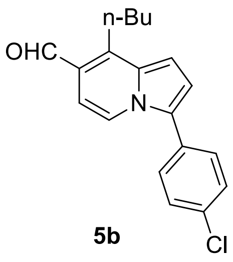


**5b**

CCDC 1894126

Table S16. Crystal data and structure refinement for d19628.

| | |
|---|---|
| Identification code | d19628 |
| Empirical formula | C19 H18 Cl N O |
| Formula weight | 311.79 |
| Temperature | 200(2) K |
| Wavelength | 0.71073 Å |
| Crystal system | Triclinic |
| Space group | P -1 |

| | | |
|---|---|---|
| Unit cell dimensions | a = 8.3841(9) Å | α= 90.770(3)°. |
| | b = 13.7451(15) Å | β= 97.215(3)°. |
| | c = 14.1044(15) Å | γ = 94.980(3)°. |
| Volume | 1606.0(3) Å$^3$ | |
| Z | 4 | |
| Density (calculated) | 1.290 Mg/m$^3$ | |
| Absorption coefficient | 0.239 mm$^{-1}$ | |
| F(000) | 656 | |
| Crystal size | 0.32 x 0.05 x 0.01 mm$^3$ | |
| Theta range for data collection | 2.46 to 25.07°. | |
| Index ranges | -9<=h<=9, -16<=k<=16, -16<=l<=16 | |
| Reflections collected | 39950 | |
| Independent reflections | 5665 [R(int) = 0.1459] | |
| Completeness to theta = 25.07° | 99.5 % | |
| Absorption correction | multi-scan | |
| Max. and min. transmission | 0.9976 and 0.9274 | |
| Refinement method | Full-matrix least-squares on F$^2$ | |
| Data / restraints / parameters | 5665 / 0 / 400 | |
| Goodness-of-fit on F$^2$ | 1.019 | |
| Final R indices [I>2sigma(I)] | R1 = 0.0641, wR2 = 0.1312 | |
| R indices (all data) | R1 = 0.1365, wR2 = 0.1607 | |
| Extinction coefficient | 0.0125(16) | |
| Largest diff. peak and hole | 0.652 and -0.251 e.Å$^{-3}$ | |

Table S17. Atomic coordinates ( x $10^4$) and equivalent isotropic displacement parameters ($Å^2$x $10^3$) for d19628. U(eq) is defined as one third of the trace of the orthogonalized $U^{ij}$ tensor.

| | x | y | z | U(eq) |
|---|---|---|---|---|
| C(1) | 8502(4) | 8849(3) | 7803(3) | 46(1) |
| C(2) | 8401(4) | 7860(3) | 7561(3) | 43(1) |
| C(3) | 8250(4) | 7577(3) | 6613(3) | 36(1) |
| C(4) | 8183(4) | 8265(3) | 5889(3) | 35(1) |
| C(5) | 8252(4) | 9248(3) | 6158(3) | 42(1) |
| C(6) | 8420(4) | 9547(3) | 7100(3) | 47(1) |
| C(7) | 8174(4) | 8011(3) | 4884(3) | 35(1) |
| C(8) | 8991(4) | 8510(3) | 4217(3) | 45(1) |
| C(9) | 8743(4) | 8001(3) | 3357(3) | 44(1) |
| C(10) | 7733(4) | 7162(3) | 3458(3) | 35(1) |
| C(11) | 7097(4) | 6373(3) | 2842(2) | 35(1) |
| C(12) | 7571(4) | 6366(3) | 1842(2) | 42(1) |
| C(13) | 6326(4) | 6771(3) | 1113(3) | 49(1) |
| C(14) | 6759(5) | 6712(4) | 87(3) | 76(2) |
| C(15) | 5580(6) | 7088(4) | -630(3) | 83(2) |
| C(16) | 6029(4) | 5674(3) | 3167(2) | 35(1) |
| C(17) | 5307(5) | 4828(3) | 2585(3) | 43(1) |
| C(18) | 5600(4) | 5766(3) | 4106(3) | 36(1) |
| C(19) | 6262(4) | 6495(3) | 4711(3) | 33(1) |
| C(20) | 8377(4) | 3422(3) | 9383(2) | 39(1) |
| C(21) | 8246(4) | 4075(3) | 8655(3) | 42(1) |
| C(22) | 7831(4) | 3744(3) | 7715(3) | 36(1) |
| C(23) | 7532(4) | 2745(3) | 7498(2) | 33(1) |
| C(24) | 7652(4) | 2103(3) | 8256(3) | 41(1) |
| C(25) | 8071(4) | 2432(3) | 9191(3) | 45(1) |
| C(26) | 7033(4) | 2351(3) | 6524(2) | 33(1) |
| C(27) | 5957(4) | 1544(3) | 6242(3) | 37(1) |
| C(28) | 5798(4) | 1434(3) | 5255(3) | 38(1) |
| C(29) | 6768(4) | 2175(3) | 4907(2) | 32(1) |
| C(30) | 7042(4) | 2448(3) | 3978(2) | 34(1) |
| C(31) | 6120(4) | 1844(3) | 3153(3) | 41(1) |

| | | | | |
|---|---|---|---|---|
| C(32) | 7011(5) | 1028(3) | 2799(3) | 58(1) |
| C(33) | 6054(6) | 490(4) | 1923(4) | 76(2) |
| C(34) | 6807(8) | -247(5) | 1472(5) | 128(3) |
| C(35) | 8133(4) | 3238(3) | 3871(2) | 35(1) |
| C(36) | 8475(5) | 3588(3) | 2944(3) | 42(1) |
| C(37) | 8970(4) | 3748(3) | 4709(2) | 35(1) |
| C(38) | 8687(4) | 3501(3) | 5592(3) | 34(1) |
| Cl(1) | 8719(2) | 9226(1) | 8998(1) | 72(1) |
| Cl(2) | 8896(1) | 3834(1) | 10558(1) | 57(1) |
| N(1) | 7378(3) | 7190(2) | 4411(2) | 30(1) |
| N(2) | 7542(3) | 2736(2) | 5706(2) | 30(1) |
| O(1) | 4350(3) | 4203(2) | 2841(2) | 57(1) |
| O(2) | 9409(3) | 4293(2) | 2820(2) | 53(1) |

Table S18. Bond lengths [Å] and angles [°] for d19628.

| | |
|---|---|
| C(1)-C(2) | 1.389(5) |
| C(1)-C(6) | 1.389(6) |
| C(1)-Cl(1) | 1.739(4) |
| C(2)-C(3) | 1.375(5) |
| C(2)-H(2) | 0.9500 |
| C(3)-C(4) | 1.400(5) |
| C(3)-H(3) | 0.9500 |
| C(4)-C(5) | 1.393(5) |
| C(4)-C(7) | 1.454(5) |
| C(5)-C(6) | 1.373(5) |
| C(5)-H(5) | 0.9500 |
| C(6)-H(6) | 0.9500 |
| C(7)-N(1) | 1.378(4) |
| C(7)-C(8) | 1.387(5) |
| C(8)-C(9) | 1.376(5) |
| C(8)-H(8) | 0.9500 |
| C(9)-C(10) | 1.390(5) |
| C(9)-H(9) | 0.9500 |
| C(10)-C(11) | 1.405(5) |
| C(10)-N(1) | 1.412(4) |
| C(11)-C(16) | 1.379(5) |
| C(11)-C(12) | 1.513(5) |
| C(12)-C(13) | 1.518(5) |
| C(12)-H(12A) | 0.9900 |
| C(12)-H(12B) | 0.9900 |
| C(13)-C(14) | 1.538(5) |
| C(13)-H(13A) | 0.9900 |
| C(13)-H(13B) | 0.9900 |
| C(14)-C(15) | 1.456(6) |
| C(14)-H(14A) | 0.9900 |
| C(14)-H(14B) | 0.9900 |
| C(15)-H(15A) | 0.9800 |
| C(15)-H(15B) | 0.9800 |
| C(15)-H(15C) | 0.9800 |

| | |
|---|---|
| C(16)-C(18) | 1.423(5) |
| C(16)-C(17) | 1.456(5) |
| C(17)-O(1) | 1.214(4) |
| C(17)-H(17) | 0.9500 |
| C(18)-C(19) | 1.342(5) |
| C(18)-H(18) | 0.9500 |
| C(19)-N(1) | 1.386(4) |
| C(19)-H(19) | 0.9500 |
| C(20)-C(21) | 1.373(5) |
| C(20)-C(25) | 1.379(5) |
| C(20)-Cl(2) | 1.734(4) |
| C(21)-C(22) | 1.387(5) |
| C(21)-H(21) | 0.9500 |
| C(22)-C(23) | 1.397(5) |
| C(22)-H(22) | 0.9500 |
| C(23)-C(24) | 1.395(5) |
| C(23)-C(26) | 1.467(5) |
| C(24)-C(25) | 1.381(5) |
| C(24)-H(24) | 0.9500 |
| C(25)-H(25) | 0.9500 |
| C(26)-N(2) | 1.375(4) |
| C(26)-C(27) | 1.388(5) |
| C(27)-C(28) | 1.387(5) |
| C(27)-H(27) | 0.9500 |
| C(28)-C(29) | 1.382(5) |
| C(28)-H(28) | 0.9500 |
| C(29)-C(30) | 1.409(5) |
| C(29)-N(2) | 1.412(4) |
| C(30)-C(35) | 1.381(5) |
| C(30)-C(31) | 1.508(5) |
| C(31)-C(32) | 1.513(5) |
| C(31)-H(31A) | 0.9900 |
| C(31)-H(31B) | 0.9900 |
| C(32)-C(33) | 1.530(6) |
| C(32)-H(32A) | 0.9900 |
| C(32)-H(32B) | 0.9900 |

| | |
|---|---|
| C(33)-C(34) | 1.423(7) |
| C(33)-H(33A) | 0.9900 |
| C(33)-H(33B) | 0.9900 |
| C(34)-H(34A) | 0.9800 |
| C(34)-H(34B) | 0.9800 |
| C(34)-H(34C) | 0.9800 |
| C(35)-C(37) | 1.437(5) |
| C(35)-C(36) | 1.453(5) |
| C(36)-O(2) | 1.221(4) |
| C(36)-H(36) | 0.9500 |
| C(37)-C(38) | 1.339(5) |
| C(37)-H(37) | 0.9500 |
| C(38)-N(2) | 1.386(4) |
| C(38)-H(38) | 0.9500 |

| | |
|---|---|
| C(2)-C(1)-C(6) | 120.7(4) |
| C(2)-C(1)-Cl(1) | 120.1(3) |
| C(6)-C(1)-Cl(1) | 119.1(3) |
| C(3)-C(2)-C(1) | 119.3(4) |
| C(3)-C(2)-H(2) | 120.4 |
| C(1)-C(2)-H(2) | 120.4 |
| C(2)-C(3)-C(4) | 121.3(4) |
| C(2)-C(3)-H(3) | 119.4 |
| C(4)-C(3)-H(3) | 119.4 |
| C(5)-C(4)-C(3) | 117.9(4) |
| C(5)-C(4)-C(7) | 118.6(3) |
| C(3)-C(4)-C(7) | 123.4(3) |
| C(6)-C(5)-C(4) | 121.8(4) |
| C(6)-C(5)-H(5) | 119.1 |
| C(4)-C(5)-H(5) | 119.1 |
| C(5)-C(6)-C(1) | 119.1(4) |
| C(5)-C(6)-H(6) | 120.5 |
| C(1)-C(6)-H(6) | 120.5 |
| N(1)-C(7)-C(8) | 106.0(3) |
| N(1)-C(7)-C(4) | 126.1(3) |
| C(8)-C(7)-C(4) | 127.9(3) |

| | |
|---|---|
| C(9)-C(8)-C(7) | 110.0(3) |
| C(9)-C(8)-H(8) | 125.0 |
| C(7)-C(8)-H(8) | 125.0 |
| C(8)-C(9)-C(10) | 108.1(3) |
| C(8)-C(9)-H(9) | 125.9 |
| C(10)-C(9)-H(9) | 125.9 |
| C(9)-C(10)-C(11) | 133.8(4) |
| C(9)-C(10)-N(1) | 106.0(3) |
| C(11)-C(10)-N(1) | 120.2(3) |
| C(16)-C(11)-C(10) | 118.4(3) |
| C(16)-C(11)-C(12) | 123.8(3) |
| C(10)-C(11)-C(12) | 117.7(3) |
| C(11)-C(12)-C(13) | 112.7(3) |
| C(11)-C(12)-H(12A) | 109.1 |
| C(13)-C(12)-H(12A) | 109.1 |
| C(11)-C(12)-H(12B) | 109.1 |
| C(13)-C(12)-H(12B) | 109.1 |
| H(12A)-C(12)-H(12B) | 107.8 |
| C(12)-C(13)-C(14) | 112.8(3) |
| C(12)-C(13)-H(13A) | 109.0 |
| C(14)-C(13)-H(13A) | 109.0 |
| C(12)-C(13)-H(13B) | 109.0 |
| C(14)-C(13)-H(13B) | 109.0 |
| H(13A)-C(13)-H(13B) | 107.8 |
| C(15)-C(14)-C(13) | 114.1(4) |
| C(15)-C(14)-H(14A) | 108.7 |
| C(13)-C(14)-H(14A) | 108.7 |
| C(15)-C(14)-H(14B) | 108.7 |
| C(13)-C(14)-H(14B) | 108.7 |
| H(14A)-C(14)-H(14B) | 107.6 |
| C(14)-C(15)-H(15A) | 109.5 |
| C(14)-C(15)-H(15B) | 109.5 |
| H(15A)-C(15)-H(15B) | 109.5 |
| C(14)-C(15)-H(15C) | 109.5 |
| H(15A)-C(15)-H(15C) | 109.5 |
| H(15B)-C(15)-H(15C) | 109.5 |

| | |
|---|---|
| C(11)-C(16)-C(18) | 119.7(3) |
| C(11)-C(16)-C(17) | 122.6(3) |
| C(18)-C(16)-C(17) | 117.7(3) |
| O(1)-C(17)-C(16) | 124.5(4) |
| O(1)-C(17)-H(17) | 117.7 |
| C(16)-C(17)-H(17) | 117.7 |
| C(19)-C(18)-C(16) | 121.9(3) |
| C(19)-C(18)-H(18) | 119.1 |
| C(16)-C(18)-H(18) | 119.1 |
| C(18)-C(19)-N(1) | 119.4(3) |
| C(18)-C(19)-H(19) | 120.3 |
| N(1)-C(19)-H(19) | 120.3 |
| C(21)-C(20)-C(25) | 120.4(3) |
| C(21)-C(20)-Cl(2) | 120.4(3) |
| C(25)-C(20)-Cl(2) | 119.2(3) |
| C(20)-C(21)-C(22) | 120.3(4) |
| C(20)-C(21)-H(21) | 119.8 |
| C(22)-C(21)-H(21) | 119.8 |
| C(21)-C(22)-C(23) | 120.5(3) |
| C(21)-C(22)-H(22) | 119.7 |
| C(23)-C(22)-H(22) | 119.7 |
| C(24)-C(23)-C(22) | 117.7(3) |
| C(24)-C(23)-C(26) | 119.1(3) |
| C(22)-C(23)-C(26) | 123.1(3) |
| C(25)-C(24)-C(23) | 121.7(4) |
| C(25)-C(24)-H(24) | 119.1 |
| C(23)-C(24)-H(24) | 119.1 |
| C(20)-C(25)-C(24) | 119.3(4) |
| C(20)-C(25)-H(25) | 120.3 |
| C(24)-C(25)-H(25) | 120.3 |
| N(2)-C(26)-C(27) | 106.9(3) |
| N(2)-C(26)-C(23) | 125.6(3) |
| C(27)-C(26)-C(23) | 127.6(3) |
| C(28)-C(27)-C(26) | 109.1(3) |
| C(28)-C(27)-H(27) | 125.4 |
| C(26)-C(27)-H(27) | 125.4 |

| | |
|---|---|
| C(29)-C(28)-C(27) | 108.1(3) |
| C(29)-C(28)-H(28) | 126.0 |
| C(27)-C(28)-H(28) | 126.0 |
| C(28)-C(29)-C(30) | 133.3(3) |
| C(28)-C(29)-N(2) | 106.7(3) |
| C(30)-C(29)-N(2) | 120.0(3) |
| C(35)-C(30)-C(29) | 118.8(3) |
| C(35)-C(30)-C(31) | 123.9(3) |
| C(29)-C(30)-C(31) | 117.3(3) |
| C(30)-C(31)-C(32) | 114.6(3) |
| C(30)-C(31)-H(31A) | 108.6 |
| C(32)-C(31)-H(31A) | 108.6 |
| C(30)-C(31)-H(31B) | 108.6 |
| C(32)-C(31)-H(31B) | 108.6 |
| H(31A)-C(31)-H(31B) | 107.6 |
| C(31)-C(32)-C(33) | 111.8(4) |
| C(31)-C(32)-H(32A) | 109.3 |
| C(33)-C(32)-H(32A) | 109.3 |
| C(31)-C(32)-H(32B) | 109.3 |
| C(33)-C(32)-H(32B) | 109.3 |
| H(32A)-C(32)-H(32B) | 107.9 |
| C(34)-C(33)-C(32) | 117.6(5) |
| C(34)-C(33)-H(33A) | 107.9 |
| C(32)-C(33)-H(33A) | 107.9 |
| C(34)-C(33)-H(33B) | 107.9 |
| C(32)-C(33)-H(33B) | 107.9 |
| H(33A)-C(33)-H(33B) | 107.2 |
| C(33)-C(34)-H(34A) | 109.5 |
| C(33)-C(34)-H(34B) | 109.5 |
| H(34A)-C(34)-H(34B) | 109.5 |
| C(33)-C(34)-H(34C) | 109.5 |
| H(34A)-C(34)-H(34C) | 109.5 |
| H(34B)-C(34)-H(34C) | 109.5 |
| C(30)-C(35)-C(37) | 119.1(3) |
| C(30)-C(35)-C(36) | 122.9(3) |
| C(37)-C(35)-C(36) | 117.9(3) |

| | |
|---|---|
| O(2)-C(36)-C(35) | 124.9(4) |
| O(2)-C(36)-H(36) | 117.6 |
| C(35)-C(36)-H(36) | 117.6 |
| C(38)-C(37)-C(35) | 122.1(3) |
| C(38)-C(37)-H(37) | 119.0 |
| C(35)-C(37)-H(37) | 119.0 |
| C(37)-C(38)-N(2) | 119.3(3) |
| C(37)-C(38)-H(38) | 120.3 |
| N(2)-C(38)-H(38) | 120.3 |
| C(7)-N(1)-C(19) | 129.9(3) |
| C(7)-N(1)-C(10) | 109.8(3) |
| C(19)-N(1)-C(10) | 120.0(3) |
| C(26)-N(2)-C(38) | 130.3(3) |
| C(26)-N(2)-C(29) | 109.1(3) |
| C(38)-N(2)-C(29) | 120.4(3) |

Symmetry transformations used to generate equivalent atoms:

Table S19. Anisotropic displacement parameters ($Å^2$x $10^3$)for d19628. The anisotropic displacement factor exponent takes the form: $-2\pi^2[ h^2a^{*2}U^{11} + ... + 2 h k a^* b^* U^{12} ]$

| | $U^{11}$ | $U^{22}$ | $U^{33}$ | $U^{23}$ | $U^{13}$ | $U^{12}$ |
|---|---|---|---|---|---|---|
| C(1) | 39(2) | 51(3) | 49(3) | -12(2) | 8(2) | -2(2) |
| C(2) | 37(2) | 46(3) | 46(3) | 2(2) | 5(2) | 1(2) |
| C(3) | 32(2) | 32(2) | 43(2) | 0(2) | 6(2) | 1(2) |
| C(4) | 24(2) | 33(2) | 48(2) | 0(2) | 7(2) | 0(2) |
| C(5) | 34(2) | 37(2) | 55(3) | 6(2) | 7(2) | -1(2) |
| C(6) | 40(2) | 40(2) | 60(3) | -8(2) | 10(2) | -1(2) |
| C(7) | 29(2) | 31(2) | 45(2) | 2(2) | 4(2) | 0(2) |
| C(8) | 38(2) | 43(2) | 54(3) | 10(2) | 12(2) | -7(2) |
| C(9) | 32(2) | 54(3) | 48(3) | 16(2) | 12(2) | 1(2) |
| C(10) | 28(2) | 40(2) | 39(2) | 12(2) | 7(2) | 7(2) |
| C(11) | 30(2) | 42(2) | 33(2) | 8(2) | 6(2) | 12(2) |
| C(12) | 34(2) | 57(3) | 37(2) | 5(2) | 8(2) | 7(2) |
| C(13) | 39(2) | 73(3) | 37(2) | 10(2) | 6(2) | 1(2) |
| C(14) | 49(3) | 136(5) | 47(3) | 33(3) | 18(2) | 13(3) |
| C(15) | 67(3) | 126(5) | 57(3) | 16(3) | 6(3) | 5(3) |
| C(16) | 36(2) | 38(2) | 33(2) | 4(2) | 2(2) | 10(2) |
| C(17) | 47(2) | 43(3) | 39(2) | 6(2) | 3(2) | 11(2) |
| C(18) | 36(2) | 36(2) | 36(2) | 9(2) | 4(2) | -1(2) |
| C(19) | 30(2) | 37(2) | 34(2) | 11(2) | 9(2) | 4(2) |
| C(20) | 36(2) | 59(3) | 25(2) | -1(2) | 9(2) | 2(2) |
| C(21) | 40(2) | 45(2) | 39(2) | -6(2) | 8(2) | -1(2) |
| C(22) | 31(2) | 44(2) | 33(2) | 3(2) | 8(2) | 4(2) |
| C(23) | 30(2) | 41(2) | 30(2) | 2(2) | 5(2) | 7(2) |
| C(24) | 49(2) | 39(2) | 37(2) | 2(2) | 11(2) | 8(2) |
| C(25) | 51(2) | 54(3) | 32(2) | 6(2) | 10(2) | 11(2) |
| C(26) | 32(2) | 39(2) | 30(2) | -1(2) | 7(2) | 10(2) |
| C(27) | 38(2) | 35(2) | 38(2) | 1(2) | 7(2) | 2(2) |
| C(28) | 35(2) | 36(2) | 40(2) | -5(2) | 0(2) | -1(2) |
| C(29) | 29(2) | 33(2) | 32(2) | -3(2) | 1(2) | 7(2) |
| C(30) | 31(2) | 39(2) | 33(2) | -3(2) | 3(2) | 11(2) |
| C(31) | 40(2) | 50(3) | 32(2) | -4(2) | 2(2) | 4(2) |

| | | | | | | |
|---|---|---|---|---|---|---|
| C(32) | 50(3) | 61(3) | 62(3) | -22(2) | 6(2) | 1(2) |
| C(33) | 81(3) | 71(4) | 71(3) | -33(3) | 8(3) | -6(3) |
| C(34) | 110(5) | 138(6) | 135(6) | -70(5) | 4(4) | 26(4) |
| C(35) | 37(2) | 37(2) | 32(2) | 1(2) | 7(2) | 14(2) |
| C(36) | 48(2) | 46(3) | 35(2) | 8(2) | 6(2) | 18(2) |
| C(37) | 37(2) | 33(2) | 35(2) | 1(2) | 8(2) | 3(2) |
| C(38) | 31(2) | 35(2) | 36(2) | -1(2) | 0(2) | 2(2) |
| Cl(1) | 79(1) | 79(1) | 56(1) | -22(1) | 13(1) | -8(1) |
| Cl(2) | 58(1) | 80(1) | 31(1) | -8(1) | 7(1) | -7(1) |
| N(1) | 27(2) | 32(2) | 31(2) | 7(1) | 7(1) | 2(1) |
| N(2) | 28(2) | 34(2) | 28(2) | -2(1) | 3(1) | 3(1) |
| O(1) | 73(2) | 42(2) | 53(2) | -3(1) | 11(2) | -5(2) |
| O(2) | 66(2) | 49(2) | 46(2) | 11(1) | 16(1) | 8(2) |

Table S20. Hydrogen coordinates ( x $10^4$) and isotropic displacement parameters ($Å^2$x $10^3$) for d19628.

| | x | y | z | U(eq) |
|---|---|---|---|---|
| H(2) | 8436 | 7386 | 8044 | 52 |
| H(3) | 8189 | 6902 | 6446 | 43 |
| H(5) | 8181 | 9724 | 5676 | 51 |
| H(6) | 8479 | 10221 | 7269 | 56 |
| H(8) | 9629 | 9115 | 4336 | 54 |
| H(9) | 9185 | 8190 | 2792 | 53 |
| H(12A) | 7717 | 5687 | 1651 | 51 |
| H(12B) | 8619 | 6758 | 1844 | 51 |
| H(13A) | 6226 | 7462 | 1285 | 59 |
| H(13B) | 5265 | 6402 | 1138 | 59 |
| H(14A) | 7819 | 7083 | 65 | 91 |
| H(14B) | 6871 | 6021 | -79 | 91 |
| H(15A) | 5910 | 7000 | -1266 | 125 |
| H(15B) | 5516 | 7785 | -500 | 125 |
| H(15C) | 4520 | 6734 | -607 | 125 |
| H(17) | 5606 | 4761 | 1961 | 51 |
| H(18) | 4822 | 5298 | 4312 | 43 |
| H(19) | 5968 | 6536 | 5338 | 39 |
| H(21) | 8441 | 4756 | 8795 | 50 |
| H(22) | 7749 | 4201 | 7215 | 43 |
| H(24) | 7439 | 1421 | 8126 | 49 |
| H(25) | 8149 | 1982 | 9696 | 54 |
| H(27) | 5415 | 1133 | 6659 | 44 |
| H(28) | 5138 | 935 | 4883 | 45 |
| H(31A) | 5848 | 2281 | 2616 | 49 |
| H(31B) | 5093 | 1557 | 3352 | 49 |
| H(32A) | 7215 | 558 | 3317 | 69 |
| H(32B) | 8069 | 1303 | 2633 | 69 |
| H(33A) | 5033 | 187 | 2116 | 91 |
| H(33B) | 5765 | 983 | 1440 | 91 |

| | | | | |
|---|---|---|---|---|
| H(34A) | 6020 | -597 | 985 | 192 |
| H(34B) | 7210 | -706 | 1951 | 192 |
| H(34C) | 7710 | 56 | 1168 | 192 |
| H(36) | 7931 | 3242 | 2392 | 51 |
| H(37) | 9750 | 4276 | 4635 | 42 |
| H(38) | 9264 | 3847 | 6134 | 41 |

## (E) X-ray crystallographic data of compound (5j).

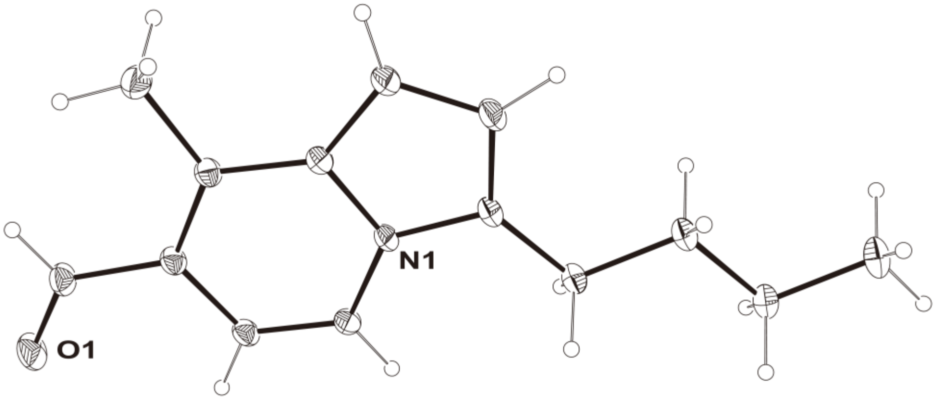


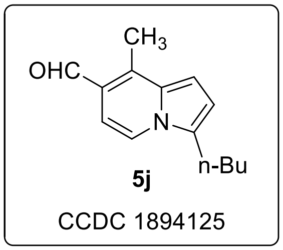


Table S21. Crystal data and structure refinement for d19548.

| | | |
|---|---|---|
| Identification code | d19548 | |
| Empirical formula | C14 H17 N O | |
| Formula weight | 215.29 | |
| Temperature | 200(2) K | |
| Wavelength | 0.71073 Å | |
| Crystal system | Monoclinic | |
| Space group | P 21/c | |
| Unit cell dimensions | a = 7.469(3) Å | $\alpha$= 90°. |
| | b = 7.941(4) Å | $\beta$= 90.051(14)°. |
| | c = 19.929(8) Å | $\gamma$ = 90°. |
| Volume | 1182.0(8) Å$^3$ | |
| Z | 4 | |
| Density (calculated) | 1.210 Mg/m$^3$ | |
| Absorption coefficient | 0.076 mm$^{-1}$ | |
| F(000) | 464 | |

| | |
|---|---|
| Crystal size | 0.65 x 0.30 x 0.11 $mm^3$ |
| Theta range for data collection | 2.73 to 25.13°. |
| Index ranges | -8<=h<=8, -9<=k<=9, -23<=l<=23 |
| Reflections collected | 17667 |
| Independent reflections | 2070 [R(int) = 0.0855] |
| Completeness to theta = 25.13° | 98.3 % |
| Absorption correction | multi-scan |
| Max. and min. transmission | 0.9917 and 0.9524 |
| Refinement method | Full-matrix least-squares on $F^2$ |
| Data / restraints / parameters | 2070 / 0 / 147 |
| Goodness-of-fit on $F^2$ | 1.078 |
| Final R indices [I>2sigma(I)] | R1 = 0.0623, wR2 = 0.1648 |
| R indices (all data) | R1 = 0.0836, wR2 = 0.1940 |
| Largest diff. peak and hole | 0.225 and -0.269 e.$Å^{-3}$ |

Table S22. Atomic coordinates ( x $10^4$) and equivalent isotropic displacement parameters ($Å^2$x $10^3$) for d19548. U(eq) is defined as one third of the trace of the orthogonalized $U^{ij}$ tensor.

| | x | y | z | U(eq) |
|---|---|---|---|---|
| C(1) | 5870(3) | 2093(3) | 2850(1) | 39(1) |
| C(2) | 6346(3) | 2710(3) | 3519(1) | 31(1) |
| C(3) | 6810(3) | 1620(3) | 4034(1) | 32(1) |
| C(4) | 6822(3) | -272(3) | 3983(1) | 44(1) |
| C(5) | 7295(3) | 2312(3) | 4664(1) | 31(1) |
| C(6) | 6799(3) | 5138(3) | 4237(1) | 32(1) |
| C(7) | 6338(3) | 4485(3) | 3637(1) | 33(1) |
| C(8) | 7839(3) | 1617(3) | 5271(1) | 40(1) |
| C(9) | 8165(3) | 2952(3) | 5714(1) | 40(1) |
| C(10) | 7833(3) | 4463(3) | 5393(1) | 32(1) |
| C(11) | 7957(3) | 6232(3) | 5624(1) | 34(1) |
| C(12) | 8493(3) | 6408(3) | 6360(1) | 37(1) |
| C(13) | 8604(3) | 8219(3) | 6595(1) | 39(1) |
| C(14) | 9153(4) | 8377(4) | 7328(1) | 52(1) |
| N(1) | 7302(2) | 4059(2) | 4750(1) | 29(1) |
| O(1) | 5463(3) | 2991(2) | 2382(1) | 54(1) |

Table S23. Bond lengths [Å] and angles [°] for d19548.

| | |
|---|---|
| C(1)-O(1) | 1.212(3) |
| C(1)-C(2) | 1.465(3) |
| C(1)-H(1) | 0.9500 |
| C(2)-C(3) | 1.386(3) |
| C(2)-C(7) | 1.429(3) |
| C(3)-C(5) | 1.417(3) |
| C(3)-C(4) | 1.505(3) |
| C(4)-H(4A) | 0.9800 |
| C(4)-H(4B) | 0.9800 |
| C(4)-H(4C) | 0.9800 |
| C(5)-C(8) | 1.391(3) |
| C(5)-N(1) | 1.397(3) |
| C(6)-C(7) | 1.349(3) |
| C(6)-N(1) | 1.385(3) |
| C(6)-H(6) | 0.9500 |
| C(7)-H(7) | 0.9500 |
| C(8)-C(9) | 1.400(3) |
| C(8)-H(8) | 0.9500 |
| C(9)-C(10) | 1.382(3) |
| C(9)-H(9) | 0.9500 |
| C(10)-N(1) | 1.378(3) |
| C(10)-C(11) | 1.481(3) |
| C(11)-C(12) | 1.528(3) |
| C(11)-H(11A) | 0.9900 |
| C(11)-H(11B) | 0.9900 |
| C(12)-C(13) | 1.514(3) |
| C(12)-H(12A) | 0.9900 |
| C(12)-H(12B) | 0.9900 |
| C(13)-C(14) | 1.523(3) |
| C(13)-H(13A) | 0.9900 |
| C(13)-H(13B) | 0.9900 |
| C(14)-H(14A) | 0.9800 |
| C(14)-H(14B) | 0.9800 |
| C(14)-H(14C) | 0.9800 |

| | |
|---|---|
| O(1)-C(1)-C(2) | 124.3(2) |
| O(1)-C(1)-H(1) | 117.8 |
| C(2)-C(1)-H(1) | 117.8 |
| C(3)-C(2)-C(7) | 119.75(19) |
| C(3)-C(2)-C(1) | 121.7(2) |
| C(7)-C(2)-C(1) | 118.53(19) |
| C(2)-C(3)-C(5) | 118.5(2) |
| C(2)-C(3)-C(4) | 125.1(2) |
| C(5)-C(3)-C(4) | 116.44(19) |
| C(3)-C(4)-H(4A) | 109.5 |
| C(3)-C(4)-H(4B) | 109.5 |
| H(4A)-C(4)-H(4B) | 109.5 |
| C(3)-C(4)-H(4C) | 109.5 |
| H(4A)-C(4)-H(4C) | 109.5 |
| H(4B)-C(4)-H(4C) | 109.5 |
| C(8)-C(5)-N(1) | 106.63(18) |
| C(8)-C(5)-C(3) | 133.7(2) |
| N(1)-C(5)-C(3) | 119.66(18) |
| C(7)-C(6)-N(1) | 119.1(2) |
| C(7)-C(6)-H(6) | 120.5 |
| N(1)-C(6)-H(6) | 120.5 |
| C(6)-C(7)-C(2) | 121.5(2) |
| C(6)-C(7)-H(7) | 119.2 |
| C(2)-C(7)-H(7) | 119.2 |
| C(5)-C(8)-C(9) | 107.3(2) |
| C(5)-C(8)-H(8) | 126.3 |
| C(9)-C(8)-H(8) | 126.3 |
| C(10)-C(9)-C(8) | 109.5(2) |
| C(10)-C(9)-H(9) | 125.2 |
| C(8)-C(9)-H(9) | 125.2 |
| N(1)-C(10)-C(9) | 106.2(2) |
| N(1)-C(10)-C(11) | 121.81(19) |
| C(9)-C(10)-C(11) | 131.9(2) |
| C(10)-C(11)-C(12) | 113.65(19) |
| C(10)-C(11)-H(11A) | 108.8 |

| | |
|---|---|
| C(12)-C(11)-H(11A) | 108.8 |
| C(10)-C(11)-H(11B) | 108.8 |
| C(12)-C(11)-H(11B) | 108.8 |
| H(11A)-C(11)-H(11B) | 107.7 |
| C(13)-C(12)-C(11) | 113.42(19) |
| C(13)-C(12)-H(12A) | 108.9 |
| C(11)-C(12)-H(12A) | 108.9 |
| C(13)-C(12)-H(12B) | 108.9 |
| C(11)-C(12)-H(12B) | 108.9 |
| H(12A)-C(12)-H(12B) | 107.7 |
| C(12)-C(13)-C(14) | 112.9(2) |
| C(12)-C(13)-H(13A) | 109.0 |
| C(14)-C(13)-H(13A) | 109.0 |
| C(12)-C(13)-H(13B) | 109.0 |
| C(14)-C(13)-H(13B) | 109.0 |
| H(13A)-C(13)-H(13B) | 107.8 |
| C(13)-C(14)-H(14A) | 109.5 |
| C(13)-C(14)-H(14B) | 109.5 |
| H(14A)-C(14)-H(14B) | 109.5 |
| C(13)-C(14)-H(14C) | 109.5 |
| H(14A)-C(14)-H(14C) | 109.5 |
| H(14B)-C(14)-H(14C) | 109.5 |
| C(10)-N(1)-C(6) | 128.2(2) |
| C(10)-N(1)-C(5) | 110.25(17) |
| C(6)-N(1)-C(5) | 121.50(18) |

Symmetry transformations used to generate equivalent atoms:

Table S24. Anisotropic displacement parameters ($Å^2 x 10^3$)for d19548. The anisotropic displacement factor exponent takes the form: $-2\pi^2[ h^2 a^{*2} U^{11} + ... + 2 h k a^* b^* U^{12} ]$

| | $U^{11}$ | $U^{22}$ | $U^{33}$ | $U^{23}$ | $U^{13}$ | $U^{12}$ |
|---|---|---|---|---|---|---|
| C(1) | 45(1) | 43(1) | 30(1) | -4(1) | -9(1) | -1(1) |
| C(2) | 32(1) | 36(1) | 26(1) | 1(1) | -6(1) | -1(1) |
| C(3) | 32(1) | 35(1) | 28(1) | 1(1) | -6(1) | 0(1) |
| C(4) | 55(1) | 37(1) | 41(1) | -2(1) | -11(1) | -2(1) |
| C(5) | 35(1) | 33(1) | 26(1) | 2(1) | -3(1) | 0(1) |
| C(6) | 38(1) | 29(1) | 28(1) | 5(1) | -6(1) | 2(1) |
| C(7) | 34(1) | 38(1) | 26(1) | 5(1) | -5(1) | -1(1) |
| C(8) | 56(1) | 35(1) | 29(1) | 5(1) | -8(1) | 1(1) |
| C(9) | 51(1) | 43(1) | 25(1) | 2(1) | -9(1) | 2(1) |
| C(10) | 35(1) | 41(1) | 21(1) | 0(1) | -3(1) | 1(1) |
| C(11) | 37(1) | 39(1) | 25(1) | -2(1) | -4(1) | 2(1) |
| C(12) | 37(1) | 47(1) | 26(1) | -2(1) | -6(1) | 2(1) |
| C(13) | 39(1) | 48(1) | 31(1) | -8(1) | -5(1) | 0(1) |
| C(14) | 59(2) | 65(2) | 33(1) | -14(1) | -9(1) | 1(1) |
| N(1) | 34(1) | 33(1) | 21(1) | 3(1) | -6(1) | 1(1) |
| O(1) | 77(1) | 56(1) | 30(1) | 2(1) | -19(1) | 5(1) |

Table S25. Hydrogen coordinates ( x $10^4$) and isotropic displacement parameters ($Å^2$x $10^3$) for d19548.

| | x | y | z | U(eq) |
|---|---|---|---|---|
| H(1) | 5880 | 911 | 2777 | 47 |
| H(4A) | 5915 | -740 | 4285 | 66 |
| H(4B) | 8006 | -699 | 4111 | 66 |
| H(4C) | 6555 | -607 | 3521 | 66 |
| H(6) | 6779 | 6321 | 4308 | 38 |
| H(7) | 6000 | 5223 | 3283 | 39 |
| H(8) | 7966 | 451 | 5368 | 48 |
| H(9) | 8554 | 2837 | 6166 | 48 |
| H(11A) | 6782 | 6785 | 5555 | 40 |
| H(11B) | 8845 | 6832 | 5343 | 40 |
| H(12A) | 7609 | 5802 | 6641 | 44 |
| H(12B) | 9672 | 5864 | 6428 | 44 |
| H(13A) | 7422 | 8762 | 6531 | 47 |
| H(13B) | 9482 | 8829 | 6313 | 47 |
| H(14A) | 10333 | 7861 | 7393 | 78 |
| H(14B) | 8273 | 7800 | 7611 | 78 |
| H(14C) | 9208 | 9570 | 7453 | 78 |

## (F) X-ray crystallographic data of compound (7b).

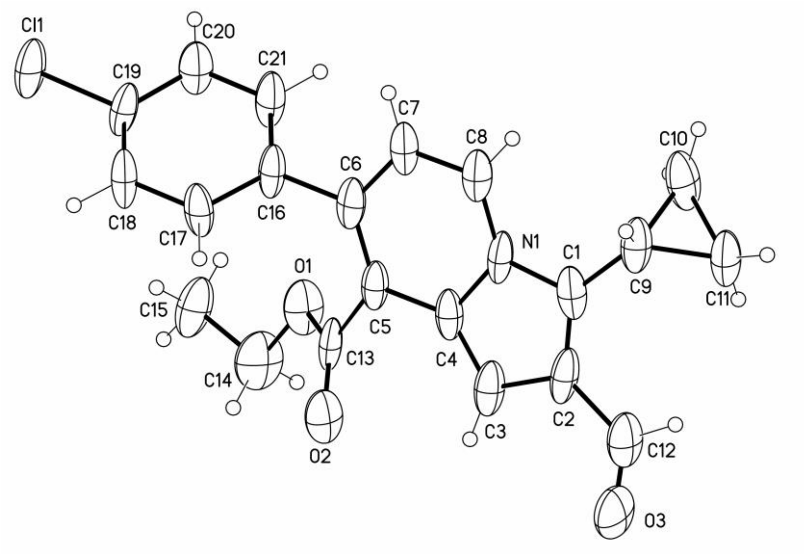


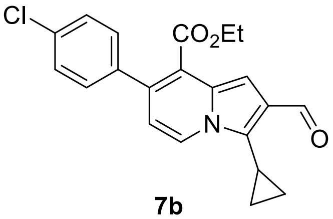


**7b**

CCDC 1913325

Table S26. Crystal data and structure refinement for 190231lt_0m_a.

| | | |
|---|---|---|
| Identification code | 190231lt_0m_a | |
| Empirical formula | C21 H18 Cl N O3 | |
| Formula weight | 367.81 | |
| Temperature | 100(2) K | |
| Wavelength | 0.71073 Å | |
| Crystal system | Monoclinic | |
| Space group | P 21/c | |
| Unit cell dimensions | a = 13.421(2) Å | a= 90°. |
| | b = 9.1933(15) Å | b= 91.484(4)°. |
| | c = 14.5321(18) Å | g = 90°. |
| Volume | 1792.4(5) Å$^3$ | |
| Z | 4 | |

| | |
|---|---|
| Density (calculated) | 1.363 $Mg/m^3$ |
| Absorption coefficient | 0.234 $mm^{-1}$ |
| F(000) | 768 |
| Crystal size | 0.12 x 0.10 x 0.03 $mm^3$ |
| Theta range for data collection | 1.518 to 26.446°. |
| Index ranges | -16<=h<=16, -11<=k<=11, -18<=l<=10 |
| Reflections collected | 9874 |
| Independent reflections | 3700 [R(int) = 0.0336] |
| Completeness to theta = 25.242° | 100.0 % |
| Absorption correction | Semi-empirical from equivalents |
| Max. and min. transmission | 0.7454 and 0.6583 |
| Refinement method | Full-matrix least-squares on $F^2$ |
| Data / restraints / parameters | 3700 / 0 / 237 |
| Goodness-of-fit on $F^2$ | 2.432 |
| Final R indices [I>2sigma(I)] | R1 = 0.1746, wR2 = 0.4876 |
| R indices (all data) | R1 = 0.1943, wR2 = 0.5549 |
| Extinction coefficient | 0.06(2) |
| Largest diff. peak and hole | 1.805 and -2.806 e.$Å^{-3}$ |

Table S27. Atomic coordinates ( x $10^4$) and equivalent isotropic displacement parameters ($Å^2$x $10^3$) for 190231lt_0m_a. U(eq) is defined as one third of the trace of the orthogonalized $U^{ij}$ tensor.

| | x | y | z | U(eq) |
|---|---|---|---|---|
| C(1) | 8515(4) | 6420(7) | 2817(5) | 46(2) |
| C(2) | 8424(4) | 6637(7) | 3763(5) | 49(2) |
| C(3) | 7419(4) | 6835(7) | 3969(5) | 48(2) |
| C(4) | 6876(4) | 6749(6) | 3163(4) | 40(1) |
| C(5) | 5845(4) | 6772(6) | 2911(4) | 39(1) |
| C(6) | 5546(4) | 6686(6) | 1984(4) | 41(1) |
| C(7) | 6286(4) | 6509(7) | 1301(4) | 45(2) |
| C(8) | 7269(4) | 6421(7) | 1546(4) | 46(2) |
| C(9) | 9360(4) | 6126(7) | 2230(5) | 49(2) |
| C(10) | 9450(4) | 4665(8) | 1768(5) | 58(2) |
| C(11) | 10162(5) | 5060(8) | 2539(5) | 55(2) |
| C(12) | 9245(5) | 6672(8) | 4438(5) | 56(2) |
| C(13) | 5138(4) | 6748(6) | 3685(4) | 42(2) |
| C(14) | 3639(5) | 5723(11) | 4242(6) | 70(2) |
| C(15) | 2656(5) | 5481(10) | 3755(5) | 63(2) |
| C(16) | 4488(4) | 6797(6) | 1683(5) | 44(2) |
| C(17) | 3912(5) | 7961(6) | 1952(5) | 46(2) |
| C(18) | 2921(5) | 8080(7) | 1649(5) | 49(2) |
| C(19) | 2522(4) | 7027(7) | 1089(5) | 45(2) |
| C(20) | 3082(5) | 5859(7) | 793(4) | 50(2) |
| C(21) | 4073(5) | 5752(7) | 1093(5) | 55(2) |
| Cl(1) | 1292(1) | 7196(2) | 686(1) | 58(1) |
| N(1) | 7566(3) | 6523(5) | 2465(4) | 44(1) |
| O(1) | 4384(3) | 5836(5) | 3514(3) | 54(1) |
| O(2) | 5265(3) | 7413(5) | 4388(3) | 54(1) |
| O(3) | 9137(4) | 6938(7) | 5250(4) | 64(2) |

Table S28. Bond lengths [Å] and angles [°] for 190231lt_0m_a.

| | |
|---|---|
| C(1)-N(1) | 1.363(8) |
| C(1)-C(2) | 1.398(10) |
| C(1)-C(9) | 1.461(8) |
| C(2)-C(3) | 1.401(9) |
| C(2)-C(12) | 1.456(9) |
| C(3)-C(4) | 1.365(9) |
| C(3)-H(3) | 0.9500 |
| C(4)-N(1) | 1.406(8) |
| C(4)-C(5) | 1.422(8) |
| C(5)-C(6) | 1.399(8) |
| C(5)-C(13) | 1.490(8) |
| C(6)-C(7) | 1.432(8) |
| C(6)-C(16) | 1.479(8) |
| C(7)-C(8) | 1.360(8) |
| C(7)-H(7) | 0.9500 |
| C(8)-N(1) | 1.388(8) |
| C(8)-H(8) | 0.9500 |
| C(9)-C(10) | 1.508(10) |
| C(9)-C(11) | 1.516(9) |
| C(9)-H(9) | 1.0000 |
| C(10)-C(11) | 1.497(9) |
| C(10)-H(10A) | 0.9900 |
| C(10)-H(10B) | 0.9900 |
| C(11)-H(11A) | 0.9900 |
| C(11)-H(11B) | 0.9900 |
| C(12)-O(3) | 1.218(9) |
| C(12)-H(12) | 0.9500 |
| C(13)-O(2) | 1.199(8) |
| C(13)-O(1) | 1.333(7) |
| C(14)-O(1) | 1.478(8) |
| C(14)-C(15) | 1.497(10) |
| C(14)-H(14A) | 0.9900 |
| C(14)-H(14B) | 0.9900 |
| C(15)-H(15A) | 0.9800 |

| | |
|---|---|
| C(15)-H(15B) | 0.9800 |
| C(15)-H(15C) | 0.9800 |
| C(16)-C(17) | 1.382(8) |
| C(16)-C(21) | 1.393(9) |
| C(17)-C(18) | 1.394(9) |
| C(17)-H(17) | 0.9500 |
| C(18)-C(19) | 1.365(10) |
| C(18)-H(18) | 0.9500 |
| C(19)-C(20) | 1.385(9) |
| C(19)-Cl(1) | 1.744(6) |
| C(20)-C(21) | 1.392(9) |
| C(20)-H(20) | 0.9500 |
| C(21)-H(21) | 0.9500 |

| | |
|---|---|
| N(1)-C(1)-C(2) | 104.7(5) |
| N(1)-C(1)-C(9) | 121.7(6) |
| C(2)-C(1)-C(9) | 133.5(6) |
| C(1)-C(2)-C(3) | 109.7(6) |
| C(1)-C(2)-C(12) | 125.6(6) |
| C(3)-C(2)-C(12) | 124.7(7) |
| C(4)-C(3)-C(2) | 107.8(6) |
| C(4)-C(3)-H(3) | 126.1 |
| C(2)-C(3)-H(3) | 126.1 |
| C(3)-C(4)-N(1) | 106.3(5) |
| C(3)-C(4)-C(5) | 135.6(6) |
| N(1)-C(4)-C(5) | 118.1(5) |
| C(6)-C(5)-C(4) | 120.1(5) |
| C(6)-C(5)-C(13) | 123.6(5) |
| C(4)-C(5)-C(13) | 116.1(5) |
| C(5)-C(6)-C(7) | 119.1(6) |
| C(5)-C(6)-C(16) | 122.1(6) |
| C(7)-C(6)-C(16) | 118.8(5) |
| C(8)-C(7)-C(6) | 120.8(6) |
| C(8)-C(7)-H(7) | 119.6 |
| C(6)-C(7)-H(7) | 119.6 |
| C(7)-C(8)-N(1) | 120.0(5) |

| | |
|---|---|
| C(7)-C(8)-H(8) | 120.0 |
| N(1)-C(8)-H(8) | 120.0 |
| C(1)-C(9)-C(10) | 119.8(5) |
| C(1)-C(9)-C(11) | 120.2(6) |
| C(10)-C(9)-C(11) | 59.3(4) |
| C(1)-C(9)-H(9) | 115.3 |
| C(10)-C(9)-H(9) | 115.3 |
| C(11)-C(9)-H(9) | 115.3 |
| C(11)-C(10)-C(9) | 60.6(4) |
| C(11)-C(10)-H(10A) | 117.7 |
| C(9)-C(10)-H(10A) | 117.7 |
| C(11)-C(10)-H(10B) | 117.7 |
| C(9)-C(10)-H(10B) | 117.7 |
| H(10A)-C(10)-H(10B) | 114.8 |
| C(10)-C(11)-C(9) | 60.1(4) |
| C(10)-C(11)-H(11A) | 117.8 |
| C(9)-C(11)-H(11A) | 117.8 |
| C(10)-C(11)-H(11B) | 117.8 |
| C(9)-C(11)-H(11B) | 117.8 |
| H(11A)-C(11)-H(11B) | 114.9 |
| O(3)-C(12)-C(2) | 123.4(7) |
| O(3)-C(12)-H(12) | 118.3 |
| C(2)-C(12)-H(12) | 118.3 |
| O(2)-C(13)-O(1) | 124.7(6) |
| O(2)-C(13)-C(5) | 123.8(5) |
| O(1)-C(13)-C(5) | 111.4(5) |
| O(1)-C(14)-C(15) | 106.0(6) |
| O(1)-C(14)-H(14A) | 110.5 |
| C(15)-C(14)-H(14A) | 110.5 |
| O(1)-C(14)-H(14B) | 110.5 |
| C(15)-C(14)-H(14B) | 110.5 |
| H(14A)-C(14)-H(14B) | 108.7 |
| C(14)-C(15)-H(15A) | 109.5 |
| C(14)-C(15)-H(15B) | 109.5 |
| H(15A)-C(15)-H(15B) | 109.5 |
| C(14)-C(15)-H(15C) | 109.5 |

| | |
|---|---|
| H(15A)-C(15)-H(15C) | 109.5 |
| H(15B)-C(15)-H(15C) | 109.5 |
| C(17)-C(16)-C(21) | 119.3(6) |
| C(17)-C(16)-C(6) | 120.6(6) |
| C(21)-C(16)-C(6) | 120.0(5) |
| C(16)-C(17)-C(18) | 120.5(6) |
| C(16)-C(17)-H(17) | 119.8 |
| C(18)-C(17)-H(17) | 119.8 |
| C(19)-C(18)-C(17) | 119.3(6) |
| C(19)-C(18)-H(18) | 120.4 |
| C(17)-C(18)-H(18) | 120.4 |
| C(18)-C(19)-C(20) | 121.8(6) |
| C(18)-C(19)-Cl(1) | 119.4(5) |
| C(20)-C(19)-Cl(1) | 118.8(5) |
| C(19)-C(20)-C(21) | 118.6(6) |
| C(19)-C(20)-H(20) | 120.7 |
| C(21)-C(20)-H(20) | 120.7 |
| C(20)-C(21)-C(16) | 120.5(6) |
| C(20)-C(21)-H(21) | 119.8 |
| C(16)-C(21)-H(21) | 119.8 |
| C(1)-N(1)-C(8) | 126.7(5) |
| C(1)-N(1)-C(4) | 111.5(5) |
| C(8)-N(1)-C(4) | 121.8(5) |
| C(13)-O(1)-C(14) | 115.8(5) |

Symmetry transformations used to generate equivalent atoms:

Table S29. Anisotropic displacement parameters ($Å^2$x $10^3$) for 190231lt_0m_a. The anisotropic displacement factor exponent takes the form: $-2p^2[ h^2 a^{*2}U^{11} + ... + 2 h k a^* b^* U^{12} ]$

| | $U^{11}$ | $U^{22}$ | $U^{33}$ | $U^{23}$ | $U^{13}$ | $U^{12}$ |
|---|---|---|---|---|---|---|
| C(1) | 23(3) | 23(3) | 92(4) | -9(3) | -2(3) | 0(2) |
| C(2) | 23(3) | 23(3) | 100(4) | -1(3) | -21(3) | -2(2) |
| C(3) | 24(3) | 27(3) | 92(4) | 4(3) | -9(3) | -3(2) |
| C(4) | 22(3) | 20(3) | 77(3) | 2(2) | 0(2) | 1(2) |
| C(5) | 21(3) | 19(3) | 75(3) | -1(2) | -7(2) | 0(2) |
| C(6) | 27(3) | 16(3) | 78(3) | 5(2) | -7(2) | 0(2) |
| C(7) | 24(3) | 27(3) | 83(3) | 1(3) | -1(2) | 3(2) |
| C(8) | 26(3) | 30(3) | 81(3) | 1(3) | -4(2) | 2(2) |
| C(9) | 25(3) | 29(3) | 93(4) | 4(3) | -6(3) | -2(2) |
| C(10) | 26(3) | 39(4) | 110(5) | -9(4) | 7(3) | -3(3) |
| C(11) | 27(3) | 39(4) | 99(4) | -5(3) | -7(3) | 6(3) |
| C(12) | 29(4) | 38(4) | 100(5) | -11(3) | -12(3) | -4(3) |
| C(13) | 17(3) | 23(3) | 87(4) | 2(3) | -13(2) | 2(2) |
| C(14) | 38(4) | 73(6) | 98(5) | 1(4) | 0(3) | -20(4) |
| C(15) | 30(4) | 58(5) | 101(4) | 13(4) | -17(3) | -5(3) |
| C(16) | 24(3) | 20(3) | 87(3) | 4(3) | -9(3) | 2(2) |
| C(17) | 29(3) | 20(3) | 90(4) | -6(3) | -1(3) | 3(2) |
| C(18) | 21(3) | 24(3) | 102(4) | 8(3) | 0(3) | 4(2) |
| C(19) | 21(3) | 34(3) | 80(3) | 9(3) | -15(2) | 0(2) |
| C(20) | 32(3) | 30(3) | 89(4) | -5(3) | -8(3) | -1(3) |
| C(21) | 32(3) | 29(3) | 103(4) | -9(3) | -17(3) | 6(3) |
| Cl(1) | 20(1) | 49(1) | 103(1) | 7(1) | -12(1) | 0(1) |
| N(1) | 20(2) | 24(3) | 87(3) | -2(2) | -14(2) | 2(2) |
| O(1) | 32(2) | 37(3) | 92(3) | 0(2) | 1(2) | -12(2) |
| O(2) | 31(3) | 38(3) | 94(3) | -10(2) | -6(2) | -7(2) |
| O(3) | 29(3) | 74(4) | 89(3) | -9(3) | -13(2) | -4(2) |

Table S30. Hydrogen coordinates ( x $10^4$) and isotropic displacement parameters ($Å^2$x $10^3$) for 190231lt_0m_a.

| | x | y | z | U(eq) |
|---|---|---|---|---|
| H(3) | 7161 | 7000 | 4562 | 57 |
| H(7) | 6088 | 6452 | 670 | 53 |
| H(8) | 7753 | 6291 | 1087 | 55 |
| H(9) | 9597 | 6975 | 1865 | 59 |
| H(10A) | 8935 | 3926 | 1893 | 70 |
| H(10B) | 9710 | 4643 | 1137 | 70 |
| H(11A) | 10859 | 5283 | 2382 | 66 |
| H(11B) | 10083 | 4565 | 3138 | 66 |
| H(12) | 9899 | 6477 | 4234 | 67 |
| H(14A) | 3625 | 6628 | 4610 | 84 |
| H(14B) | 3800 | 4898 | 4659 | 84 |
| H(15A) | 2501 | 6317 | 3359 | 95 |
| H(15B) | 2135 | 5369 | 4210 | 95 |
| H(15C) | 2690 | 4599 | 3379 | 95 |
| H(17) | 4193 | 8685 | 2346 | 56 |
| H(18) | 2527 | 8884 | 1831 | 59 |
| H(20) | 2795 | 5145 | 395 | 61 |
| H(21) | 4469 | 4961 | 894 | 66 |

## 7. Cartesian Coordinates for Optimized Structures

**A**

| | | | |
|---|---|---|---|
| C1 | 2.8651898575 | 0.8815578228 | 0.5226673070 |
| C2 | 2.2773073053 | 1.2849595545 | -0.6192363583 |
| C3 | 1.6031370236 | 1.5369007244 | -1.7079764976 |
| C4 | 0.4242174207 | 0.6723039790 | -2.1960347307 |
| H5 | 3.6270301734 | 0.1033375407 | 0.5059824235 |
| H6 | 2.4175997014 | 1.1215311090 | 1.4861371170 |
| H7 | 1.8444244762 | 2.4038132724 | -2.3184219791 |
| H8 | 0.7246883646 | 0.2522519480 | -3.1721543820 |
| C9 | 0.1128219228 | -0.4222044146 | -1.2835104341 |
| C10 | -0.1261229272 | -1.3357857040 | -0.5263559446 |
| C11 | -0.4051601532 | -2.4124370223 | 0.3707231202 |
| C12 | -0.0836002746 | -3.7359965254 | 0.0173988421 |
| C13 | -0.9981055037 | -2.1586686421 | 1.6213223444 |
| C14 | -0.3466380457 | -4.7789452037 | 0.9006254569 |
| H15 | 0.3690882690 | -3.9344633425 | -0.9487515826 |
| C16 | -1.2593765774 | -3.2080894470 | 2.4970432196 |
| H17 | -1.2541422496 | -1.1391530710 | 1.8914718954 |
| C18 | -0.9331660965 | -4.5185563043 | 2.1410339027 |
| H19 | -0.0959544288 | -5.7976479188 | 0.6196195059 |
| H20 | -1.7205614019 | -3.0040298942 | 3.4589498066 |
| H21 | -1.1385713290 | -5.3353104128 | 2.8268753810 |
| O22 | -0.7161236049 | 1.5021135632 | -2.3585584399 |
| C23 | -0.7795550461 | 2.1749391471 | -3.6087140733 |
| H24 | -1.7336923905 | 2.7046052014 | -3.6260502420 |
| H25 | 0.0301684367 | 2.9099042540 | -3.7334909435 |

| | | | |
|---|---|---|---|
| H26 | -0.7468913769 | 1.4667808545 | -4.4488759768 |
| Au27 | 4.0362122049 | 2.7933495115 | -0.0137722677 |
| C28 | 6.2561043013 | 6.3057564813 | -0.8886698417 |
| C29 | 6.9460214759 | 5.8745781962 | 0.1984508569 |
| H30 | 6.3906553408 | 7.1651910666 | -1.5258207435 |
| H31 | 7.8064804857 | 6.2804057315 | 0.7058838892 |
| C32 | 5.2758715007 | 4.4065696849 | -0.1711676768 |
| N33 | 5.2321970877 | 5.3935846452 | -1.1012030276 |
| N34 | 6.3293731361 | 4.7083275385 | 0.6271504213 |
| C35 | 6.7499363513 | 3.9400324900 | 1.7801173415 |
| C36 | 7.7268768749 | 2.9403716102 | 1.6003141359 |
| C37 | 6.1741290155 | 4.2468831619 | 3.0288397937 |
| C38 | 8.1084245639 | 2.2154768731 | 2.7352770914 |
| C39 | 6.5954580391 | 3.4866709664 | 4.1258285178 |
| C40 | 7.5477731750 | 2.4815725836 | 3.9814235401 |
| H41 | 8.8581591406 | 1.4356020618 | 2.6424555572 |
| H42 | 6.1764944495 | 3.6891556679 | 5.1069281197 |
| H43 | 7.8614152070 | 1.9068093766 | 4.8481833834 |
| C44 | 4.2549380209 | 5.5085969566 | -2.1626260610 |
| C45 | 4.5380741563 | 4.9028047811 | -3.4030537537 |
| C46 | 3.0832828073 | 6.2508189538 | -1.9125227594 |
| C47 | 3.5806912170 | 5.0494020204 | -4.4141730809 |
| C48 | 2.1646468839 | 6.3630045624 | -2.9628021630 |
| C49 | 2.4084638208 | 5.7690983317 | -4.1980813977 |
| H50 | 3.7609736270 | 4.6032332350 | -5.3872487845 |
| H51 | 1.2503668127 | 6.9291865301 | -2.8126998784 |
| H52 | 1.6854111165 | 5.8777427619 | -5.0017162100 |
| C53 | 2.7937177809 | 6.9261626126 | -0.5738796876 |
| H54 | 3.6386071080 | 6.7385041412 | 0.0960215969 |

| | | | |
|---|---|---|---|
| C55 | 5.8234767306 | 4.1244956242 | -3.6768448150 |
| H56 | 6.4357709280 | 4.1431711071 | -2.7699855576 |
| C57 | 2.6677931439 | 8.4547963828 | -0.7307943599 |
| H58 | 1.8128096003 | 8.7268060575 | -1.3582757009 |
| H59 | 2.5224793442 | 8.9259189018 | 0.2467916884 |
| H60 | 3.5647857744 | 8.8875968215 | -1.1852872153 |
| C61 | 1.5404954475 | 6.3310595965 | 0.0972366529 |
| H62 | 0.6422058160 | 6.5025088539 | -0.5050516694 |
| H63 | 1.6429252397 | 5.2509258636 | 0.2468311416 |
| H64 | 1.3755687604 | 6.7953864072 | 1.0750971405 |
| C65 | 5.5309441139 | 2.6449372375 | -3.9956521227 |
| H66 | 4.9838686229 | 2.1617328785 | -3.1784252555 |
| H67 | 4.9349533500 | 2.5401135001 | -4.9084680955 |
| H68 | 6.4660753179 | 2.0954363473 | -4.1471036676 |
| C69 | 6.6512182446 | 4.7821495649 | -4.7992760576 |
| H70 | 7.5960078458 | 4.2464832193 | -4.9383320784 |
| H71 | 6.1185684135 | 4.7660750300 | -5.7558626933 |
| H72 | 6.8851349536 | 5.8259426866 | -4.5667041204 |
| C73 | 8.3706104637 | 2.6374139963 | 0.2482826421 |
| H74 | 7.9584503376 | 3.3305740982 | -0.4918072911 |
| C75 | 5.1437042161 | 5.3583437684 | 3.2194430529 |
| H76 | 4.9775463746 | 5.8428409704 | 2.2520568958 |
| C77 | 3.7859103505 | 4.7973353958 | 3.6848326682 |
| H78 | 3.3950840114 | 4.0611246226 | 2.9738993768 |
| H79 | 3.8660386285 | 4.3104911008 | 4.6623114723 |
| H80 | 3.0509838380 | 5.6039258262 | 3.7761407078 |
| C81 | 5.6571483718 | 6.4432177011 | 4.1870384286 |
| H82 | 6.6083935914 | 6.8657619519 | 3.8481050584 |
| H83 | 4.9311889796 | 7.2595282595 | 4.2613862097 |

| | | | |
|---|---|---|---|
| H84 | 5.8102903035 | 6.0449667414 | 5.1952924814 |
| C85 | 9.8947145287 | 2.8679412854 | 0.2839701203 |
| H86 | 10.3917253717 | 2.1770889310 | 0.9726536514 |
| H87 | 10.3260979450 | 2.7101972178 | -0.7100393904 |
| H88 | 10.1393692490 | 3.8867156919 | 0.6013303221 |
| C89 | 8.0352068089 | 1.2107302496 | -0.2295122872 |
| H90 | 8.4358818201 | 0.4536285225 | 0.4526428698 |
| H91 | 6.9524247805 | 1.0593986015 | -0.2999094125 |
| H92 | 8.4686633568 | 1.0277097804 | -1.2184409335 |

**AB-TS**

| | | | |
|---|---|---|---|
| C1 | 2.7158356927 | 0.4793390408 | 0.1989515751 |
| C2 | 2.8240438109 | 1.5339912226 | -0.7212136851 |
| C3 | 1.8641923302 | 2.0191497169 | -1.4985126637 |
| C4 | 0.4666610465 | 1.4243091674 | -1.3237382099 |
| H5 | 3.0572293210 | -0.5187499752 | -0.0708655296 |
| H6 | 2.6798824867 | 0.6845251249 | 1.2673690566 |
| H7 | 1.9748820481 | 2.8310766041 | -2.2081224411 |
| H8 | 0.1017361511 | 1.0268496759 | -2.2876607066 |
| C9 | 0.5417195217 | 0.3014270586 | -0.3539122764 |
| C10 | 0.3413820847 | -0.6291643050 | 0.4199505721 |
| C11 | 0.1893293265 | -1.6932118635 | 1.3483671226 |
| C12 | 0.3765149287 | -3.0302558378 | 0.9390743125 |
| C13 | -0.1367032652 | -1.4132277393 | 2.6924299062 |
| C14 | 0.2455821503 | -4.0613668271 | 1.8623619351 |
| H15 | 0.6200759427 | -3.2434631342 | -0.0970336603 |
| C16 | -0.2648810006 | -2.4534936571 | 3.6053924921 |

| | | | |
|---|---|---|---|
| H17 | -0.2872930201 | -0.3835029307 | 3.0012518088 |
| C18 | -0.0724027263 | -3.7752480904 | 3.1931680989 |
| H19 | 0.3899587308 | -5.0899814048 | 1.5467405875 |
| H20 | -0.5158639220 | -2.2366847971 | 4.6391522028 |
| H21 | -0.1728900896 | -4.5845747881 | 3.9104286811 |
| O22 | -0.3842746556 | 2.4614515210 | -0.8800966589 |
| C23 | -1.7726623930 | 2.1617012961 | -0.9846126813 |
| H24 | -2.3085490644 | 3.0627733016 | -0.6821306468 |
| H25 | -2.0503414517 | 1.9040971033 | -2.0168025122 |
| H26 | -2.0584413557 | 1.3331750364 | -0.3222226339 |
| Au27 | 4.8180315499 | 2.1359219653 | -0.4226621709 |
| C28 | 8.7243763759 | 3.3982705495 | 0.7613344625 |
| C29 | 8.5686436720 | 4.1630733505 | -0.3495655911 |
| H30 | 9.5331417185 | 3.3259938268 | 1.4707105575 |
| H31 | 9.2124965374 | 4.8966390869 | -0.8077828236 |
| C32 | 6.7112649753 | 2.8945937901 | -0.1329583622 |
| N33 | 7.5768576060 | 2.6254891872 | 0.8788767184 |
| N34 | 7.3299309497 | 3.8400224661 | -0.8852058636 |
| C35 | 6.7862758573 | 4.4124209573 | -2.0975098438 |
| C36 | 5.9837159201 | 5.5654832864 | -1.9963583708 |
| C37 | 7.0914860531 | 3.7886661173 | -3.3238188611 |
| C38 | 5.4646383915 | 6.0858731425 | -3.1875412990 |
| C39 | 6.5460001105 | 4.3563232851 | -4.4806938977 |
| C40 | 5.7398255092 | 5.4889925121 | -4.4143682745 |
| H41 | 4.8392216235 | 6.9729480893 | -3.1532864235 |
| H42 | 6.7561377959 | 3.9051903767 | -5.4459052619 |
| H43 | 5.3273194169 | 5.9122677428 | -5.3258580626 |
| C44 | 7.3686468228 | 1.6379402944 | 1.9151532474 |
| C45 | 6.7312533981 | 2.0322716699 | 3.1074698526 |

| | | | |
|---|---|---|---|
| C46 | 7.8381900436 | 0.3282443298 | 1.6894199411 |
| C47 | 6.5599792630 | 1.0529479953 | 4.0932130867 |
| C48 | 7.6390606988 | -0.6069665482 | 2.7113162715 |
| C49 | 7.0069512452 | -0.2506703772 | 3.8990974284 |
| H50 | 6.0765954856 | 1.3183175815 | 5.0288390570 |
| H51 | 7.9889334709 | -1.6262079583 | 2.5764097513 |
| H52 | 6.8688864946 | -0.9919049084 | 4.6813618854 |
| C53 | 6.2413323019 | 3.4558625402 | 3.3596460468 |
| H54 | 6.4718556868 | 4.0582756208 | 2.4762029930 |
| C55 | 4.7125695654 | 3.4986094479 | 3.5519444319 |
| H56 | 4.1930467052 | 3.0943068029 | 2.6762972945 |
| H57 | 4.4009732677 | 2.9198377594 | 4.4282256598 |
| H58 | 4.3747971021 | 4.5299298920 | 3.6990731834 |
| C59 | 6.9715923624 | 4.0986593061 | 4.5558316802 |
| H60 | 8.0567584164 | 4.0969001870 | 4.4109071359 |
| H61 | 6.6493154369 | 5.1371475855 | 4.6856659550 |
| H62 | 6.7604811517 | 3.5676176538 | 5.4900673843 |
| C63 | 8.5410439282 | -0.0937849215 | 0.4007184172 |
| H64 | 8.6191959752 | 0.7812416956 | -0.2513420570 |
| C65 | 7.7238482779 | -1.1554644604 | -0.3617537162 |
| H66 | 7.6155263918 | -2.0749703490 | 0.2235196721 |
| H67 | 6.7206517623 | -0.7862005367 | -0.6004156439 |
| H68 | 8.2217165414 | -1.4177260316 | -1.3015279360 |
| C69 | 9.9762052723 | -0.5859878433 | 0.6738565864 |
| H70 | 10.4819588977 | -0.8243911289 | -0.2677328464 |
| H71 | 10.5690522948 | 0.1739484990 | 1.1930023051 |
| H72 | 9.9829001553 | -1.4909904846 | 1.2902572703 |
| C73 | 5.6732757230 | 6.2497505847 | -0.6671642433 |
| H74 | 6.1867648397 | 5.7015644273 | 0.1289567963 |

| | | | |
|---|---|---|---|
| C75 | 6.2062070692 | 7.6958837786 | -0.6409513550 |
| H76 | 5.7087627898 | 8.3220027309 | -1.3889340789 |
| H77 | 6.0285189187 | 8.1488269133 | 0.3400710483 |
| H78 | 7.2820594498 | 7.7307840732 | -0.8405186396 |
| C79 | 4.1654350365 | 6.2067524825 | -0.3507896682 |
| H80 | 3.5826791347 | 6.7541575209 | -1.0991142675 |
| H81 | 3.7939857175 | 5.1767459800 | -0.3233381718 |
| H82 | 3.9670883437 | 6.6658383844 | 0.6236344432 |
| C83 | 7.9715541113 | 2.5448720742 | -3.4285508386 |
| H84 | 8.3137235903 | 2.2797266418 | -2.4234630517 |
| C85 | 7.1771103744 | 1.3401038804 | -3.9698228376 |
| H86 | 6.3082609280 | 1.1227140079 | -3.3395859247 |
| H87 | 6.8168875539 | 1.5219810656 | -4.9877940676 |
| H88 | 7.8109609957 | 0.4471260146 | -3.9974087040 |
| C89 | 9.2285335755 | 2.8102597367 | -4.2803366466 |
| H90 | 9.8118164061 | 3.6480938263 | -3.8849591524 |
| H91 | 9.8731468067 | 1.9250289101 | -4.2926598611 |
| H92 | 8.9707141262 | 3.0448825252 | -5.3182462446 |

**B**

| | | | |
|---|---|---|---|
| C1 | 4.0128649356 | 0.6392856083 | -0.6431596473 |
| C2 | 4.7619797343 | 1.5804568952 | -1.5590312444 |
| C3 | 4.1890247261 | 1.6483878385 | -2.7645087721 |
| C4 | 2.9337724260 | 0.8079991683 | -2.9471401444 |
| H5 | 4.6293338645 | -0.1811736233 | -0.2624429001 |
| H6 | 3.5714642196 | 1.1483288650 | 0.2211791038 |
| H7 | 4.5208795025 | 2.2638220054 | -3.5962527491 |
| H8 | 3.0217249901 | 0.0693572387 | -3.7603072407 |

| | | | |
|---|---|---|---|
| C9 | 2.8872305918 | 0.0775294768 | -1.5672519473 |
| C10 | 2.0557753330 | -0.8444975256 | -1.2803027283 |
| C11 | 1.1051915740 | -1.7995547772 | -0.9808917550 |
| C12 | 1.3769717067 | -3.1826182966 | -1.1962239511 |
| C13 | -0.1685302264 | -1.3985052210 | -0.4789065137 |
| C14 | 0.4021381509 | -4.1242863558 | -0.9211017695 |
| H15 | 2.3485872746 | -3.4756325659 | -1.5789600075 |
| C16 | -1.1313388002 | -2.3557747585 | -0.2105132633 |
| H17 | -0.3636291998 | -0.3423646513 | -0.3248960485 |
| C18 | -0.8464392508 | -3.7112687520 | -0.4320213351 |
| H19 | 0.5994168144 | -5.1785721644 | -1.0847446027 |
| H20 | -2.1044574420 | -2.0599104807 | 0.1672909484 |
| H21 | -1.6080207602 | -4.4565397660 | -0.2208186260 |
| O22 | 1.7528126557 | 1.5567450983 | -3.1189658169 |
| C23 | 1.4107615354 | 1.8016053920 | -4.4795702344 |
| H24 | 0.4498642804 | 2.3191501917 | -4.4713834018 |
| H25 | 2.1526396729 | 2.4382466905 | -4.9788795337 |
| H26 | 1.3117595295 | 0.8629154230 | -5.0445526422 |
| Au27 | 6.4101854470 | 2.6267771841 | -0.9241981048 |
| C28 | 9.7327851375 | 4.5885783977 | 0.9369832796 |
| C29 | 9.7416644197 | 5.2380312904 | -0.2546567333 |
| H30 | 10.3599278193 | 4.6876546090 | 1.8084499829 |
| H31 | 10.3785587976 | 6.0198766120 | -0.6366476933 |
| C32 | 8.0473129009 | 3.7345650984 | -0.3140654424 |
| N33 | 8.6900806794 | 3.6726471922 | 0.8827810777 |
| N34 | 8.7050164452 | 4.7019126179 | -1.0076113060 |
| C35 | 8.3483415299 | 2.7657424976 | 1.9544655266 |
| C36 | 7.4014141272 | 3.1776998731 | 2.9118603598 |
| C37 | 8.9772030325 | 1.5056065336 | 1.9893866488 |

| | | | |
|---|---|---|---|
| C38 | 7.0930235961 | 2.2751768091 | 3.9363784920 |
| C39 | 8.6301233604 | 0.6451906993 | 3.0372053076 |
| C40 | 7.7004903065 | 1.0246800362 | 4.0009573731 |
| H41 | 6.3696354483 | 2.5579466646 | 4.6955773518 |
| H42 | 9.0955470694 | -0.3342233873 | 3.0990007017 |
| H43 | 7.4490371893 | 0.3421150309 | 4.8081556759 |
| C44 | 8.3815775411 | 5.1149301268 | -2.3543595433 |
| C45 | 9.0123119623 | 4.4538181908 | -3.4264963515 |
| C46 | 7.4589531670 | 6.1638668953 | -2.5338684880 |
| C47 | 8.6867432997 | 4.8774659365 | -4.7198377237 |
| C48 | 7.1738677815 | 6.5463284461 | -3.8499327252 |
| C49 | 7.7787720872 | 5.9109511492 | -4.9305199252 |
| H50 | 9.1537622587 | 4.3939890675 | -5.5728620888 |
| H51 | 6.4689859807 | 7.3528350678 | -4.0298960301 |
| H52 | 7.5441712846 | 6.2264949167 | -5.9434644393 |
| C53 | 6.7188891625 | 4.5425618896 | 2.8698177432 |
| H54 | 7.1000091940 | 5.0895781229 | 2.0026940623 |
| C55 | 5.1954797247 | 4.3998379791 | 2.6812230547 |
| H56 | 4.9638524153 | 3.8560529898 | 1.7592387560 |
| H57 | 4.7355214385 | 3.8635350667 | 3.5184532573 |
| H58 | 4.7246350702 | 5.3871958627 | 2.6212062889 |
| C59 | 7.0504566609 | 5.3786101377 | 4.1213632559 |
| H60 | 8.1306903387 | 5.5111201039 | 4.2400083540 |
| H61 | 6.5938528063 | 6.3713826133 | 4.0477987653 |
| H62 | 6.6707404983 | 4.9068961273 | 5.0338655326 |
| C63 | 6.7762086971 | 6.8773764848 | -1.3695686307 |
| H64 | 7.1279413595 | 6.4250538946 | -0.4375968260 |
| C65 | 5.2470529401 | 6.6860655061 | -1.4146985625 |
| H66 | 4.8118325861 | 7.1346945357 | -2.3142212459 |

| | | | |
|---|---|---|---|
| H67 | 4.9817733177 | 5.6238935190 | -1.4036678266 |
| H68 | 4.7785300639 | 7.1645258951 | -0.5475708738 |
| C69 | 7.1516466644 | 8.3718420516 | -1.3258451210 |
| H70 | 6.6942533735 | 8.8550202940 | -0.4558728889 |
| H71 | 8.2356202046 | 8.5115980848 | -1.2595469080 |
| H72 | 6.8027064470 | 8.9006828805 | -2.2192280809 |
| C73 | 10.0220810196 | 3.3261341586 | -3.2263527181 |
| H74 | 10.1138592654 | 3.1352120210 | -2.1531279298 |
| C75 | 9.5388866382 | 2.0151184755 | -3.8770045156 |
| H76 | 8.5694708381 | 1.7087234465 | -3.4705720865 |
| H77 | 9.4341494137 | 2.1178611123 | -4.9623409656 |
| H78 | 10.2578341268 | 1.2099807791 | -3.6894193558 |
| C79 | 11.4190129050 | 3.7254544628 | -3.7429976085 |
| H80 | 12.1409420717 | 2.9246342108 | -3.5501954484 |
| H81 | 11.4106611863 | 3.9104895928 | -4.8224070942 |
| H82 | 11.7836099889 | 4.6344318774 | -3.2533943763 |
| C83 | 9.9913033578 | 1.0563209452 | 0.9400291922 |
| H84 | 10.1395498283 | 1.8779656278 | 0.2331574931 |
| C85 | 9.4607322591 | -0.1445544633 | 0.1319073585 |
| H86 | 9.3020404983 | -1.0191185818 | 0.7723396574 |
| H87 | 8.5102781064 | 0.0975275228 | -0.3551044432 |
| H88 | 10.1795633493 | -0.4283917110 | -0.6446612658 |
| C89 | 11.3623750373 | 0.7437029834 | 1.5700197802 |
| H90 | 12.0867075453 | 0.4802046654 | 0.7919801219 |
| H91 | 11.7556924666 | 1.6039984091 | 2.1211484475 |
| H92 | 11.3050848923 | -0.0994478006 | 2.2664896893 |

**BC-TS**

| | | | |
|---|---|---|---|
| C1 | 3.3801190947 | 0.1007305773 | 1.0324121060 |
| C2 | 3.6153214680 | 0.9891696445 | -0.1757760315 |
| C3 | 2.5676644633 | 0.9816045406 | -1.0241829354 |
| C4 | 1.4654039942 | 0.0159642824 | -0.8651735689 |
| H5 | 4.1024175496 | -0.7269678662 | 1.0671533784 |
| H6 | 3.5245972221 | 0.6644467032 | 1.9614371459 |
| H7 | 2.4588524626 | 1.6602845413 | -1.8676162737 |
| H8 | 1.6147219655 | -1.0231614873 | -1.1774321432 |
| C9 | 1.9761320642 | -0.4292353884 | 1.0173107496 |
| C10 | 1.0290519776 | -0.9845853765 | 1.5989897952 |
| C11 | -0.1232738320 | -1.5793169124 | 2.1459757414 |
| C12 | -0.3387207156 | -2.9729645130 | 2.0170025067 |
| C13 | -1.0753442332 | -0.7831543089 | 2.8279536811 |
| C14 | -1.4853996085 | -3.5466299336 | 2.5475223270 |
| H15 | 0.3984238315 | -3.5797476173 | 1.5006132202 |
| C16 | -2.2171583289 | -1.3714893694 | 3.3538801455 |
| H17 | -0.9004926736 | 0.2831931252 | 2.9285088466 |
| C18 | -2.4235911470 | -2.7484016338 | 3.2123195670 |
| H19 | -1.6540268213 | -4.6141599017 | 2.4465690197 |
| H20 | -2.9493166972 | -0.7626167669 | 3.8747844019 |
| H21 | -3.3199196988 | -3.2028914858 | 3.6242781714 |
| O22 | 0.2537334658 | 0.4923587022 | -1.1205719096 |
| C23 | -0.7952779108 | -0.4421044965 | -1.4396745430 |
| H24 | -1.5662924695 | 0.1294592473 | -1.9558647070 |
| H25 | -0.4194788256 | -1.2366837330 | -2.0922876434 |
| H26 | -1.2012549436 | -0.8683338181 | -0.5193665625 |
| Au27 | 5.3118430827 | 2.1175641080 | -0.3630516761 |

| | | | |
|---|---|---|---|
| C28 | 8.9802708951 | 4.2594746766 | 0.1063234034 |
| C29 | 8.5798647697 | 4.8161013676 | -1.0650614338 |
| H30 | 9.8523949818 | 4.4308576125 | 0.7167044409 |
| H31 | 9.0307093239 | 5.5731980640 | -1.6864040122 |
| C32 | 7.0106486005 | 3.2948151810 | -0.4678070638 |
| N33 | 8.0087341814 | 3.3315800553 | 0.4562619793 |
| N34 | 7.3734373450 | 4.2148319296 | -1.4009102759 |
| C35 | 8.0566309594 | 2.5035352778 | 1.6397022518 |
| C36 | 8.7245583209 | 1.2655626176 | 1.5636444661 |
| C37 | 7.4298655695 | 2.9674613799 | 2.8129275811 |
| C38 | 8.7508974469 | 0.4802699520 | 2.7219750188 |
| C39 | 7.4870615363 | 2.1384436973 | 3.9393776947 |
| C40 | 8.1396546240 | 0.9098336874 | 3.8962733426 |
| H41 | 9.2565327524 | -0.4807230985 | 2.7029092438 |
| H42 | 7.0162053848 | 2.4616068930 | 4.8631866713 |
| H43 | 8.1753935166 | 0.2846034198 | 4.7842008992 |
| C44 | 6.6184639926 | 4.5325405545 | -2.5921107776 |
| C45 | 6.8829751149 | 3.8032465770 | -3.7677529682 |
| C46 | 5.6684319315 | 5.5704692519 | -2.5295403264 |
| C47 | 6.1456534211 | 4.1392974597 | -4.9089592474 |
| C48 | 4.9618389970 | 5.8624216461 | -3.7019959244 |
| C49 | 5.1956353358 | 5.1557892279 | -4.8779001992 |
| H50 | 6.3206942919 | 3.5990113519 | -5.8346540469 |
| H51 | 4.2206394975 | 6.6561689099 | -3.6937825871 |
| H52 | 4.6377276932 | 5.4023133636 | -5.7770301728 |
| C53 | 5.3898984079 | 6.3655816680 | -1.2562780842 |
| H54 | 6.0638928929 | 6.0053125680 | -0.4732572009 |
| C55 | 3.9498124285 | 6.1341840935 | -0.7577654194 |
| H56 | 3.2124025994 | 6.4857763622 | -1.4874772049 |

| | | | |
|---|---|---|---|
| H57 | 3.7628604038 | 5.0711794176 | -0.5730636922 |
| H58 | 3.7775078449 | 6.6793276062 | 0.1768271514 |
| C59 | 5.6792906568 | 7.8668907646 | -1.4508840416 |
| H60 | 5.5323042994 | 8.4080532763 | -0.5101156639 |
| H61 | 6.7089406754 | 8.0370602275 | -1.7817922998 |
| H62 | 5.0127648546 | 8.3138112977 | -2.1960198197 |
| C63 | 7.9192420804 | 2.6840222638 | -3.8339603194 |
| H64 | 8.4020668947 | 2.6058213955 | -2.8552935439 |
| C65 | 7.2531246112 | 1.3240797704 | -4.1210342278 |
| H66 | 6.5023567199 | 1.0850795326 | -3.3605817370 |
| H67 | 6.7583581786 | 1.3196340545 | -5.0982782142 |
| H68 | 8.0028775385 | 0.5252585627 | -4.1249134358 |
| C69 | 9.0241706945 | 2.9938702644 | -4.8627262441 |
| H70 | 9.7868196305 | 2.2079565493 | -4.8500895459 |
| H71 | 8.6250345628 | 3.0494972093 | -5.8809208611 |
| H72 | 9.5172477601 | 3.9471638535 | -4.6464414786 |
| C73 | 9.3892900289 | 0.7592088588 | 0.2854624832 |
| H74 | 9.2931401443 | 1.5342103034 | -0.4807539467 |
| C75 | 8.6780543392 | -0.4991086404 | -0.2511122670 |
| H76 | 8.7457224229 | -1.3302977333 | 0.4593106362 |
| H77 | 7.6179036453 | -0.3015268574 | -0.4420678985 |
| H78 | 9.1385561036 | -0.8262967758 | -1.1898262706 |
| C79 | 10.8955396093 | 0.5054352262 | 0.4885071997 |
| H80 | 11.3606483827 | 0.2036234944 | -0.4558958466 |
| H81 | 11.4084299514 | 1.4040650071 | 0.8461507068 |
| H82 | 11.0776161799 | -0.2930327570 | 1.2154006870 |
| C83 | 6.7012081365 | 4.3072210154 | 2.8901430012 |
| H84 | 6.8077727827 | 4.8103335449 | 1.9244910188 |
| C85 | 5.1915527559 | 4.1097568572 | 3.1341057422 |

| | | | |
|---|---|---|---|
| H86 | 4.7426125825 | 3.5002324432 | 2.3425458506 |
| H87 | 5.0031169628 | 3.6160680100 | 4.0940302130 |
| H88 | 4.6779620657 | 5.0772384517 | 3.1513715311 |
| C89 | 7.3217226627 | 5.2309599854 | 3.9559981659 |
| H90 | 8.3884027186 | 5.3944227187 | 3.7720081688 |
| H91 | 6.8236549881 | 6.2062605289 | 3.9497124260 |
| H92 | 7.2178772934 | 4.8141087506 | 4.9633206436 |

**C**

| | | | |
|---|---|---|---|
| C1 | 2.5746975099 | 0.2475397579 | -0.2240175585 |
| C2 | 2.8090551353 | 1.6974782973 | 0.1217531648 |
| C3 | 1.8372323445 | 2.6187159171 | 0.5254858684 |
| C4 | 0.4798665623 | 2.3532162443 | 0.6742660392 |
| H5 | 3.0067354354 | 0.1194491148 | -1.2301918241 |
| H6 | 3.2788748168 | -0.3160058653 | 0.4098513524 |
| H7 | 2.1778663561 | 3.6272472234 | 0.7381544082 |
| H8 | 0.0635124865 | 1.3701106032 | 0.4792731274 |
| C9 | 1.2662047444 | -0.4024156154 | -0.1784082486 |
| C10 | 0.2309630561 | -1.0348459999 | -0.1731162051 |
| C11 | -1.0019501261 | -1.7591855291 | -0.1589267809 |
| C12 | -1.8397272066 | -1.7592501672 | -1.2902608115 |
| C13 | -1.3888559049 | -2.4817939818 | 0.9855146595 |
| C14 | -3.0384408276 | -2.4664679306 | -1.2717931479 |
| H15 | -1.5403459801 | -1.2071569710 | -2.1758402253 |
| C16 | -2.5892208310 | -3.1861025214 | 0.9939931909 |
| H17 | -0.7416092330 | -2.4870930036 | 1.8570658812 |
| C18 | -3.4162506231 | -3.1794199337 | -0.1316313765 |

| | | | |
|---|---|---|---|
| H19 | -3.6788830855 | -2.4631542441 | -2.1489380132 |
| H20 | -2.8801792286 | -3.7428292649 | 1.8799077218 |
| H21 | -4.3520020085 | -3.7308322447 | -0.1211656177 |
| O22 | -0.4351223979 | 3.2034458170 | 1.0487095434 |
| C23 | -0.0969927035 | 4.5772870412 | 1.3607242523 |
| H24 | -1.0398412585 | 5.0450466470 | 1.6377162080 |
| H25 | 0.6049061568 | 4.6092181191 | 2.1975352031 |
| H26 | 0.3278314834 | 5.0672147340 | 0.4812409658 |
| Au27 | 4.7587034656 | 2.3056891129 | -0.0379673961 |
| C28 | 8.9598586816 | 2.8203785395 | -0.5802117839 |
| C29 | 8.6400435058 | 4.0886893509 | -0.2110142137 |
| H30 | 9.9044253881 | 2.3718436731 | -0.8439190981 |
| H31 | 9.2493341659 | 4.9696755961 | -0.0876358448 |
| C32 | 6.7313474212 | 2.8789102151 | -0.2102845502 |
| N33 | 7.7785383933 | 2.0933309996 | -0.5744559321 |
| N34 | 7.2703355898 | 4.1061184367 | 0.0111304696 |
| C35 | 7.6708822698 | 0.6906392172 | -0.9209997268 |
| C36 | 7.4214956603 | 0.3486676091 | -2.2649782554 |
| C37 | 7.8192146375 | -0.2669572431 | 0.1015873980 |
| C38 | 7.3098009779 | -1.0134842902 | -2.5671748850 |
| C39 | 7.6993328536 | -1.6134544436 | -0.2615687959 |
| C40 | 7.4463066377 | -1.9839692407 | -1.5789762980 |
| H41 | 7.1173440604 | -1.3182589821 | -3.5915905629 |
| H42 | 7.8078421078 | -2.3819559700 | 0.4981057214 |
| H43 | 7.3591313444 | -3.0355453723 | -1.8378612315 |
| C44 | 6.5175356498 | 5.2740704392 | 0.4195399017 |
| C45 | 5.9801103780 | 6.1096305588 | -0.5798717844 |
| C46 | 6.3637114272 | 5.5240292479 | 1.7975887158 |
| C47 | 5.2610211127 | 7.2338056385 | -0.1546210130 |

| | | | |
|---|---|---|---|
| C48 | 5.6354275600 | 6.6629966815 | 2.1638178863 |
| C49 | 5.0911636450 | 7.5077051009 | 1.2000477126 |
| H50 | 4.8342787021 | 7.9043255863 | -0.8946200228 |
| H51 | 5.4983443614 | 6.8921663739 | 3.2163879718 |
| H52 | 4.5361218850 | 8.3899718355 | 1.5069765587 |
| C53 | 6.9466615715 | 4.6132137444 | 2.8755436325 |
| H54 | 7.4781765605 | 3.7956511674 | 2.3803957962 |
| C55 | 5.8357609276 | 3.9752691329 | 3.7330542692 |
| H56 | 5.2656788556 | 4.7331754587 | 4.2811662243 |
| H57 | 5.1348976959 | 3.4064804969 | 3.1122970270 |
| H58 | 6.2695593658 | 3.2905592908 | 4.4697931779 |
| C59 | 7.9723870890 | 5.3575404501 | 3.7522883428 |
| H60 | 8.4198496024 | 4.6717869362 | 4.4794180727 |
| H61 | 8.7798177830 | 5.7841497810 | 3.1485717519 |
| H62 | 7.5072920338 | 6.1756413887 | 4.3121090865 |
| C63 | 6.1480085411 | 5.8322646571 | -2.0721102565 |
| H64 | 6.7492223666 | 4.9256955332 | -2.1849386932 |
| C65 | 4.7895129326 | 5.5603626710 | -2.7483133410 |
| H66 | 4.2746174673 | 4.7169879102 | -2.2754201877 |
| H67 | 4.1310852093 | 6.4339896691 | -2.6922076258 |
| H68 | 4.9320628724 | 5.3206286594 | -3.8075776241 |
| C69 | 6.9063549178 | 6.9717710081 | -2.7799768550 |
| H70 | 7.0648954296 | 6.7246472760 | -3.8349500160 |
| H71 | 6.3489555385 | 7.9134801234 | -2.7415275284 |
| H72 | 7.8855290365 | 7.1456247974 | -2.3225215968 |
| C73 | 7.2710581636 | 1.3853600939 | -3.3758875805 |
| H74 | 7.4227789737 | 2.3786499804 | -2.9425538918 |
| C75 | 8.3391948616 | 1.1984177622 | -4.4716020224 |
| H76 | 8.2262676799 | 0.2384509551 | -4.9861051671 |

| | | | |
|---|---|---|---|
| H77 | 8.2535079347 | 1.9880791451 | -5.2253261655 |
| H78 | 9.3512021445 | 1.2355150673 | -4.0559491294 |
| C79 | 5.8509785093 | 1.3617921940 | -3.9751689994 |
| H80 | 5.6286821326 | 0.3957454746 | -4.4409387244 |
| H81 | 5.0940656874 | 1.5482282648 | -3.2055695081 |
| H82 | 5.7488387941 | 2.1329623178 | -4.7463244457 |
| C83 | 8.0966645208 | 0.1035578104 | 1.5562761691 |
| H84 | 8.1826249977 | 1.1921121914 | 1.6216273330 |
| C85 | 9.4320610868 | -0.4900573501 | 2.0461786078 |
| H86 | 9.6416878359 | -0.1625315218 | 3.0698241674 |
| H87 | 9.4108778252 | -1.5848775182 | 2.0482396389 |
| H88 | 10.2670556350 | -0.1752680058 | 1.4121780356 |
| C89 | 6.9298334811 | -0.3151387985 | 2.4727519287 |
| H90 | 5.9901923617 | 0.1486775269 | 2.1533725105 |
| H91 | 6.7882659386 | -1.4012665590 | 2.4710000600 |
| H92 | 7.1266629713 | -0.0086054375 | 3.5057255355 |

**CC'-TS**

| | | | |
|---|---|---|---|
| C1 | 1.9687074814 | -0.3108368697 | 0.7939088149 |
| C2 | 2.9494829315 | 0.8436819733 | 1.0393853997 |
| C3 | 2.8012452704 | 1.7067331086 | 2.1828325802 |
| C4 | 1.7604288540 | 1.6403174407 | 3.0603502334 |
| H5 | 2.2494869441 | -0.8116211969 | -0.1384367197 |
| H6 | 2.2008405256 | -1.0409258275 | 1.5862666448 |
| H7 | 3.6008997263 | 2.4178174235 | 2.3611286734 |
| H8 | 0.9313722941 | 0.9541195166 | 2.9214597759 |
| C9 | 0.5330630613 | -0.0364468469 | 0.7484782579 |

| | | | |
|---|---|---|---|
| C10 | -0.6539237046 | 0.1684491078 | 0.6123449213 |
| C11 | -2.0492003005 | 0.4164044313 | 0.4176839322 |
| C12 | -2.6530378640 | 0.0962757533 | -0.8134886862 |
| C13 | -2.8347442590 | 0.9871570718 | 1.4362039622 |
| C14 | -4.0072088539 | 0.3442404780 | -1.0176430332 |
| H15 | -2.0505989082 | -0.3484555679 | -1.5997586936 |
| C16 | -4.1887191991 | 1.2323863488 | 1.2231986449 |
| H17 | -2.3764949751 | 1.2331127884 | 2.3893240179 |
| C18 | -4.7782066992 | 0.9132065758 | -0.0017658302 |
| H19 | -4.4630457283 | 0.0915021168 | -1.9707306860 |
| H20 | -4.7868949389 | 1.6720427080 | 2.0160586687 |
| H21 | -5.8350234071 | 1.1048098300 | -0.1634560046 |
| O22 | 1.6055092145 | 2.3379262564 | 4.1792960160 |
| C23 | 2.5817017984 | 3.3313554147 | 4.5235177855 |
| H24 | 2.2331312280 | 3.7781520045 | 5.4539935636 |
| H25 | 3.5632319218 | 2.8702481874 | 4.6746203465 |
| H26 | 2.6463067323 | 4.0958158885 | 3.7424215287 |
| Au27 | 4.8714310059 | 0.4646275569 | 0.3397431997 |
| C28 | 8.5537405415 | -1.2853337000 | -0.9364149992 |
| C29 | 9.0039890158 | -0.0309864668 | -0.6782455294 |
| H30 | 9.0662371307 | -2.1720938547 | -1.2734861632 |
| H31 | 9.9895430082 | 0.4013881889 | -0.7446224730 |
| C32 | 6.7782469554 | -0.0515571378 | -0.2647468593 |
| N33 | 7.1902086693 | -1.2800046445 | -0.6783480852 |
| N34 | 7.9039140132 | 0.7103854745 | -0.2680959733 |
| N35 | 2.2369803290 | 2.0756283094 | -0.4463072349 |
| C36 | 2.3421585785 | 2.1614685534 | -1.7525770300 |
| C37 | 1.5421596520 | 3.2211218969 | -2.2516204725 |
| H38 | 2.9822570295 | 1.4688218890 | -2.2822484255 |

| | | | |
|---|---|---|---|
| C39 | 0.9691609401 | 3.7423433602 | -1.1320199394 |
| H40 | 1.4195153219 | 3.5385578504 | -3.2753287224 |
| H41 | 0.2831150771 | 4.5570396255 | -0.9516440049 |
| O42 | 1.3740825403 | 3.0709202516 | -0.0390326966 |
| C43 | 7.9613144049 | 2.1053790398 | 0.1070629012 |
| C44 | 8.1226691596 | 2.4308182095 | 1.4682530530 |
| C45 | 7.8722902038 | 3.0766068660 | -0.9095392577 |
| C46 | 8.1863384619 | 3.7899917243 | 1.7965445137 |
| C47 | 7.9470215712 | 4.4190985120 | -0.5213938251 |
| C48 | 8.1007560655 | 4.7733573858 | 0.8155912213 |
| H49 | 8.3133614659 | 4.0814856867 | 2.8350013003 |
| H50 | 7.8851421760 | 5.1968560510 | -1.2769139100 |
| H51 | 8.1593716755 | 5.8218776977 | 1.0939382525 |
| C52 | 6.3312314358 | -2.4341739384 | -0.8266305744 |
| C53 | 6.1105673324 | -3.2590210934 | 0.2941786878 |
| C54 | 5.7627120187 | -2.6880494759 | -2.0904328074 |
| C55 | 5.2736385161 | -4.3673387226 | 0.1167957075 |
| C56 | 4.9388740806 | -3.8132554207 | -2.2084929460 |
| C57 | 4.6937694222 | -4.6425824190 | -1.1179412093 |
| H58 | 5.0776201678 | -5.0266368724 | 0.9572461467 |
| H59 | 4.4842576627 | -4.0436810805 | -3.1675967559 |
| H60 | 4.0510563532 | -5.5109705044 | -1.2324431735 |
| C61 | 7.6948636586 | 2.7210116409 | -2.3837786668 |
| H62 | 7.6505265806 | 1.6315273224 | -2.4698939876 |
| C63 | 6.3656054107 | 3.2725500520 | -2.9348630952 |
| H64 | 6.2236329085 | 2.9564014161 | -3.9742246112 |
| H65 | 5.5164039381 | 2.9116473742 | -2.3448877109 |
| H66 | 6.3457017955 | 4.3673497607 | -2.9168925912 |
| C67 | 8.8897944443 | 3.1997509058 | -3.2317219645 |

| | | | |
|---|---|---|---|
| H68 | 9.8338033080 | 2.7837984702 | -2.8651475134 |
| H69 | 8.7665034577 | 2.8901315422 | -4.2750415720 |
| H70 | 8.9810594787 | 4.2909998774 | -3.2190314094 |
| C71 | 6.0087665920 | -1.7967236783 | -3.3057194553 |
| H72 | 6.6564447832 | -0.9699035314 | -2.9992305569 |
| C73 | 4.6954932843 | -1.1747238209 | -3.8191714998 |
| H74 | 4.0015906007 | -1.9380813433 | -4.1868541296 |
| H75 | 4.1905586992 | -0.6203675636 | -3.0200559704 |
| H76 | 4.8964296493 | -0.4845032883 | -4.6459123198 |
| C77 | 6.7383638612 | -2.5602040016 | -4.4289533865 |
| H78 | 6.9468202460 | -1.8935951535 | -5.2726065051 |
| H79 | 7.6909725265 | -2.9714563223 | -4.0798976540 |
| H80 | 6.1359407276 | -3.3933223706 | -4.8061158409 |
| C81 | 6.7423927290 | -2.9982256851 | 1.6600183204 |
| H82 | 7.3578210539 | -2.0965176325 | 1.5866449844 |
| C83 | 5.6715379648 | -2.7314509472 | 2.7363522991 |
| H84 | 5.0434156298 | -1.8765153966 | 2.4638342300 |
| H85 | 5.0191848615 | -3.5996953893 | 2.8789875971 |
| H86 | 6.1454464379 | -2.5122855988 | 3.6994642811 |
| C87 | 7.6733430701 | -4.1539159853 | 2.0767857788 |
| H88 | 8.1638471204 | -3.9232985133 | 3.0283374314 |
| H89 | 7.1196525725 | -5.0893988188 | 2.2085143320 |
| H90 | 8.4523728169 | -4.3308538628 | 1.3283432563 |
| C91 | 8.2419387878 | 1.3785973788 | 2.5683186873 |
| H92 | 8.1588417551 | 0.3897603220 | 2.1078333495 |
| C93 | 7.0956981054 | 1.4982774009 | 3.5921755790 |
| H94 | 7.1198942412 | 2.4628403222 | 4.1113047071 |
| H95 | 6.1203212540 | 1.3974951643 | 3.1040209919 |
| H96 | 7.1794001720 | 0.7122636625 | 4.3505321567 |

| | | | |
|---|---|---|---|
| C97 | 9.6168763240 | 1.4468460843 | 3.2621458189 |
| H98 | 9.7073817341 | 0.6507922115 | 4.0087829964 |
| H99 | 10.4340443057 | 1.3308816786 | 2.5430429359 |
| H100 | 9.7605497697 | 2.4021841519 | 3.7778535809 |

**C’**

| | | | |
|---|---|---|---|
| C1 | 1.9102217775 | -0.0152032257 | 0.6837285986 |
| C2 | 2.8023493780 | 1.2507523727 | 0.4891833689 |
| C3 | 2.8653566814 | 2.1981262044 | 1.6535199162 |
| C4 | 2.2507959954 | 2.0129775751 | 2.8347234673 |
| H5 | 2.0992311562 | -0.7005173926 | -0.1505724820 |
| H6 | 2.2935478013 | -0.5231522744 | 1.5771153512 |
| H7 | 3.5123368913 | 3.0583256374 | 1.5082044683 |
| H8 | 1.5877256299 | 1.1760200502 | 3.0267054795 |
| C9 | 0.4666699922 | 0.1935862035 | 0.7941621934 |
| C10 | -0.7390638708 | 0.3151702335 | 0.8212203594 |
| C11 | -2.1629316422 | 0.4486852569 | 0.8449799977 |
| C12 | -2.9416285449 | -0.0934410769 | -0.1960025961 |
| C13 | -2.8075776359 | 1.1159961294 | 1.9035967926 |
| C14 | -4.3277056974 | 0.0305120587 | -0.1750667476 |
| H15 | -2.4494590845 | -0.6152438430 | -1.0108739798 |
| C16 | -4.1942509133 | 1.2357657559 | 1.9163354278 |
| H17 | -2.2126832515 | 1.5311907719 | 2.7106089302 |
| C18 | -4.9575087232 | 0.6948888226 | 0.8795790888 |
| H19 | -4.9183954324 | -0.3950367935 | -0.9812381312 |
| H20 | -4.6817928361 | 1.7498637438 | 2.7394145233 |
| H21 | -6.0395327727 | 0.7880289861 | 0.8947817721 |

| | | | |
|---|---|---|---|
| O22 | 2.3500580085 | 2.7805652633 | 3.9418411393 |
| C23 | 3.1652917648 | 3.9447543225 | 3.8725883109 |
| H24 | 3.1121176748 | 4.4115353832 | 4.8568787375 |
| H25 | 4.2066122204 | 3.6857369133 | 3.6421053269 |
| H26 | 2.7919181880 | 4.6450257151 | 3.1145523498 |
| Au27 | 4.8012948840 | 0.5741346202 | 0.0843101929 |
| C28 | 8.5369411452 | -1.4392699391 | -0.5107907330 |
| C29 | 8.9782079366 | -0.3138018570 | 0.1051244518 |
| H30 | 9.0626122588 | -2.3097607226 | -0.8692902476 |
| H31 | 9.9680954819 | 0.0010205551 | 0.3941439174 |
| C32 | 6.7303681000 | -0.1348086720 | -0.1068473530 |
| N33 | 7.1593824349 | -1.3140226918 | -0.6339013965 |
| N34 | 7.8603857808 | 0.4734018729 | 0.3438618373 |
| N35 | 2.2710517789 | 1.9998030716 | -0.6763333309 |
| C36 | 2.5268363317 | 1.9776354524 | -1.9734976636 |
| C37 | 1.5947617644 | 2.7827132940 | -2.6564352233 |
| H38 | 3.3654200582 | 1.4029330929 | -2.3384432805 |
| C39 | 0.7895856432 | 3.2783089775 | -1.6701936256 |
| H40 | 1.5481900636 | 2.9776671657 | -3.7163754885 |
| H41 | -0.0500926962 | 3.9594621287 | -1.6633876136 |
| O42 | 1.1615466975 | 2.8194893596 | -0.4704338309 |
| C43 | 7.9105720549 | 1.7604786238 | 1.0019118292 |
| C44 | 8.1257586898 | 2.9074872085 | 0.2128436128 |
| C45 | 7.7711652327 | 1.8030999226 | 2.4028921945 |
| C46 | 8.1914195081 | 4.1372532181 | 0.8772588819 |
| C47 | 7.8490740580 | 3.0610726499 | 3.0124312371 |
| C48 | 8.0560612316 | 4.2141084676 | 2.2605304021 |
| H49 | 8.3575777832 | 5.0452341734 | 0.3048502894 |
| H50 | 7.7540182858 | 3.1365763851 | 4.0917421227 |

| | | | |
|---|---|---|---|
| H51 | 8.1191299942 | 5.1786458580 | 2.7568170961 |
| C52 | 6.3172250263 | -2.3355044853 | -1.2174313479 |
| C53 | 5.7836780869 | -3.3273302330 | -0.3710541427 |
| C54 | 6.1051231544 | -2.3182271340 | -2.6099363794 |
| C55 | 4.9892133765 | -4.3145762055 | -0.9658864812 |
| C56 | 5.3055339664 | -3.3325848884 | -3.1485586630 |
| C57 | 4.7495434156 | -4.3168335908 | -2.3366780939 |
| H58 | 4.5594063584 | -5.0968866917 | -0.3472347295 |
| H59 | 5.1222621988 | -3.3565873240 | -4.2189062565 |
| H60 | 4.1345515746 | -5.0967996461 | -2.7768338629 |
| C61 | 8.2836183683 | 2.8512144634 | -1.3050764462 |
| H62 | 8.2377735194 | 1.8030697897 | -1.6157475390 |
| C63 | 7.1272563409 | 3.5838721759 | -2.0130997649 |
| H64 | 7.2260667562 | 3.4933996767 | -3.1003905627 |
| H65 | 6.1592588167 | 3.1638112177 | -1.7186566399 |
| H66 | 7.1197575981 | 4.6514710856 | -1.7687771516 |
| C67 | 9.6503305124 | 3.4010897338 | -1.7568031729 |
| H68 | 10.4754983992 | 2.8653761583 | -1.2769694334 |
| H69 | 9.7638808328 | 3.2953896448 | -2.8408307850 |
| H70 | 9.7593097632 | 4.4635968075 | -1.5155854449 |
| C71 | 6.7194822561 | -1.2665046487 | -3.5302808957 |
| H72 | 7.2730435585 | -0.5534539283 | -2.9127616164 |
| C73 | 5.6360099004 | -0.4684108269 | -4.2797978170 |
| H74 | 5.0516368232 | -1.1067310205 | -4.9507474174 |
| H75 | 4.9416453618 | 0.0002211958 | -3.5732336732 |
| H76 | 6.0920154066 | 0.3210779799 | -4.8867923195 |
| C77 | 7.7232593775 | -1.8982738724 | -4.5157790224 |
| H78 | 8.1891838793 | -1.1251451191 | -5.1361033178 |
| H79 | 8.5195661736 | -2.4331196390 | -3.9881444265 |

| | | | |
|---|---|---|---|
| H80 | 7.2328794602 | -2.6118567704 | -5.1864200648 |
| C81 | 6.0552224903 | -3.3750235929 | 1.1308178910 |
| H82 | 6.6842580107 | -2.5192473619 | 1.3930346927 |
| C83 | 6.8340074650 | -4.6486565358 | 1.5163733359 |
| H84 | 7.0705748337 | -4.6416206722 | 2.5855978973 |
| H85 | 6.2521818816 | -5.5535907282 | 1.3119835706 |
| H86 | 7.7751005794 | -4.7261944830 | 0.9622589646 |
| C87 | 4.7549909431 | -3.2487023526 | 1.9478902626 |
| H88 | 4.2276354899 | -2.3197138047 | 1.7072822674 |
| H89 | 4.0729809799 | -4.0835540113 | 1.7551455898 |
| H90 | 4.9784536428 | -3.2453031210 | 3.0201383130 |
| C91 | 7.5572887970 | 0.5554406621 | 3.2569183572 |
| H92 | 7.5034414020 | -0.3102006855 | 2.5900098848 |
| C93 | 6.2233226957 | 0.6180959167 | 4.0269111908 |
| H94 | 6.2134635380 | 1.4419325462 | 4.7487775562 |
| H95 | 5.3761904483 | 0.7554174573 | 3.3471140908 |
| H96 | 6.0645297835 | -0.3096720306 | 4.5873934061 |
| C97 | 8.7428042803 | 0.3217062180 | 4.2143948028 |
| H98 | 8.5985131145 | -0.6045274516 | 4.7806578945 |
| H99 | 9.6893431331 | 0.2412726444 | 3.6700409672 |
| H100 | 8.8429653670 | 1.1386139858 | 4.9368349317 |

**C’’**

| | | | |
|---|---|---|---|
| C1 | 2.4490594681 | -0.9444942378 | 0.1695682375 |
| C2 | 3.4081926840 | -1.9912651120 | 0.8044054272 |
| C3 | 3.2727057224 | -3.3963895477 | 0.2968425051 |
| C4 | 2.4917561746 | -3.7821983525 | -0.7283553037 |

| | | | |
|---|---|---|---|
| H5 | 2.7499853409 | 0.0633291795 | 0.4748091601 |
| H6 | 2.5949154681 | -0.9873680971 | -0.9123355997 |
| H7 | 3.9316885436 | -4.1161912343 | 0.7730148945 |
| H8 | 1.8217158763 | -3.1042808218 | -1.2487796493 |
| C9 | 1.0124110241 | -1.0905322225 | 0.5128761534 |
| C10 | 0.0123836252 | -1.3315460221 | 1.2050004434 |
| C11 | -1.0472453823 | -1.6695062088 | 2.1065516967 |
| C12 | -1.5746378020 | -0.6935029449 | 2.9759376593 |
| C13 | -1.5392921838 | -2.9899176356 | 2.1551754196 |
| C14 | -2.5749524180 | -1.0385927692 | 3.8785855621 |
| H15 | -1.1992470151 | 0.3238184124 | 2.9316250885 |
| C16 | -2.5397075099 | -3.3219889815 | 3.0636988964 |
| H17 | -1.1331424502 | -3.7379336769 | 1.4823240027 |
| C18 | -3.0588532982 | -2.3489861516 | 3.9220627208 |
| H19 | -2.9819994289 | -0.2850214587 | 4.5459206425 |
| H20 | -2.9192998250 | -4.3385921782 | 3.0995756903 |
| H21 | -3.8434605879 | -2.6120348057 | 4.6254150306 |
| O22 | 2.4170747522 | -5.0052099982 | -1.2905350927 |
| C23 | 3.2449253464 | -6.0371372857 | -0.7578027208 |
| H24 | 3.0417751634 | -6.9271341789 | -1.3539679817 |
| H25 | 4.3058858264 | -5.7707094963 | -0.8410380625 |
| H26 | 3.0009537955 | -6.2362304792 | 0.2931579763 |
| Au27 | 5.4041079288 | -1.2745931756 | 0.4202548748 |
| C28 | 9.1265816838 | 0.7581997504 | 0.2407114781 |
| C29 | 9.3177741874 | -0.0823365823 | -0.8088769993 |
| H30 | 9.7583433605 | 1.5208188529 | 0.6677600485 |
| H31 | 10.1521381130 | -0.2032515374 | -1.4815401424 |
| C32 | 7.2713444093 | -0.5200280300 | 0.0405757675 |
| N33 | 7.8662934929 | 0.4741217667 | 0.7514148088 |

| | | | |
|---|---|---|---|
| N34 | 8.1708878040 | -0.8574858494 | -0.9187929227 |
| N35 | 3.1884049413 | -2.0006119267 | 2.2734091331 |
| C36 | 3.7461619447 | -1.3671249217 | 3.2935549971 |
| C37 | 3.0809599138 | -1.7097374401 | 4.4869430514 |
| H38 | 4.5936746695 | -0.7162305607 | 3.1290762175 |
| C39 | 2.1166380720 | -2.5948695241 | 4.0986845776 |
| H40 | 3.3096491666 | -1.3670080755 | 5.4842303372 |
| H41 | 1.3711059479 | -3.1695173506 | 4.6310069711 |
| O42 | 2.1432348952 | -2.7856758799 | 2.7728530819 |
| Au43 | -0.5739551678 | -0.3372276942 | -0.9030986724 |
| C44 | -1.6130997701 | 0.3799848520 | -2.4889555917 |
| N45 | -2.1464691142 | 1.6147512449 | -2.6691331575 |
| N46 | -1.9142429559 | -0.3155603314 | -3.6173109525 |
| C47 | -2.7792662657 | 1.6916609933 | -3.8994459834 |
| C48 | -2.6341627357 | 0.4785653940 | -4.4949561193 |
| H49 | -3.2671382922 | 2.5947146552 | -4.2303017857 |
| H50 | -2.9718848904 | 0.1089653905 | -5.4502342133 |
| C51 | -1.5590917643 | -1.6988567793 | -3.8697789507 |
| C52 | -0.3560320806 | -1.9728061416 | -4.5519802867 |
| C53 | -2.4472741737 | -2.7010678242 | -3.4314658847 |
| C54 | -0.0379656453 | -3.3194037333 | -4.7643364066 |
| C55 | -2.0761793940 | -4.0304085112 | -3.6736000658 |
| C56 | -0.8848717609 | -4.3350532477 | -4.3275017014 |
| H57 | 0.8792104662 | -3.5755782899 | -5.2865715815 |
| H58 | -2.7324434440 | -4.8343907597 | -3.3542055233 |
| H59 | -0.6181124084 | -5.3727312000 | -4.5070762787 |
| C60 | -2.0735350735 | 2.7191166586 | -1.7322246209 |
| C61 | -3.1146328949 | 2.8723017419 | -0.7969027503 |
| C62 | -0.9898233177 | 3.6130283458 | -1.8361059560 |

| | | | |
|---|---|---|---|
| C63 | -3.0359915489 | 3.9664437513 | 0.0743263916 |
| C64 | -0.9645911891 | 4.6866862133 | -0.9382743672 |
| C65 | -1.9731222045 | 4.8622800879 | 0.0053513184 |
| H66 | -3.8237417026 | 4.1250840648 | 0.8044935257 |
| H67 | -0.1507053860 | 5.4031853596 | -0.9857852940 |
| H68 | -1.9356516591 | 5.7101473971 | 0.6837029165 |
| C69 | 7.9702720575 | -1.8767502272 | -1.9301458207 |
| C70 | 8.2523846087 | -3.2177246780 | -1.6037573298 |
| C71 | 7.5201487654 | -1.4696090602 | -3.2002007363 |
| C72 | 8.0315885898 | -4.1779832387 | -2.5983361950 |
| C73 | 7.3234458691 | -2.4730756041 | -4.1560906315 |
| C74 | 7.5681121862 | -3.8114668298 | -3.8584443908 |
| H75 | 8.2397492912 | -5.2222983757 | -2.3883754473 |
| H76 | 6.9794809304 | -2.2026185919 | -5.1502099433 |
| H77 | 7.4106020237 | -4.5721667259 | -4.6182367937 |
| C78 | 7.2563700925 | 1.1528570662 | 1.8741010027 |
| C79 | 7.5274592748 | 0.6719488634 | 3.1732501465 |
| C80 | 6.4373005447 | 2.2723789405 | 1.6224737033 |
| C81 | 6.9263014371 | 1.3478224213 | 4.2447542834 |
| C82 | 5.8635903064 | 2.9083621977 | 2.7319141739 |
| C83 | 6.1073797598 | 2.4541885156 | 4.0262369543 |
| H84 | 7.1225592803 | 1.0192191325 | 5.2612869241 |
| H85 | 5.2342395409 | 3.7807187212 | 2.5812964071 |
| H86 | 5.6670783212 | 2.9754675441 | 4.8721523366 |
| C87 | -4.3139927105 | 1.9323455591 | -0.7332434706 |
| H88 | -4.1582158825 | 1.1285271171 | -1.4589168870 |
| C89 | -5.6115231752 | 2.6659898838 | -1.1283626915 |
| H90 | -6.4587642420 | 1.9727051967 | -1.1268977833 |
| H91 | -5.8420296670 | 3.4753869094 | -0.4279177142 |

| | | | |
|---|---|---|---|
| H92 | -5.5366295631 | 3.1056797156 | -2.1283142517 |
| C93 | -4.4425005140 | 1.2671075646 | 0.6490246105 |
| H94 | -3.5324846065 | 0.7168263558 | 0.9104167687 |
| H95 | -4.6298623751 | 2.0030929809 | 1.4381500625 |
| H96 | -5.2800663528 | 0.5616008849 | 0.6539995997 |
| C97 | 0.1194516534 | 3.4555618678 | -2.8745951854 |
| H98 | -0.1001008125 | 2.5713910363 | -3.4801476097 |
| C99 | 1.4866385565 | 3.2146858914 | -2.2054856016 |
| H100 | 1.4603387824 | 2.3295437124 | -1.5601765208 |
| H101 | 2.2609683889 | 3.0607546798 | -2.9650543190 |
| H102 | 1.7901704814 | 4.0696092578 | -1.5916230077 |
| C103 | 0.1749306939 | 4.6597757588 | -3.8333987607 |
| H104 | 0.4198259741 | 5.5846311169 | -3.3021570498 |
| H105 | 0.9421372986 | 4.5035592668 | -4.5989849858 |
| H106 | -0.7824591534 | 4.8120433765 | -4.3411694806 |
| C107 | -3.7667424459 | -2.3878919790 | -2.7281143791 |
| H108 | -3.8704425316 | -1.3004294569 | -2.6595164267 |
| C109 | -4.9725400481 | -2.9088818523 | -3.5348476759 |
| H110 | -4.9656043312 | -4.0009500312 | -3.6127687472 |
| H111 | -5.9094525658 | -2.6181940291 | -3.0484996876 |
| H112 | -4.9798336933 | -2.5031206704 | -4.5514010669 |
| C113 | -3.7813368138 | -2.9338510235 | -1.2872265850 |
| H114 | -2.9521519193 | -2.5273924294 | -0.6978703055 |
| H115 | -4.7174776879 | -2.6629301468 | -0.7873638278 |
| H116 | -3.7021619303 | -4.0261668601 | -1.2718928290 |
| C117 | 0.5806821704 | -0.8809344250 | -5.0647691709 |
| H118 | 0.1295975903 | 0.0905429813 | -4.8423733146 |
| C119 | 0.7601878743 | -0.9579002658 | -6.5938308843 |
| H120 | -0.2017692274 | -0.9014137209 | -7.1127906185 |

| | | | |
|---|---|---|---|
| H121 | 1.3825749700 | -0.1286001612 | -6.9465303556 |
| H122 | 1.2479573106 | -1.8893838297 | -6.8994073259 |
| C123 | 1.9421852089 | -0.9249240885 | -4.3456011430 |
| H124 | 1.8163552172 | -0.8089109737 | -3.2629705106 |
| H125 | 2.4631654946 | -1.8713350143 | -4.5265567714 |
| H126 | 2.5885643547 | -0.1150433414 | -4.6995149244 |
| C127 | 6.1756155458 | 2.8073924451 | 0.2159233551 |
| H128 | 6.6985302483 | 2.1626184153 | -0.4958484755 |
| C129 | 4.6770714227 | 2.7521936987 | -0.1415658351 |
| H130 | 4.2945604744 | 1.7279607038 | -0.0686440312 |
| H131 | 4.0788259012 | 3.3872633671 | 0.5211566695 |
| H132 | 4.5173639463 | 3.1008464541 | -1.1676131189 |
| C133 | 6.7393919652 | 4.2316872542 | 0.0423060355 |
| H134 | 7.8110059973 | 4.2655392908 | 0.2605114157 |
| H135 | 6.5947869014 | 4.5764505542 | -0.9869444038 |
| H136 | 6.2422999046 | 4.9478626623 | 0.7049029091 |
| C137 | 7.2557974842 | -0.0080614013 | -3.5565593346 |
| H138 | 7.4769737424 | 0.6050540279 | -2.6776515908 |
| C139 | 5.7743561420 | 0.2245970649 | -3.9112129049 |
| H140 | 5.4819322630 | -0.3399570312 | -4.8030443117 |
| H141 | 5.1192441960 | -0.0822963924 | -3.0885644433 |
| H142 | 5.5940903095 | 1.2857153545 | -4.1176913173 |
| C143 | 8.1812477275 | 0.4732983242 | -4.6916765415 |
| H144 | 8.0186500516 | 1.5377581063 | -4.8918057116 |
| H145 | 9.2365816174 | 0.3358613031 | -4.4352830662 |
| H146 | 7.9941485224 | -0.0706029546 | -5.6235147999 |
| C147 | 8.8086621107 | -3.6397403050 | -0.2464186289 |
| H148 | 8.9276737042 | -2.7420136360 | 0.3669797727 |
| C149 | 7.8370525301 | -4.5729615644 | 0.5014498014 |

| | | | |
|---|---|---|---|
| H150 | 6.8610125900 | -4.0941871197 | 0.6418654752 |
| H151 | 7.6796864422 | -5.5094489833 | -0.0446392940 |
| H152 | 8.2380912165 | -4.8305114253 | 1.4875748726 |
| C153 | 10.2026340758 | -4.2840323740 | -0.3878604152 |
| H154 | 10.9043500844 | -3.6127524223 | -0.8929367737 |
| H155 | 10.6143421775 | -4.5234309387 | 0.5982735606 |
| H156 | 10.1608980912 | -5.2146752401 | -0.9632440290 |
| C157 | 8.4469819731 | -0.5187858053 | 3.4416970315 |
| H158 | 8.7457631052 | -0.9432128492 | 2.4800296842 |
| C159 | 7.7297616834 | -1.6382091900 | 4.2190768477 |
| H160 | 7.4009855327 | -1.3023453768 | 5.2087781980 |
| H161 | 6.8536864984 | -2.0018173884 | 3.6693886602 |
| H162 | 8.4028250945 | -2.4881391373 | 4.3702937435 |
| C163 | 9.7334728138 | -0.0741418892 | 4.1653968113 |
| H164 | 10.4045337882 | -0.9267575660 | 4.3099142561 |
| H165 | 10.2731231093 | 0.6861961229 | 3.5919275893 |
| H166 | 9.5129692944 | 0.3487692278 | 5.1505170921 |

**C’’D’-TS**

| | | | |
|---|---|---|---|
| C1 | 2.3867064813 | 0.0185145369 | 0.0600442226 |
| C2 | 3.4577200898 | -0.1720728144 | 1.1678788528 |
| C3 | 3.8639378662 | -1.5693600655 | 1.4935137572 |
| C4 | 3.5965413967 | -2.6430392175 | 0.7217332880 |
| H5 | 2.3114053919 | 1.0802483203 | -0.1992882696 |
| H6 | 2.7225129627 | -0.4881395975 | -0.8465401688 |
| H7 | 4.4193782702 | -1.6808560215 | 2.4185624630 |
| H8 | 3.0457228550 | -2.5729984619 | -0.2113738190 |

| | | | |
|---|---|---|---|
| C9 | 1.0335110822 | -0.4527538414 | 0.4605227227 |
| C10 | 0.0966971792 | -0.5915706947 | 1.2623921630 |
| C11 | -0.9242540536 | -0.6984905438 | 2.2543271687 |
| C12 | -1.8170113604 | 0.3727439378 | 2.4620297980 |
| C13 | -1.0093230859 | -1.8515788897 | 3.0605929585 |
| C14 | -2.7721469138 | 0.2895633265 | 3.4690995145 |
| H15 | -1.7495391069 | 1.2565820579 | 1.8350633067 |
| C16 | -1.9727634174 | -1.9232610658 | 4.0605377777 |
| H17 | -0.3184084520 | -2.6725391217 | 2.8964244508 |
| C18 | -2.8518371143 | -0.8558365519 | 4.2662261383 |
| H19 | -3.4564673366 | 1.1163059802 | 3.6335069919 |
| H20 | -2.0400460570 | -2.8110715454 | 4.6820018893 |
| H21 | -3.6011249811 | -0.9167174249 | 5.0503160878 |
| O22 | 3.9447189008 | -3.9221189205 | 0.9455294490 |
| C23 | 4.6731501864 | -4.2273640980 | 2.1365499167 |
| H24 | 4.8464307045 | -5.3033495378 | 2.1157345767 |
| H25 | 5.6333968981 | -3.6986068039 | 2.1548555939 |
| H26 | 4.0917776948 | -3.9637271513 | 3.0282743032 |
| Au27 | 5.3563313902 | 0.6525312228 | 0.3457422921 |
| C28 | 9.2056872186 | 1.8880680158 | -0.9400969396 |
| C29 | 8.4627079710 | 2.3662675708 | -1.9711691400 |
| H30 | 10.2661131883 | 1.9252368910 | -0.7470086673 |
| H31 | 8.7421273513 | 2.9035889658 | -2.8634522997 |
| C32 | 7.0534903711 | 1.3568781240 | -0.5273750216 |
| N33 | 8.3250719623 | 1.2701904142 | -0.0634176336 |
| N34 | 7.1432001302 | 2.0305813052 | -1.7021654417 |
| N35 | 3.2460146693 | 0.5906559626 | 2.2971194543 |
| C36 | 3.3109144822 | 1.8485194357 | 2.7082170536 |
| C37 | 2.5433400861 | 2.1358143818 | 3.8381078029 |

| | | | |
|---|---|---|---|
| H38 | 3.9806336861 | 2.5326126048 | 2.2028713424 |
| C39 | 1.9287753600 | 0.9542498093 | 4.2136464508 |
| H40 | 2.5225510389 | 3.0780096280 | 4.3638043163 |
| H41 | 1.3544202672 | 0.7614681820 | 5.1193034280 |
| O42 | 2.1326855206 | -0.0463054203 | 3.4191770685 |
| Au43 | -0.4195498148 | -1.5285473089 | -0.9108714869 |
| C44 | -1.5152565025 | -2.4668481629 | -2.3492012059 |
| N45 | -1.1819085443 | -2.7021410037 | -3.6435797377 |
| N46 | -2.7630996318 | -2.9816108546 | -2.2031222574 |
| C47 | -2.2138646866 | -3.3558510766 | -4.3027934551 |
| C48 | -3.2069914227 | -3.5319700888 | -3.3963891531 |
| H49 | -2.1366933103 | -3.6300607934 | -5.3428262556 |
| H50 | -4.1791491537 | -3.9908318199 | -3.4807574245 |
| C51 | 6.0281155814 | 2.3195052386 | -2.5766066851 |
| C52 | 5.3630603496 | 3.5523877170 | -2.4343638331 |
| C53 | 5.6703573230 | 1.3524867596 | -3.5376914587 |
| C54 | 4.2835317789 | 3.7957878375 | -3.2913142277 |
| C55 | 4.5763167638 | 1.6480966093 | -4.3587199396 |
| C56 | 3.8911101604 | 2.8540259859 | -4.2378771229 |
| H57 | 3.7480820148 | 4.7380855641 | -3.2214775820 |
| H58 | 4.2622101975 | 0.9269750457 | -5.1073042448 |
| H59 | 3.0527205912 | 3.0683692980 | -4.8952991462 |
| C60 | 8.7244713344 | 0.6040668860 | 1.1582198459 |
| C61 | 8.8058233636 | 1.3651240465 | 2.3403633458 |
| C62 | 9.0355830461 | -0.7690580697 | 1.0968518785 |
| C63 | 9.2019516399 | 0.6939240442 | 3.5026572034 |
| C64 | 9.4266601057 | -1.3843971269 | 2.2916063970 |
| C65 | 9.5068216965 | -0.6639918509 | 3.4800532428 |
| H66 | 9.2821945026 | 1.2436492361 | 4.4357481400 |

| | | | |
|---|---|---|---|
| H67 | 9.6832683511 | -2.4397898034 | 2.2888537389 |
| H68 | 9.8200995270 | -1.1623658926 | 4.3931449207 |
| C69 | -3.5590649040 | -2.9825410974 | -0.9941969232 |
| C70 | -3.4241671380 | -4.0685172303 | -0.1067055479 |
| C71 | -4.4830608570 | -1.9362034669 | -0.8065364415 |
| C72 | -4.2606064234 | -4.0773862282 | 1.0152179228 |
| C73 | -5.2906496865 | -2.0000649137 | 0.3346395726 |
| C74 | -5.1833523461 | -3.0579808196 | 1.2327629434 |
| H75 | -4.1993427084 | -4.9018233609 | 1.7196147991 |
| H76 | -6.0216875100 | -1.2175808019 | 0.5153107595 |
| H77 | -5.8314343486 | -3.0938975742 | 2.1040927940 |
| C78 | 0.0688853315 | -2.3696662971 | -4.2887265448 |
| C79 | 0.1764124663 | -1.1256547308 | -4.9398122953 |
| C80 | 1.0842118052 | -3.3471600953 | -4.3060366057 |
| C81 | 1.3665295006 | -0.8736465389 | -5.6318986916 |
| C82 | 2.2524762340 | -3.0363729790 | -5.0109641486 |
| C83 | 2.3882258989 | -1.8185358785 | -5.6728669025 |
| H84 | 1.4848293120 | 0.0663362170 | -6.1631107151 |
| H85 | 3.0569795697 | -3.7644466735 | -5.0589810398 |
| H86 | 3.2922007255 | -1.6127152034 | -6.2400687829 |
| C87 | 8.4950240752 | 2.8596267866 | 2.3906972807 |
| H88 | 8.2197643140 | 3.1881437485 | 1.3834747828 |
| C89 | 8.9830830056 | -1.5814330022 | -0.1958377128 |
| H90 | 8.6349076901 | -0.9261953985 | -1.0005085761 |
| C91 | 7.2910318939 | 3.1519622476 | 3.3077394398 |
| H92 | 7.0491273653 | 4.2203786144 | 3.2921451183 |
| H93 | 6.4065946131 | 2.5915162147 | 2.9823529486 |
| H94 | 7.4971144659 | 2.8738442289 | 4.3467216373 |
| C95 | 9.7286484971 | 3.6801544819 | 2.8171269077 |

| | | | |
|---|---|---|---|
| H96 | 10.5802396564 | 3.4954523056 | 2.1548350610 |
| H97 | 9.5036441837 | 4.7513829184 | 2.7873269801 |
| H98 | 10.0426421479 | 3.4364576064 | 3.8372887957 |
| C99 | 7.9779017387 | -2.7457346473 | -0.0941131842 |
| H100 | 8.2730929952 | -3.4656258734 | 0.6772178107 |
| H101 | 6.9722002172 | -2.3833520940 | 0.1462635547 |
| H102 | 7.9255287614 | -3.2880228217 | -1.0445922618 |
| C103 | 10.3836928989 | -2.0890774667 | -0.5943076844 |
| H104 | 10.3390101957 | -2.6233240775 | -1.5492323881 |
| H105 | 11.0936891098 | -1.2629265484 | -0.7024652367 |
| H106 | 10.7891403346 | -2.7796080981 | 0.1525565807 |
| C107 | 6.4231503700 | 0.0342524938 | -3.7131873172 |
| H108 | 7.2974854230 | 0.0476988836 | -3.0553932966 |
| C109 | 5.5564454609 | -1.1695049458 | -3.2931896092 |
| H110 | 5.2510508005 | -1.0914722415 | -2.2433011448 |
| H111 | 4.6499073480 | -1.2442762744 | -3.9033208296 |
| H112 | 6.1175766773 | -2.1032800467 | -3.4114899669 |
| C113 | 6.9462726309 | -0.1343651473 | -5.1534123147 |
| H114 | 7.5727547943 | 0.7107303885 | -5.4548250332 |
| H115 | 7.5477117714 | -1.0458102769 | -5.2337756996 |
| H116 | 6.1283533624 | -0.2152676310 | -5.8772258960 |
| C117 | 5.7772262139 | 4.6079911663 | -1.4114888992 |
| H118 | 6.6261177493 | 4.2189054939 | -0.8409940513 |
| C119 | 4.6451360757 | 4.8893862430 | -0.4035993321 |
| H120 | 3.7606863734 | 5.3109686157 | -0.8932519522 |
| H121 | 4.3380528526 | 3.9678940046 | 0.1059176971 |
| H122 | 4.9772815577 | 5.6078161773 | 0.3536045093 |
| C123 | 6.2428283579 | 5.9066012164 | -2.1009373197 |
| H124 | 6.5855275079 | 6.6330884543 | -1.3567864563 |

| | | | |
|---|---|---|---|
| H125 | 7.0691634773 | 5.7174253585 | -2.7934674074 |
| H126 | 5.4327679058 | 6.3734622949 | -2.6710734631 |
| C127 | 0.9460481159 | -4.7015414044 | -3.6129100446 |
| H128 | -0.0626314884 | -4.7725500949 | -3.1945137321 |
| C129 | 1.9337384593 | -4.8303176465 | -2.4367345087 |
| H130 | 1.7670299471 | -4.0421546167 | -1.6939962110 |
| H131 | 1.8092609428 | -5.7958568828 | -1.9355543269 |
| H132 | 2.9735550220 | -4.7642635812 | -2.7747658741 |
| C133 | 1.1076975375 | -5.8691925746 | -4.6058508890 |
| H134 | 2.1150263585 | -5.9028291326 | -5.0333751859 |
| H135 | 0.9335800020 | -6.8239006417 | -4.0990728252 |
| H136 | 0.3967698934 | -5.7925303498 | -5.4344946191 |
| C137 | -0.9376689121 | -0.0813121603 | -4.9269652839 |
| H138 | -1.7890849779 | -0.4945612087 | -4.3775518636 |
| C139 | -0.4948946524 | 1.1941453892 | -4.1830494445 |
| H140 | -1.3128923066 | 1.9217026272 | -4.1510509364 |
| H141 | -0.1995121340 | 0.9687863770 | -3.1520900500 |
| H142 | 0.3561835667 | 1.6729944302 | -4.6795968517 |
| C143 | -1.4294529213 | 0.2443330806 | -6.3508478193 |
| H144 | -2.2721195323 | 0.9421166508 | -6.3109493798 |
| H145 | -0.6443082402 | 0.7101120333 | -6.9554821100 |
| H146 | -1.7622483441 | -0.6566569981 | -6.8757316528 |
| C147 | -4.6344605212 | -0.7743201867 | -1.7863836518 |
| H148 | -3.9255605564 | -0.9213956547 | -2.6072270704 |
| C149 | -4.2803780492 | 0.5692446459 | -1.1196481182 |
| H150 | -3.2636086797 | 0.5527007601 | -0.7115369029 |
| H151 | -4.3432077107 | 1.3850444665 | -1.8475256383 |
| H152 | -4.9681829923 | 0.8043415363 | -0.3005234435 |
| C153 | -6.0462362290 | -0.7354865189 | -2.4048238283 |

| | | | |
|---|---|---|---|
| H154 | -6.1171978549 | 0.0699384033 | -3.1432511412 |
| H155 | -6.2901458833 | -1.6767224317 | -2.9078816581 |
| H156 | -6.8150029666 | -0.5562475376 | -1.6460181636 |
| C157 | -2.4506680606 | -5.2212413274 | -0.3431279005 |
| H158 | -1.8452742162 | -4.9852951786 | -1.2239450164 |
| C159 | -3.2062639287 | -6.5317480478 | -0.6422888304 |
| H160 | -3.8635538604 | -6.4263522873 | -1.5115288057 |
| H161 | -2.4998645157 | -7.3422227691 | -0.8497081871 |
| H162 | -3.8261471331 | -6.8387485995 | 0.2064045640 |
| C163 | -1.4744457985 | -5.3983363435 | 0.8360336899 |
| H164 | -0.9151573174 | -4.4759369380 | 1.0284723021 |
| H165 | -1.9961371743 | -5.6752976169 | 1.7582987480 |
| H166 | -0.7535516979 | -6.1929868893 | 0.6158548981 |

**D'**

| | | | |
|---|---|---|---|
| C1 | 2.7618532106 | 0.3560442954 | 0.6976532664 |
| C2 | 3.0129367722 | -0.9133096232 | 1.5225517978 |
| C3 | 3.7295950816 | -2.0765597905 | 0.9099456718 |
| C4 | 3.3944172736 | -2.5632010481 | -0.3515114503 |
| H5 | 3.3727863692 | 1.1504870144 | 1.1466543349 |
| H6 | 3.1512253084 | 0.2243147614 | -0.3159929772 |
| H7 | 4.1002670173 | -2.7781100023 | 1.6523168479 |
| H8 | 2.8134143443 | -1.9736933228 | -1.0570881107 |
| C9 | 1.3789138503 | 0.8716998351 | 0.5895215880 |
| C10 | 0.5863949796 | 1.8235994013 | 0.6681110422 |
| C11 | -0.2471682332 | 2.9662715278 | 0.8395544349 |
| C12 | -0.3148134965 | 3.9497167845 | -0.1694414756 |
| C13 | -0.9550927015 | 3.1472325383 | 2.0454448344 |

| | | | |
|---|---|---|---|
| C14 | -1.0769409619 | 5.0947185692 | 0.0328102584 |
| H15 | 0.2309423902 | 3.8033015679 | -1.0965003205 |
| C16 | -1.7170775603 | 4.2950274560 | 2.2321496501 |
| H17 | -0.8892510313 | 2.3923413365 | 2.8225640418 |
| C18 | -1.7796977957 | 5.2665029189 | 1.2291612142 |
| H19 | -1.1279631147 | 5.8540120711 | -0.7415843131 |
| H20 | -2.2613428028 | 4.4368498610 | 3.1607800707 |
| H21 | -2.3759874100 | 6.1614999496 | 1.3810831611 |
| O22 | 3.6439391896 | -3.7607128038 | -0.8358717751 |
| C23 | 4.3704953557 | -4.7204272245 | -0.0337507768 |
| H24 | 4.4471875360 | -5.6131618210 | -0.6513516103 |
| H25 | 5.3672146785 | -4.3308137455 | 0.1971996092 |
| H26 | 3.8182393503 | -4.9405669125 | 0.8837999877 |
| Au27 | 5.5273940649 | -1.1049988208 | -0.0977301759 |
| C28 | 8.3717507798 | 1.7546848390 | -1.3859740536 |
| C29 | 9.1022716605 | 0.6208844777 | -1.5558445237 |
| H30 | 8.6133056240 | 2.7908182438 | -1.5633212319 |
| H31 | 10.1095311856 | 0.4693385834 | -1.9104607119 |
| C32 | 7.0877365206 | 0.0151051083 | -0.7583902426 |
| N33 | 7.1333140588 | 1.3664052383 | -0.8939570863 |
| N34 | 8.2976725339 | -0.4373720070 | -1.1637770147 |
| N35 | 2.7550510773 | -1.0400357020 | 2.7570978102 |
| C36 | 2.1686897770 | -0.2524091973 | 3.7062593294 |
| C37 | 0.8543683987 | -0.2251867518 | 4.0559933243 |
| H38 | 2.8923694312 | 0.2567571630 | 4.3463548846 |
| C39 | -0.1966580024 | -0.9115989596 | 3.3192810818 |
| H40 | 0.5821328121 | 0.3004748363 | 4.9659703026 |
| H41 | -1.1902729100 | -0.9294946432 | 3.8094144843 |
| O42 | -0.0518687091 | -1.4483909985 | 2.2213248826 |

| | | | |
|---|---|---|---|
| Au43 | -0.3206269672 | -0.1835163939 | -0.4178071739 |
| C44 | -1.6323470768 | -1.3783784236 | -1.4303451377 |
| N45 | -1.8091924019 | -1.4112896528 | -2.7806678094 |
| N46 | -2.5111867086 | -2.2934574543 | -0.9394741259 |
| C47 | -2.7885239823 | -2.3282927905 | -3.1253272821 |
| C48 | -3.2287491688 | -2.8823639397 | -1.9680026787 |
| H49 | -3.0825998813 | -2.4875368758 | -4.1502092909 |
| H50 | -3.9834781155 | -3.6274548570 | -1.7736457827 |
| C51 | 6.0602866297 | 2.2850325308 | -0.5632047268 |
| C52 | 5.9539995743 | 2.7443311595 | 0.7666747818 |
| C53 | 5.1887032637 | 2.6966749349 | -1.5927883829 |
| C54 | 4.9093259311 | 3.6347892524 | 1.0532457941 |
| C55 | 4.1642857799 | 3.5890908331 | -1.2470294819 |
| C56 | 4.0284232048 | 4.0546190225 | 0.0588730488 |
| H57 | 4.8024414961 | 4.0244863429 | 2.0612832368 |
| H58 | 3.4799606459 | 3.9365943032 | -2.0154317352 |
| H59 | 3.2365336947 | 4.7579082742 | 0.3023163809 |
| C60 | 8.7080411429 | -1.8283060331 | -1.1239532506 |
| C61 | 9.3256969924 | -2.2985505321 | 0.0527237190 |
| C62 | 8.4701884755 | -2.6358539769 | -2.2532463670 |
| C63 | 9.7001595007 | -3.6469646757 | 0.0749415456 |
| C64 | 8.8664477524 | -3.9770533656 | -2.1704272955 |
| C65 | 9.4725301486 | -4.4763600499 | -1.0204740195 |
| H66 | 10.1830117027 | -4.0512318379 | 0.9597017224 |
| H67 | 8.7079179648 | -4.6342566943 | -3.0201004489 |
| H68 | 9.7801733187 | -5.5174960193 | -0.9804352947 |
| C69 | -2.7723534410 | -2.6299529006 | 0.4469503217 |
| C70 | -3.7246346762 | -1.8669873813 | 1.1492276645 |
| C71 | -2.1429577773 | -3.7672419467 | 0.9863755169 |

| | | | |
|---|---|---|---|
| C72 | -4.0365846247 | -2.2816234583 | 2.4495612798 |
| C73 | -2.5061253433 | -4.1416310101 | 2.2841723537 |
| C74 | -3.4420846840 | -3.4092991439 | 3.0080252782 |
| H75 | -4.7700286518 | -1.7245072006 | 3.0251063990 |
| H76 | -2.0516518423 | -5.0189609923 | 2.7346312749 |
| H77 | -3.7147203232 | -3.7228333694 | 4.0119460588 |
| C78 | -1.1541909051 | -0.5707811087 | -3.7602160168 |
| C79 | -1.7769575681 | 0.6398552371 | -4.1194701106 |
| C80 | 0.0337000831 | -1.0378262400 | -4.3583702494 |
| C81 | -1.1580065393 | 1.4009916131 | -5.1184798848 |
| C82 | 0.6030171786 | -0.2369254143 | -5.3537538851 |
| C83 | 0.0145385074 | 0.9687594509 | -5.7288260457 |
| H84 | -1.6099618340 | 2.3374860164 | -5.4315769418 |
| H85 | 1.5104434186 | -0.5636479876 | -5.8518008262 |
| H86 | 0.4682115393 | 1.5694544662 | -6.5122610805 |
| C87 | 5.3254296988 | 2.2214641760 | -3.0376292359 |
| H88 | 6.1909366271 | 1.5558701206 | -3.0994603724 |
| C89 | 5.5825489462 | 3.3997917869 | -3.9982345427 |
| H90 | 5.7428443173 | 3.0317882660 | -5.0168629264 |
| H91 | 4.7339191440 | 4.0913940987 | -4.0265142636 |
| H92 | 6.4673384365 | 3.9726512957 | -3.7036537702 |
| C93 | 4.0975425038 | 1.4021430900 | -3.4786138934 |
| H94 | 3.9505582353 | 0.5318627612 | -2.8286225375 |
| H95 | 3.1796323758 | 1.9993843955 | -3.4578383259 |
| H96 | 4.2305503613 | 1.0371365154 | -4.5022857825 |
| C97 | 6.9321893408 | 2.3451138677 | 1.8698706194 |
| H98 | 7.6534395623 | 1.6373438921 | 1.4504804840 |
| C99 | 7.7271805076 | 3.5667471603 | 2.3753432207 |
| H100 | 7.0717245197 | 4.3077592521 | 2.8448243143 |

| | | | |
|---|---|---|---|
| H101 | 8.4654292413 | 3.2576704715 | 3.1222805660 |
| H102 | 8.2603106001 | 4.0642314950 | 1.5591596590 |
| C103 | 6.2196965106 | 1.6288714783 | 3.0341601908 |
| H104 | 5.6875152500 | 0.7345100779 | 2.6892856909 |
| H105 | 6.9470352582 | 1.3155725174 | 3.7898520325 |
| H106 | 5.4967358961 | 2.2865547823 | 3.5296480279 |
| C107 | 9.5937199165 | -1.4115777557 | 1.2680592991 |
| H108 | 9.2545509074 | -0.3965302299 | 1.0371932247 |
| C109 | 11.1002474062 | -1.3227418727 | 1.5832610469 |
| H110 | 11.2737780108 | -0.6327488067 | 2.4154825948 |
| H111 | 11.5112898473 | -2.2956162500 | 1.8706006854 |
| H112 | 11.6698990498 | -0.9620935362 | 0.7210418632 |
| C113 | 8.7956837522 | -1.8870942421 | 2.4986808053 |
| H114 | 7.7184481727 | -1.9013856467 | 2.2949870003 |
| H115 | 9.0926080317 | -2.8950464961 | 2.8062852682 |
| H116 | 8.9710443560 | -1.2185493587 | 3.3484330742 |
| C117 | 7.8191065611 | -2.1097571813 | -3.5298305453 |
| H118 | 7.6021920474 | -1.0462070696 | -3.3900640214 |
| C119 | 6.4769745509 | -2.8152395015 | -3.8072726310 |
| H120 | 6.6168055161 | -3.8863131425 | -3.9880171995 |
| H121 | 5.7855741699 | -2.7050230146 | -2.9638772342 |
| H122 | 5.9993379024 | -2.3913215465 | -4.6970349046 |
| C123 | 8.7695704983 | -2.2282534509 | -4.7376272221 |
| H124 | 8.3080254160 | -1.7938551293 | -5.6305120162 |
| H125 | 9.7139828225 | -1.7054850388 | -4.5567966809 |
| H126 | 9.0062535663 | -3.2730944346 | -4.9630730851 |
| C127 | -3.0863987380 | 1.1219081603 | -3.4991455881 |
| H128 | -3.3787489590 | 0.4138057147 | -2.7179813702 |
| C129 | -2.9243640794 | 2.4958285496 | -2.8208273347 |

| | | | |
|---|---|---|---|
| H130 | -3.8646855817 | 2.7965581396 | -2.3463219334 |
| H131 | -2.1493531645 | 2.4679423294 | -2.0478371798 |
| H132 | -2.6564458225 | 3.2763620675 | -3.5411760529 |
| C133 | -4.2210729063 | 1.1443980937 | -4.5434838354 |
| H134 | -4.3714909603 | 0.1585147311 | -4.9955951927 |
| H135 | -5.1634429673 | 1.4478802537 | -4.0760193159 |
| H136 | -4.0084665577 | 1.8510816117 | -5.3524703175 |
| C137 | 0.6577569133 | -2.3798925387 | -3.9851058999 |
| H138 | 0.2823667682 | -2.6589146496 | -2.9950541084 |
| C139 | 2.1912816301 | -2.3067242970 | -3.8834050563 |
| H140 | 2.5098179365 | -1.4559613436 | -3.2708083346 |
| H141 | 2.5858758954 | -3.2280644608 | -3.4414220436 |
| H142 | 2.6646669546 | -2.1945977900 | -4.8646746582 |
| C143 | 0.2372737101 | -3.4886562212 | -4.9725283135 |
| H144 | 0.6765384670 | -4.4492952976 | -4.6822333967 |
| H145 | -0.8491327245 | -3.6141928285 | -5.0033515557 |
| H146 | 0.5750301068 | -3.2556077578 | -5.9877226462 |
| C147 | -4.4235976667 | -0.6458340402 | 0.5555051261 |
| H148 | -4.0807063840 | -0.5178873481 | -0.4759830594 |
| C149 | -5.9522499697 | -0.8350128943 | 0.5076568611 |
| H150 | -6.4277211756 | 0.0219070120 | 0.0191473563 |
| H151 | -6.2256406450 | -1.7365359102 | -0.0488949773 |
| H152 | -6.3810473254 | -0.9215831822 | 1.5112213444 |
| C153 | -4.0468649172 | 0.6378538695 | 1.3203314953 |
| H154 | -4.3703285056 | 0.5867965950 | 2.3651268326 |
| H155 | -2.9642855551 | 0.8040614192 | 1.3104486167 |
| H156 | -4.5289203014 | 1.5105616226 | 0.8663132445 |
| C157 | -1.1078153060 | -4.5845785435 | 0.2185974460 |
| H158 | -0.8907503431 | -4.0647557081 | -0.7205240710 |

| | | | |
|---|---|---|---|
| C159 | -1.6484278734 | -5.9834342075 | -0.1371679870 |
| H160 | -0.9063625930 | -6.5499944080 | -0.7097612053 |
| H161 | -1.8834856878 | -6.5585576145 | 0.7644952009 |
| H162 | -2.5621259681 | -5.9233992525 | -0.7374018107 |
| C163 | 0.2191114513 | -4.6849181811 | 0.9976706343 |
| H164 | 0.5748980305 | -3.6935312673 | 1.2925007972 |
| H165 | 0.1072133351 | -5.2838758000 | 1.9078066242 |
| H166 | 0.9795562826 | -5.1753916913 | 0.3775261744 |

**D**

| | | | |
|---|---|---|---|
| C1 | 3.2185388761 | -0.4156359141 | 0.8992633816 |
| C2 | 2.3736433449 | 0.8336123929 | 0.6392373339 |
| C3 | 1.1432102653 | 0.9858628238 | 1.3978097728 |
| C4 | 0.7537433960 | 0.1389940341 | 2.3829726861 |
| H5 | 3.5387864889 | -0.4171330587 | 1.9504020114 |
| H6 | 2.6057468643 | -1.3134017950 | 0.7633316302 |
| H7 | 0.5067072944 | 1.8211400415 | 1.1337348690 |
| H8 | 1.3433296692 | -0.7227866245 | 2.6832582303 |
| C9 | 4.4363807425 | -0.5017491372 | 0.0697836821 |
| C10 | 5.6326454893 | -0.4108459599 | -0.2298707071 |
| O11 | -0.3540021843 | 0.2017493844 | 3.1206545493 |
| C12 | -1.2678841987 | 1.2833136360 | 2.8998708997 |
| H13 | -2.0779185389 | 1.1378199787 | 3.6135454962 |
| H14 | -0.7780910216 | 2.2458388370 | 3.0827971983 |
| H15 | -1.6616487727 | 1.2550379190 | 1.8780570744 |
| Au16 | 4.3805915908 | -1.3846853192 | -2.0356322793 |
| C17 | 3.9284915487 | -2.3472057850 | -3.7691799804 |

| | | | |
|---|---|---|---|
| N18 | 4.5852655495 | -3.4217106715 | -4.2813519901 |
| N19 | 2.8827932535 | -2.1305195128 | -4.6092682757 |
| C20 | 3.9529916033 | -3.8752888702 | -5.4291385720 |
| C21 | 2.8809198197 | -3.0680362224 | -5.6315138389 |
| H22 | 4.3269912849 | -4.7185385456 | -5.9872197481 |
| H23 | 2.1275697105 | -3.0618067859 | -6.4029336927 |
| N24 | 2.8179345856 | 1.6578210137 | -0.2557340627 |
| C25 | 2.2479855425 | 2.9012474719 | -0.4594274717 |
| C26 | 3.0474888575 | 3.0781227104 | -2.8057239533 |
| C27 | 2.3538034307 | 3.5864638928 | -1.6235639059 |
| H28 | 1.7344540215 | 3.3812947759 | 0.3780380920 |
| H29 | 3.5428373559 | 2.0952148642 | -2.6874150480 |
| H30 | 1.9067945440 | 4.5724521888 | -1.7096209633 |
| O31 | 3.0960986591 | 3.6879477445 | -3.8660696788 |
| C32 | 7.0341813792 | -0.2381554775 | -0.4685162387 |
| C33 | 7.9385914401 | -1.2791210896 | -0.1824382428 |
| C34 | 7.5107995708 | 0.9951259934 | -0.9548496816 |
| C35 | 9.3017188042 | -1.0792848422 | -0.3743284726 |
| H36 | 7.5647725867 | -2.2279807732 | 0.1890252041 |
| C37 | 8.8766868991 | 1.1808884780 | -1.1425394285 |
| H38 | 6.8067012068 | 1.7920410689 | -1.1722195428 |
| C39 | 9.7712043742 | 0.1468657137 | -0.8533995257 |
| H40 | 10.0001999722 | -1.8794559497 | -0.1493086772 |
| H41 | 9.2445389145 | 2.1323689731 | -1.5141258162 |
| H42 | 10.8367342805 | 0.2974727462 | -1.0010874328 |
| C43 | 5.7828415537 | -4.0288305684 | -3.7365304936 |
| C44 | 7.0351105151 | -3.5185811288 | -4.1340657329 |
| C45 | 5.6389311624 | -5.1357851811 | -2.8766847110 |
| C46 | 8.1736779757 | -4.1526465342 | -3.6209496637 |

| | | | |
|---|---|---|---|
| C47 | 6.8125932577 | -5.7286243738 | -2.3948779053 |
| C48 | 8.0646492882 | -5.2426661839 | -2.7619476931 |
| H49 | 9.1571502801 | -3.7948805771 | -3.9097061307 |
| H50 | 6.7439361710 | -6.5870665986 | -1.7335609771 |
| H51 | 8.9623700034 | -5.7243718868 | -2.3844451162 |
| C52 | 1.9180873445 | -1.0518398642 | -4.5068704118 |
| C53 | 0.7865710096 | -1.2333077719 | -3.6875021017 |
| C54 | 2.1468616399 | 0.1133360197 | -5.2690803053 |
| C55 | -0.1203192927 | -0.1688463767 | -3.6115189966 |
| C56 | 1.2162108418 | 1.1512137653 | -5.1349256127 |
| C57 | 0.0992039975 | 1.0116444969 | -4.3157802756 |
| H58 | -1.0121751956 | -0.2693654008 | -3.0004438968 |
| H59 | 1.3697874248 | 2.0788506637 | -5.6747255420 |
| H60 | -0.6124148756 | 1.8286247490 | -4.2354332930 |
| C61 | 4.2794892854 | -5.7090207368 | -2.4846475588 |
| H62 | 3.5021189384 | -5.0846093251 | -2.9346924784 |
| C63 | 4.0618792815 | -5.6681137705 | -0.9601949680 |
| H64 | 4.7866682542 | -6.2944303510 | -0.4298487020 |
| H65 | 4.1553576474 | -4.6465792445 | -0.5760468584 |
| H66 | 3.0618747481 | -6.0377685012 | -0.7095771049 |
| C67 | 4.0966120672 | -7.1364205801 | -3.0380615958 |
| H68 | 3.0999524337 | -7.5171860802 | -2.7920262780 |
| H69 | 4.2085863529 | -7.1623671798 | -4.1270298597 |
| H70 | 4.8314394745 | -7.8282709383 | -2.6135900375 |
| C71 | 0.4983275112 | -2.5382574673 | -2.9488753345 |
| H72 | 1.3821916884 | -3.1788804756 | -3.0270270270 |
| C73 | 0.2390800468 | -2.3135341489 | -1.4484905995 |
| H74 | 1.0722452794 | -1.7758862088 | -0.9846009546 |
| H75 | -0.6720279893 | -1.7324164255 | -1.2726288266 |

| | | | |
|---|---|---|---|
| H76 | 0.1193042254 | -3.2738609603 | -0.9355618541 |
| C77 | -0.6759334361 | -3.2921541733 | -3.6054995681 |
| H78 | -0.4786595792 | -3.4995076891 | -4.6622471072 |
| H79 | -0.8499117635 | -4.2479849050 | -3.0999640353 |
| H80 | -1.6012017139 | -2.7091924177 | -3.5493416000 |
| C81 | 7.1865925132 | -2.3588146764 | -5.1149143336 |
| H82 | 6.1936948177 | -1.9452090055 | -5.3134494530 |
| C83 | 7.7570517984 | -2.8499417866 | -6.4612701930 |
| H84 | 7.8202191415 | -2.0209452051 | -7.1738245064 |
| H85 | 8.7634690083 | -3.2643770985 | -6.3404556303 |
| H86 | 7.1285487674 | -3.6293332447 | -6.9048272726 |
| C87 | 8.0382890016 | -1.2159214223 | -4.5329761256 |
| H88 | 7.6190094455 | -0.8447661080 | -3.5929364158 |
| H89 | 9.0688027736 | -1.5318324556 | -4.3400617743 |
| H90 | 8.0787256554 | -0.3806887161 | -5.2402254329 |
| C91 | 3.3434288666 | 0.2659262154 | -6.2087920659 |
| H92 | 3.7631064995 | -0.7296648471 | -6.3849302641 |
| C93 | 4.4549750500 | 1.1291889198 | -5.5781294570 |
| H94 | 4.0930168279 | 2.1329987291 | -5.3351063301 |
| H95 | 4.8317869382 | 0.6733067006 | -4.6552273481 |
| H96 | 5.2966479650 | 1.2244265801 | -6.2736024464 |
| C97 | 2.9289155310 | 0.8206765116 | -7.5856894408 |
| H98 | 3.7843249854 | 0.7987984804 | -8.2687329128 |
| H99 | 2.1230031854 | 0.2301141939 | -8.0339159155 |
| H100 | 2.5914207942 | 1.8598806114 | -7.5240884563 |

**EF-TS**

| | | | |
|---|---|---|---|
| C1 | 3.2073480523 | -1.0565684049 | 0.6343991154 |
| C2 | 3.1459864536 | 0.0124908096 | 1.7274120320 |
| C3 | 2.5390037521 | -0.2867716192 | 3.0097919199 |
| C4 | 2.1095754380 | -1.5280566273 | 3.3529519006 |
| H5 | 3.7328229286 | -1.9444283446 | 1.0077922734 |
| H6 | 2.1934265838 | -1.3910857493 | 0.3846870957 |
| H7 | 2.4291985722 | 0.5374036627 | 3.7053396574 |
| H8 | 2.1907117778 | -2.3770617142 | 2.6800160435 |
| C9 | 3.9113713737 | -0.5376965081 | -0.5769889820 |
| C10 | 4.4799002502 | 0.5966089003 | -0.7027472830 |
| O11 | 1.5525286331 | -1.9122338960 | 4.4965007079 |
| C12 | 1.3586769916 | -0.9444228622 | 5.5382012313 |
| H13 | 0.9035538368 | -1.4882338352 | 6.3651401752 |
| H14 | 2.3183455440 | -0.5214878363 | 5.8534020700 |
| H15 | 0.6891207702 | -0.1457329542 | 5.2030237827 |
| Au16 | 4.0187086867 | -1.5328904579 | -2.4462962928 |
| C17 | 4.0176116697 | -2.5762853908 | -4.2098686096 |
| N18 | 4.5428463840 | -3.8111262837 | -4.4283081996 |
| N19 | 3.4311191606 | -2.2308259301 | -5.3869972948 |
| C20 | 4.2835753925 | -4.2351656874 | -5.7239103498 |
| C21 | 3.5835588367 | -3.2409265171 | -6.3269839907 |
| H22 | 4.6216384209 | -5.1924708850 | -6.0869544867 |
| H23 | 3.1853390907 | -3.1520910583 | -7.3249955501 |
| N24 | 3.6310144317 | 1.1645400718 | 1.4015570357 |
| C25 | 3.7725678278 | 2.2398816359 | 2.2712284513 |
| C26 | 2.7978008083 | 3.9027241783 | 0.7019495044 |
| C27 | 3.4400290037 | 3.5134188513 | 1.9639359881 |
| H28 | 4.2317519579 | 2.0264397451 | 3.2393961558 |
| H29 | 2.5706131572 | 3.0644040724 | 0.0120659601 |

| | | | |
|---|---|---|---|
| H30 | 3.6314453262 | 4.3067575057 | 2.6806268833 |
| O31 | 2.5207967889 | 5.0526291674 | 0.4099646731 |
| C32 | 5.2674198835 | 1.6649787992 | -1.2086469544 |
| C33 | 6.6593677603 | 1.6912315930 | -0.9634661180 |
| C34 | 4.6754095559 | 2.6969438704 | -1.9694590761 |
| C35 | 7.4328648458 | 2.7314793529 | -1.4623561983 |
| H36 | 7.1132919448 | 0.8900623193 | -0.3893604556 |
| C37 | 5.4590667765 | 3.7332527860 | -2.4621941448 |
| H38 | 3.6103990753 | 2.6631035941 | -2.1727170646 |
| C39 | 6.8329949784 | 3.7532948530 | -2.2052434808 |
| H40 | 8.5022877782 | 2.7503679596 | -1.2758249877 |
| H41 | 5.0007892755 | 4.5277851731 | -3.0420989658 |
| H42 | 7.4402272621 | 4.5686239481 | -2.5878286016 |
| C43 | 5.3074364184 | -4.5728579537 | -3.4646640342 |
| C44 | 6.7077255214 | -4.4141690169 | -3.4487899259 |
| C45 | 4.6230763733 | -5.4470427267 | -2.5986345185 |
| C46 | 7.4235407110 | -5.1535168231 | -2.5002410171 |
| C47 | 5.3921989252 | -6.1605169526 | -1.6719401403 |
| C48 | 6.7749663195 | -6.0131821422 | -1.6187179662 |
| H49 | 8.5042013028 | -5.0562232836 | -2.4534303757 |
| H50 | 4.9013102290 | -6.8443293031 | -0.9857685502 |
| H51 | 7.3518863532 | -6.5774326485 | -0.8912907962 |
| C52 | 2.7739778449 | -0.9708009843 | -5.6646074045 |
| C53 | 1.4076130849 | -0.8390530114 | -5.3531086143 |
| C54 | 3.5296092795 | 0.0547136495 | -6.2683764564 |
| C55 | 0.8001194207 | 0.3849810895 | -5.6568567100 |
| C56 | 2.8679858052 | 1.2562509945 | -6.5506465579 |
| C57 | 1.5194412047 | 1.4201697241 | -6.2471347989 |
| H58 | -0.2533524178 | 0.5267661110 | -5.4346887472 |

| | | | |
|---|---|---|---|
| H59 | 3.4156063973 | 2.0700330931 | -7.0166246305 |
| H60 | 1.0236802394 | 2.3589506517 | -6.4780013093 |
| C61 | 3.1096938995 | -5.6423858041 | -2.6406927562 |
| H62 | 2.7056223471 | -5.0186335098 | -3.4438776946 |
| C63 | 2.4477024110 | -5.1787531490 | -1.3283545787 |
| H64 | 2.7944682636 | -5.7702265177 | -0.4741363892 |
| H65 | 2.6746611817 | -4.1262059763 | -1.1262704743 |
| H66 | 1.3594775336 | -5.2886777296 | -1.3893828696 |
| C67 | 2.7377292286 | -7.1025468689 | -2.9658781089 |
| H68 | 1.6511005219 | -7.2092224815 | -3.0498829983 |
| H69 | 3.1835162471 | -7.4283945757 | -3.9110457355 |
| H70 | 3.0777616701 | -7.7896349876 | -2.1839859729 |
| C71 | 0.5867000174 | -1.9633822615 | -4.7270992810 |
| H72 | 1.2500450212 | -2.8163469046 | -4.5552865906 |
| C73 | 0.0171088732 | -1.5498788227 | -3.3564827048 |
| H74 | 0.8192387928 | -1.2543275081 | -2.6716123478 |
| H75 | -0.6775914012 | -0.7080935897 | -3.4451709563 |
| H76 | -0.5303278495 | -2.3842412111 | -2.9046061284 |
| C77 | -0.5316343291 | -2.4362335858 | -5.6769862166 |
| H78 | -0.1273419263 | -2.7601642111 | -6.6414603106 |
| H79 | -1.0748383706 | -3.2795127548 | -5.2373617746 |
| H80 | -1.2574108796 | -1.6395931654 | -5.8714898763 |
| C81 | 7.4527567075 | -3.4865179721 | -4.4064272504 |
| H82 | 6.7251364897 | -3.0451118204 | -5.0942644042 |
| C83 | 8.4761148287 | -4.2589988263 | -5.2622429240 |
| H84 | 8.9502279550 | -3.5878474393 | -5.9860650130 |
| H85 | 9.2711319212 | -4.6939644290 | -4.6475822905 |
| H86 | 8.0019947123 | -5.0747100085 | -5.8174418795 |
| C87 | 8.1256887673 | -2.3247908265 | -3.6493672416 |

| | | | |
|---|---|---|---|
| H88 | 7.3909698165 | -1.7459643959 | -3.0799658465 |
| H89 | 8.8838943600 | -2.6905935122 | -2.9487140372 |
| H90 | 8.6227853935 | -1.6486665559 | -4.3535406664 |
| C91 | 5.0081233949 | -0.0971092653 | -6.6187373233 |
| H92 | 5.3331738182 | -1.0964630185 | -6.3139401858 |
| C93 | 5.8743918468 | 0.9141301142 | -5.8426865127 |
| H94 | 5.6314188758 | 1.9458893728 | -6.1184985456 |
| H95 | 5.7332620136 | 0.8127587600 | -4.7619700803 |
| H96 | 6.9352801580 | 0.7555246953 | -6.0655796989 |
| C97 | 5.2423981906 | 0.0147017551 | -8.1385231981 |
| H98 | 6.2992222406 | -0.1483869325 | -8.3751170934 |
| H99 | 4.6543216627 | -0.7259171848 | -8.6905550306 |
| H100 | 4.9672122988 | 1.0048321423 | -8.5171655372 |

**F**

| | | | |
|---|---|---|---|
| C1 | 2.6120704421 | 1.1549659761 | -0.9524778397 |
| C2 | 2.7515372310 | 1.7303139451 | 0.4296778499 |
| C3 | 2.1302988285 | 2.8778385477 | 0.9961511829 |
| C4 | 1.1931956139 | 3.5883667905 | 0.3037769823 |
| H5 | 1.5770866531 | 0.8475758642 | -1.1534934844 |
| H6 | 2.8570206889 | 1.9096585555 | -1.7108103625 |
| H7 | 2.4323286753 | 3.1878366016 | 1.9895377328 |
| H8 | 0.8793223771 | 3.2983782576 | -0.6956347522 |
| C9 | 3.5559251004 | -0.0266826405 | -0.9960424493 |
| C10 | 4.1394896365 | -0.1119990213 | 0.2211609366 |
| O11 | 0.5499100765 | 4.6708731667 | 0.6991993713 |
| C12 | 0.8300693197 | 5.2240116925 | 1.9983741040 |

| | | | |
|---|---|---|---|
| H13 | 0.1901983232 | 6.1001530728 | 2.0877807752 |
| H14 | 0.5854835750 | 4.4992836960 | 2.7811187292 |
| H15 | 1.8818171496 | 5.5180832693 | 2.0677050863 |
| Au16 | 3.8252211834 | -1.2369787571 | -2.6276809876 |
| C17 | 4.0596404513 | -2.4360764564 | -4.2919909825 |
| N18 | 5.1479604178 | -3.1245275246 | -4.7305415586 |
| N19 | 3.0977040909 | -2.6883996748 | -5.2212354391 |
| C20 | 4.8682930118 | -3.7955125507 | -5.9147555458 |
| C21 | 3.5762031974 | -3.5207419650 | -6.2243500500 |
| H22 | 5.6095200001 | -4.3984508307 | -6.4142693919 |
| H23 | 2.9577768262 | -3.8339636370 | -7.0502165969 |
| N24 | 3.6439726760 | 0.9841448901 | 1.0748361374 |
| C25 | 4.0525025189 | 1.2398884296 | 2.4233638467 |
| C26 | 6.4184065845 | 1.8380602767 | 1.8280623957 |
| C27 | 5.2964806332 | 1.5927653015 | 2.7696601741 |
| H28 | 3.2670800126 | 1.1106647965 | 3.1636755207 |
| H29 | 6.1929878238 | 1.7320086132 | 0.7493601745 |
| H30 | 5.5280766622 | 1.7345393735 | 3.8217862209 |
| O31 | 7.5215652525 | 2.1587156100 | 2.2181159228 |
| C32 | 5.1074335811 | -1.0768470310 | 0.7789098245 |
| C33 | 4.8284405652 | -1.7938255238 | 1.9544218962 |
| C34 | 6.3239980386 | -1.3063555580 | 0.1185562705 |
| C35 | 5.7479324908 | -2.7085836929 | 2.4612892034 |
| H36 | 3.8812227013 | -1.6473757868 | 2.4674510445 |
| C37 | 7.2424195190 | -2.2244542110 | 0.6269462473 |
| H38 | 6.5453822688 | -0.7620654894 | -0.7942279763 |
| C39 | 6.9600731246 | -2.9221144303 | 1.8011532631 |
| H40 | 5.5175464837 | -3.2581181664 | 3.3694514185 |
| H41 | 8.1786658297 | -2.3898062841 | 0.1032724163 |

| | | | |
|---|---|---|---|
| H42 | 7.6796423712 | -3.6301051314 | 2.2022528945 |
| C43 | 6.4324830763 | -3.1987621100 | -4.0691165034 |
| C44 | 6.6659494677 | -4.2701617815 | -3.1842643882 |
| C45 | 7.4092152864 | -2.2316190725 | -4.3782665886 |
| C46 | 7.9364748627 | -4.3540441839 | -2.6026067734 |
| C47 | 8.6599522166 | -2.3647565971 | -3.7634377084 |
| C48 | 8.9236951550 | -3.4156577429 | -2.8898487117 |
| H49 | 8.1560235499 | -5.1676444826 | -1.9176792181 |
| H50 | 9.4401111469 | -1.6406437285 | -3.9796395484 |
| H51 | 9.9064852880 | -3.5071292048 | -2.4349477210 |
| C52 | 1.7490312226 | -2.1709624966 | -5.1700020861 |
| C53 | 1.4736798179 | -0.9507200697 | -5.8163051935 |
| C54 | 0.7712685480 | -2.9157213209 | -4.4822158586 |
| C55 | 0.1583438311 | -0.4764210811 | -5.7522693290 |
| C56 | -0.5263418407 | -2.3925947790 | -4.4520004356 |
| C57 | -0.8308603542 | -1.1872456763 | -5.0782954935 |
| H58 | -0.0949040910 | 0.4605294158 | -6.2398905976 |
| H59 | -1.3088198561 | -2.9386623710 | -3.9330942174 |
| H60 | -1.8467218684 | -0.8028648903 | -5.0462951827 |
| C61 | 7.1606952374 | -1.0741438945 | -5.3416837833 |
| H62 | 6.1432367304 | -1.1669615371 | -5.7314606336 |
| C63 | 7.2461506050 | 0.2841288271 | -4.6165420984 |
| H64 | 6.5201251440 | 0.3383076279 | -3.7984613217 |
| H65 | 8.2433957676 | 0.4530859611 | -4.1957698062 |
| H66 | 7.0377794833 | 1.1035817278 | -5.3132497820 |
| C67 | 8.1187017611 | -1.1263399074 | -6.5477697000 |
| H68 | 8.0404600087 | -2.0801415618 | -7.0794643007 |
| H69 | 7.8841015080 | -0.3241336082 | -7.2555810993 |
| H70 | 9.1623937662 | -1.0014439278 | -6.2406070499 |

| | | | |
|---|---|---|---|
| C71 | 5.6000960803 | -5.3058826841 | -2.8338316002 |
| H72 | 4.7214704248 | -5.1165511405 | -3.4570448450 |
| C73 | 5.1555491340 | -5.1626064972 | -1.3642703513 |
| H74 | 5.9896476159 | -5.3394581313 | -0.6775872938 |
| H75 | 4.7661770624 | -4.1597539524 | -1.1630309445 |
| H76 | 4.3698133379 | -5.8899295872 | -1.1312543023 |
| C77 | 6.0706799202 | -6.7409105217 | -3.1403648099 |
| H78 | 5.2645430063 | -7.4549875806 | -2.9413222771 |
| H79 | 6.3680698794 | -6.8502821887 | -4.1883922867 |
| H80 | 6.9251648396 | -7.0309196898 | -2.5201774994 |
| C81 | 2.5407480154 | -0.1453212873 | -6.5551328880 |
| H82 | 3.4802027937 | -0.7045044574 | -6.5152581237 |
| C83 | 2.7905226372 | 1.2120797164 | -5.8677187996 |
| H84 | 1.8907335583 | 1.8370764212 | -5.8779276752 |
| H85 | 3.0971474816 | 1.0720393193 | -4.8255265819 |
| H86 | 3.5834199317 | 1.7629657799 | -6.3854154424 |
| C87 | 2.1852925588 | 0.0391907624 | -8.0427452913 |
| H88 | 2.9925146266 | 0.5638810729 | -8.5644600639 |
| H89 | 2.0315116435 | -0.9241186219 | -8.5393775839 |
| H90 | 1.2716902555 | 0.6289664732 | -8.1708681668 |
| C91 | 1.0770359151 | -4.2376723593 | -3.7807602195 |
| H92 | 2.1334966017 | -4.4723734051 | -3.9392682393 |
| C93 | 0.8617840017 | -4.1280639081 | -2.2576850273 |
| H94 | 1.4806809348 | -3.3333361676 | -1.8280359236 |
| H95 | -0.1844453873 | -3.9128194795 | -2.0146654038 |
| H96 | 1.1286376040 | -5.0703741988 | -1.7669049539 |
| C97 | 0.2591936173 | -5.3992546977 | -4.3789768692 |
| H98 | 0.4339460936 | -5.5012288760 | -5.4549286858 |
| H99 | 0.5366561714 | -6.3444889155 | -3.9005967885 |

| | | | |
|---|---|---|---|
| H100 | -0.8161174619 | -5.2562445368 | -4.2287982318 |

α

| | | | |
|---|---|---|---|
| C1 | -4.1573163975 | -1.7210856139 | -0.0860761656 |
| C2 | -3.0857510087 | -1.1002932462 | -0.0227225518 |
| C3 | -1.6035722717 | -0.9505400566 | 0.0598396778 |
| H4 | -1.1831825954 | -1.7151802647 | -0.6167717832 |
| O5 | -1.2651789316 | 0.3391132795 | -0.3988027113 |
| C6 | 0.0889693370 | 0.4510210786 | -0.8342554097 |
| H7 | 0.2094336943 | 1.4737331431 | -1.1923651533 |
| H8 | 0.7947645415 | 0.2643022681 | -0.0177572608 |
| H9 | 0.2946111508 | -0.2471036739 | -1.6576543253 |
| C10 | -1.1261892534 | -1.2640838316 | 1.4768012247 |
| C11 | -0.7296933694 | -0.3618229316 | 2.3385065379 |
| C12 | -0.3025052024 | 0.5136163418 | 3.2071501394 |
| H13 | -0.9815089083 | 0.9912965679 | 3.9093853734 |
| H14 | 0.7476127791 | 0.7909443793 | 3.2661080570 |
| Au15 | -4.4096271785 | 0.7021125731 | 0.0946969907 |
| C16 | -5.7749569287 | 4.7057518009 | -0.0924226970 |
| C17 | -6.6360317798 | 4.2332610006 | 0.8445208634 |
| H18 | -5.6640156361 | 5.6838886710 | -0.5322026887 |
| H19 | -7.4326153618 | 4.7140731476 | 1.3891370208 |
| C20 | -5.2935849686 | 2.5255831249 | 0.2331279782 |
| N21 | -6.3270791778 | 2.8946219532 | 1.0347302379 |
| N22 | -4.9586372032 | 3.6459364997 | -0.4571920991 |
| C23 | -5.3511156057 | -2.4984678595 | -0.1900573435 |
| C24 | -6.1013913968 | -2.8123250401 | 0.9611853215 |
| C25 | -5.7598203564 | -2.9824933769 | -1.4494593510 |

| | | | |
|---|---|---|---|
| C26 | -7.2405369340 | -3.6012501393 | 0.8470382526 |
| H27 | -5.7832439981 | -2.4335131693 | 1.9271256663 |
| C28 | -6.8996593970 | -3.7726161082 | -1.5488571740 |
| H29 | -5.1766150709 | -2.7380491615 | -2.3317019710 |
| C30 | -7.6399850117 | -4.0806755042 | -0.4040283261 |
| H31 | -7.8185588356 | -3.8450665973 | 1.7330276838 |
| H32 | -7.2124729848 | -4.1490631524 | -2.5179207823 |
| H33 | -8.5303007383 | -4.6973656389 | -0.4871588040 |
| C34 | -7.0436997654 | 2.0335657510 | 1.9516346831 |
| C35 | -8.2140766060 | 1.3978101249 | 1.4916956576 |
| C36 | -6.5661030685 | 1.9118394732 | 3.2703059347 |
| C37 | -8.9082197389 | 0.6005811753 | 2.4089188875 |
| C38 | -7.3000176814 | 1.0977391700 | 4.1405076275 |
| C39 | -8.4561294592 | 0.4494667899 | 3.7165378633 |
| H40 | -9.8185770224 | 0.0979209782 | 2.0956021959 |
| H41 | -6.9672012419 | 0.9788385900 | 5.1672462065 |
| H42 | -9.0145205495 | -0.1696987052 | 4.4131242170 |
| C43 | -3.9065960969 | 3.7493062410 | -1.4468443982 |
| C44 | -2.6316577959 | 4.1735789568 | -1.0244224120 |
| C45 | -4.2221830668 | 3.4662329438 | -2.7901233543 |
| C46 | -1.6457106003 | 4.3023050144 | -2.0094591932 |
| C47 | -3.1931065650 | 3.6065468211 | -3.7282826677 |
| C48 | -1.9206786446 | 4.0193063717 | -3.3443282362 |
| H49 | -0.6520771897 | 4.6375302398 | -1.7273220352 |
| H50 | -3.3942528405 | 3.3988504171 | -4.7749861647 |
| H51 | -1.1403936375 | 4.1297099887 | -4.0921501144 |
| C52 | -5.3218141779 | 2.6376145383 | 3.7750400034 |
| H53 | -4.8962023253 | 3.2086013162 | 2.9443634132 |
| C54 | -5.6762201073 | 3.6438298393 | 4.8884169144 |

| | | | |
|---|---|---|---|
| H55 | -6.0810983567 | 3.1397554253 | 5.7722450926 |
| H56 | -4.7839051730 | 4.1959404447 | 5.2017199372 |
| H57 | -6.4218205185 | 4.3704334099 | 4.5497525699 |
| C58 | -4.2398568790 | 1.6460702101 | 4.2447388115 |
| H59 | -3.9479305781 | 0.9657375849 | 3.4378958533 |
| H60 | -3.3479813788 | 2.1908176561 | 4.5735964458 |
| H61 | -4.5860485157 | 1.0401985508 | 5.0886453900 |
| C62 | -8.7489154564 | 1.5636243516 | 0.0714908884 |
| H63 | -8.0389582869 | 2.1754289675 | -0.4927491037 |
| C64 | -8.8565499546 | 0.2116544282 | -0.6592136438 |
| H65 | -9.5818017947 | -0.4527195645 | -0.1778440711 |
| H66 | -9.1873463135 | 0.3659285669 | -1.6919730878 |
| H67 | -7.8918448297 | -0.3052288187 | -0.6830973411 |
| C68 | -10.0990163749 | 2.3090809737 | 0.0696378182 |
| H69 | -10.8693939487 | 1.7440138000 | 0.6049833539 |
| H70 | -10.0172291796 | 3.2911487913 | 0.5471283102 |
| H71 | -10.4502853802 | 2.4606909605 | -0.9564384736 |
| C72 | -2.3112973230 | 4.5238342626 | 0.4262520732 |
| H73 | -3.1803560185 | 4.2657412131 | 1.0389311057 |
| C74 | -1.1224550631 | 3.7088236198 | 0.9684540635 |
| H75 | -0.1925329373 | 3.9546959487 | 0.4437248593 |
| H76 | -0.9650867569 | 3.9357010612 | 2.0285943802 |
| H77 | -1.2958904036 | 2.6340650433 | 0.8686335746 |
| C78 | -2.0687176824 | 6.0381476122 | 0.5860593306 |
| H79 | -1.1981789113 | 6.3641166044 | 0.0072274790 |
| H80 | -2.9297002269 | 6.6241150386 | 0.2472656005 |
| H81 | -1.8829154254 | 6.2864678656 | 1.6361856262 |
| C82 | -5.6166958727 | 3.0454072084 | -3.2483218163 |
| H83 | -6.2613163057 | 2.9737003654 | -2.3666991657 |

| | | | |
|---|---|---|---|
| C84 | -5.5997348837 | 1.6539284108 | -3.9095747177 |
| H85 | -5.1888742160 | 0.8991401989 | -3.2305901741 |
| H86 | -6.6161811382 | 1.3514791141 | -4.1836650909 |
| H87 | -4.9960291866 | 1.6481376379 | -4.8230552642 |
| C88 | -6.2388041268 | 4.1001762578 | -4.1856835706 |
| H89 | -5.6595609366 | 4.2082637510 | -5.1087226646 |
| H90 | -7.2569871783 | 3.8094442236 | -4.4653972804 |
| H91 | -6.2871978550 | 5.0838113158 | -3.7071968783 |
| H92 | -1.1343220949 | -2.3184847506 | 1.7544619839 |

**αβ-TS**

| | | | |
|---|---|---|---|
| C1 | -3.7825245621 | -2.0473162221 | -0.1696722702 |
| C2 | -3.1693094292 | -0.9844877634 | 0.2420182368 |
| N3 | -2.6533038679 | -3.6315201993 | -0.0604307382 |
| C4 | -2.1601894836 | -4.4519290826 | -0.9623186993 |
| C5 | -2.1611288899 | -5.5362133112 | 0.9358406583 |
| C6 | -1.8160538560 | -5.6928896543 | -0.3728881322 |
| H7 | -2.0741791666 | -4.1286962790 | -1.9912887858 |
| H8 | -2.1059035297 | -6.1726795906 | 1.8071553399 |
| H9 | -1.3826700393 | -6.5574864333 | -0.8510669180 |
| O10 | -2.6719440930 | -4.3082737114 | 1.1455541230 |
| C11 | -1.7473123924 | -0.8648085813 | 0.7477923121 |
| H12 | -1.1442020623 | -1.6430226512 | 0.2483350351 |
| O13 | -1.2575265626 | 0.4103934644 | 0.3848360153 |
| C14 | 0.1609279401 | 0.4861615039 | 0.3120273463 |
| H15 | 0.3989247976 | 1.5055695297 | 0.0038886212 |
| H16 | 0.6364433608 | 0.2821563007 | 1.2785901310 |

| | | | |
|---|---|---|---|
| H17 | 0.5567894299 | -0.2156584175 | -0.4369867834 |
| C18 | -1.6949375190 | -1.1380963146 | 2.2490948057 |
| C19 | -1.3606909234 | -0.2639979138 | 3.1641689095 |
| C20 | -1.0050567870 | 0.5795785292 | 4.0961065481 |
| H21 | -1.7341910773 | 1.2128596053 | 4.5960253253 |
| H22 | 0.0324119757 | 0.6797577846 | 4.4082585702 |
| Au23 | -4.3881387972 | 0.7381009176 | 0.1976404365 |
| C24 | -6.0786374514 | 4.6479973476 | 0.0336771893 |
| C25 | -7.0652008011 | 4.0174589896 | 0.7185759753 |
| H26 | -5.9699275877 | 5.6737520491 | -0.2799644803 |
| H27 | -7.9956788639 | 4.3791199462 | 1.1257551891 |
| C28 | -5.4729082230 | 2.4770965461 | 0.2486911787 |
| N29 | -6.6790625904 | 2.6896854840 | 0.8418788783 |
| N30 | -5.1114954965 | 3.6921283473 | -0.2455761248 |
| C31 | -4.9795722612 | -2.6295194648 | -0.7512830751 |
| C32 | -5.9121438648 | -3.3156806889 | 0.0458915921 |
| C33 | -5.1873717884 | -2.5406740776 | -2.1401526489 |
| C34 | -7.0338676797 | -3.8968277955 | -0.5385691576 |
| H35 | -5.7512501589 | -3.3832663096 | 1.1174379225 |
| C36 | -6.3121063238 | -3.1240800530 | -2.7179189382 |
| H37 | -4.4691677785 | -2.0023396599 | -2.7519659300 |
| C38 | -7.2331372839 | -3.8053553787 | -1.9185811746 |
| H39 | -7.7555226171 | -4.4208483420 | 0.0809389263 |
| H40 | -6.4718200995 | -3.0464294314 | -3.7894193011 |
| H41 | -8.1082475643 | -4.2626705592 | -2.3715305179 |
| C42 | -3.8953343361 | 3.9845273126 | -0.9730199645 |
| C43 | -2.7700553173 | 4.4274987856 | -0.2511959649 |
| C44 | -3.9081506950 | 3.8625391778 | -2.3761064689 |
| C45 | -1.6241590571 | 4.7493577570 | -0.9876567925 |

| | | | |
|---|---|---|---|
| C46 | -2.7318222786 | 4.1931900680 | -3.0584717015 |
| C47 | -1.6027410950 | 4.6325035866 | -2.3740049765 |
| H48 | -0.7384179719 | 5.1011229047 | -0.4669960201 |
| H49 | -2.7025205544 | 4.1119422473 | -4.1411069232 |
| H50 | -0.7027657226 | 4.8912008102 | -2.9251219305 |
| C51 | -7.4832089848 | 1.6798427474 | 1.4934022230 |
| C52 | -8.4813015288 | 1.0350850081 | 0.7367904533 |
| C53 | -7.2569438872 | 1.4104474534 | 2.8567734437 |
| C54 | -9.2614297225 | 0.0762112412 | 1.3923794577 |
| C55 | -8.0661259397 | 0.4393426685 | 3.4581088682 |
| C56 | -9.0555510821 | -0.2210738063 | 2.7362817545 |
| H57 | -10.0427456766 | -0.4410416773 | 0.8431377901 |
| H58 | -7.9244471983 | 0.2041417818 | 4.5088603357 |
| H59 | -9.6758815615 | -0.9665145330 | 3.2264078626 |
| C60 | -8.7371053137 | 1.3453034246 | -0.7362971160 |
| H61 | -8.0291034718 | 2.1170575725 | -1.0522405218 |
| C62 | -8.4830120184 | 0.1110752816 | -1.6223539606 |
| H63 | -7.4619098141 | -0.2620523927 | -1.4980071615 |
| H64 | -9.1714409173 | -0.7060029513 | -1.3814911345 |
| H65 | -8.6288960962 | 0.3657741725 | -2.6781411196 |
| C66 | -10.1547269398 | 1.9090899852 | -0.9568296283 |
| H67 | -10.3283192700 | 2.8064964034 | -0.3540526430 |
| H68 | -10.2997001686 | 2.1758162393 | -2.0092235070 |
| H69 | -10.9255371744 | 1.1775083199 | -0.6920097820 |
| C70 | -6.2051123616 | 2.1420619409 | 3.6864218255 |
| H71 | -5.6667175818 | 2.8295688308 | 3.0273339364 |
| C72 | -5.1644252493 | 1.1682938913 | 4.2719351061 |
| H73 | -5.6250971527 | 0.4456878523 | 4.9542103991 |
| H74 | -4.6541533590 | 0.6099665730 | 3.4805158422 |

| | | | |
|---|---|---|---|
| H75 | -4.4101105634 | 1.7224402687 | 4.8419222976 |
| C76 | -6.8632188572 | 2.9888919752 | 4.7946561126 |
| H77 | -6.1032659028 | 3.5511385116 | 5.3478646767 |
| H78 | -7.5790551100 | 3.7061651823 | 4.3798549571 |
| H79 | -7.4006249993 | 2.3615628804 | 5.5139088746 |
| C80 | -2.7671219179 | 4.5876916372 | 1.2661392165 |
| H81 | -3.7146966728 | 4.1992700003 | 1.6518671221 |
| C82 | -2.6814224518 | 6.0747189992 | 1.6643993701 |
| H83 | -1.7467595080 | 6.5267174594 | 1.3155845911 |
| H84 | -3.5081630621 | 6.6546891450 | 1.2405241241 |
| H85 | -2.7170876363 | 6.1821080565 | 2.7537059559 |
| C86 | -1.6424487007 | 3.7661271448 | 1.9234361980 |
| H87 | -0.6522295829 | 4.1362963940 | 1.6346913952 |
| H88 | -1.7103069164 | 3.8414937649 | 3.0144505026 |
| H89 | -1.7045270504 | 2.7100419235 | 1.6468887476 |
| C90 | -5.1350002036 | 3.4013085076 | -3.1584373959 |
| H91 | -5.9401614426 | 3.1988330412 | -2.4459152220 |
| C92 | -5.6370287735 | 4.4989495727 | -4.1174426147 |
| H93 | -4.8924090809 | 4.7349296650 | -4.8850926293 |
| H94 | -6.5480366812 | 4.1718587052 | -4.6300744902 |
| H95 | -5.8652968714 | 5.4256398996 | -3.5808864466 |
| C96 | -4.8572026039 | 2.0860003761 | -3.9116977510 |
| H97 | -4.0710507097 | 2.2120433017 | -4.6638226335 |
| H98 | -4.5404161929 | 1.2981473822 | -3.2203339170 |
| H99 | -5.7606888312 | 1.7463594120 | -4.4301509562 |
| H100 | -1.9571684451 | -2.1528493452 | 2.5474015632 |

β

| | | | |
|---|---|---|---|
| C1 | -3.9640419022 | -2.2043075183 | 0.7696878580 |
| C2 | -3.6420150570 | -1.1444505513 | -0.0153435646 |
| N3 | -3.5131638983 | -3.5310706251 | 0.3901485201 |
| C4 | -3.8554657443 | -4.3671270796 | -0.5732076598 |
| C5 | -2.3442454039 | -5.3690195409 | 0.6602924642 |
| C6 | -3.1209999272 | -5.5629457261 | -0.4476528053 |
| H7 | -4.6059418397 | -4.0721727534 | -1.2936967259 |
| H8 | -1.6256489204 | -5.9857378470 | 1.1821300896 |
| H9 | -3.1745894358 | -6.4369728704 | -1.0778559058 |
| O10 | -2.5474720299 | -4.1547025444 | 1.1881225261 |
| C11 | -2.6654461765 | -1.2978297069 | -1.1727540255 |
| H12 | -2.4828760093 | -2.3566756238 | -1.4287717150 |
| O13 | -3.2239048898 | -0.6572529963 | -2.3061666985 |
| C14 | -2.6278718303 | -1.0397881658 | -3.5382206535 |
| H15 | -3.1640223789 | -0.5034328718 | -4.3235526052 |
| H16 | -1.5663515561 | -0.7730692501 | -3.5837478377 |
| H17 | -2.7291942870 | -2.1224058029 | -3.7134243531 |
| C18 | -1.3162926324 | -0.7278074265 | -0.7363530708 |
| C19 | -0.7773352067 | 0.3615044551 | -1.2200795213 |
| C20 | -0.2285680712 | 1.4389051202 | -1.7150270384 |
| H21 | -0.4340427854 | 2.4206719174 | -1.2931749909 |
| H22 | 0.4399065459 | 1.3923340284 | -2.5729664211 |
| Au23 | -4.4974724489 | 0.7299688283 | 0.2347282792 |
| C24 | -5.9901564474 | 4.7529642723 | 0.0201950645 |
| C25 | -7.1563348755 | 4.0640423225 | 0.0191214208 |
| H26 | -5.7826741384 | 5.8084629781 | -0.0500354002 |
| H27 | -8.1804181868 | 4.3943681944 | -0.0492512444 |

| | | | |
|---|---|---|---|
| C28 | -5.4768226761 | 2.5531390114 | 0.2141171599 |
| N29 | -6.8277159790 | 2.7204498110 | 0.1423130769 |
| N30 | -4.9714606523 | 3.8135015606 | 0.1335103406 |
| C31 | -4.7015405192 | -2.2525689150 | 2.0624496937 |
| C32 | -5.7633139671 | -3.1509648194 | 2.2465026046 |
| C33 | -4.2917228234 | -1.4567014718 | 3.1423385646 |
| C34 | -6.4115500722 | -3.2432156326 | 3.4755119852 |
| H35 | -6.0927965786 | -3.7792250387 | 1.4221910568 |
| C36 | -4.9354220253 | -1.5572241055 | 4.3774811164 |
| H37 | -3.4551836690 | -0.7764042419 | 3.0113001864 |
| C38 | -5.9939733488 | -2.4516241260 | 4.5471157126 |
| H39 | -7.2375002250 | -3.9375422953 | 3.6005089321 |
| H40 | -4.5984923879 | -0.9464794835 | 5.2112231404 |
| H41 | -6.4903394128 | -2.5333499211 | 5.5100806162 |
| C42 | -3.5724693127 | 4.1805484064 | 0.1384014765 |
| C43 | -2.9025041080 | 4.2811489907 | 1.3729037560 |
| C44 | -2.9673185048 | 4.5115672328 | -1.0925567963 |
| C45 | -1.5770792006 | 4.7317209279 | 1.3492798180 |
| C46 | -1.6497245358 | 4.9806291675 | -1.0496268353 |
| C47 | -0.9595357373 | 5.0930717451 | 0.1553449872 |
| H48 | -1.0264484600 | 4.8121759367 | 2.2811285842 |
| H49 | -1.1538037441 | 5.2580107896 | -1.9754418116 |
| H50 | 0.0627755212 | 5.4616111373 | 0.1644453429 |
| C51 | -7.7895803092 | 1.6459494163 | 0.2366516165 |
| C52 | -8.1252510516 | 0.9383424072 | -0.9336136146 |
| C53 | -8.3476192720 | 1.3449050104 | 1.4973303199 |
| C54 | -9.0253407844 | -0.1251432788 | -0.8046537108 |
| C55 | -9.2366984955 | 0.2678814292 | 1.5635876973 |
| C56 | -9.5675253845 | -0.4674508858 | 0.4289017194 |

| | | | |
|---|---|---|---|
| H57 | -9.3079772775 | -0.6902476095 | -1.6880442430 |
| H58 | -9.6793809474 | 0.0013297984 | 2.5186918753 |
| H59 | -10.2659049977 | -1.2966297705 | 0.5029391709 |
| C60 | -7.5737122000 | 1.2943560316 | -2.3120483646 |
| H61 | -6.8105833068 | 2.0676854477 | -2.1850569874 |
| C62 | -8.6858086401 | 1.8835040005 | -3.2047930335 |
| H63 | -8.2789384948 | 2.1815021573 | -4.1770759953 |
| H64 | -9.4804253535 | 1.1513082595 | -3.3848030796 |
| H65 | -9.1452714882 | 2.7653834482 | -2.7459170244 |
| C66 | -6.8898372880 | 0.0958185165 | -2.9980840827 |
| H67 | -6.0580991927 | -0.2807999802 | -2.3952563677 |
| H68 | -7.5933001567 | -0.7262362502 | -3.1738596638 |
| H69 | -6.4903517090 | 0.3987125084 | -3.9718703646 |
| C70 | -7.9890231380 | 2.1046501748 | 2.7748351529 |
| H71 | -7.3327503587 | 2.9373740501 | 2.5097294920 |
| C72 | -7.2091776057 | 1.2084707712 | 3.7550443854 |
| H73 | -7.8067421916 | 0.3463458667 | 4.0701466480 |
| H74 | -6.2954037225 | 0.8233249563 | 3.2932237296 |
| H75 | -6.9340626846 | 1.7720273874 | 4.6537844480 |
| C76 | -9.2351991371 | 2.7136754282 | 3.4467853761 |
| H77 | -8.9423598723 | 3.3107081769 | 4.3169754556 |
| H78 | -9.7845923560 | 3.3656320592 | 2.7599269447 |
| H79 | -9.9280462471 | 1.9412327816 | 3.7969133194 |
| C80 | -3.5519677300 | 3.9141312354 | 2.7043194772 |
| H81 | -4.5920883513 | 3.6341958429 | 2.5126345476 |
| C82 | -2.8634850321 | 2.6847740284 | 3.3284885992 |
| H83 | -1.8109162758 | 2.8853706933 | 3.5538922464 |
| H84 | -3.3581722587 | 2.4019362653 | 4.2647092681 |
| H85 | -2.9069867520 | 1.8314157949 | 2.6435307038 |

| | | | |
|---|---|---|---|
| C86 | -3.5693791747 | 5.1047474552 | 3.6824877490 |
| H87 | -2.5575540888 | 5.4131040177 | 3.9660990786 |
| H88 | -4.0718616690 | 5.9749771841 | 3.2474243599 |
| H89 | -4.0993793554 | 4.8320085182 | 4.6016006577 |
| C90 | -3.6684771817 | 4.3586874992 | -2.4425436495 |
| H91 | -4.6944247740 | 4.0271100219 | -2.2630492511 |
| C92 | -2.9837192133 | 3.2668206926 | -3.2928272441 |
| H93 | -2.9484194702 | 2.3107612531 | -2.7618318860 |
| H94 | -3.5312015049 | 3.1231814665 | -4.2315691211 |
| H95 | -1.9558927270 | 3.5466342017 | -3.5487638040 |
| C96 | -3.7483033378 | 5.6946990395 | -3.2069297082 |
| H97 | -4.3123634130 | 5.5671852451 | -4.1370467186 |
| H98 | -4.2451185898 | 6.4695611200 | -2.6136312961 |
| H99 | -2.7548578468 | 6.0694741022 | -3.4756274355 |
| H100 | -0.8004526896 | -1.2888438455 | 0.0438108436 |

**βγ-TS**

| | | | |
|---|---|---|---|
| C1 | -3.7419663688 | -2.2188357733 | -0.1117820865 |
| C2 | -3.2777090361 | -0.9472189103 | 0.2524571876 |
| N3 | -2.8318053984 | -3.1218889761 | -0.5565383588 |
| C4 | -2.4327527836 | -3.6872475231 | -1.6742903543 |
| C5 | -1.5903681016 | -5.1254841852 | -0.1846698661 |
| C6 | -1.6738749512 | -4.8513365979 | -1.5420749037 |
| H7 | -2.6979675646 | -3.1995606782 | -2.6078839280 |
| H8 | -1.0426614193 | -5.9472848596 | 0.2791922962 |
| H9 | -1.2289292316 | -5.4133810515 | -2.3479982907 |
| O10 | -2.2035000074 | -4.3079245194 | 0.5967229556 |
| C11 | -1.8085317778 | -0.7583741616 | 0.5177255489 |

| | | | |
|---|---|---|---|
| H12 | -1.1903328360 | -1.4184486049 | -0.1136357057 |
| O13 | -1.4598683563 | 0.5864806184 | 0.2909314134 |
| C14 | -0.0571189905 | 0.8245194335 | 0.2543103954 |
| H15 | 0.0665614129 | 1.8852320910 | 0.0317895335 |
| H16 | 0.4241539326 | 0.5945841374 | 1.2125260278 |
| H17 | 0.4259171294 | 0.2327539090 | -0.5373715346 |
| C18 | -1.6249947927 | -1.2172221237 | 1.9751283107 |
| C19 | -1.5663387788 | -0.4070154350 | 3.0038024864 |
| C20 | -1.4618053229 | 0.3750908593 | 4.0440373211 |
| H21 | -2.3352785052 | 0.6692555910 | 4.6216339157 |
| H22 | -0.4979269853 | 0.7574032893 | 4.3736875607 |
| Au23 | -4.4557335338 | 0.7351028559 | 0.3219807280 |
| C24 | -6.0093897554 | 4.6938192846 | -0.1149104849 |
| C25 | -7.1026160798 | 4.1170300126 | 0.4433324866 |
| H26 | -5.8245228134 | 5.7029956125 | -0.4462495739 |
| H27 | -8.0691433907 | 4.5183718895 | 0.7024268435 |
| C28 | -5.5091528199 | 2.5155133333 | 0.2633660919 |
| N29 | -6.7799471733 | 2.7858284555 | 0.6668134188 |
| N30 | -5.0441109063 | 3.7029164399 | -0.2157375312 |
| C31 | -5.1540062649 | -2.6830704473 | -0.0096349371 |
| C32 | -5.7056972507 | -3.5614948129 | -0.9555595857 |
| C33 | -5.9452349375 | -2.2760976424 | 1.0761000593 |
| C34 | -7.0208878967 | -4.0020856958 | -0.8324977013 |
| H35 | -5.1137747789 | -3.9039382932 | -1.8000467541 |
| C36 | -7.2591552075 | -2.7229997379 | 1.2010410403 |
| H37 | -5.5230402334 | -1.6188509268 | 1.8291713677 |
| C38 | -7.8012672713 | -3.5848835475 | 0.2476648712 |
| H39 | -7.4361449530 | -4.6736370291 | -1.5783639791 |
| H40 | -7.8566543709 | -2.3957940980 | 2.0461182396 |

| | | | |
|---|---|---|---|
| H41 | -8.8240187586 | -3.9369941530 | 0.3477911747 |
| C42 | -3.7259557644 | 3.9472475167 | -0.7585429296 |
| C43 | -2.7421261487 | 4.4823054519 | 0.0954581712 |
| C44 | -3.5037580791 | 3.7000065418 | -2.1270291392 |
| C45 | -1.4936682051 | 4.7703853590 | -0.4684283570 |
| C46 | -2.2327688943 | 3.9964832734 | -2.6320575346 |
| C47 | -1.2396636814 | 4.5291997088 | -1.8155345161 |
| H48 | -0.7124666434 | 5.1936481957 | 0.1563282011 |
| H49 | -2.0223391058 | 3.8198651872 | -3.6827853409 |
| H50 | -0.2645987969 | 4.7650073306 | -2.2330880808 |
| C51 | -7.7148145700 | 1.8475852526 | 1.2443472648 |
| C52 | -8.6463633547 | 1.2267216136 | 0.3895199799 |
| C53 | -7.7055568098 | 1.6591192128 | 2.6398243173 |
| C54 | -9.6033854412 | 0.3957342697 | 0.9825639045 |
| C55 | -8.6819151760 | 0.8109001887 | 3.1760451675 |
| C56 | -9.6254402128 | 0.1933287155 | 2.3597482053 |
| H57 | -10.3436139692 | -0.0947617714 | 0.3573095173 |
| H58 | -8.7129029420 | 0.6450024195 | 4.2489597099 |
| H59 | -10.3860064617 | -0.4454325584 | 2.8006506452 |
| C60 | -8.6445390306 | 1.4291290107 | -1.1237944651 |
| H61 | -7.8181479545 | 2.0992741580 | -1.3782716094 |
| C62 | -9.9443882422 | 2.1014896700 | -1.6082274383 |
| H63 | -9.9028126966 | 2.2783163762 | -2.6883281538 |
| H64 | -10.8207924911 | 1.4754304880 | -1.4092065231 |
| H65 | -10.1065611773 | 3.0654572767 | -1.1146158966 |
| C66 | -8.3915943543 | 0.1011191538 | -1.8633454772 |
| H67 | -7.4533722304 | -0.3593312637 | -1.5386841424 |
| H68 | -9.1973257853 | -0.6188658622 | -1.6854514981 |
| H69 | -8.3355296044 | 0.2741243271 | -2.9437064302 |

| | | | |
|---|---|---|---|
| C70 | -6.7041738466 | 2.3467093484 | 3.5644287183 |
| H71 | -6.0432727309 | 2.9675302591 | 2.9524959755 |
| C72 | -5.8138226637 | 1.3192039213 | 4.2906452139 |
| H73 | -6.4039389085 | 0.6544343999 | 4.9306894858 |
| H74 | -5.2628878879 | 0.7003727447 | 3.5742816589 |
| H75 | -5.0863316034 | 1.8329524655 | 4.9291827378 |
| C76 | -7.4111235099 | 3.2801374515 | 4.5669757582 |
| H77 | -6.6740876849 | 3.8078247471 | 5.1816738493 |
| H78 | -8.0215862040 | 4.0299330150 | 4.0534410309 |
| H79 | -8.0676879800 | 2.7232236366 | 5.2436638225 |
| C80 | -2.9859222290 | 4.7567637354 | 1.5769210168 |
| H81 | -3.9972246854 | 4.4205836502 | 1.8249144854 |
| C82 | -2.0137857328 | 3.9514436421 | 2.4612604652 |
| H83 | -0.9778728587 | 4.2782786241 | 2.3180430730 |
| H84 | -2.2577077359 | 4.0952051687 | 3.5198107617 |
| H85 | -2.0646265477 | 2.8818442084 | 2.2369595179 |
| C86 | -2.9114944162 | 6.2640483963 | 1.8920714450 |
| H87 | -1.9149875128 | 6.6683704718 | 1.6840760601 |
| H88 | -3.6316645229 | 6.8368709890 | 1.2982298118 |
| H89 | -3.1277129778 | 6.4441715825 | 2.9505786923 |
| C90 | -4.5808022391 | 3.1501177777 | -3.0586515749 |
| H91 | -5.4930819308 | 2.9960649913 | -2.4748836777 |
| C92 | -4.1779590401 | 1.7797962234 | -3.6374225861 |
| H93 | -3.9848330611 | 1.0588918100 | -2.8360223066 |
| H94 | -4.9796332936 | 1.3838727991 | -4.2706227072 |
| H95 | -3.2747012461 | 1.8537523643 | -4.2526771286 |
| C96 | -4.9182954640 | 4.1518941268 | -4.1807418517 |
| H97 | -5.7316790270 | 3.7665169590 | -4.8048052109 |
| H98 | -5.2331669415 | 5.1181115005 | -3.7734608118 |

| | | | |
|---|---|---|---|
| H99 | -4.0573654723 | 4.3312338734 | -4.8334516787 |
| H100 | -1.5593101026 | -2.2939408291 | 2.1242588605 |

γ

| | | | |
|---|---|---|---|
| C1 | -3.5512564120 | -2.1248779274 | -0.3721970149 |
| C2 | -3.0869608214 | -0.7906637614 | 0.1566118542 |
| N3 | -2.7103972966 | -2.7351685401 | -1.1272793968 |
| C4 | -2.7700666796 | -3.9587132257 | -1.7384293635 |
| C5 | -2.0964143709 | -5.3424309281 | 0.2262645062 |
| C6 | -2.4546374629 | -5.1548404603 | -1.1749445593 |
| H7 | -2.9503459114 | -3.9122844793 | -2.8140326595 |
| H8 | -1.8614869268 | -6.3893896290 | 0.5060328027 |
| H9 | -2.4321413234 | -6.0282934586 | -1.8191101358 |
| O10 | -2.0270827892 | -4.4588944201 | 1.0761179844 |
| C11 | -1.6537934319 | -0.6463138721 | 0.2608350257 |
| H12 | -1.0452646826 | -1.3485728133 | -0.3192412502 |
| O13 | -1.1656505843 | 0.6397107939 | 0.3035306755 |
| C14 | 0.2426170610 | 0.7173043590 | 0.5357393125 |
| H15 | 0.5044257555 | 1.7735767093 | 0.4734946101 |
| H16 | 0.5054353348 | 0.3324893108 | 1.5292763568 |
| H17 | 0.7996995925 | 0.1576623969 | -0.2262215222 |
| C18 | -1.9489476115 | -1.3503821092 | 1.6533346924 |
| C19 | -2.1350625152 | -0.6568175806 | 2.7663137317 |
| C20 | -2.2556155849 | 0.0012914641 | 3.8797018154 |
| H21 | -3.2305852157 | 0.2079748763 | 4.3148270345 |
| H22 | -1.3788544878 | 0.3646649440 | 4.4123520934 |
| Au23 | -4.3436292012 | 0.8337514790 | 0.3308913750 |

| | | | |
|---|---|---|---|
| C24 | -6.1096621895 | 4.6906970400 | -0.0472357250 |
| C25 | -7.1749936637 | 4.0563290224 | 0.5039437064 |
| H26 | -5.9735731912 | 5.7108793566 | -0.3684179216 |
| H27 | -8.1597580826 | 4.4094343730 | 0.7645794390 |
| C28 | -5.5108827628 | 2.5350142351 | 0.3028897455 |
| N29 | -6.7937433449 | 2.7374983659 | 0.7109515862 |
| N30 | -5.1011967870 | 3.7467673197 | -0.1638990921 |
| C31 | -4.9115947457 | -2.6239875644 | -0.0358276522 |
| C32 | -5.6519038068 | -3.3650848938 | -0.9716237273 |
| C33 | -5.4696721654 | -2.3824127311 | 1.2299751487 |
| C34 | -6.9133194632 | -3.8571138504 | -0.6476756621 |
| H35 | -5.2546940074 | -3.5404412137 | -1.9658786731 |
| C36 | -6.7240413186 | -2.8907564331 | 1.5580452006 |
| H37 | -4.9126547844 | -1.8127444996 | 1.9652812750 |
| C38 | -7.4488622663 | -3.6277791391 | 0.6209007943 |
| H39 | -7.4785005463 | -4.4178254990 | -1.3863469843 |
| H40 | -7.1384999939 | -2.7047268540 | 2.5439915071 |
| H41 | -8.4297490446 | -4.0188505585 | 0.8754971161 |
| C42 | -3.8142913551 | 4.0293741806 | -0.7653529718 |
| C43 | -2.7741737457 | 4.5012907258 | 0.0569963595 |
| C44 | -3.6784304816 | 3.8594341969 | -2.1578923362 |
| C45 | -1.5556557869 | 4.8006493272 | -0.5630928301 |
| C46 | -2.4353082824 | 4.1693862925 | -2.7204577642 |
| C47 | -1.3858803620 | 4.6350359095 | -1.9342338375 |
| H48 | -0.7304562225 | 5.1738767220 | 0.0363859024 |
| H49 | -2.2899669577 | 4.0511112171 | -3.7901881309 |
| H50 | -0.4319243087 | 4.8766684524 | -2.3947711592 |
| C51 | -7.7018770792 | 1.7415300297 | 1.2412317421 |
| C52 | -8.5348831872 | 1.0514416975 | 0.3373244143 |

| | | | |
|---|---|---|---|
| C53 | -7.7772797051 | 1.5694314164 | 2.6370732205 |
| C54 | -9.4635019217 | 0.1537528044 | 0.8786235310 |
| C55 | -8.7241264677 | 0.6584098457 | 3.1221500439 |
| C56 | -9.5593809051 | -0.0390943199 | 2.2538702589 |
| H57 | -10.1268590659 | -0.3912074475 | 0.2138537035 |
| H58 | -8.8179522830 | 0.5052637177 | 4.1931216910 |
| H59 | -10.2962956520 | -0.7309297379 | 2.6524674658 |
| C60 | -8.4803129278 | 1.2689033250 | -1.1728462405 |
| H61 | -7.6518137695 | 1.9490859208 | -1.3911323045 |
| C62 | -9.7715445919 | 1.9392554210 | -1.6842930702 |
| H63 | -9.7051948481 | 2.1248023138 | -2.7615380011 |
| H64 | -10.6479064985 | 1.3063661015 | -1.5086734661 |
| H65 | -9.9507689486 | 2.8985806145 | -1.1873637637 |
| C66 | -8.1943511684 | -0.0427469979 | -1.9276679094 |
| H67 | -7.2679953736 | -0.5086161994 | -1.5780700559 |
| H68 | -9.0040316549 | -0.7689071407 | -1.7998247206 |
| H69 | -8.0969705758 | 0.1538746980 | -3.0009329516 |
| C70 | -6.8968644087 | 2.3438573072 | 3.6135149700 |
| H71 | -6.1978937874 | 2.9526251213 | 3.0329099556 |
| C72 | -6.0526219615 | 1.3927503403 | 4.4827147190 |
| H73 | -6.6772716788 | 0.7768985094 | 5.1381474454 |
| H74 | -5.4614548072 | 0.7193185526 | 3.8523369273 |
| H75 | -5.3691434321 | 1.9644145269 | 5.1204461801 |
| C76 | -7.7315062305 | 3.3033799151 | 4.4846409369 |
| H77 | -7.0817477963 | 3.8774463920 | 5.1536763860 |
| H78 | -8.2946472408 | 4.0134236053 | 3.8702316268 |
| H79 | -8.4507789569 | 2.7584339218 | 5.1050138428 |
| C80 | -2.9294935582 | 4.7012130360 | 1.5618935339 |
| H81 | -3.9342143991 | 4.3753899531 | 1.8483269972 |

| | | | |
|---|---|---|---|
| C82 | -1.9296077748 | 3.8315880396 | 2.3485183204 |
| H83 | -0.8956275553 | 4.1391516620 | 2.1568989533 |
| H84 | -2.1057786786 | 3.9312298272 | 3.4254229280 |
| H85 | -2.0237385248 | 2.7760492975 | 2.0756811031 |
| C86 | -2.7994874204 | 6.1886835145 | 1.9463328319 |
| H87 | -1.8055589573 | 6.5796770319 | 1.7037529250 |
| H88 | -3.5353229552 | 6.8057064293 | 1.4203017616 |
| H89 | -2.9542139836 | 6.3214902367 | 3.0224066557 |
| C90 | -4.8148585227 | 3.3724751905 | -3.0544804510 |
| H91 | -5.6930731428 | 3.1889353994 | -2.4282427750 |
| C92 | -4.4605752664 | 2.0386116437 | -3.7406822554 |
| H93 | -4.2133631012 | 1.2681190174 | -3.0029532967 |
| H94 | -5.3067015503 | 1.6822012981 | -4.3384172646 |
| H95 | -3.6037038140 | 2.1478147199 | -4.4137902795 |
| C96 | -5.2112462849 | 4.4421759011 | -4.0917527228 |
| H97 | -6.0648648084 | 4.0991354144 | -4.6860170571 |
| H98 | -5.4915243438 | 5.3845654451 | -3.6101207608 |
| H99 | -4.3903253799 | 4.6545467611 | -4.7847017106 |
| H100 | -1.8675917968 | -2.4378167344 | 1.6527148283 |

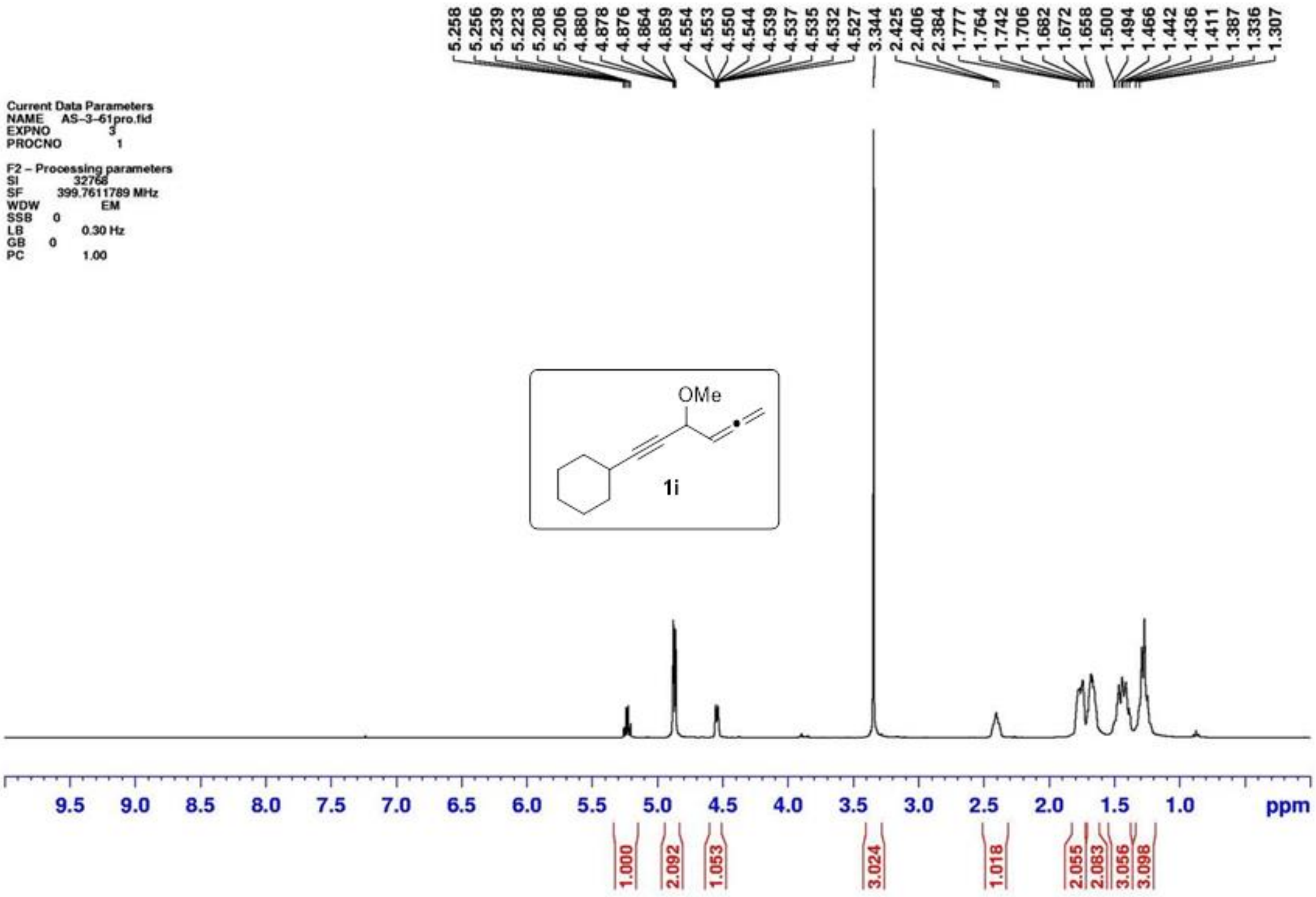
Current Data Parameters
NAME AS-3-61pro.fid
EXPNO 3
PROCNO 1
F2 - Processing parameters
SI 32768
SF 399.7611789 MHz
WDW EM
SSB 0
LB 0.30 Hz
GB 0
PC 1.00
5.258 5.256 5.239 5.223 5.208 5.206 4.880 4.878 4.876 4.864 4.859 4.554 4.553 4.550 4.544 4.539 4.537 4.535 4.532 4.527 3.344 2.425 2.406 2.384 1.777 1.764 1.742 1.706 1.682 1.672 1.658 1.500 1.494 1.466 1.442 1.436 1.411 1.387 1.336 1.307
OMe
1i
9.5 9.0 8.5 8.0 7.5 7.0 6.5 6.0 5.5 5.0 4.5 4.0 3.5 3.0 2.5 2.0 1.5 1.0 ppm
1.000 2.092 1.053 3.024 1.018 2.055 2.083 3.056 3.098

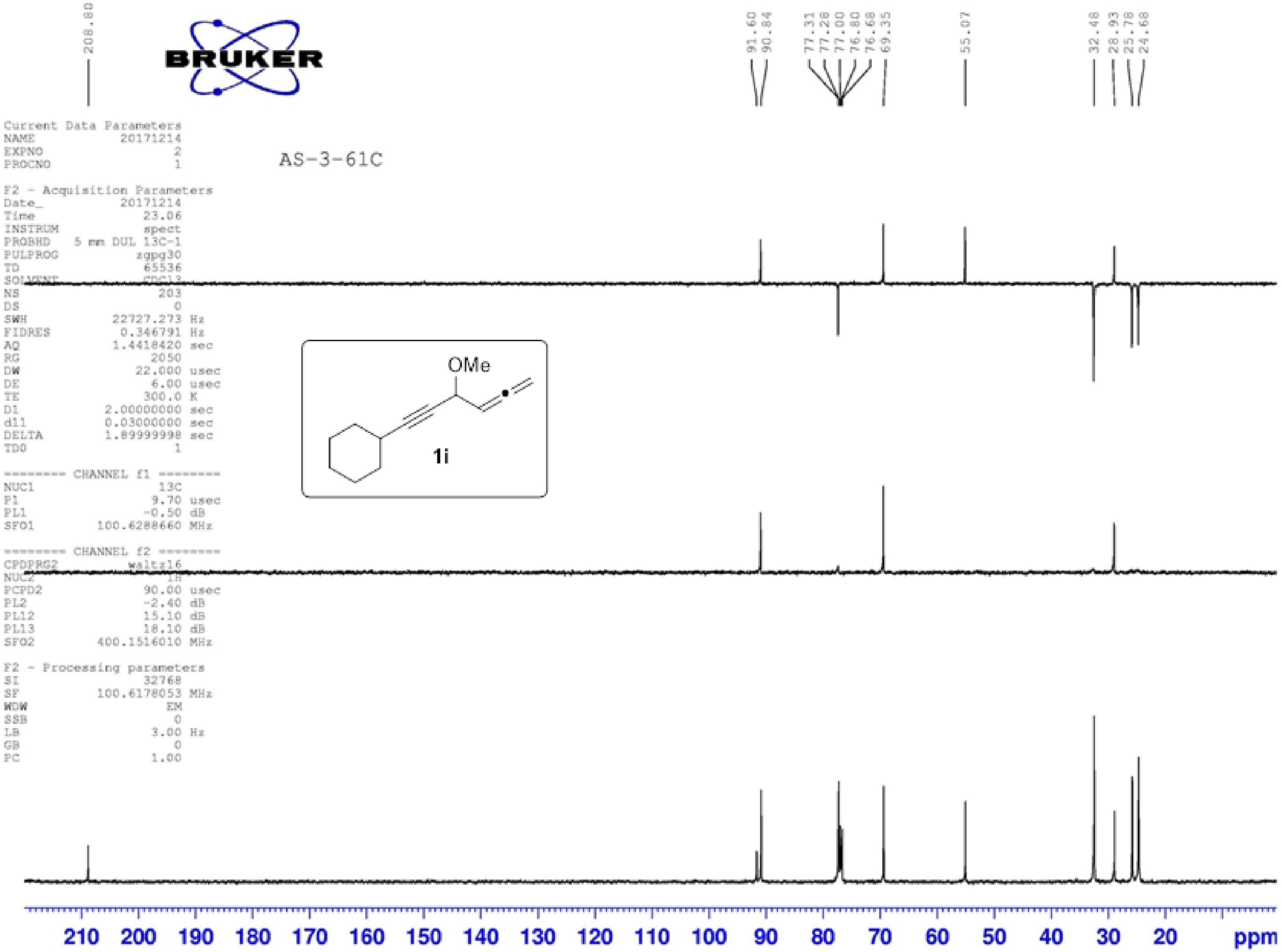
208.80
91.60
90.84
77.31
77.28
77.00
76.80
76.68
69.35
55.07
32.48
28.93
25.78
24.68
BRUKER
Current Data Parameters
NAME 20171214
EXPNO 2
PROCNO 1
AS-3-61C
F2 - Acquisition Parameters
Date_ 20171214
Time 23.06
INSTRUM spect
PROBHD 5 mm DUL 13C-1
PULPROG zgpg30
TD 65536
SOLVENT CDCl3
NS 203
DS 0
SWH 22727.273 Hz
FIDRES 0.346791 Hz
AQ 1.4418420 sec
RG 2050
DW 22.000 usec
DE 6.00 usec
TE 300.0 K
D1 2.00000000 sec
d11 0.03000000 sec
DELTA 1.89999998 sec
TD0 1
======== CHANNEL f1 ========
NUC1 13C
P1 9.70 usec
PL1 -0.50 dB
SFO1 100.6288660 MHz
======== CHANNEL f2 ========
CPDPRG2 waltz16
NUC2 1H
PCPD2 90.00 usec
PL2 -2.40 dB
PL12 15.10 dB
PL13 18.10 dB
SFO2 400.1516010 MHz
F2 - Processing parameters
SI 32768
SF 100.6178053 MHz
WDW EM
SSB 0
LB 3.00 Hz
GB 0
PC 1.00
OMe
1i
210 200 190 180 170 160 150 140 130 120 110 100 90 80 70 60 50 40 30 20 ppm

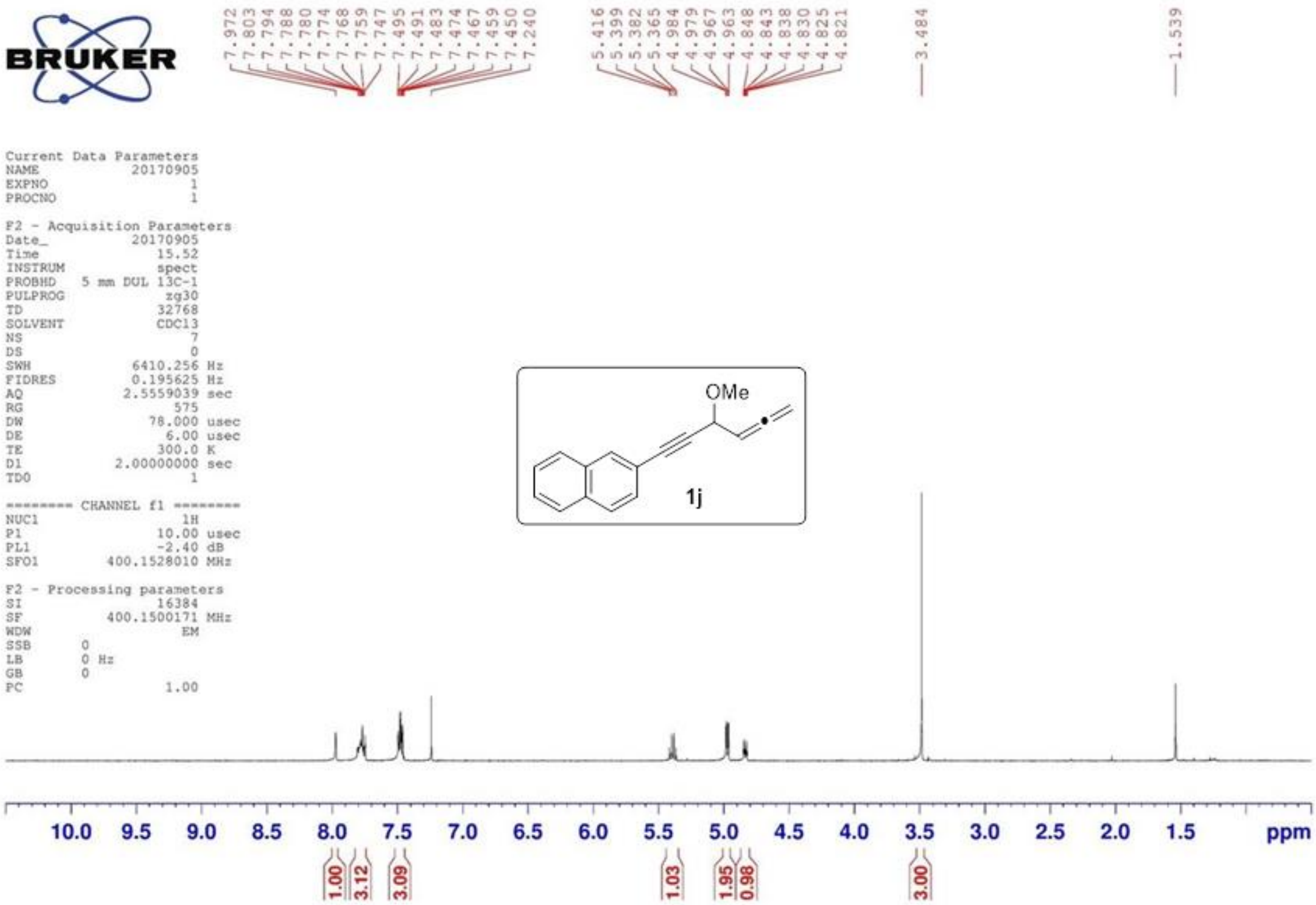
BRUKER
7.972
7.803
7.794
7.788
7.780
7.774
7.768
7.759
7.747
7.495
7.491
7.483
7.474
7.467
7.459
7.450
7.240
5.416
5.399
5.382
5.365
4.984
4.979
4.967
4.963
4.848
4.843
4.838
4.830
4.825
4.821
3.484
1.539
Current Data Parameters
NAME 20170905
EXPNO 1
PROCNO 1
F2 - Acquisition Parameters
Date_ 20170905
Time 15.52
INSTRUM spect
PROBHD 5 mm DUL 13C-1
PULPROG zg30
TD 32768
SOLVENT CDCl3
NS 7
DS 0
SWH 6410.256 Hz
FIDRES 0.195625 Hz
AQ 2.5559039 sec
RG 575
DW 78.000 usec
DE 6.00 usec
TE 300.0 K
D1 2.00000000 sec
TD0 1
======== CHANNEL f1 ========
NUC1 1H
P1 10.00 usec
PL1 -2.40 dB
SFO1 400.1528010 MHz
F2 - Processing parameters
SI 16384
SF 400.1500171 MHz
WDW EM
SSB 0
LB 0 Hz
GB 0
PC 1.00
OMe
1j
10.0 9.5 9.0 8.5 8.0 7.5 7.0 6.5 6.0 5.5 5.0 4.5 4.0 3.5 3.0 2.5 2.0 1.5 ppm
1.00
3.12
3.09
1.03
1.95
0.98
3.00

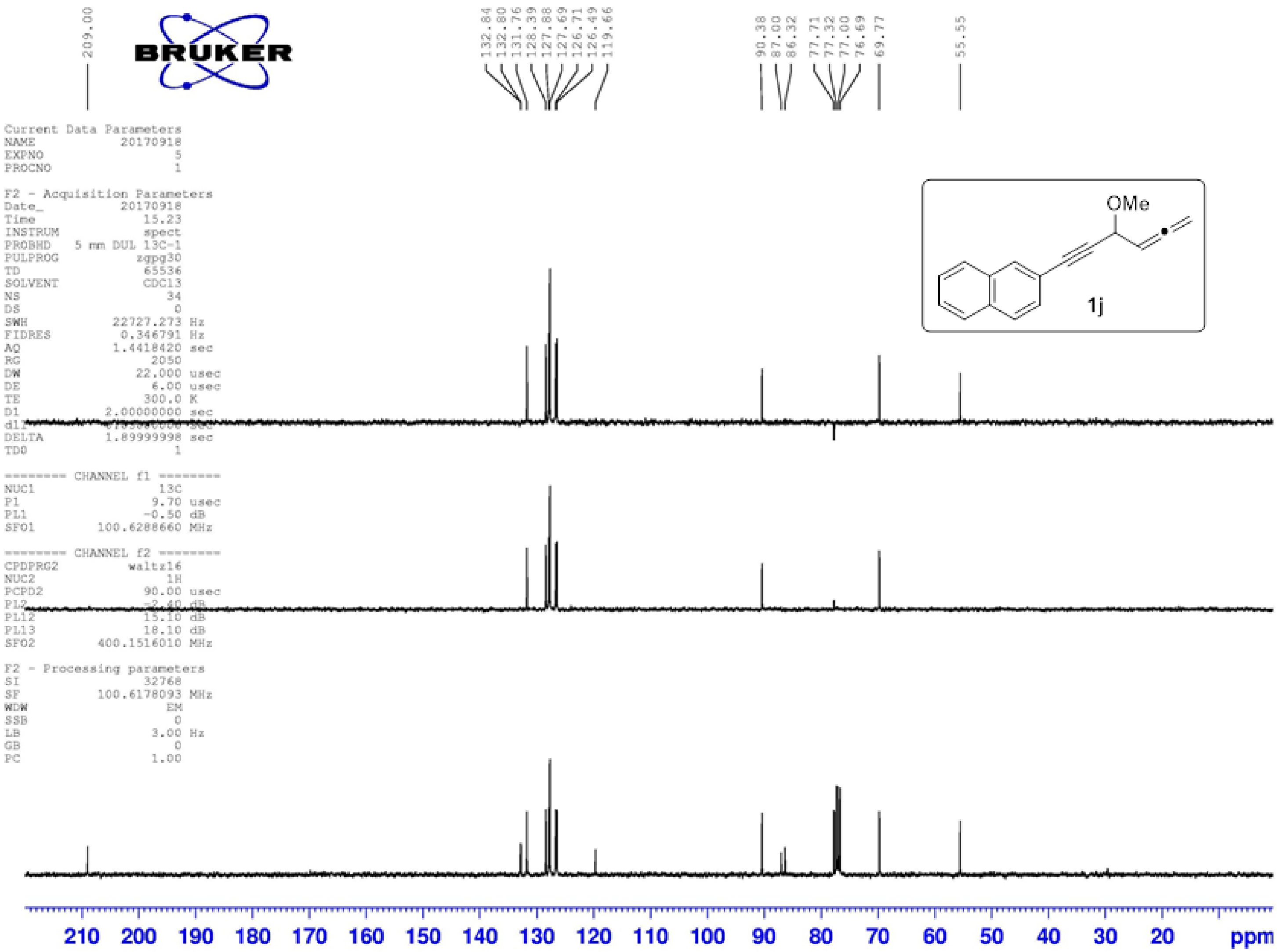
209.00
132.84
132.80
131.76
128.39
127.88
127.69
126.71
126.49
119.66
90.38
87.00
86.32
77.71
77.32
77.00
76.69
69.77
55.55
Current Data Parameters
NAME 20170918
EXPNO 5
PROCNO 1
F2 - Acquisition Parameters
Date_ 20170918
Time 15.23
INSTRUM spect
PROBHD 5 mm DUL 13C-1
PULPROG zgpg30
TD 65536
SOLVENT CDCl3
NS 34
DS 0
SWH 22727.273 Hz
FIDRES 0.346791 Hz
AQ 1.4418420 sec
RG 2050
DW 22.000 usec
DE 6.00 usec
TE 300.0 K
D1 2.00000000 sec
DELTA 1.89999998 sec
TD0 1
======== CHANNEL f1 ========
NUC1 13C
P1 9.70 usec
PL1 -0.50 dB
SFO1 100.6288660 MHz
======== CHANNEL f2 ========
CPDPRG2 waltz16
NUC2 1H
PCPD2 90.00 usec
PL12 15.10 dB
PL13 18.10 dB
SFO2 400.1516010 MHz
F2 - Processing parameters
SI 32768
SF 100.6178093 MHz
WDW EM
SSB 0
LB 3.00 Hz
GB 0
PC 1.00
OMe
1j
210 200 190 180 170 160 150 140 130 120 110 100 90 80 70 60 50 40 30 20 ppm

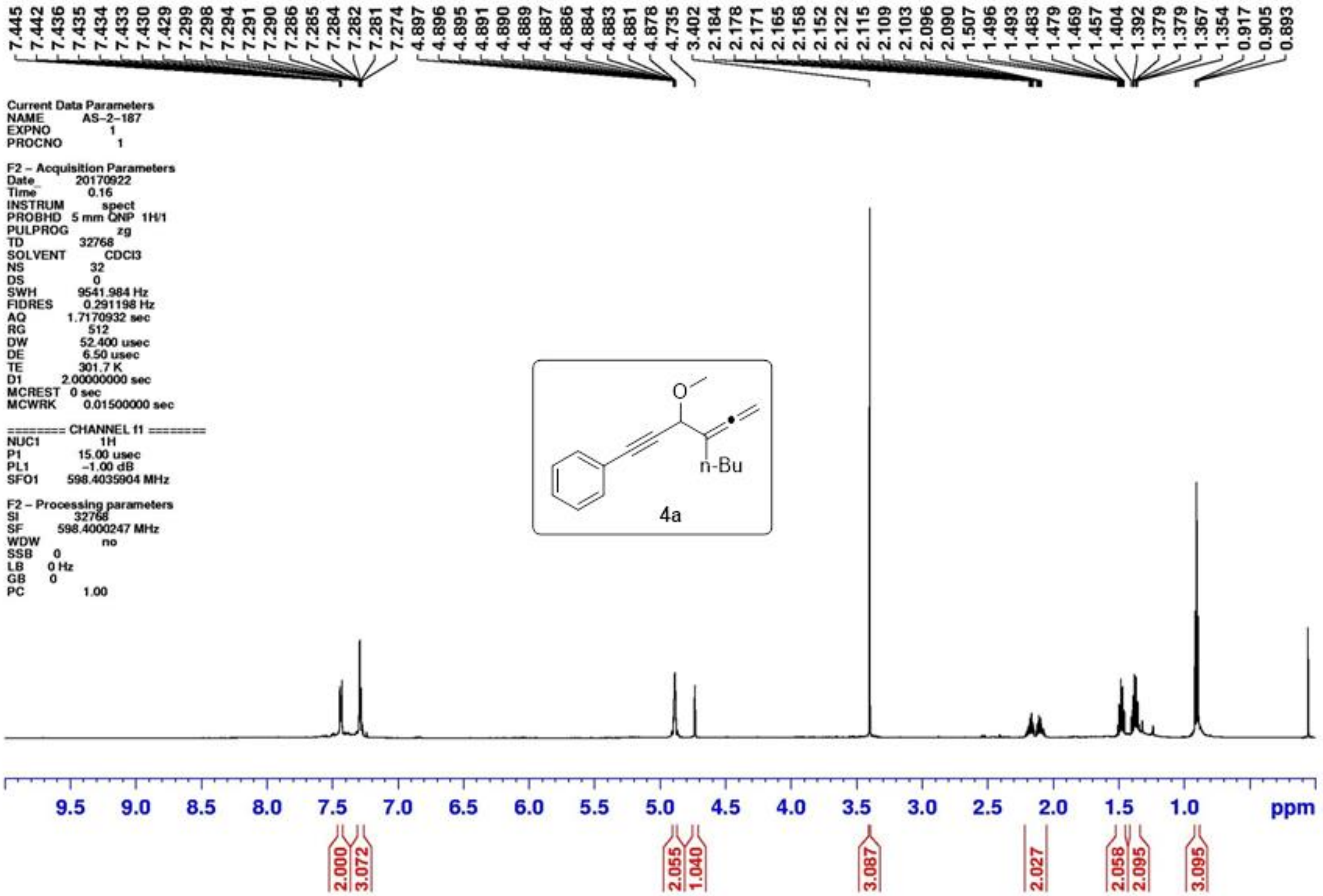
7.445 7.442 7.436 7.435 7.434 7.433 7.430 7.429 7.299 7.298 7.294 7.291 7.290 7.286 7.285 7.284 7.282 7.281 7.274
4.897 4.896 4.895 4.891 4.890 4.889 4.887 4.886 4.884 4.883 4.881 4.878 4.735
3.402
2.184 2.178 2.171 2.165 2.158 2.152 2.122 2.115 2.109 2.103 2.096 2.090
1.507 1.496 1.493 1.483 1.479 1.469 1.457 1.404 1.392 1.379 1.379 1.367 1.354
0.917 0.905 0.893
Current Data Parameters
NAME AS-2-187
EXPNO 1
PROCNO 1
F2 - Acquisition Parameters
Date_ 20170922
Time 0.16
INSTRUM spect
PROBHD 5 mm QNP 1H/1
PULPROG zg
TD 32768
SOLVENT CDCl3
NS 32
DS 0
SWH 9541.984 Hz
FIDRES 0.291198 Hz
AQ 1.7170932 sec
RG 512
DW 52.400 usec
DE 6.50 usec
TE 301.7 K
D1 2.00000000 sec
MCREST 0 sec
MCWRK 0.01500000 sec
======== CHANNEL f1 ========
NUC1 1H
P1 15.00 usec
PL1 -1.00 dB
SFO1 598.4035904 MHz
F2 - Processing parameters
SI 32768
SF 598.4000247 MHz
WDW no
SSB 0
LB 0 Hz
GB 0
PC 1.00
O
n-Bu
4a
9.5 9.0 8.5 8.0 7.5 7.0 6.5 6.0 5.5 5.0 4.5 4.0 3.5 3.0 2.5 2.0 1.5 1.0 ppm
2.000 3.072 2.055 1.040 3.087 2.027 2.058 2.095 3.095

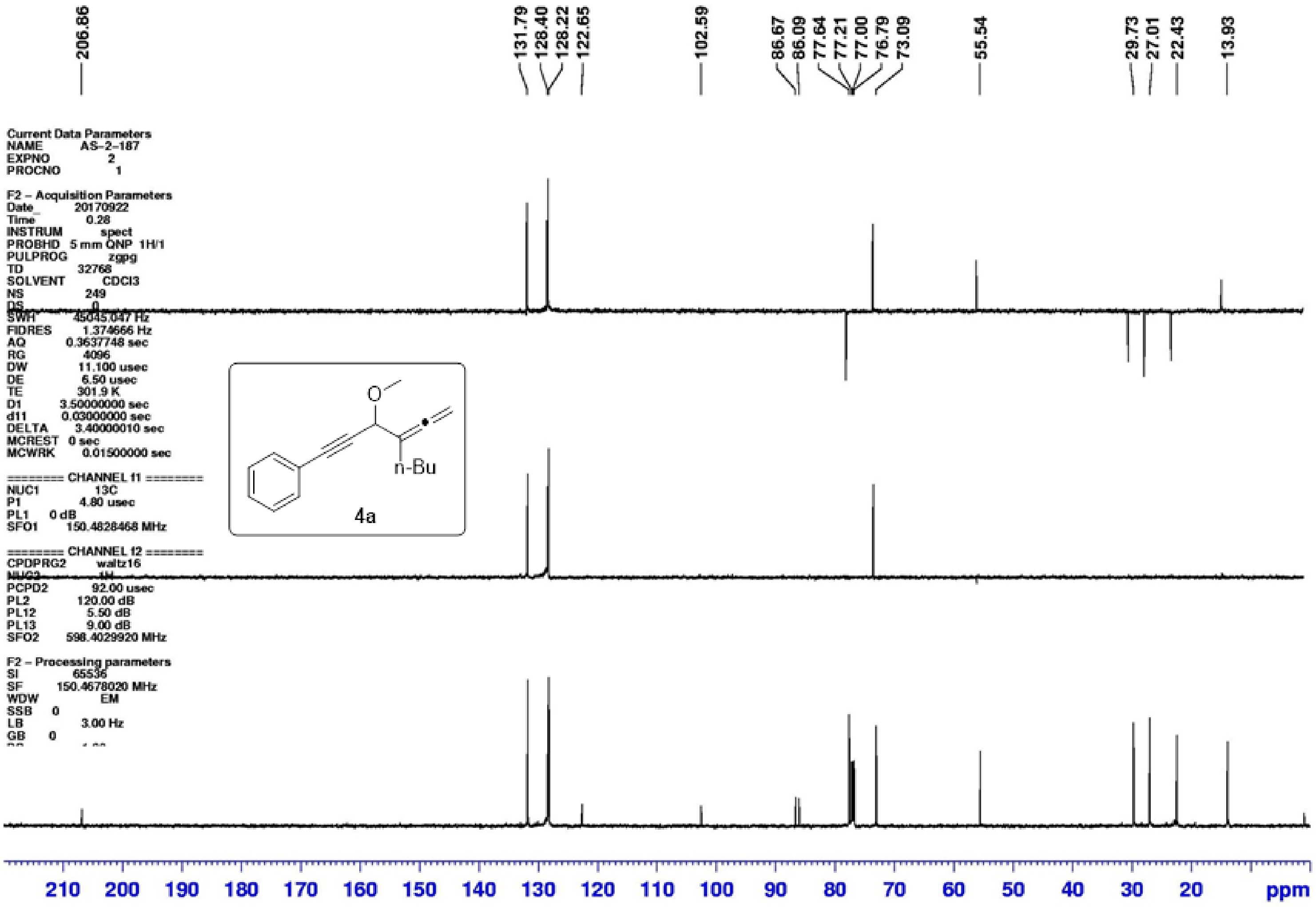

206.86
131.79
128.40
128.22
122.65
102.59
86.67
86.09
77.64
77.21
77.00
76.79
73.09
55.54
29.73
27.01
22.43
13.93
Current Data Parameters
NAME AS-2-187
EXPNO 2
PROCNO 1
F2 - Acquisition Parameters
Date_ 20170922
Time 0.28
INSTRUM spect
PROBHD 5 mm QNP 1H/1
PULPROG zgpg
TD 32768
SOLVENT CDCl3
NS 249
SWH 45045.047 Hz
FIDRES 1.374666 Hz
AQ 0.3637748 sec
RG 4096
DW 11.100 usec
DE 6.50 usec
TE 301.9 K
D1 3.50000000 sec
d11 0.03000000 sec
DELTA 3.40000010 sec
MCREST 0 sec
MCWRK 0.01500000 sec
======== CHANNEL f1 ========
NUC1 13C
P1 4.80 usec
PL1 0 dB
SFO1 150.4828468 MHz
======== CHANNEL f2 ========
CPDPRG2 waltz16
PCPD2 92.00 usec
PL2 120.00 dB
PL12 5.50 dB
PL13 9.00 dB
SFO2 598.4029920 MHz
F2 - Processing parameters
SI 65536
SF 150.4678020 MHz
WDW EM
SSB 0
LB 3.00 Hz
GB 0
O
n-Bu
4a
210 200 190 180 170 160 150 140 130 120 110 100 90 80 70 60 50 40 30 20 ppm

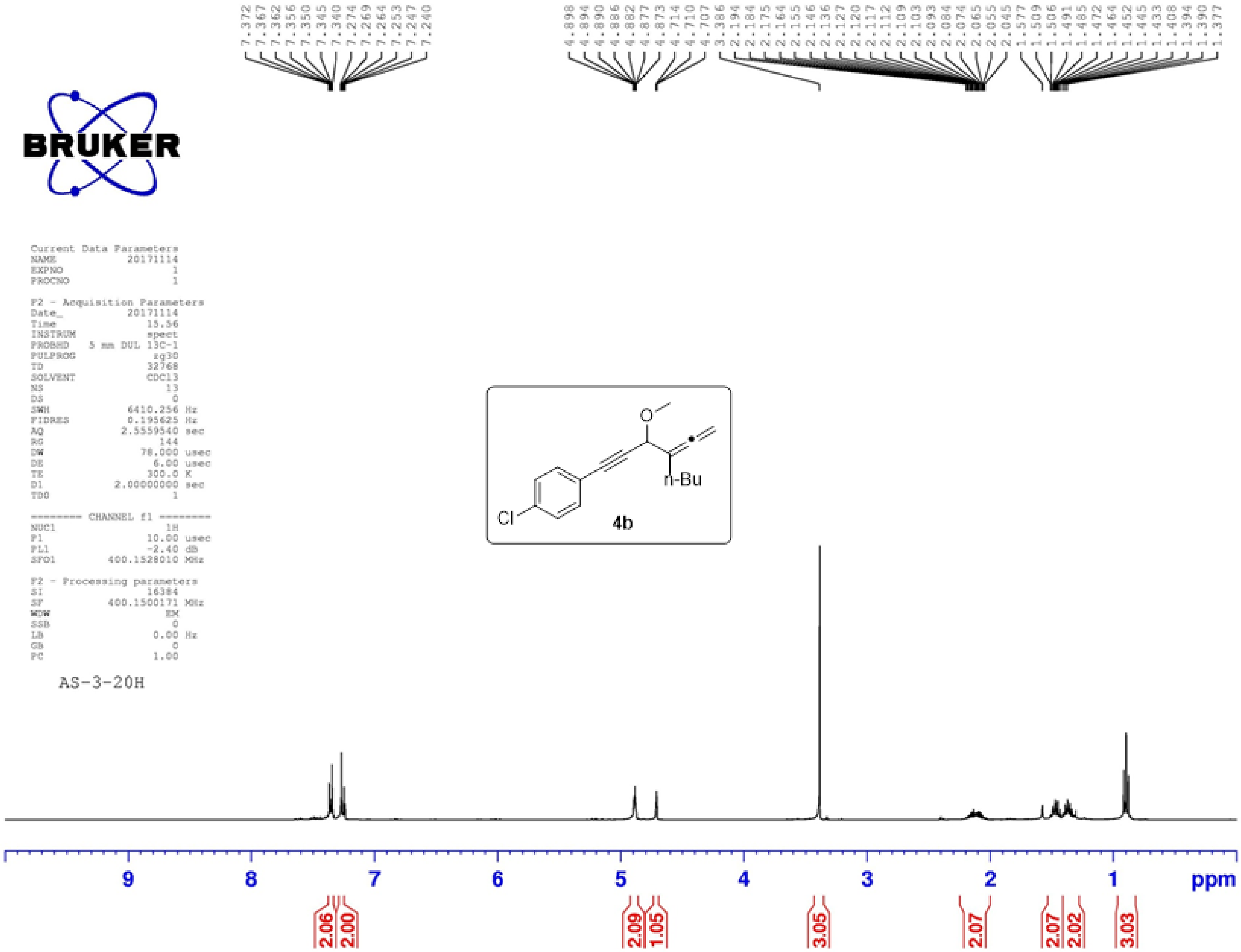

7.372
7.367
7.362
7.356
7.350
7.345
7.340
7.274
7.269
7.264
7.253
7.247
7.240
4.898
4.894
4.890
4.886
4.882
4.877
4.873
4.714
4.710
4.707
3.386
2.194
2.184
2.175
2.164
2.155
2.146
2.136
2.127
2.120
2.117
2.112
2.109
2.103
2.093
2.084
2.074
2.065
2.055
2.045
1.577
1.509
1.506
1.491
1.485
1.472
1.464
1.452
1.445
1.433
1.408
1.394
1.390
1.377
Current Data Parameters
NAME 20171114
EXPNO 1
PROCNO 1
F2 - Acquisition Parameters
Date_ 20171114
Time 15.56
INSTRUM spect
PROBHD 5 mm DUL 13C-1
PULPROG zg30
TD 32768
SOLVENT CDCl3
NS 13
DS 0
SWH 6410.256 Hz
FIDRES 0.195625 Hz
AQ 2.5559540 sec
RG 144
DW 78.000 usec
DE 6.00 usec
TE 300.0 K
D1 2.00000000 sec
TD0 1
======== CHANNEL f1 ========
NUC1 1H
P1 10.00 usec
PL1 -2.40 dB
SFO1 400.1528010 MHz
F2 - Processing parameters
SI 16384
SF 400.1500171 MHz
WDW EM
SSB 0
LB 0.00 Hz
GB 0
PC 1.00
AS-3-20H
O
Cl
n-Bu
4b
9
8
7
6
5
4
3
2
1
ppm
2.06
2.00
2.09
1.05
3.05
2.07
2.07
2.02
3.03

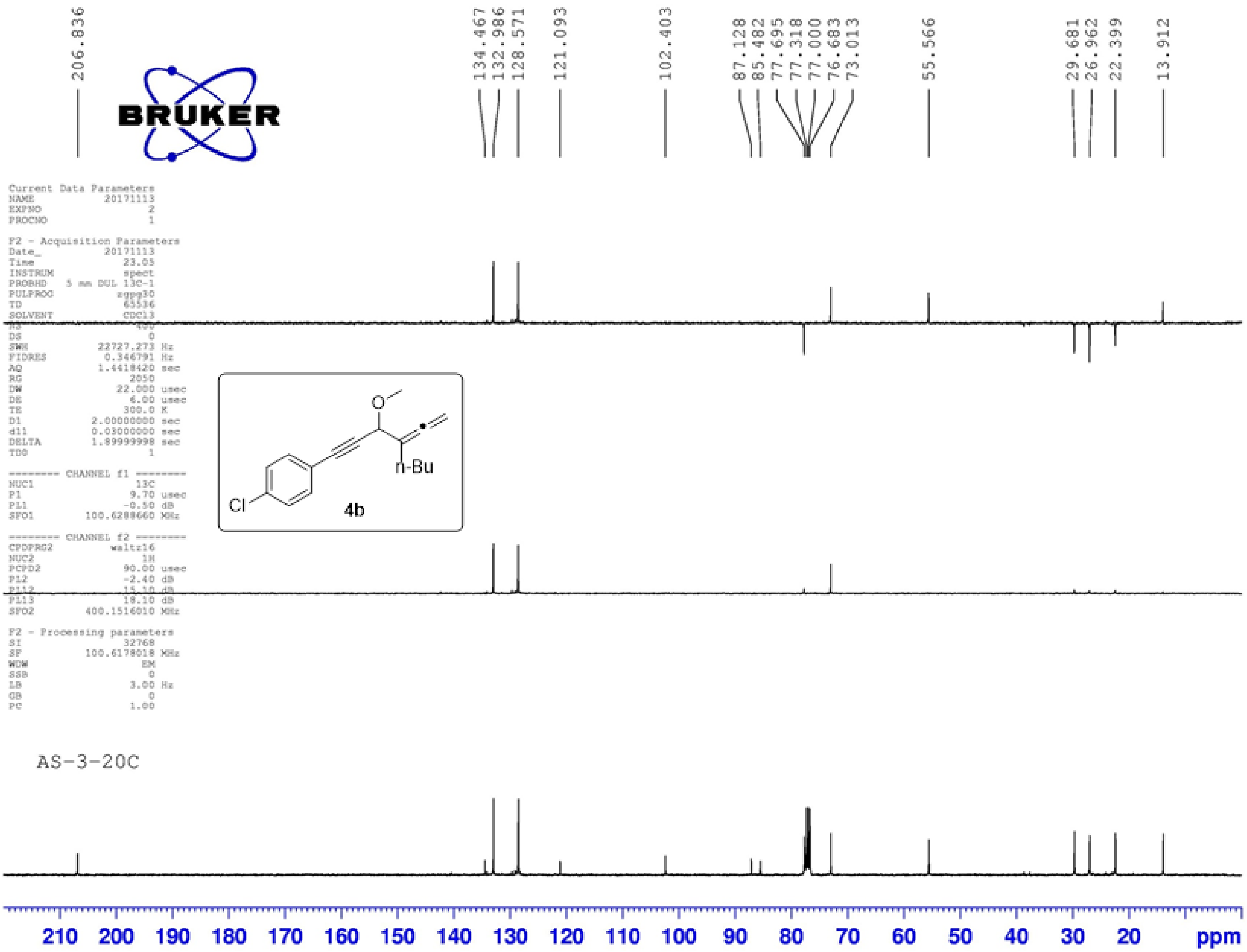

206.836
134.467
132.986
128.571
121.093
102.403
87.128
85.482
77.695
77.318
77.000
76.683
73.013
55.566
29.681
26.962
22.399
13.912
Current Data Parameters
NAME 20171113
EXPNO 2
PROCNO 1
F2 - Acquisition Parameters
Date_ 20171113
Time 23.05
INSTRUM spect
PROBHD 5 mm DUL 13C-1
PULPROG zgpg30
TD 65536
SOLVENT CDCl3
DS 0
SWH 22727.273 Hz
FIDRES 0.346791 Hz
AQ 1.4418420 sec
RG 2050
DW 22.000 usec
DE 6.00 usec
TE 300.0 K
D1 2.00000000 sec
d11 0.03000000 sec
DELTA 1.89999998 sec
TD0 1
======== CHANNEL f1 ========
NUC1 13C
P1 9.70 usec
PL1 -0.50 dB
SFO1 100.6288660 MHz
======== CHANNEL f2 ========
CPDPRG2 waltz16
NUC2 1H
PCPD2 90.00 usec
PL2 -2.40 dB
PL12 15.10 dB
PL13 18.10 dB
SFO2 400.1516010 MHz
F2 - Processing parameters
SI 32768
SF 100.6178018 MHz
WDW EM
SSB 0
LB 3.00 Hz
GB 0
PC 1.00
O
Cl
n-Bu
4b
AS-3-20C
210 200 190 180 170 160 150 140 130 120 110 100 90 80 70 60 50 40 30 20 ppm

```
Current Data Parameters
NAME              20171130
EXPNO                    1
PROCNO                   1

F2 - Acquisition Parameters
Date_             20171130
Time                 22.09
INSTRUM              spect
PROBHD   5 mm DUL 13C-1
PULPROG               zg30
TD                   32768
SOLVENT              CDCl3
NS                      11
DS                       0
SWH               6410.256 Hz
FIDRES            0.195625 Hz
AQ               2.5559540 sec
RG                     362
DW                  78.000 usec
DE                    6.00 usec
TE                   300.0 K
D1              2.00000000 sec
TD0                      1

======== CHANNEL f1 ========
NUC1                    1H
P1                   10.00 usec
PL1                  -2.40 dB
SFO1           400.1528010 MHz

F2 - Processing parameters
SI                   16384
SF             400.1500168 MHz
WDW                     EM
SSB                      0
LB                    0.00 Hz
GB                       0
PC                    1.00
```

AS-3-31A

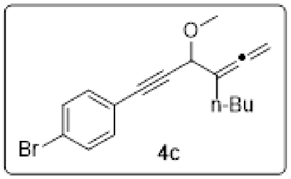


4c

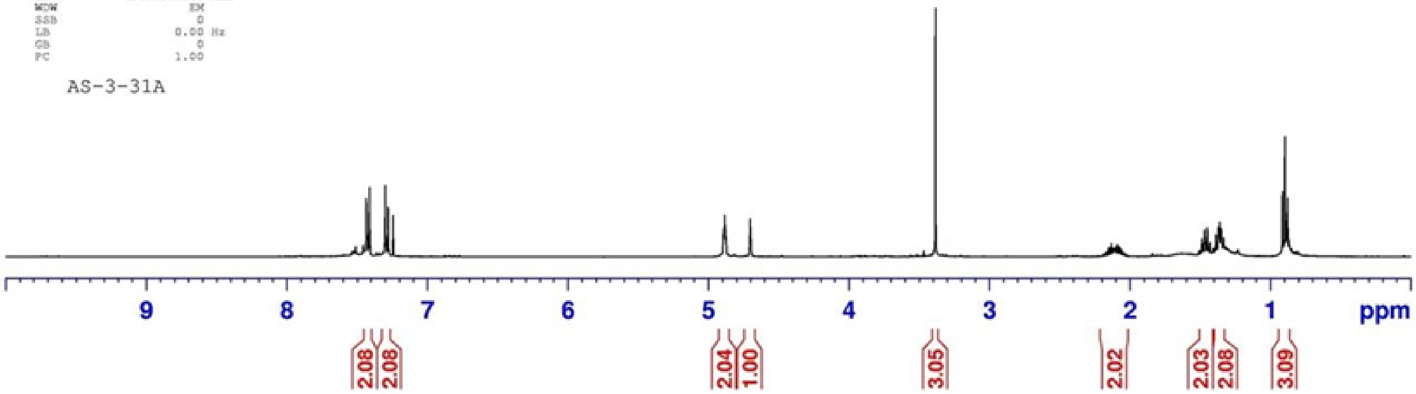

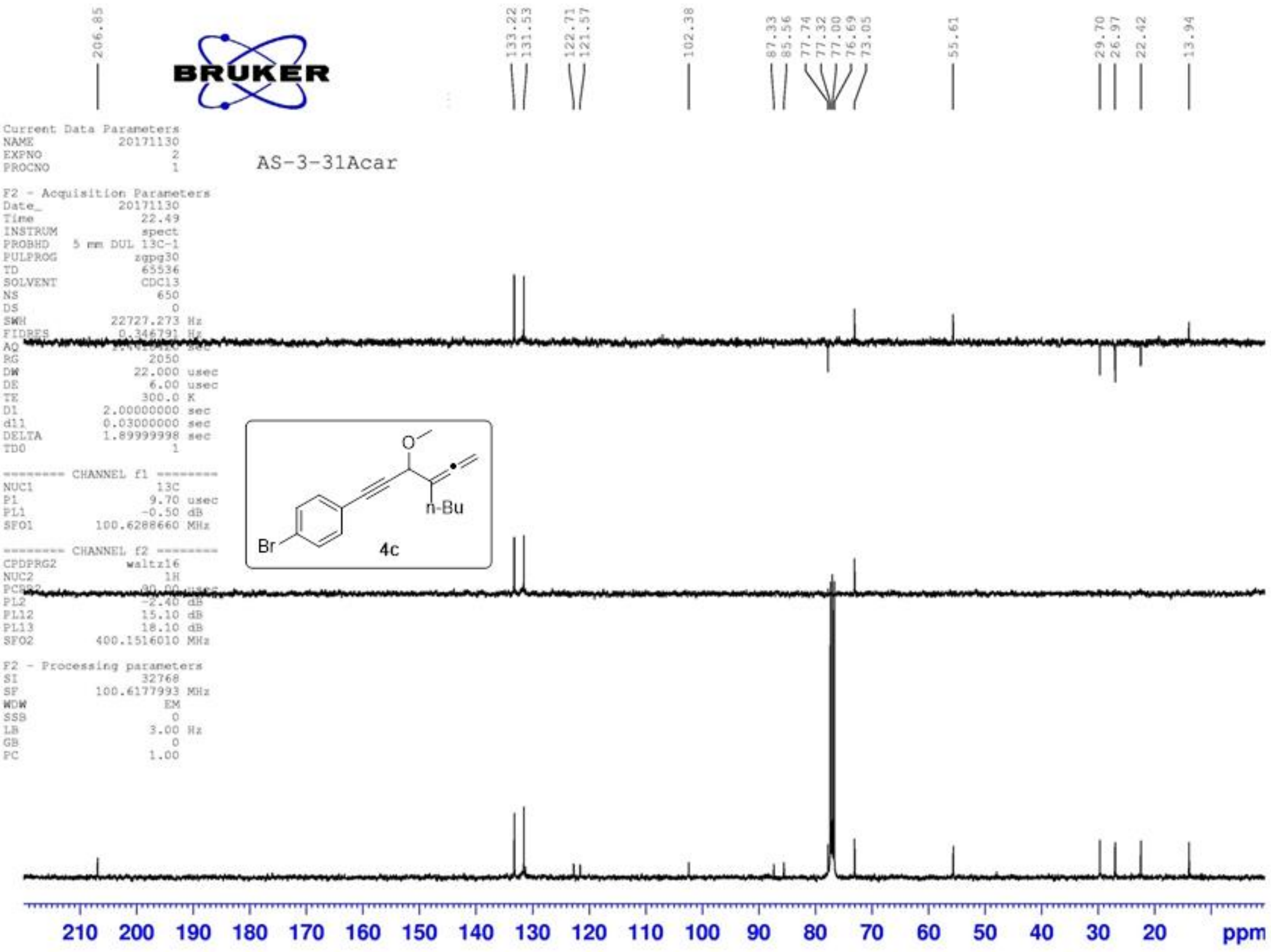
206.85
133.22
131.53
122.71
121.57
102.38
87.33
85.56
77.74
77.32
77.00
76.69
73.05
55.61
29.70
26.97
22.42
13.94
BRUKER
AS-3-31Acar
Current Data Parameters
NAME 20171130
EXPNO 2
PROCNO 1
F2 - Acquisition Parameters
Date_ 20171130
Time 22.49
INSTRUM spect
PROBHD 5 mm DUL 13C-1
PULPROG zgpg30
TD 65536
SOLVENT CDCl3
NS 650
DS 0
SWH 22727.273 Hz
FIDRES 0.346791 Hz
RG 2050
DW 22.000 usec
DE 6.00 usec
TE 300.0 K
D1 2.00000000 sec
d11 0.03000000 sec
DELTA 1.89999998 sec
TD0 1
======== CHANNEL f1 ========
NUC1 13C
P1 9.70 usec
PL1 -0.50 dB
SFO1 100.6288660 MHz
======== CHANNEL f2 ========
CPDPRG2 waltz16
NUC2 1H
PL12 15.10 dB
PL13 18.10 dB
SFO2 400.1516010 MHz
F2 - Processing parameters
SI 32768
SF 100.6177993 MHz
WDW EM
SSB 0
LB 3.00 Hz
GB 0
PC 1.00
O
n-Bu
Br
4c
210 200 190 180 170 160 150 140 130 120 110 100 90 80 70 60 50 40 30 20 ppm

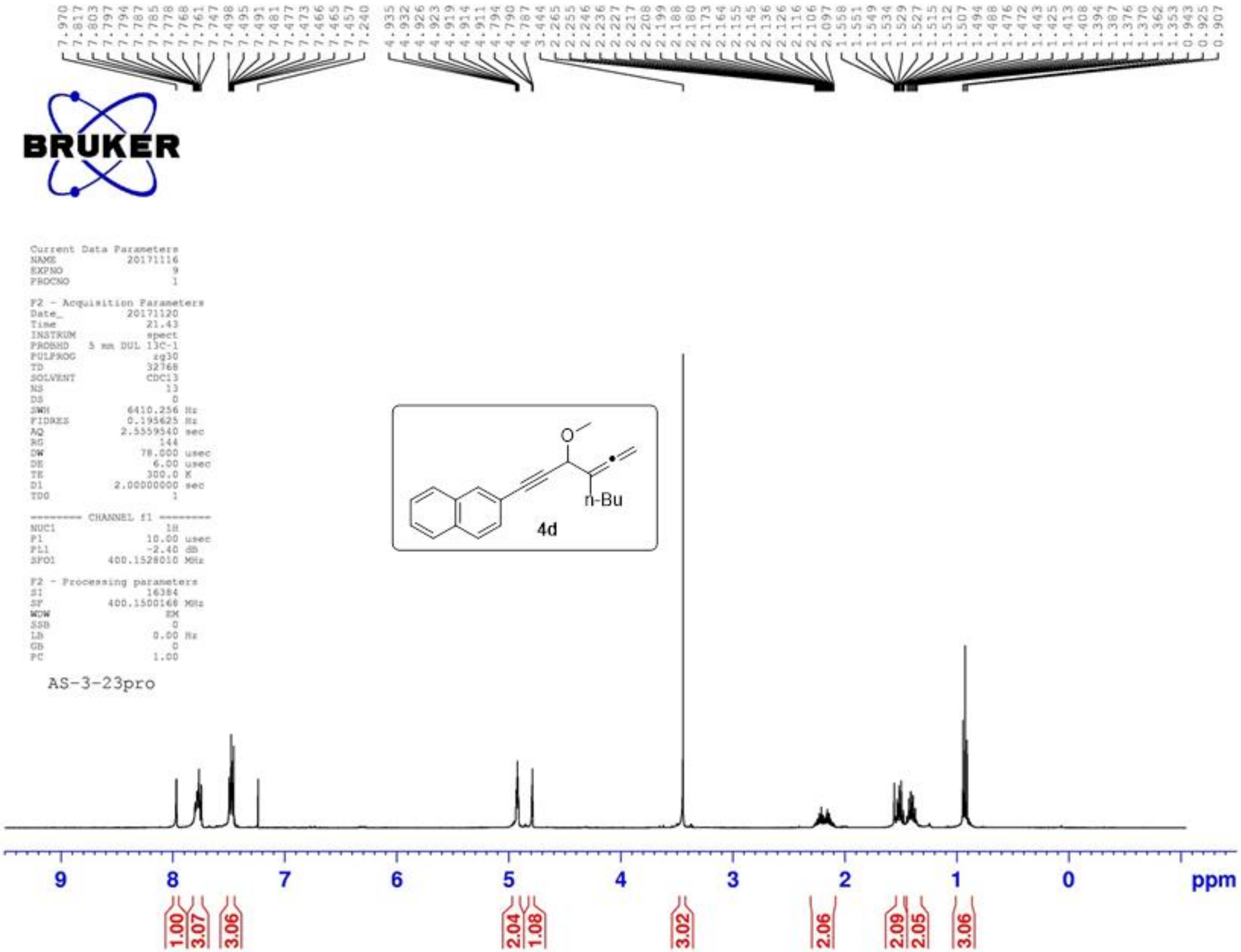

7.970 7.817 7.803 7.797 7.794 7.787 7.785 7.778 7.768 7.761 7.747 7.498 7.495 7.491 7.481 7.477 7.473 7.466 7.465 7.457 7.240 4.935 4.932 4.926 4.923 4.919 4.914 4.911 4.794 4.790 4.787 3.444 2.265 2.255 2.246 2.236 2.227 2.217 2.208 2.199 2.188 2.180 2.173 2.164 2.155 2.145 2.136 2.126 2.116 2.106 2.097 1.558 1.551 1.549 1.534 1.529 1.527 1.515 1.512 1.507 1.494 1.488 1.476 1.472 1.443 1.425 1.413 1.408 1.394 1.387 1.376 1.370 1.362 1.353 0.943 0.925 0.907
BRUKER
Current Data Parameters
NAME 20171116
EXPNO 9
PROCNO 1
F2 - Acquisition Parameters
Date_ 20171120
Time 21.43
INSTRUM spect
PROBHD 5 mm DUL 13C-1
PULPROG zg30
TD 32768
SOLVENT CDCl3
NS 13
DS 0
SWH 6410.256 Hz
FIDRES 0.195625 Hz
AQ 2.5559540 sec
RG 144
DW 78.000 usec
DE 6.00 usec
TE 300.0 K
D1 2.00000000 sec
TD0 1
======== CHANNEL f1 ========
NUC1 1H
P1 10.00 usec
PL1 -2.40 dB
SFO1 400.1528010 MHz
F2 - Processing parameters
SI 16384
SF 400.1500168 MHz
WDW EM
SSB 0
LB 0.00 Hz
GB 0
PC 1.00
AS-3-23pro
O
n-Bu
4d
9 8 7 6 5 4 3 2 1 0 ppm
1.00 3.07 3.06 2.04 1.08 3.02 2.06 2.09 2.05 3.06

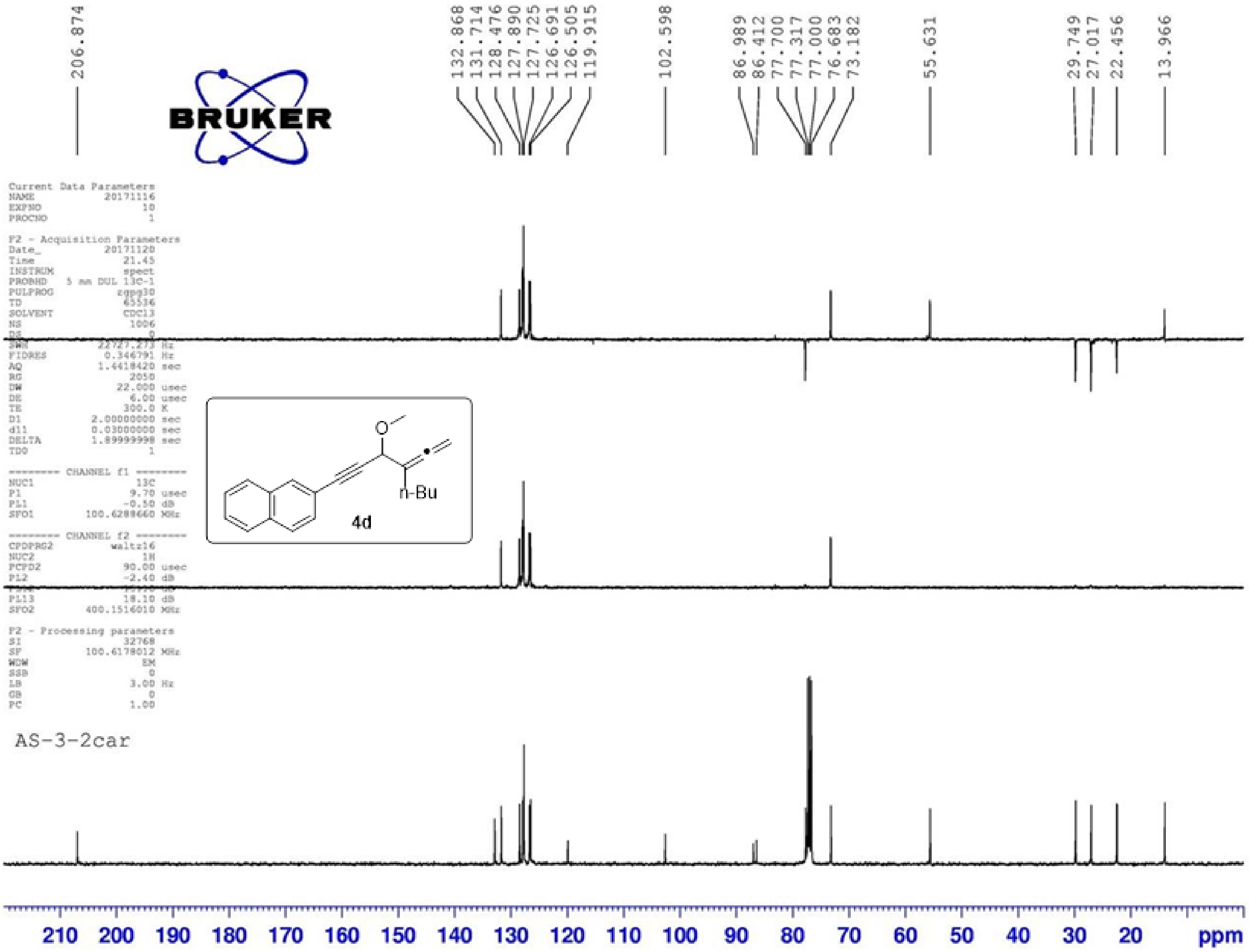
206.874
132.868
131.714
128.476
127.890
127.725
126.691
126.505
119.915
102.598
86.989
86.412
77.700
77.317
77.000
76.683
73.182
55.631
29.749
27.017
22.456
13.966
BRUKER
Current Data Parameters
NAME 20171116
EXPNO 10
PROCNO 1
F2 - Acquisition Parameters
Date_ 20171120
Time 21.45
INSTRUM spect
PROBHD 5 mm DUL 13C-1
PULPROG zgpg30
TD 65536
SOLVENT CDCl3
NS 1006
DS 0
SWH 22727.273 Hz
FIDRES 0.346791 Hz
AQ 1.4418420 sec
RG 2050
DW 22.000 usec
DE 6.00 usec
TE 300.0 K
D1 2.00000000 sec
d11 0.03000000 sec
DELTA 1.89999998 sec
TD0 1
======== CHANNEL f1 ========
NUC1 13C
P1 9.70 usec
PL1 -0.50 dB
SFO1 100.6288660 MHz
======== CHANNEL f2 ========
CPDPRG2 waltz16
NUC2 1H
PCPD2 90.00 usec
PL2 -2.40 dB
PL13 18.10 dB
SFO2 400.1516010 MHz
F2 - Processing parameters
SI 32768
SF 100.6178012 MHz
WDW EM
SSB 0
LB 3.00 Hz
GB 0
PC 1.00
AS-3-2car
O
n-Bu
4d
210
200
190
180
170
160
150
140
130
120
110
100
90
80
70
60
50
40
30
20
ppm

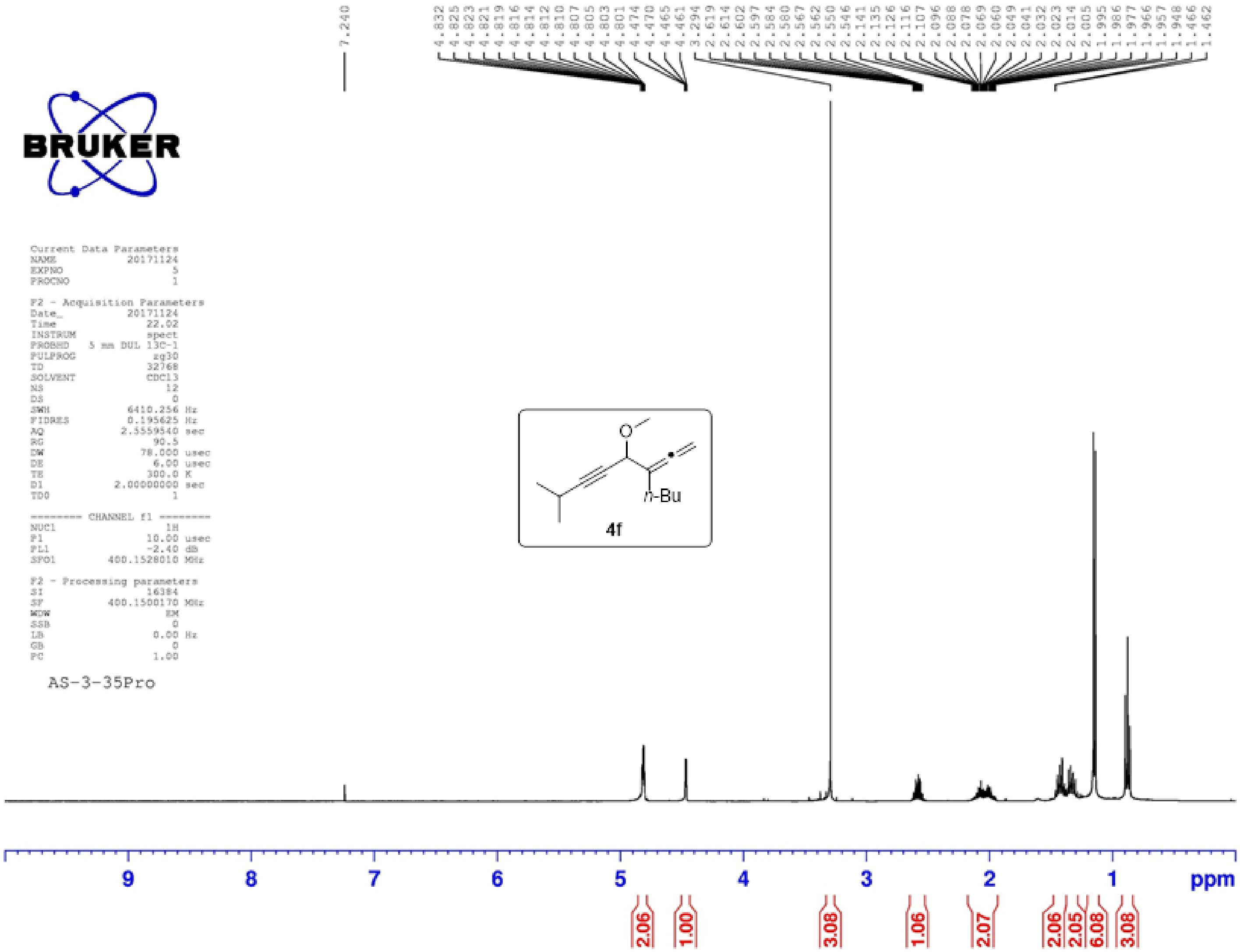
7.240
4.832 4.825 4.823 4.821 4.819 4.816 4.814 4.812 4.810 4.807 4.805 4.803 4.801 4.474 4.470 4.465 4.461 3.294 2.619 2.614 2.602 2.597 2.584 2.580 2.567 2.562 2.550 2.546 2.141 2.135 2.126 2.116 2.107 2.096 2.088 2.078 2.069 2.060 2.049 2.041 2.032 2.023 2.014 2.005 1.995 1.986 1.977 1.966 1.957 1.948 1.466 1.462
BRUKER
Current Data Parameters
NAME 20171124
EXPNO 5
PROCNO 1
F2 - Acquisition Parameters
Date_ 20171124
Time 22.02
INSTRUM spect
PROBHD 5 mm DUL 13C-1
PULPROG zg30
TD 32768
SOLVENT CDCl3
NS 12
DS 0
SWH 6410.256 Hz
FIDRES 0.195625 Hz
AQ 2.5559540 sec
RG 90.5
DW 78.000 usec
DE 6.00 usec
TE 300.0 K
D1 2.00000000 sec
TD0 1
======== CHANNEL f1 ========
NUC1 1H
P1 10.00 usec
PL1 -2.40 dB
SFO1 400.1528010 MHz
F2 - Processing parameters
SI 16384
SF 400.1500170 MHz
WDW EM
SSB 0
LB 0.00 Hz
GB 0
PC 1.00
AS-3-35Pro
O
n-Bu
4f
9 8 7 6 5 4 3 2 1 ppm
2.06 1.00 3.08 1.06 2.07 2.06 2.05 6.08 3.08

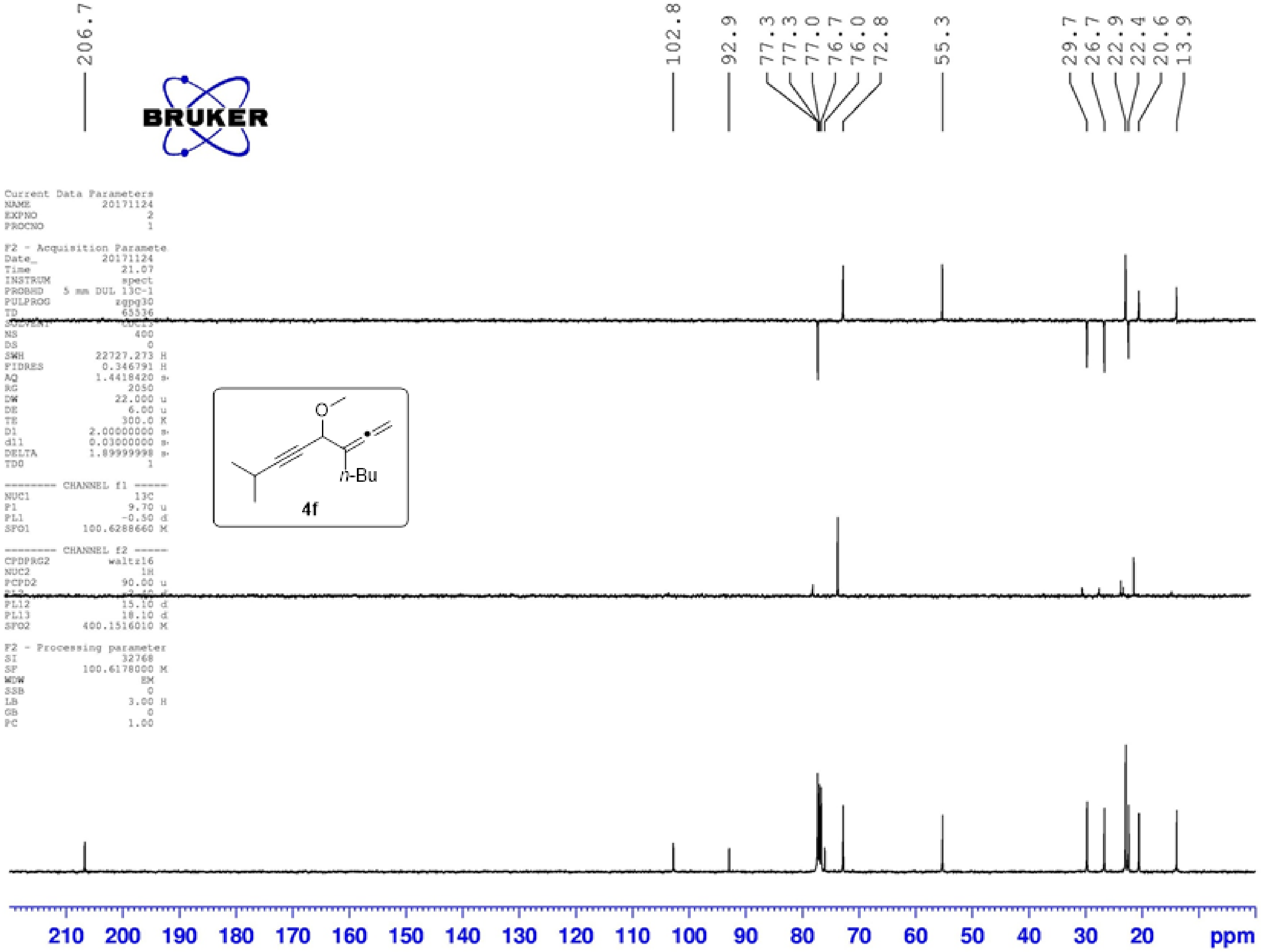
206.7
102.8
92.9
77.3
77.3
77.0
76.7
76.0
72.8
55.3
29.7
26.7
22.9
22.4
20.6
13.9
BRUKER
Current Data Parameters
NAME 20171124
EXPNO 2
PROCNO 1
F2 - Acquisition Paramete
Date_ 20171124
Time 21.07
INSTRUM spect
PROBHD 5 mm DUL 13C-1
PULPROG zgpg30
TD 65536
SOLVENT CDCl3
NS 400
DS 0
SWH 22727.273 H
FIDRES 0.346791 H
AQ 1.4418420 s
RG 2050
DW 22.000 u
DE 6.00 u
TE 300.0 K
D1 2.00000000 s
d11 0.03000000 s
DELTA 1.89999998 s
TD0 1
======== CHANNEL f1 ======
NUC1 13C
P1 9.70 u
PL1 -0.50 d
SFO1 100.6288660 M
======== CHANNEL f2 ======
CPDPRG2 waltz16
NUC2 1H
PCPD2 90.00 u
PL12 15.10 d
PL13 18.10 d
SFO2 400.1516010 M
F2 - Processing parameter
SI 32768
SF 100.6178000 M
WDW EM
SSB 0
LB 3.00 H
GB 0
PC 1.00
O
n-Bu
4f
210 200 190 180 170 160 150 140 130 120 110 100 90 80 70 60 50 40 30 20 ppm

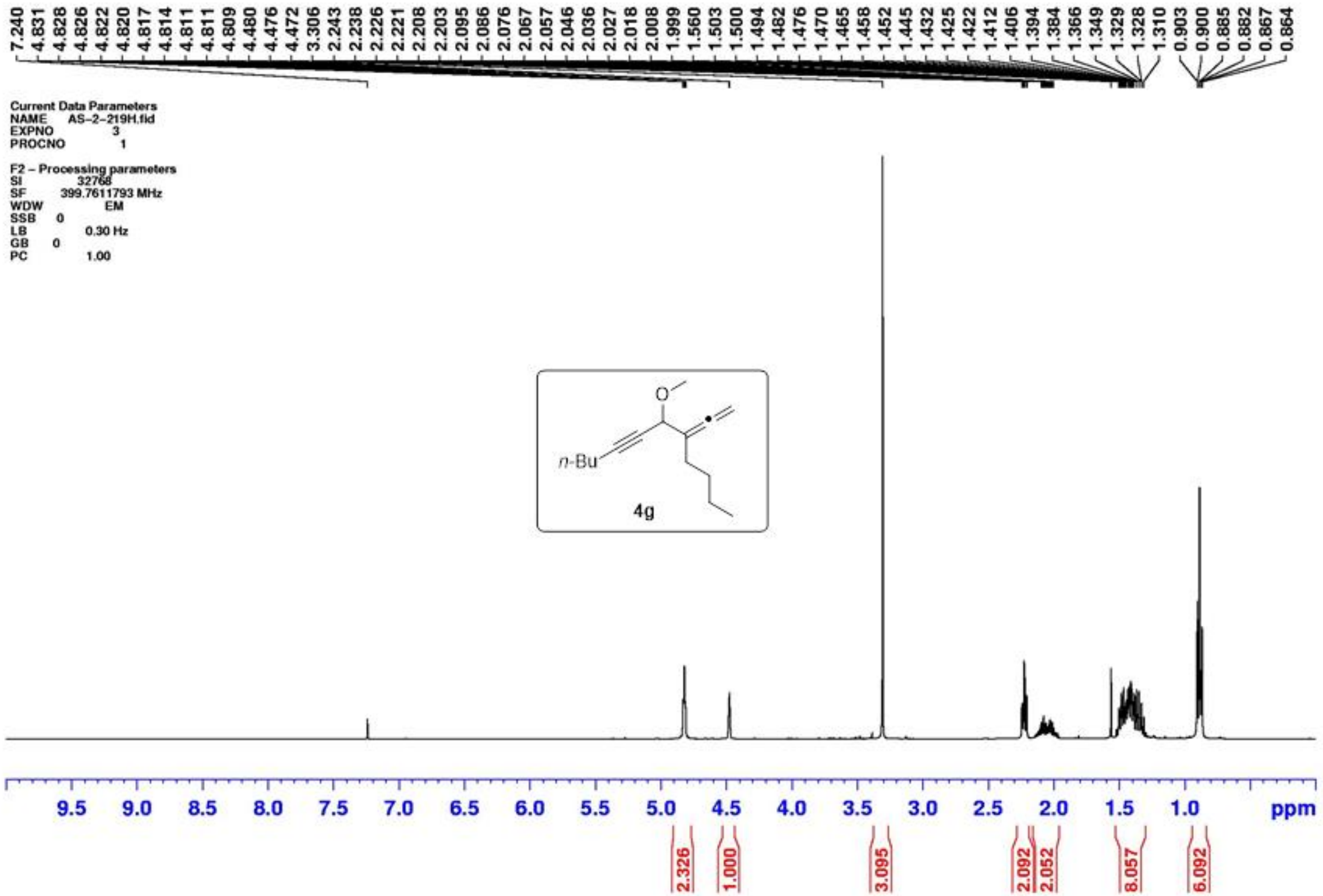
7.240
4.831
4.828
4.826
4.822
4.820
4.817
4.814
4.811
4.811
4.809
4.480
4.476
4.472
3.306
2.243
2.238
2.226
2.221
2.208
2.203
2.095
2.086
2.076
2.067
2.057
2.046
2.036
2.027
2.018
2.008
1.999
1.560
1.503
1.500
1.494
1.482
1.476
1.470
1.465
1.458
1.452
1.445
1.432
1.425
1.422
1.412
1.406
1.394
1.384
1.366
1.349
1.329
1.328
1.310
0.903
0.900
0.885
0.882
0.867
0.864
Current Data Parameters
NAME AS-2-219H.fid
EXPNO 3
PROCNO 1
F2 - Processing parameters
SI 32768
SF 399.7611793 MHz
WDW EM
SSB 0
LB 0.30 Hz
GB 0
PC 1.00
n-Bu
O
4g
9.5
9.0
8.5
8.0
7.5
7.0
6.5
6.0
5.5
5.0
4.5
4.0
3.5
3.0
2.5
2.0
1.5
1.0
ppm
2.326
1.000
3.095
2.092
2.052
8.057
6.092

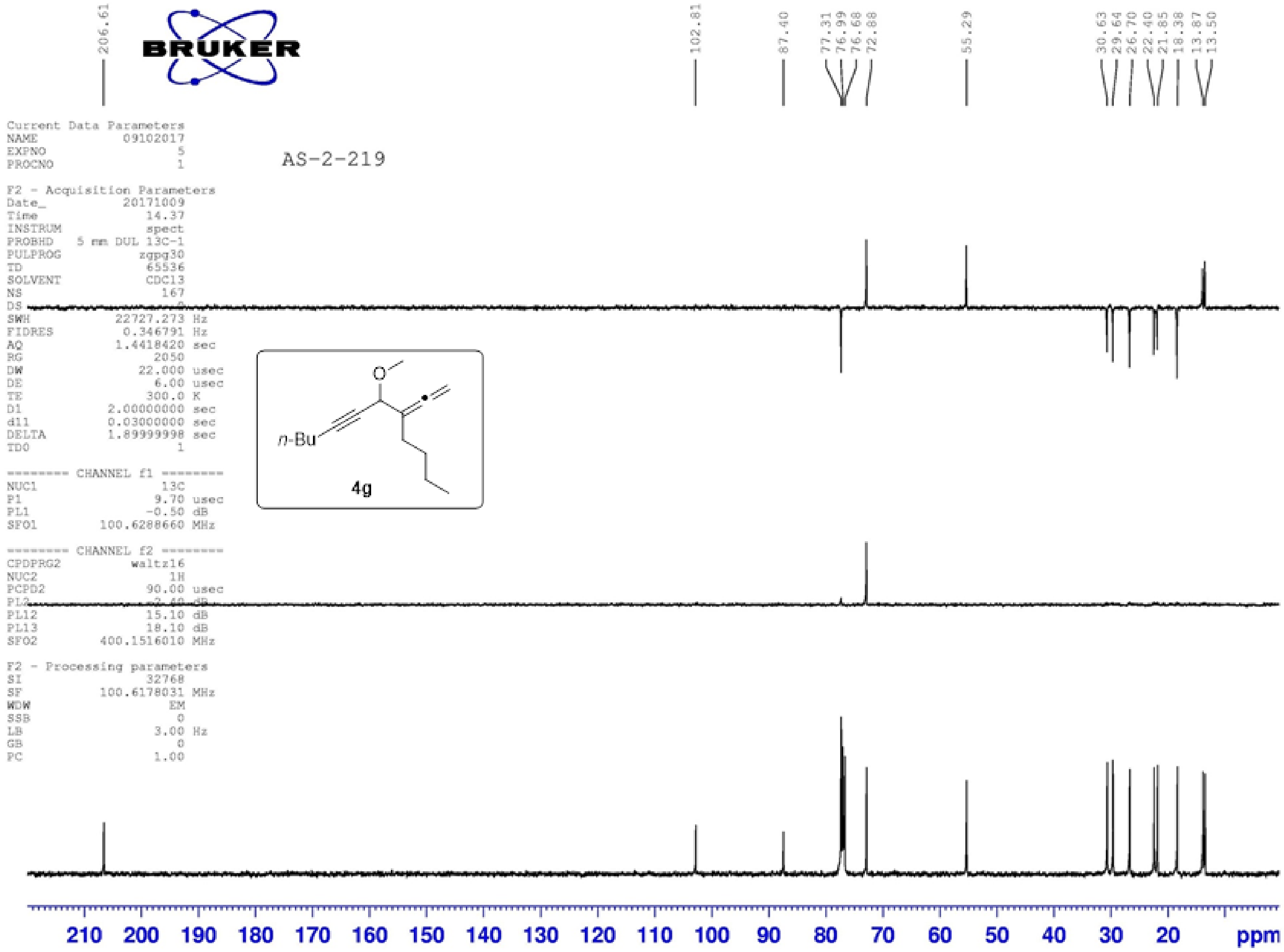

206.61
102.81
87.40
77.31
76.99
76.68
72.88
55.29
30.63
29.64
26.70
22.40
21.85
18.38
13.87
13.50
BRUKER
Current Data Parameters
NAME 09102017
EXPNO 5
PROCNO 1
AS-2-219
F2 - Acquisition Parameters
Date_ 20171009
Time 14.37
INSTRUM spect
PROBHD 5 mm DUL 13C-1
PULPROG zgpg30
TD 65536
SOLVENT CDCl3
NS 167
DS 0
SWH 22727.273 Hz
FIDRES 0.346791 Hz
AQ 1.4418420 sec
RG 2050
DW 22.000 usec
DE 6.00 usec
TE 300.0 K
D1 2.00000000 sec
d11 0.03000000 sec
DELTA 1.89999998 sec
TD0 1
======== CHANNEL f1 ========
NUC1 13C
P1 9.70 usec
PL1 -0.50 dB
SFO1 100.6288660 MHz
======== CHANNEL f2 ========
CPDPRG2 waltz16
NUC2 1H
PCPD2 90.00 usec
PL2 -2.40 dB
PL12 15.10 dB
PL13 18.10 dB
SFO2 400.1516010 MHz
F2 - Processing parameters
SI 32768
SF 100.6178031 MHz
WDW EM
SSB 0
LB 3.00 Hz
GB 0
PC 1.00
O
n-Bu
4g
210 200 190 180 170 160 150 140 130 120 110 100 90 80 70 60 50 40 30 20 ppm

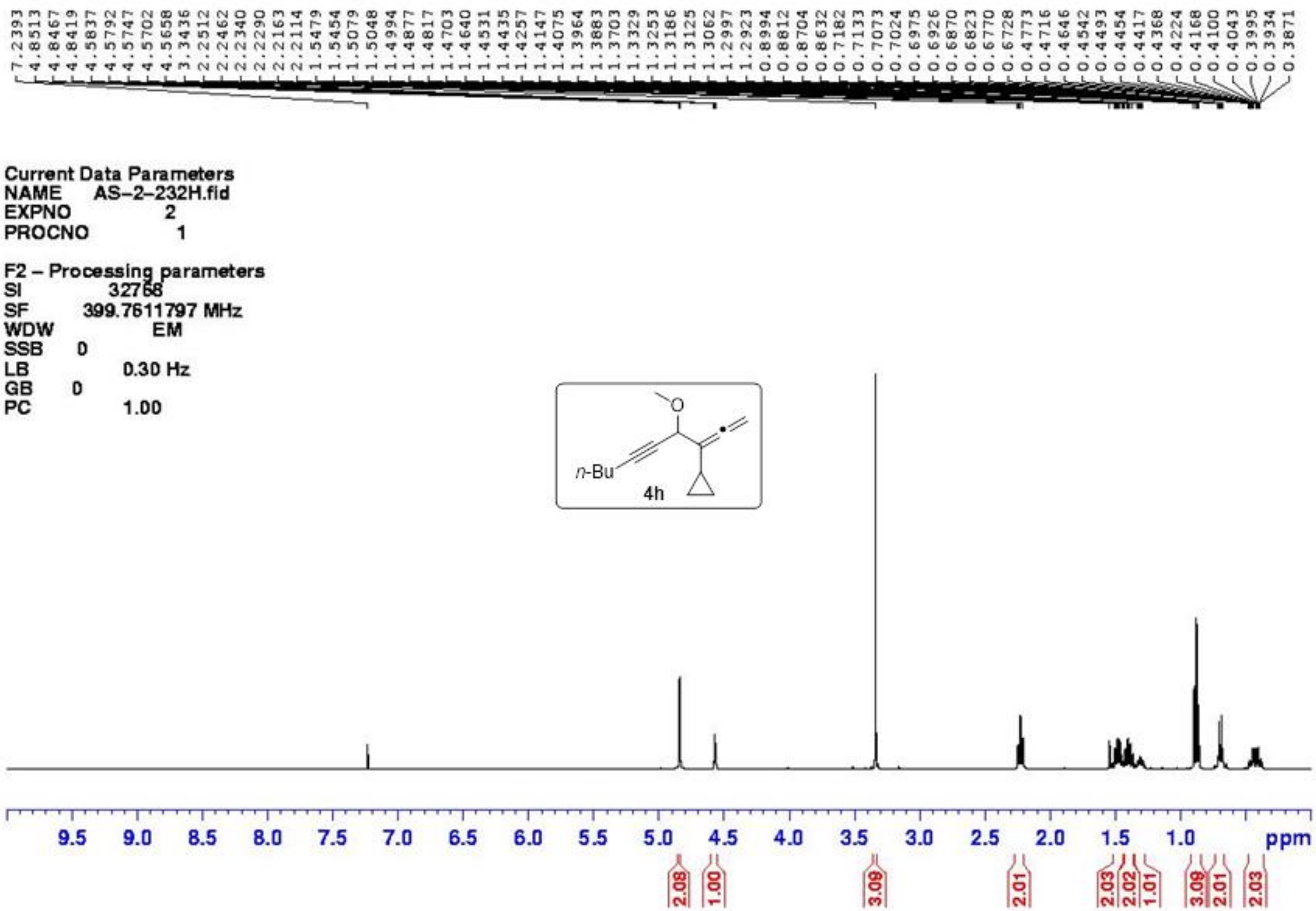
7.2393
4.8513
4.8467
4.8419
4.5837
4.5792
4.5747
4.5702
4.5658
3.3436
2.2512
2.2462
2.2340
2.2290
2.2163
2.2114
1.5479
1.5454
1.5079
1.5048
1.4994
1.4877
1.4817
1.4703
1.4640
1.4531
1.4435
1.4257
1.4147
1.4075
1.3964
1.3883
1.3703
1.3329
1.3253
1.3186
1.3125
1.3062
1.2997
1.2923
0.8994
0.8812
0.8704
0.8632
0.7182
0.7133
0.7073
0.7024
0.6975
0.6926
0.6870
0.6823
0.6770
0.6728
0.4773
0.4716
0.4646
0.4542
0.4493
0.4454
0.4417
0.4368
0.4224
0.4168
0.4100
0.4043
0.3995
0.3934
0.3871
Current Data Parameters
NAME AS-2-232H.fid
EXPNO 2
PROCNO 1
F2 - Processing parameters
SI 32768
SF 399.7611797 MHz
WDW EM
SSB 0
LB 0.30 Hz
GB 0
PC 1.00
O
n-Bu
4h
9.5 9.0 8.5 8.0 7.5 7.0 6.5 6.0 5.5 5.0 4.5 4.0 3.5 3.0 2.5 2.0 1.5 1.0 ppm
2.08
1.00
3.09
2.01
2.03
2.02
1.01
3.09
2.01
2.03

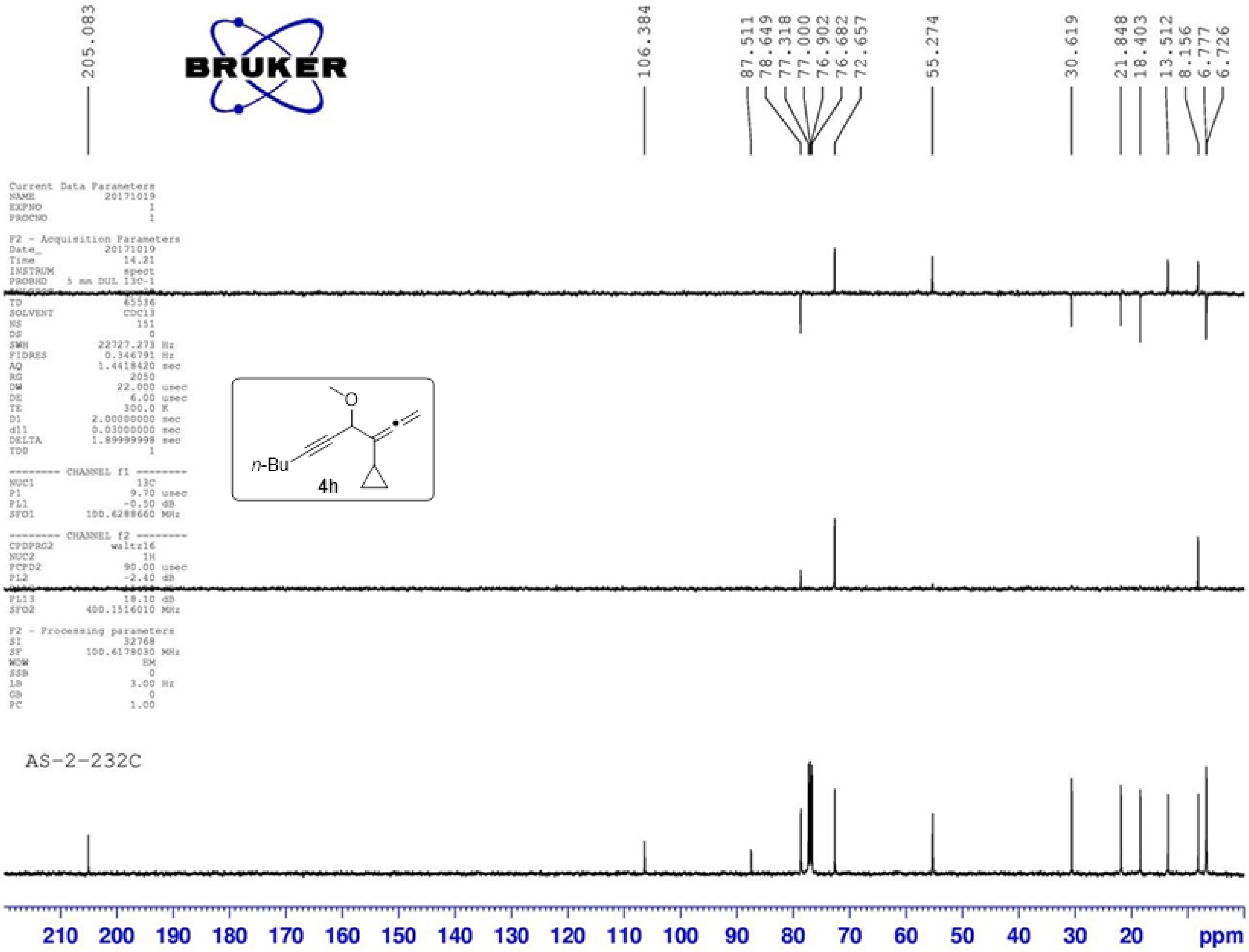
205.083
106.384
87.511
78.649
77.318
77.000
76.902
76.682
72.657
55.274
30.619
21.848
18.403
13.512
8.156
6.777
6.726
Current Data Parameters
NAME 20171019
EXPNO 1
PROCNO 1
F2 - Acquisition Parameters
Date_ 20171019
Time 14.21
INSTRUM spect
PROBHD 5 mm DUL 13C-1
TD 65536
SOLVENT CDCl3
NS 151
DS 0
SWH 22727.273 Hz
FIDRES 0.346791 Hz
AQ 1.4418420 sec
RG 2050
DW 22.000 usec
DE 6.00 usec
TE 300.0 K
D1 2.00000000 sec
d11 0.03000000 sec
DELTA 1.89999998 sec
TD0 1
======== CHANNEL f1 ========
NUC1 13C
P1 9.70 usec
PL1 -0.50 dB
SFO1 100.6288660 MHz
======== CHANNEL f2 ========
CPDPRG2 waltz16
NUC2 1H
PCPD2 90.00 usec
PL2 -2.40 dB
PL13 18.10 dB
SFO2 400.1516010 MHz
F2 - Processing parameters
SI 32768
SF 100.6178030 MHz
WDW EM
SSB 0
LB 3.00 Hz
GB 0
PC 1.00
O
n-Bu
4h
AS-2-232C
210 200 190 180 170 160 150 140 130 120 110 100 90 80 70 60 50 40 30 20 ppm

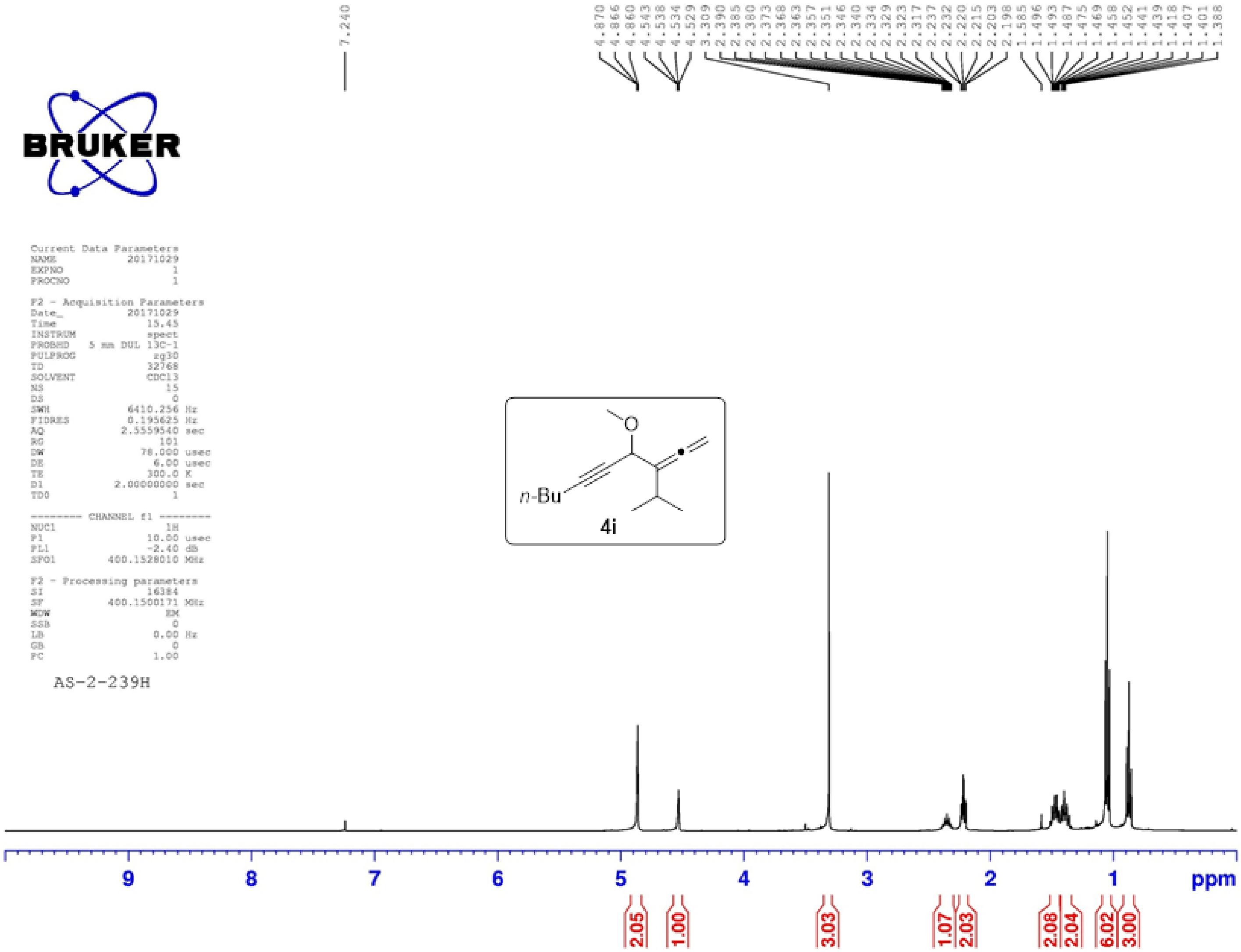
7.240
4.870
4.866
4.860
4.543
4.538
4.534
4.529
3.309
2.390
2.385
2.380
2.373
2.368
2.363
2.357
2.351
2.346
2.340
2.334
2.329
2.323
2.317
2.237
2.232
2.220
2.215
2.203
2.198
1.585
1.496
1.493
1.487
1.475
1.469
1.458
1.452
1.441
1.439
1.418
1.407
1.401
1.388
BRUKER
Current Data Parameters
NAME 20171029
EXPNO 1
PROCNO 1
F2 - Acquisition Parameters
Date_ 20171029
Time 13.45
INSTRUM spect
PROBHD 5 mm DUL 13C-1
PULPROG zg30
TD 32768
SOLVENT CDCl3
NS 15
DS 0
SWH 6410.256 Hz
FIDRES 0.195625 Hz
AQ 2.5559540 sec
RG 101
DW 78.000 usec
DE 6.00 usec
TE 300.0 K
D1 2.00000000 sec
TD0 1
======== CHANNEL f1 ========
NUC1 1H
P1 10.00 usec
PL1 -2.40 dB
SFO1 400.1528010 MHz
F2 - Processing parameters
SI 16384
SF 400.1500171 MHz
WDW EM
SSB 0
LB 0.00 Hz
GB 0
PC 1.00
AS-2-239H
O
n-Bu
4i
9
8
7
6
5
4
3
2
1
ppm
2.05
1.00
3.03
1.07
2.03
2.08
2.04
6.02
3.00

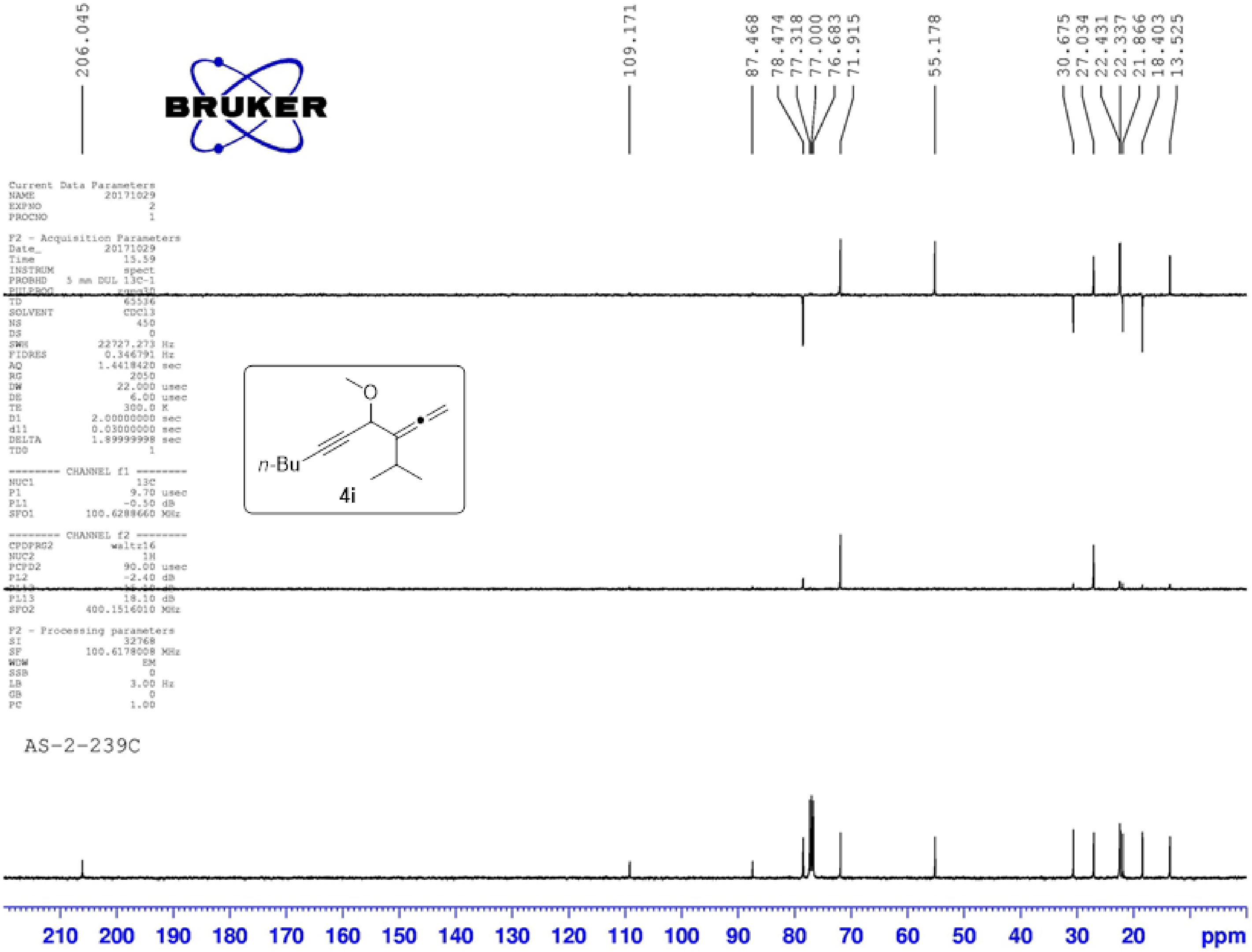
206.045
109.171
87.468
78.474
77.318
77.000
76.683
71.915
55.178
30.675
27.034
22.431
22.337
21.866
18.403
13.525
Current Data Parameters
NAME 20171029
EXPNO 2
PROCNO 1
F2 - Acquisition Parameters
Date_ 20171029
Time 15.59
INSTRUM spect
PROBHD 5 mm DUL 13C-1
TD 65536
SOLVENT CDCl3
NS 450
DS 0
SWH 22727.273 Hz
FIDRES 0.346791 Hz
AQ 1.4418420 sec
RG 2050
DW 22.000 usec
DE 6.00 usec
TE 300.0 K
D1 2.00000000 sec
d11 0.03000000 sec
DELTA 1.89999998 sec
TD0 1
======== CHANNEL f1 ========
NUC1 13C
P1 9.70 usec
PL1 -0.30 dB
SFO1 100.6288660 MHz
======== CHANNEL f2 ========
CPDPRG2 waltz16
NUC2 1H
PCPD2 90.00 usec
PL2 -2.40 dB
PL13 18.10 dB
SFO2 400.1516010 MHz
F2 - Processing parameters
SI 32768
SF 100.6178008 MHz
WDW EM
SSB 0
LB 3.00 Hz
GB 0
PC 1.00
AS-2-239C
O
n-Bu
4i
210
200
190
180
170
160
150
140
130
120
110
100
90
80
70
60
50
40
30
20
ppm

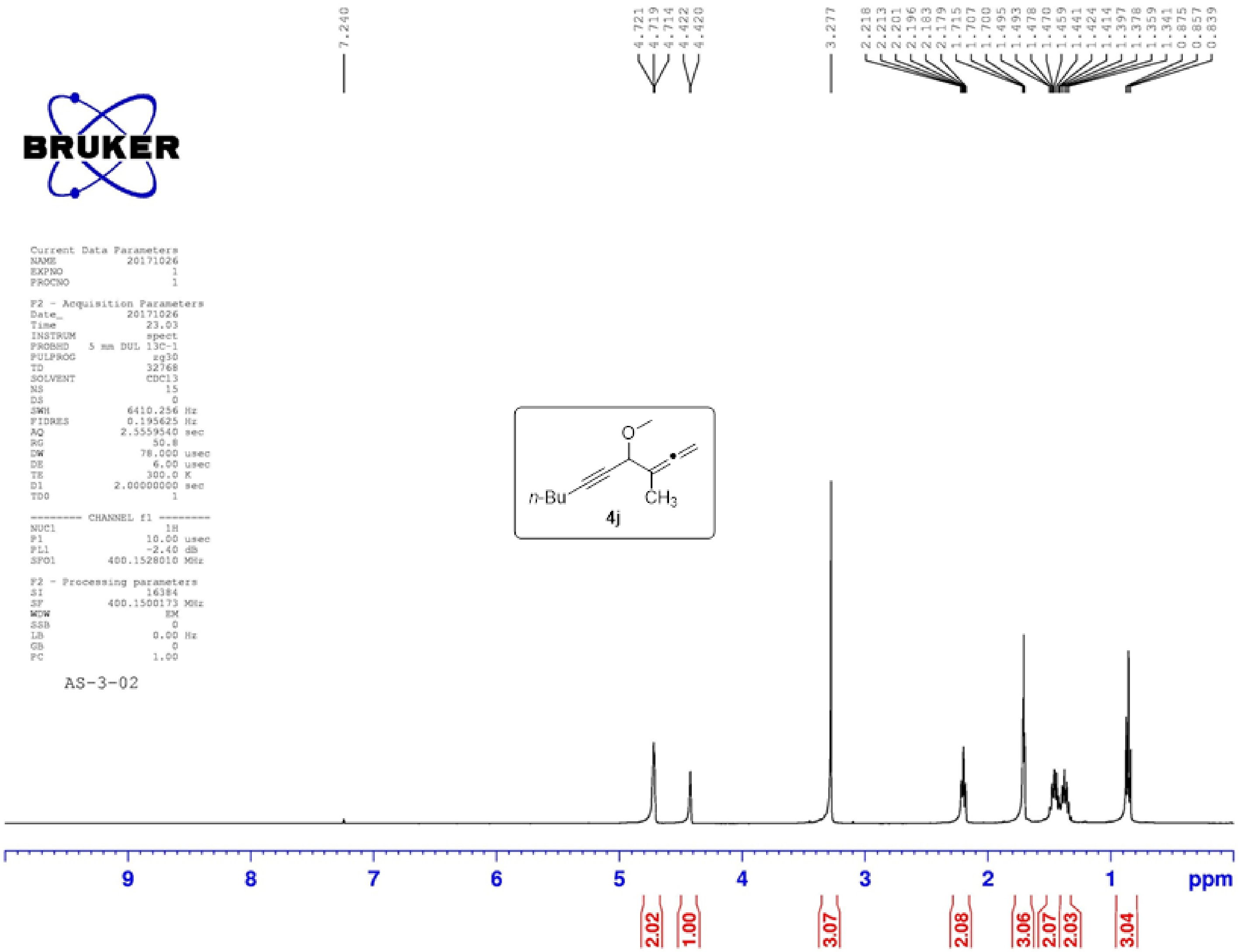
7.240
4.721
4.719
4.714
4.422
4.420
3.277
2.218
2.213
2.201
2.196
2.183
2.179
1.715
1.707
1.700
1.495
1.493
1.478
1.470
1.459
1.441
1.424
1.414
1.397
1.378
1.359
1.341
0.875
0.857
0.839
BRUKER
Current Data Parameters
NAME 20171026
EXPNO 1
PROCNO 1
F2 - Acquisition Parameters
Date_ 20171026
Time 23.03
INSTRUM spect
PROBHD 5 mm DUL 13C-1
PULPROG zg30
TD 32768
SOLVENT CDCl3
NS 15
DS 0
SWH 6410.256 Hz
FIDRES 0.195625 Hz
AQ 2.5559540 sec
RG 50.8
DW 78.000 usec
DE 6.00 usec
TE 300.0 K
D1 2.00000000 sec
TD0 1
======== CHANNEL f1 ========
NUC1 1H
P1 10.00 usec
PL1 -2.40 dB
SFO1 400.1528010 MHz
F2 - Processing parameters
SI 16384
SF 400.1500173 MHz
WDW EM
SSB 0
LB 0.00 Hz
GB 0
PC 1.00
AS-3-02
O
n-Bu
CH3
4j
9
8
7
6
5
4
3
2
1
ppm
2.02
1.00
3.07
2.08
3.06
2.07
2.03
3.04

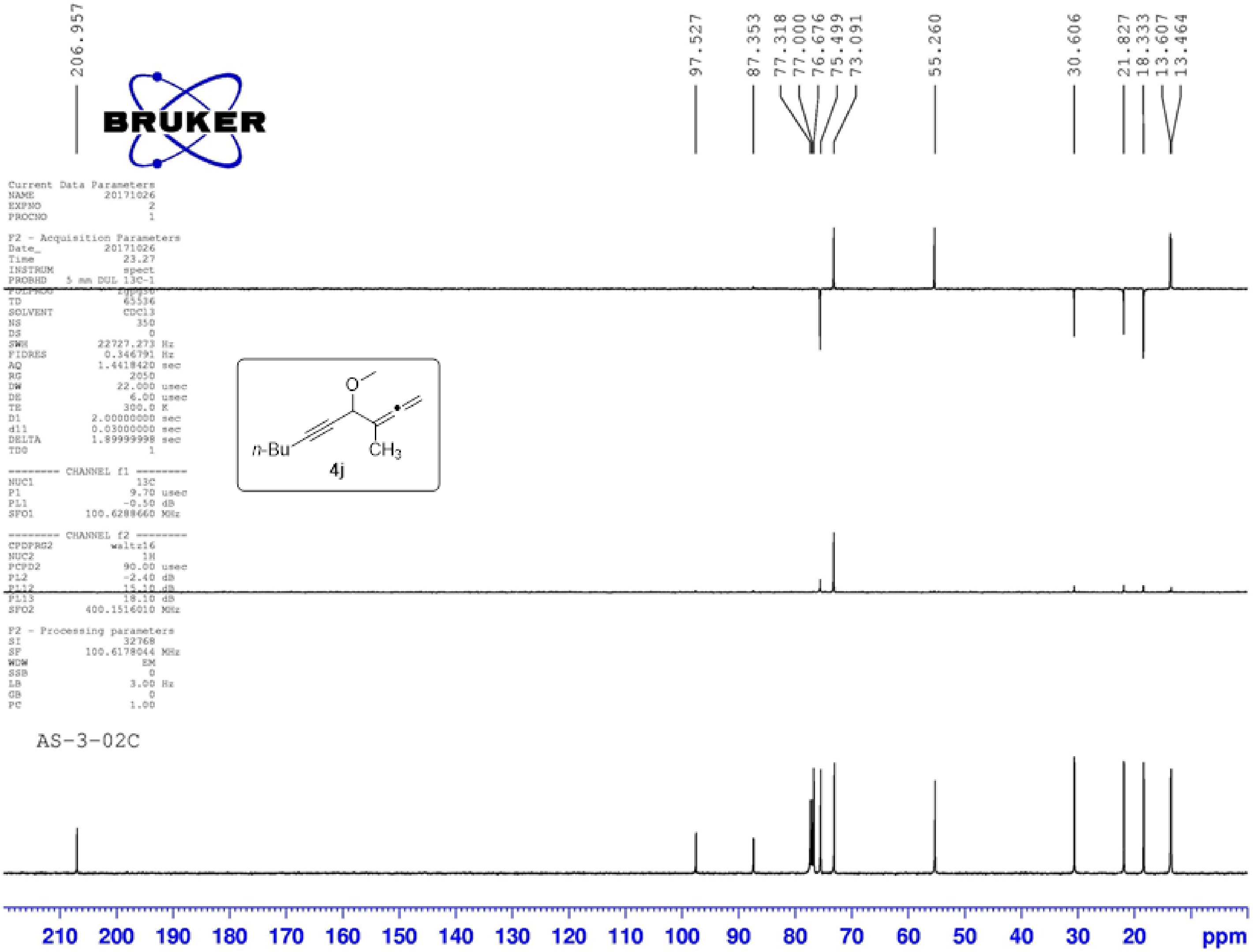
206.957
97.527
87.353
77.318
77.000
76.676
75.499
73.091
55.260
30.606
21.827
18.333
13.607
13.464
Current Data Parameters
NAME 20171026
EXPNO 2
PROCNO 1
F2 - Acquisition Parameters
Date_ 20171026
Time 23.27
INSTRUM spect
PROBHD 5 mm DUL 13C-1
TD 65536
SOLVENT CDCl3
NS 350
DS 0
SWH 22727.273 Hz
FIDRES 0.346791 Hz
AQ 1.4418420 sec
RG 2050
DW 22.000 usec
DE 6.00 usec
TE 300.0 K
D1 2.00000000 sec
d11 0.03000000 sec
DELTA 1.89999998 sec
TD0 1
======== CHANNEL f1 ========
NUC1 13C
P1 9.70 usec
PL1 -0.50 dB
SFO1 100.6288660 MHz
======== CHANNEL f2 ========
CPDPRG2 waltz16
NUC2 1H
PCPD2 90.00 usec
PL2 -2.40 dB
PL12 15.10 dB
PL13 18.10 dB
SFO2 400.1516010 MHz
F2 - Processing parameters
SI 32768
SF 100.6178044 MHz
WDW EM
SSB 0
LB 3.00 Hz
GB 0
PC 1.00
AS-3-02C
n-Bu
O
$CH_3$
4j
210 200 190 180 170 160 150 140 130 120 110 100 90 80 70 60 50 40 30 20 ppm

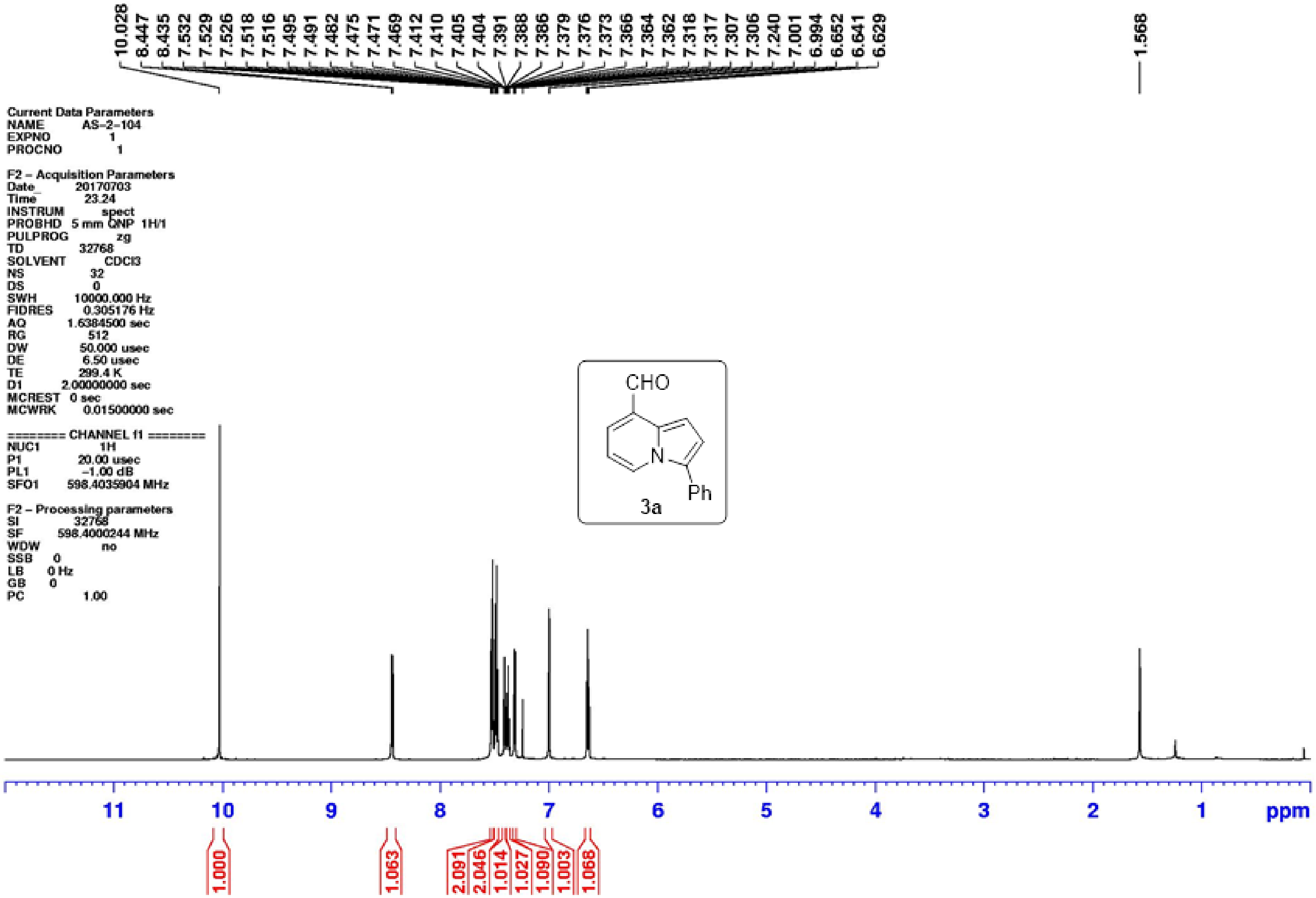
10.028
8.447
8.435
7.532
7.529
7.526
7.518
7.516
7.495
7.491
7.482
7.475
7.471
7.469
7.412
7.410
7.405
7.404
7.391
7.388
7.386
7.379
7.376
7.373
7.366
7.364
7.362
7.318
7.317
7.307
7.306
7.240
7.001
6.994
6.652
6.641
6.629
1.568
Current Data Parameters
NAME AS-2-104
EXPNO 1
PROCNO 1
F2 - Acquisition Parameters
Date_ 20170703
Time 23.24
INSTRUM spect
PROBHD 5 mm QNP 1H/1
PULPROG zg
TD 32768
SOLVENT CDCl3
NS 32
DS 0
SWH 10000.000 Hz
FIDRES 0.305176 Hz
AQ 1.6384500 sec
RG 512
DW 50.000 usec
DE 6.50 usec
TE 299.4 K
D1 2.00000000 sec
MCREST 0 sec
MCWRK 0.01500000 sec
======== CHANNEL f1 ========
NUC1 1H
P1 20.00 usec
PL1 -1.00 dB
SFO1 598.4035904 MHz
F2 - Processing parameters
SI 32768
SF 598.4000244 MHz
WDW no
SSB 0
LB 0 Hz
GB 0
PC 1.00
CHO
N
Ph
3a
11
10
9
8
7
6
5
4
3
2
1
ppm
1.000
1.063
2.091
2.046
1.014
1.027
1.090
1.003
1.068

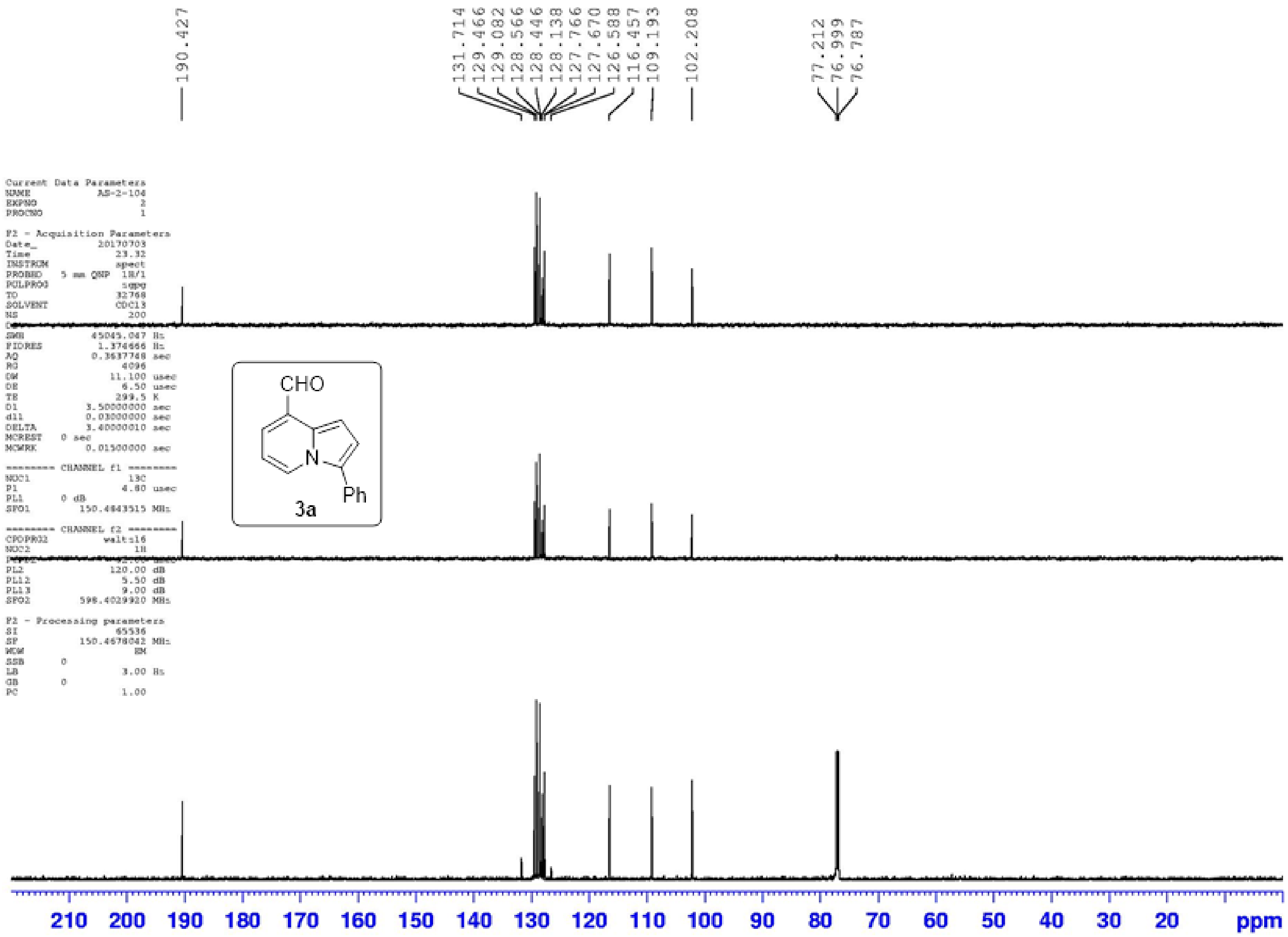
190.427
131.714
129.466
129.082
128.566
128.446
128.138
127.766
127.670
126.588
116.457
109.193
102.208
77.212
76.999
76.787
Current Data Parameters
NAME AS-2-104
EXPNO 2
PROCNO 1
F2 - Acquisition Parameters
Date_ 20170703
Time 23.32
INSTRUM spect
PROBHD 5 mm QNP 1H/1
TD 32768
SOLVENT CDCl3
NS 200
SWH 45045.047 Hz
FIDRES 1.374666 Hz
AQ 0.3637748 sec
RG 4096
DW 11.100 usec
DE 6.50 usec
TE 299.5 K
D1 3.50000000 sec
d11 0.03000000 sec
DELTA 3.40000010 sec
MCREST 0 sec
MCWRK 0.01500000 sec
======== CHANNEL f1 ========
NUC1 13C
P1 4.80 usec
PL1 0 dB
SFO1 150.4843515 MHz
======== CHANNEL f2 ========
CPDPRG2 waltz16
NUC2 1H
PL2 120.00 dB
PL12 5.50 dB
PL13 9.00 dB
SFO2 598.4029920 MHz
F2 - Processing parameters
SI 65536
SF 150.4678042 MHz
WDW EM
SSB 0
LB 3.00 Hz
GB 0
PC 1.00
CHO
N
Ph
3a
210 200 190 180 170 160 150 140 130 120 110 100 90 80 70 60 50 40 30 20 ppm

<sup></sup>

# $^{1}$H NOE of compound **3a**

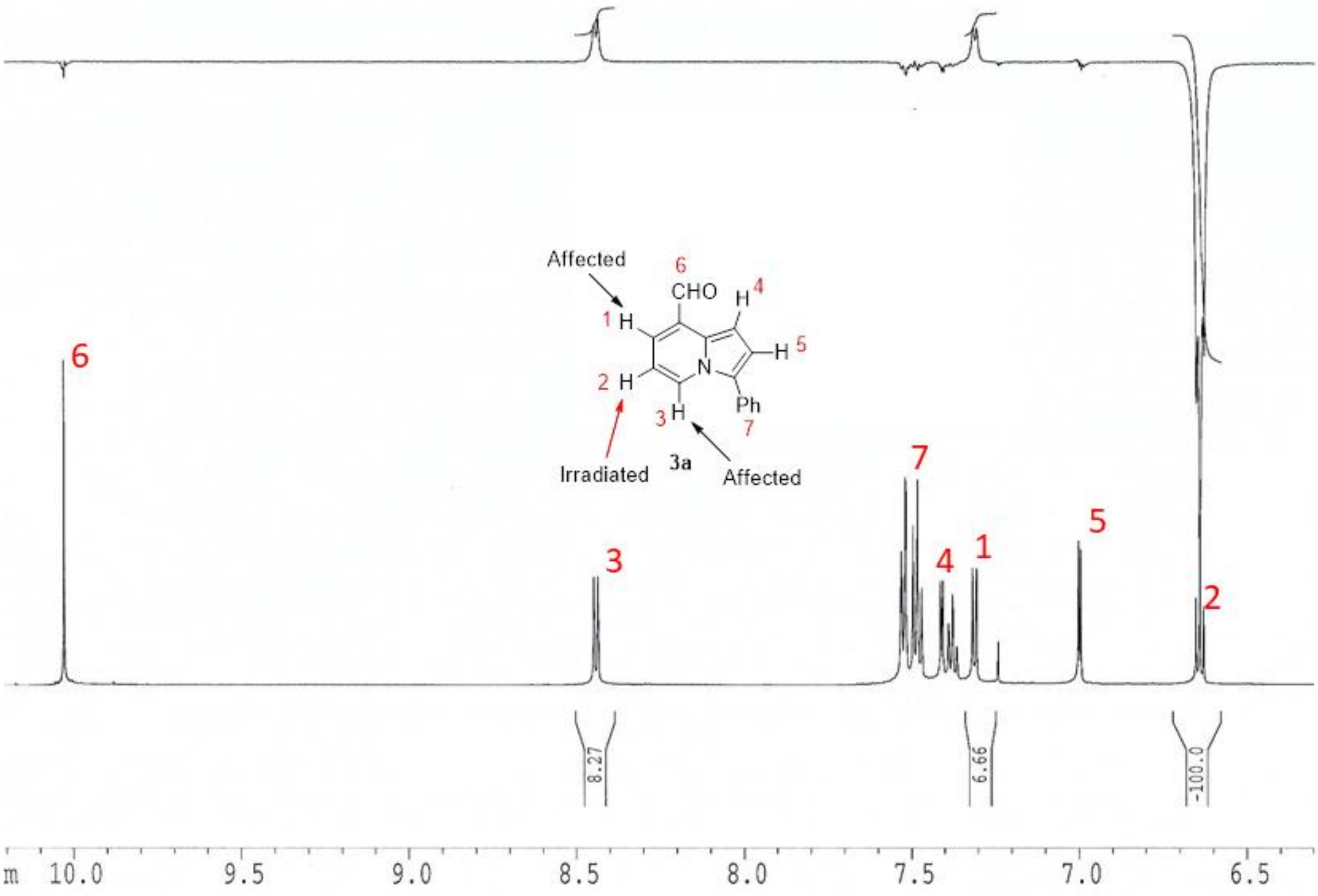

## $^1H$ NOE of compound **3a**

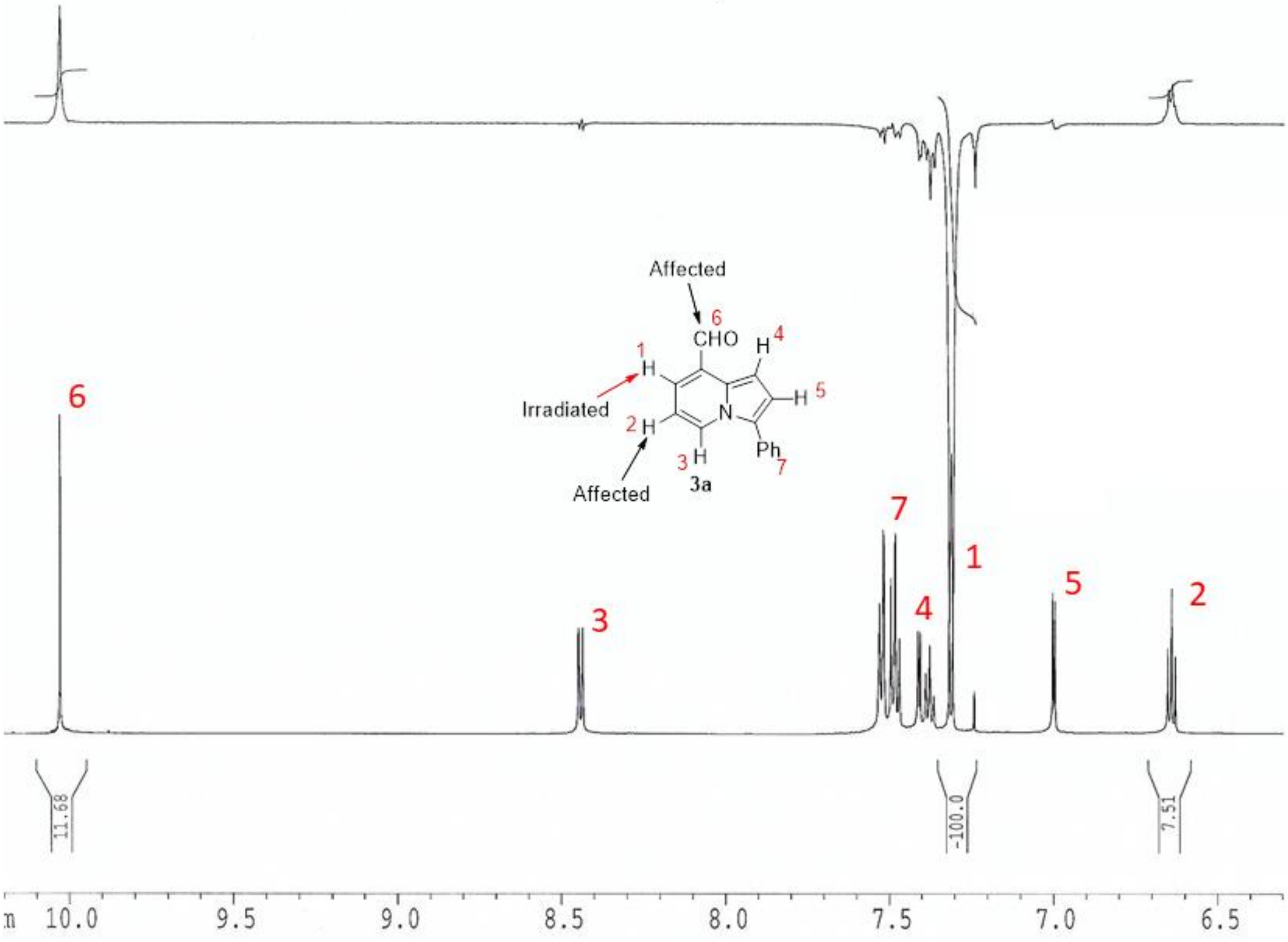

## $^1H$ NOE of compound **3a**

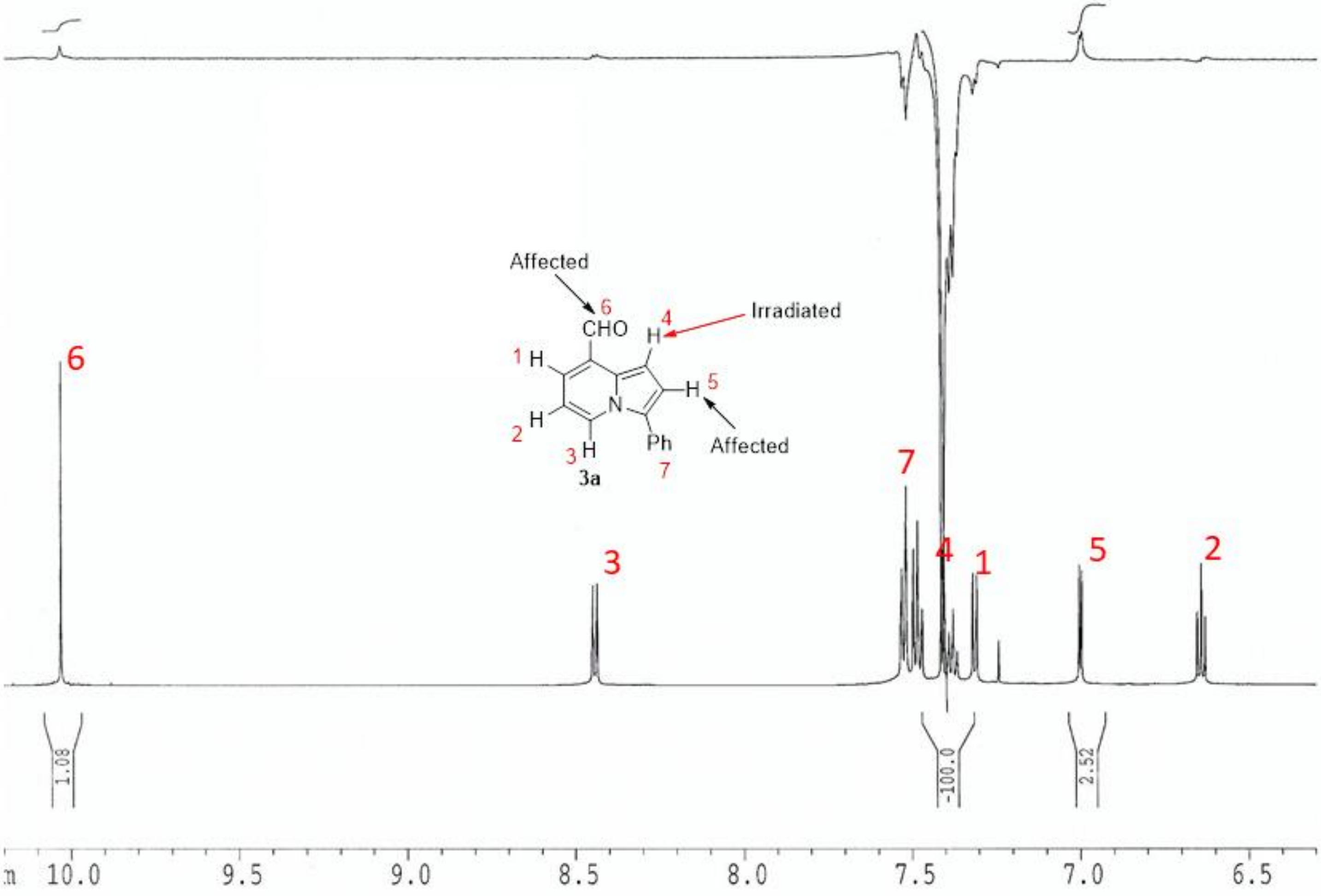

# $^1$H NOE of compound **3a**

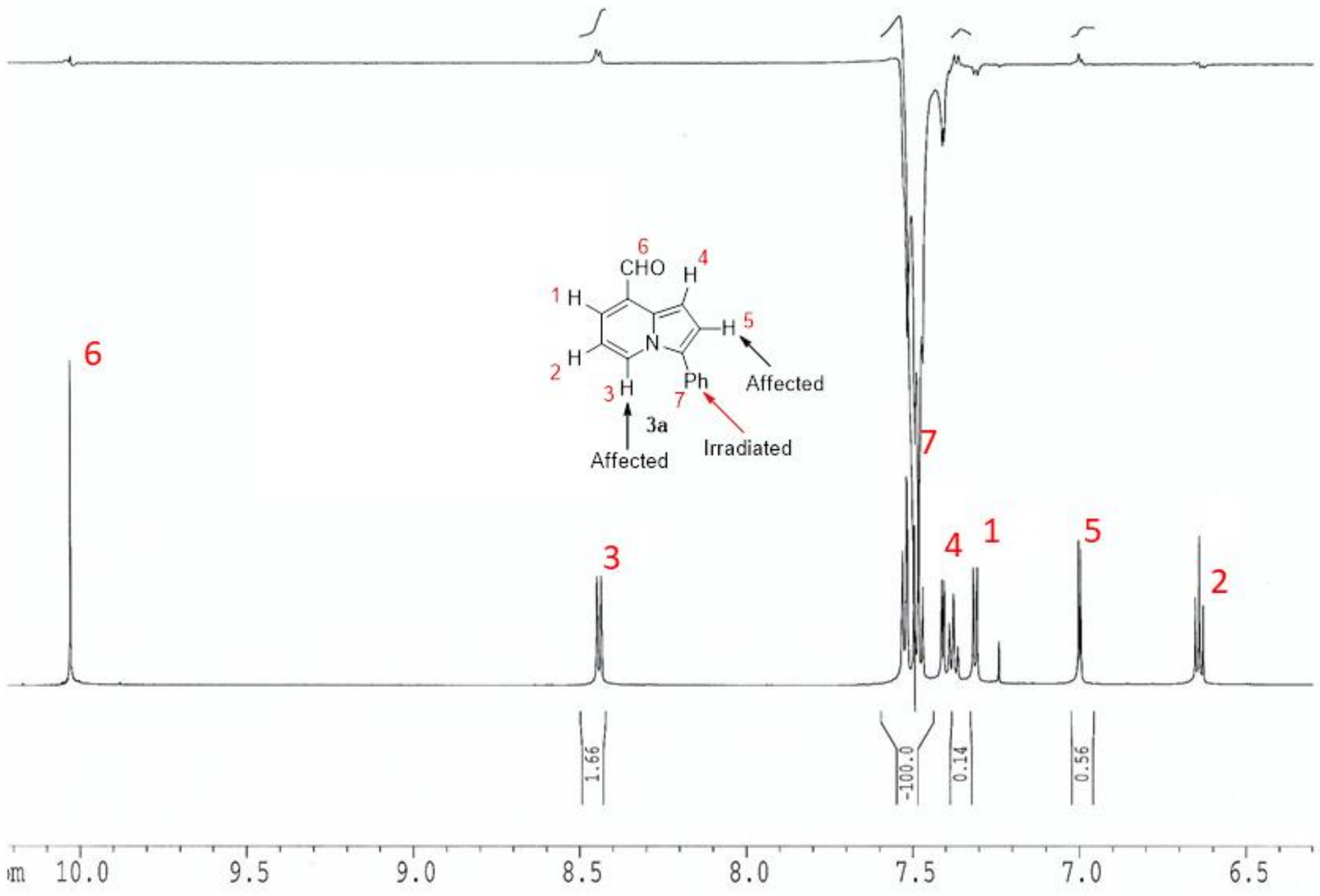

$^1H$ NOE of compound **3a**

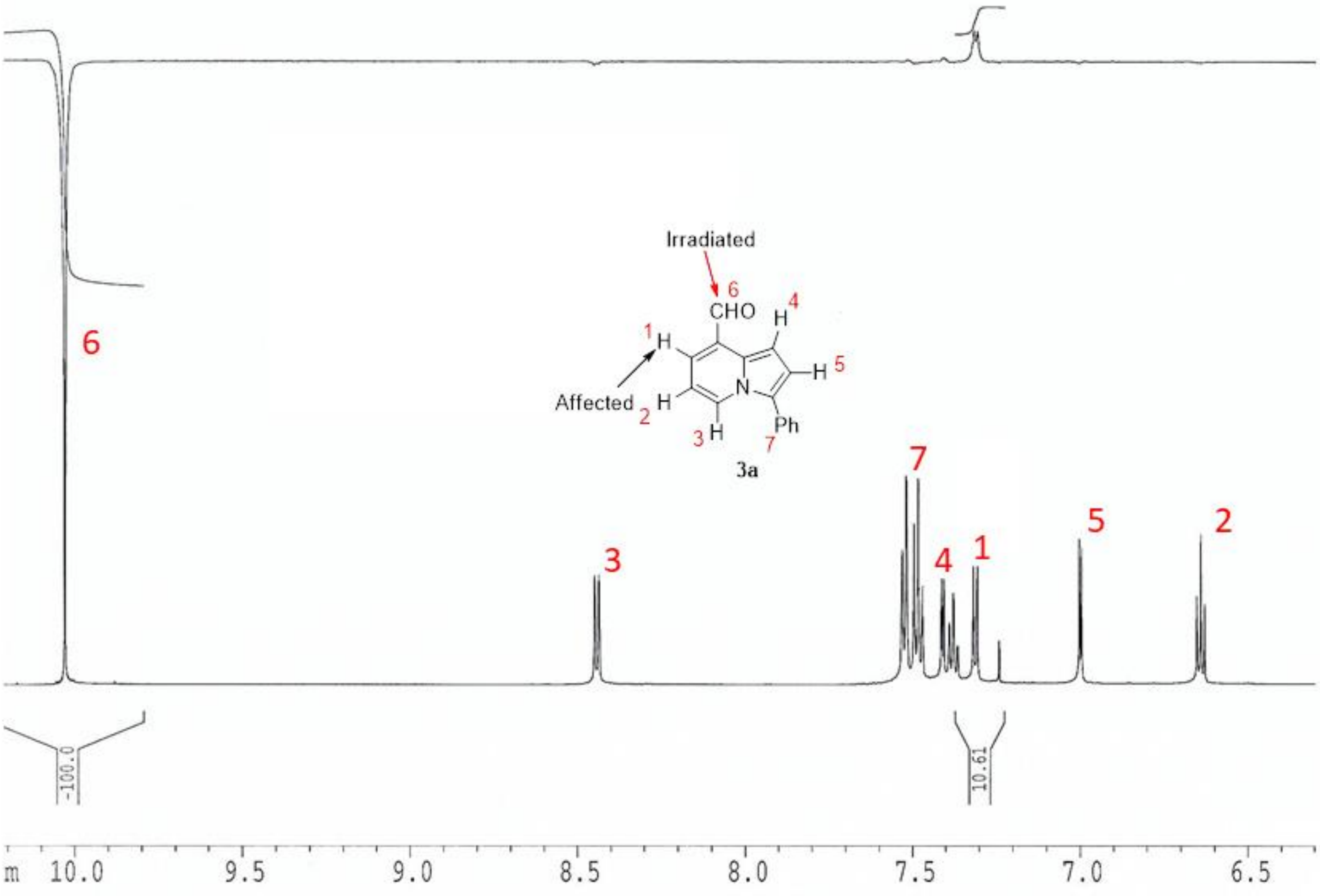

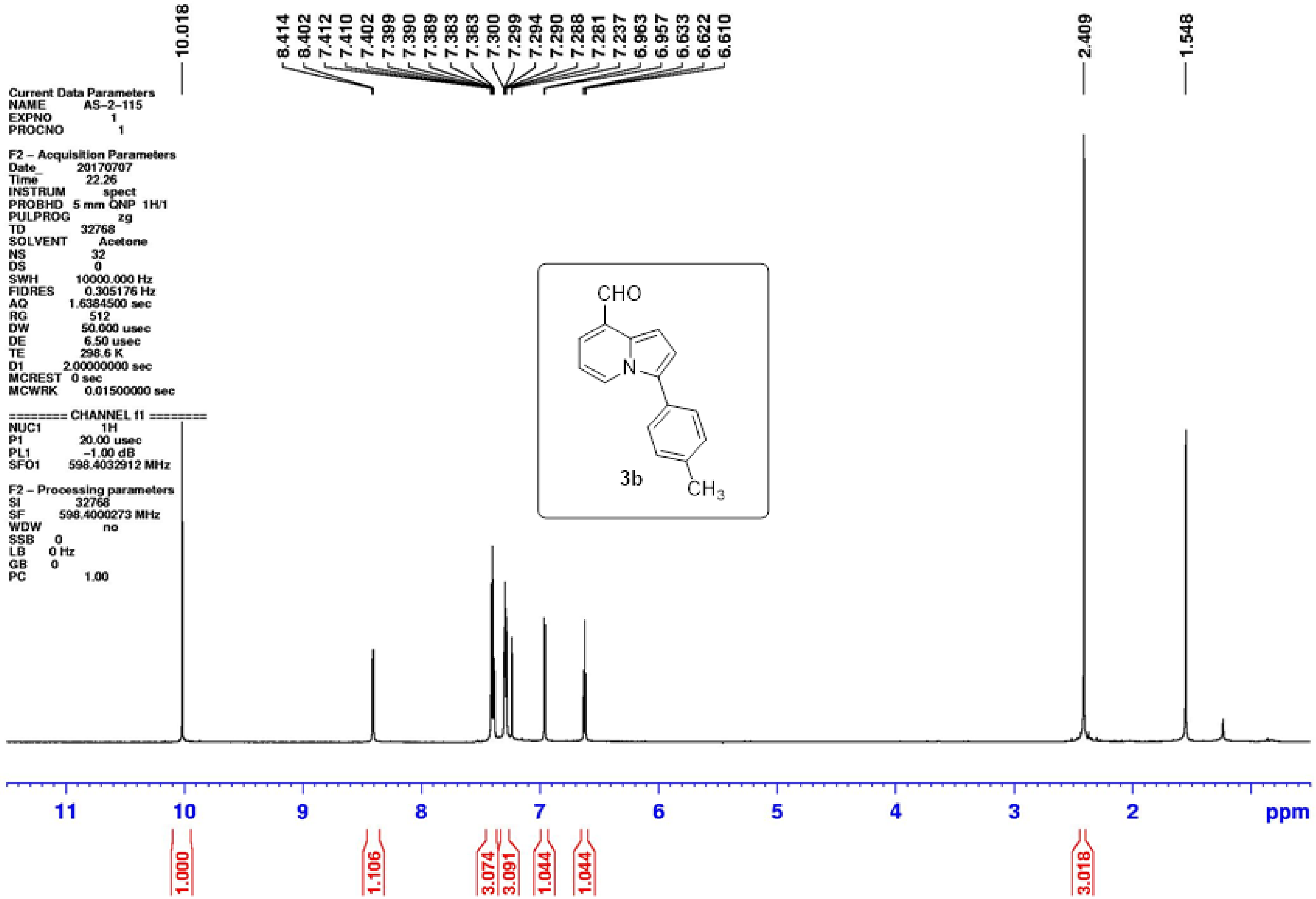
10.018
8.414
8.402
7.412
7.410
7.402
7.399
7.390
7.389
7.383
7.363
7.300
7.299
7.294
7.290
7.288
7.281
7.237
6.963
6.957
6.633
6.622
6.610
2.409
1.548
Current Data Parameters
NAME AS-2-115
EXPNO 1
PROCNO 1
F2 - Acquisition Parameters
Date_ 20170707
Time 22.26
INSTRUM spect
PROBHD 5 mm QNP 1H/1
PULPROG zg
TD 32768
SOLVENT Acetone
NS 32
DS 0
SWH 10000.000 Hz
FIDRES 0.305176 Hz
AQ 1.6384500 sec
RG 512
DW 50.000 usec
DE 6.50 usec
TE 298.6 K
D1 2.00000000 sec
MCREST 0 sec
MCWRK 0.01500000 sec
======= CHANNEL f1 =======
NUC1 1H
P1 20.00 usec
PL1 -1.00 dB
SFO1 598.4032912 MHz
F2 - Processing parameters
SI 32768
SF 598.4000273 MHz
WDW no
SSB 0
LB 0 Hz
GB 0
PC 1.00
CHO
N
3b
CH3
11
10
9
8
7
6
5
4
3
2
ppm
1.000
1.106
3.074
3.091
1.044
1.044
3.018

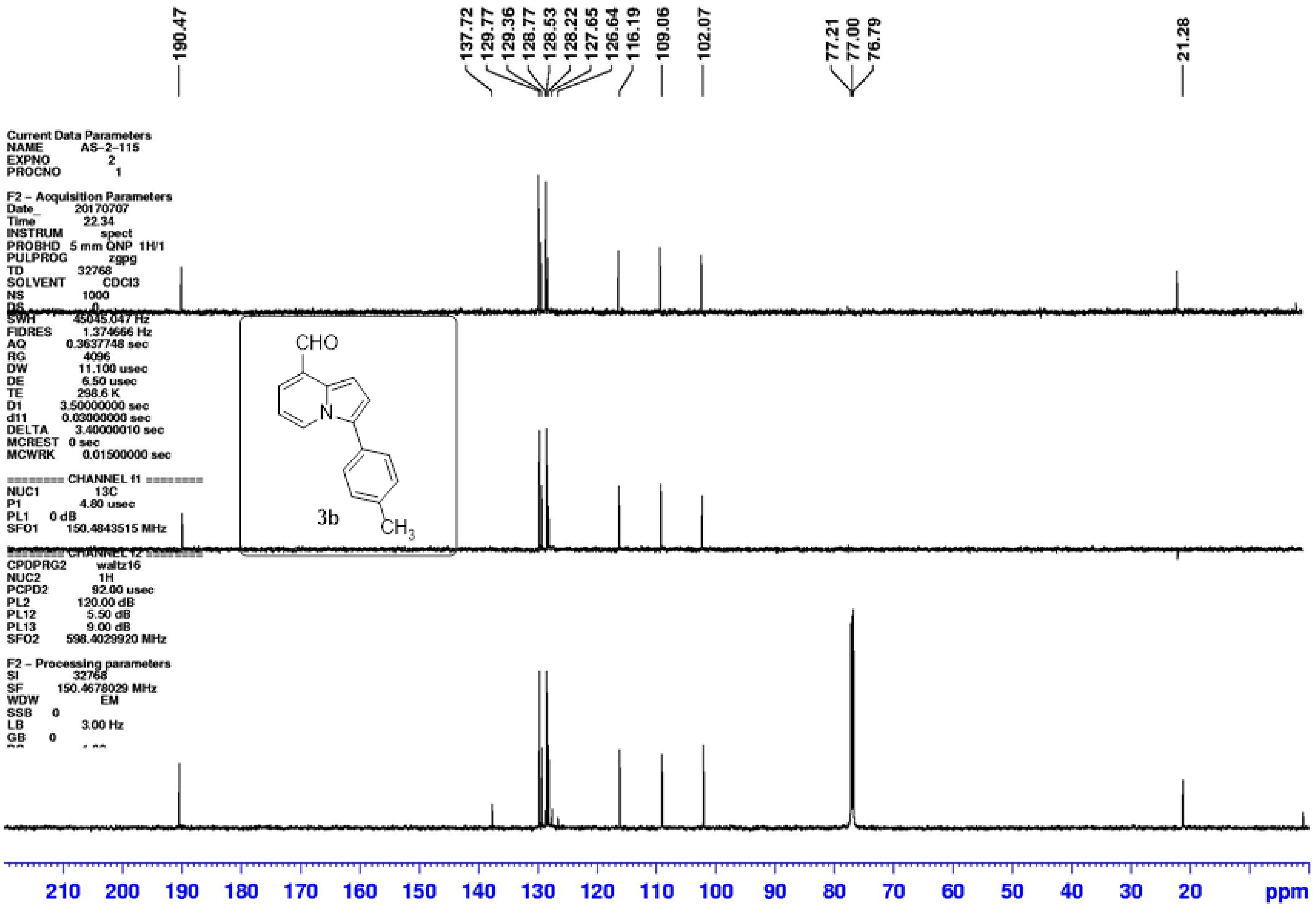
190.47
137.72
129.77
129.36
128.77
128.53
128.22
127.65
126.64
116.19
109.06
102.07
77.21
77.00
76.79
21.28
Current Data Parameters
NAME AS-2-115
EXPNO 2
PROCNO 1
F2 - Acquisition Parameters
Date_ 20170707
Time 22.34
INSTRUM spect
PROBHD 5 mm QNP 1H/1
PULPROG zgpg
TD 32768
SOLVENT CDCl3
NS 1000
SWH 45045.047 Hz
FIDRES 1.374666 Hz
AQ 0.3637748 sec
RG 4096
DW 11.100 usec
DE 6.50 usec
TE 298.6 K
D1 3.50000000 sec
d11 0.03000000 sec
DELTA 3.40000010 sec
MCREST 0 sec
MCWRK 0.01500000 sec
======== CHANNEL f1 ========
NUC1 13C
P1 4.80 usec
PL1 0 dB
SFO1 150.4843515 MHz
CPDPRG2 waltz16
NUC2 1H
PCPD2 92.00 usec
PL2 120.00 dB
PL12 5.50 dB
PL13 9.00 dB
SFO2 598.4029920 MHz
F2 - Processing parameters
SI 32768
SF 150.4678029 MHz
WDW EM
SSB 0
LB 3.00 Hz
GB 0
CHO
N
3b
CH3
210 200 190 180 170 160 150 140 130 120 110 100 90 80 70 60 50 40 30 20 ppm

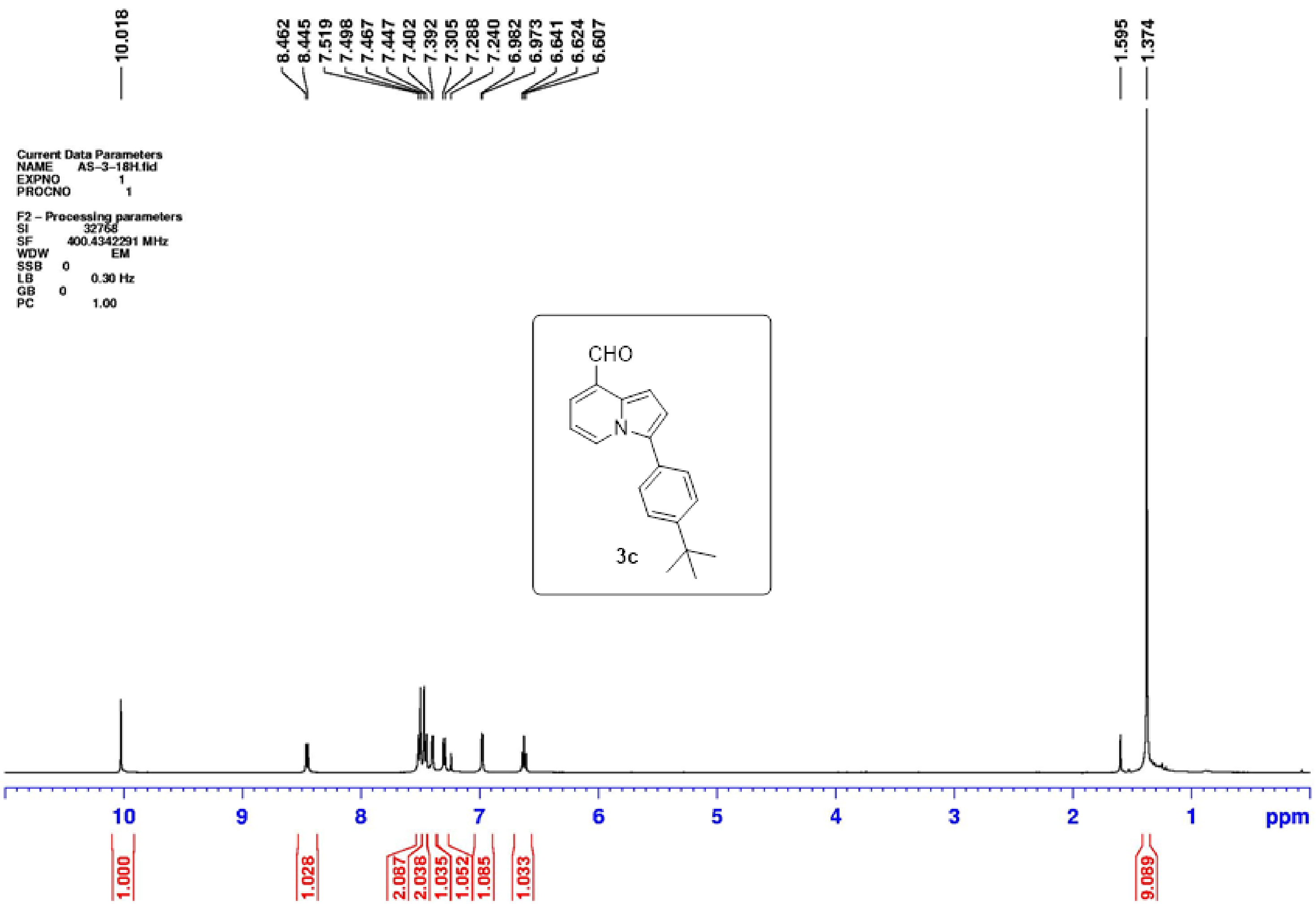
10.018
8.462
8.445
7.519
7.498
7.467
7.447
7.402
7.392
7.305
7.288
7.240
6.982
6.973
6.641
6.624
6.607
1.595
1.374
Current Data Parameters
NAME AS-3-18H.fid
EXPNO 1
PROCNO 1
F2 - Processing parameters
SI 32768
SF 400.4342291 MHz
WDW EM
SSB 0
LB 0.30 Hz
GB 0
PC 1.00
CHO
N
3c
10
9
8
7
6
5
4
3
2
1
ppm
1.000
1.028
2.087
2.038
1.035
1.052
1.085
1.033
9.089

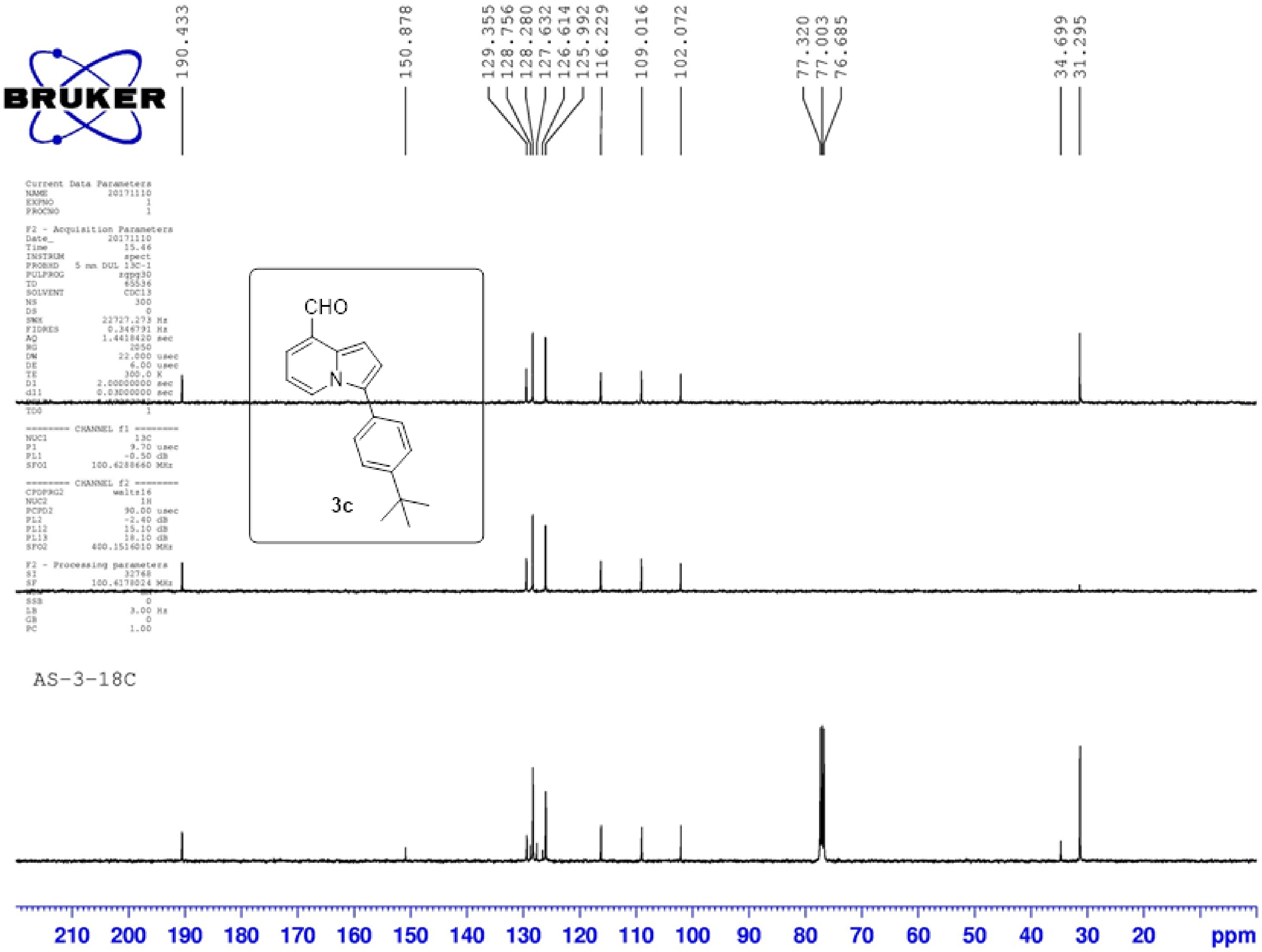

BRUKER
190.433
150.878
129.355
128.756
128.280
127.632
126.614
125.992
116.229
109.016
102.072
77.320
77.003
76.685
34.699
31.295
CHO
N
3c
AS-3-18C
210 200 190 180 170 160 150 140 130 120 110 100 90 80 70 60 50 40 30 20 ppm

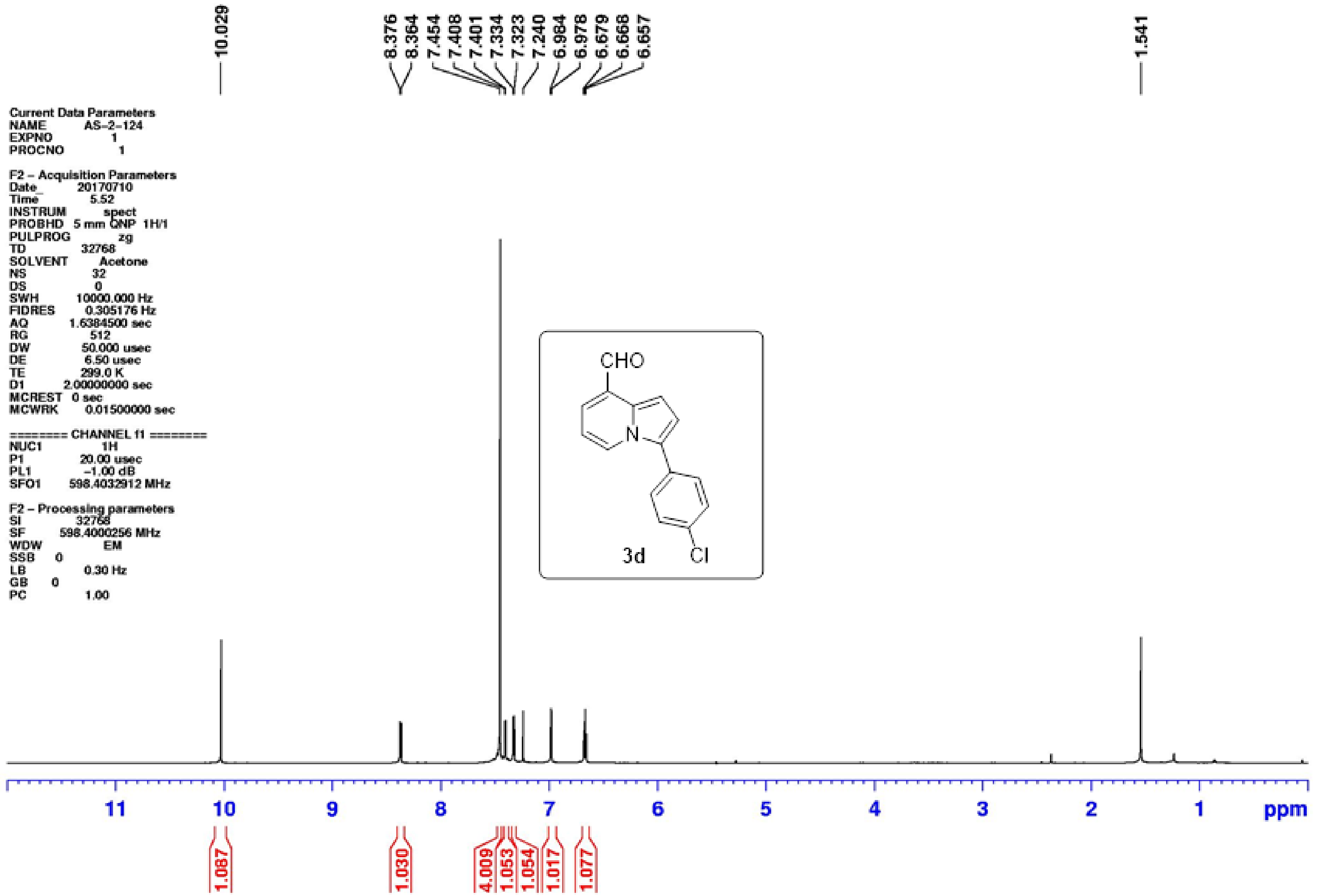
10.029
8.376
8.364
7.454
7.408
7.401
7.334
7.323
7.240
6.984
6.978
6.679
6.668
6.657
1.541
Current Data Parameters
NAME AS-2-124
EXPNO 1
PROCNO 1
F2 - Acquisition Parameters
Date_ 20170710
Time 5.52
INSTRUM spect
PROBHD 5 mm QNP 1H/1
PULPROG zg
TD 32768
SOLVENT Acetone
NS 32
DS 0
SWH 10000.000 Hz
FIDRES 0.305176 Hz
AQ 1.6384500 sec
RG 512
DW 50.000 usec
DE 6.50 usec
TE 299.0 K
D1 2.00000000 sec
MCREST 0 sec
MCWRK 0.01500000 sec
======== CHANNEL f1 ========
NUC1 1H
P1 20.00 usec
PL1 -1.00 dB
SFO1 598.4032912 MHz
F2 - Processing parameters
SI 32768
SF 598.4000256 MHz
WDW EM
SSB 0
LB 0.30 Hz
GB 0
PC 1.00
CHO
N
3d
Cl
11
10
9
8
7
6
5
4
3
2
1
ppm
1.087
1.030
4.009
1.053
1.054
1.017
1.077

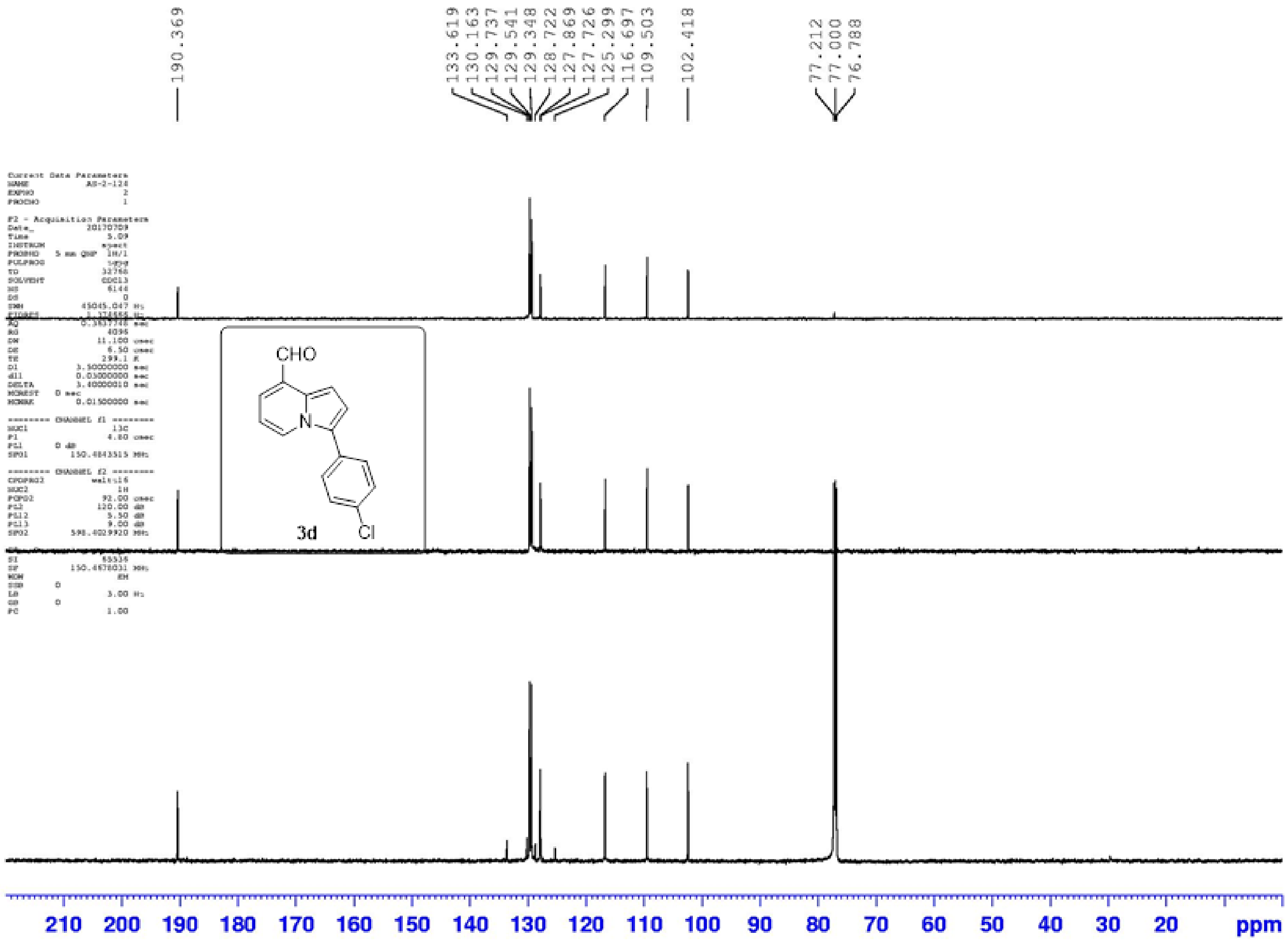

190.369
133.619
130.163
129.737
129.541
129.348
128.722
127.869
127.726
125.299
116.697
109.503
102.418
77.212
77.000
76.788
CHO
N
3d
Cl
210 200 190 180 170 160 150 140 130 120 110 100 90 80 70 60 50 40 30 20 ppm

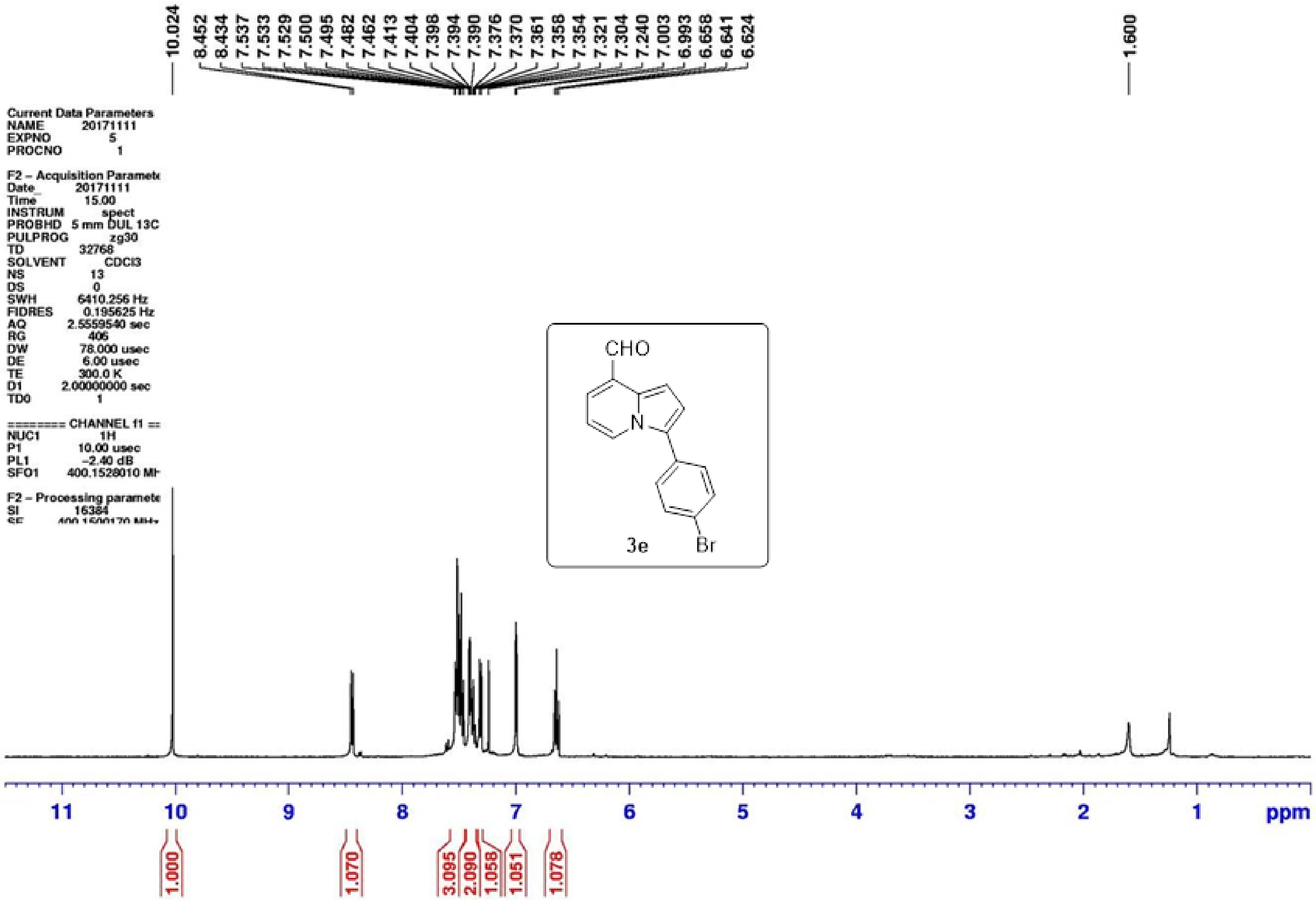
10.024
8.452
8.434
7.537
7.533
7.529
7.500
7.495
7.482
7.462
7.413
7.404
7.398
7.394
7.390
7.376
7.370
7.361
7.358
7.354
7.321
7.304
7.240
7.003
6.993
6.658
6.641
6.624
1.600
Current Data Parameters
NAME 20171111
EXPNO 5
PROCNO 1
F2 – Acquisition Paramete
Date_ 20171111
Time 15.00
INSTRUM spect
PROBHD 5 mm DUL 13C
PULPROG zg30
TD 32768
SOLVENT CDCl3
NS 13
DS 0
SWH 6410.256 Hz
FIDRES 0.195625 Hz
AQ 2.5559540 sec
RG 406
DW 78.000 usec
DE 6.00 usec
TE 300.0 K
D1 2.00000000 sec
TD0 1
======== CHANNEL f1 ==
NUC1 1H
P1 10.00 usec
PL1 -2.40 dB
SFO1 400.1528010 MH
F2 – Processing paramete
SI 16384
CHO
N
3e
Br
11
10
9
8
7
6
5
4
3
2
1
ppm
1.000
1.070
3.095
2.090
1.058
1.051
1.078

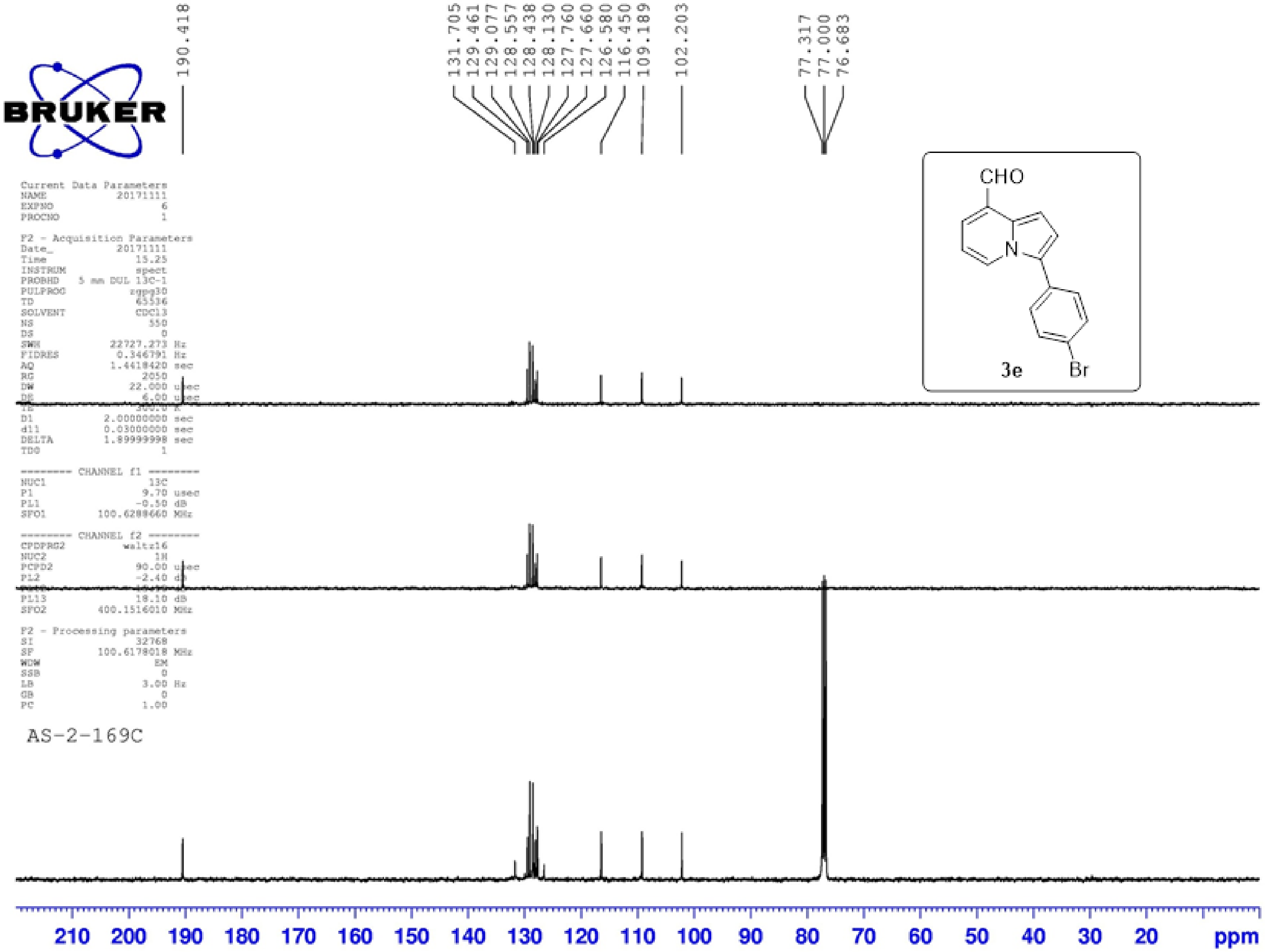
190.418
131.705
129.461
129.077
128.557
128.438
128.130
127.760
127.660
126.580
116.450
109.189
102.203
77.317
77.000
76.683
BRUKER
Current Data Parameters
NAME 20171111
EXPNO 6
PROCNO 1
F2 - Acquisition Parameters
Date_ 20171111
Time 15.25
INSTRUM spect
PROBHD 5 mm DUL 13C-1
PULPROG zgpg30
TD 65536
SOLVENT CDCl3
NS 550
DS 0
SWH 22727.273 Hz
FIDRES 0.346791 Hz
AQ 1.4418420 sec
RG 2050
DW 22.000 usec
DE 6.00 usec
D1 2.00000000 sec
d11 0.03000000 sec
DELTA 1.89999998 sec
TD0 1
======== CHANNEL f1 ========
NUC1 13C
P1 9.70 usec
PL1 -0.30 dB
SFO1 100.6288660 MHz
======== CHANNEL f2 ========
CPDPRG2 waltz16
NUC2 1H
PCPD2 90.00 usec
PL2 -2.40 dB
PL13 18.10 dB
SFO2 400.1516010 MHz
F2 - Processing parameters
SI 32768
SF 100.6178018 MHz
WDW EM
SSB 0
LB 3.00 Hz
GB 0
PC 1.00
AS-2-169C
CHO
N
3e
Br
210 200 190 180 170 160 150 140 130 120 110 100 90 80 70 60 50 40 30 20 ppm

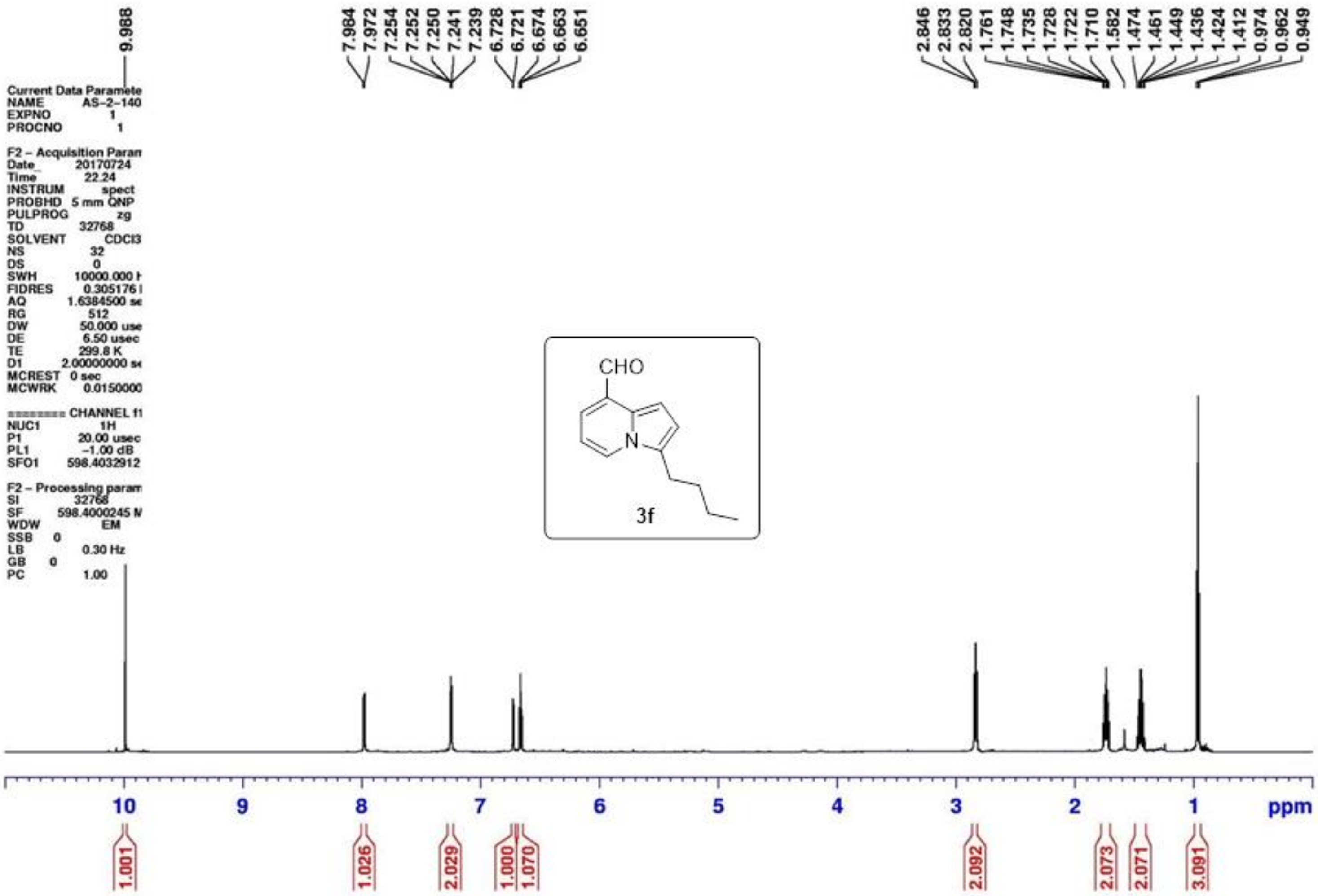
Current Data Paramete
NAME AS-2-140
EXPNO 1
PROCNO 1
F2 - Acquisition Paran
Date_ 20170724
Time_ 22.24
INSTRUM spect
PROBHD 5 mm QNP
PULPROG zg
TD 32768
SOLVENT CDCl3
NS 32
DS 0
SWH 10000.000 H
FIDRES 0.305176 I
AQ 1.6384500 se
RG 512
DW 50.000 use
DE 6.50 usec
TE 299.8 K
D1 2.00000000 se
MCREST 0 sec
MCWRK 0.0150000
======== CHANNEL f1
NUC1 1H
P1 20.00 usec
PL1 -1.00 dB
SFO1 598.4032912
F2 - Processing param
SI 32768
SF 598.4000245 M
WDW EM
SSB 0
LB 0.30 Hz
GB 0
PC 1.00
9.988
7.984
7.972
7.254
7.252
7.250
7.241
7.239
6.728
6.721
6.674
6.663
6.651
2.846
2.833
2.820
1.761
1.748
1.735
1.728
1.722
1.710
1.582
1.474
1.461
1.449
1.436
1.424
1.412
0.974
0.962
0.949
CHO
N
3f
10
9
8
7
6
5
4
3
2
1
ppm
1.001
1.026
2.029
1.000
1.070
2.092
2.073
2.071
3.091

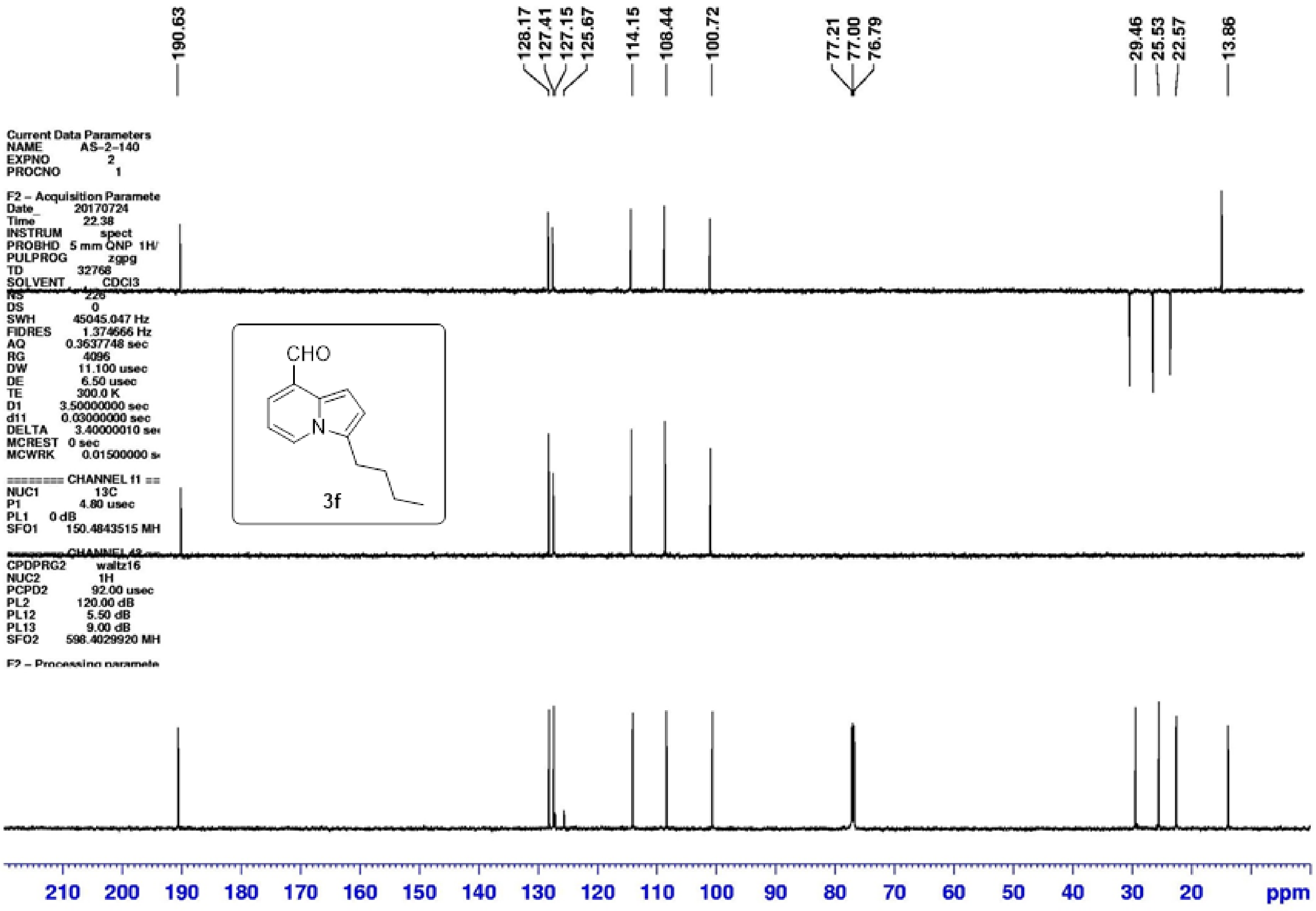
190.63
128.17
127.41
127.15
125.67
114.15
108.44
100.72
77.21
77.00
76.79
29.46
25.53
22.57
13.86
Current Data Parameters
NAME AS-2-140
EXPNO 2
PROCNO 1
F2 - Acquisition Paramete
Date_ 20170724
Time 22.38
INSTRUM spect
PROBHD 5 mm QNP 1H/
PULPROG zgpg
TD 32768
SOLVENT CDCl3
DS 0
SWH 45045.047 Hz
FIDRES 1.374666 Hz
AQ 0.3637748 sec
RG 4096
DW 11.100 usec
DE 6.50 usec
TE 300.0 K
D1 3.50000000 sec
d11 0.03000000 sec
DELTA 3.40000010 se
MCREST 0 sec
MCWRK 0.01500000 s
======== CHANNEL f1 ==
NUC1 13C
P1 4.80 usec
PL1 0 dB
SFO1 150.4843515 MH
CPDPRG2 waltz16
NUC2 1H
PCPD2 92.00 usec
PL2 120.00 dB
PL12 5.50 dB
PL13 9.00 dB
SFO2 598.4029920 MH
CHO
N
3f
210 200 190 180 170 160 150 140 130 120 110 100 90 80 70 60 50 40 30 20 ppm

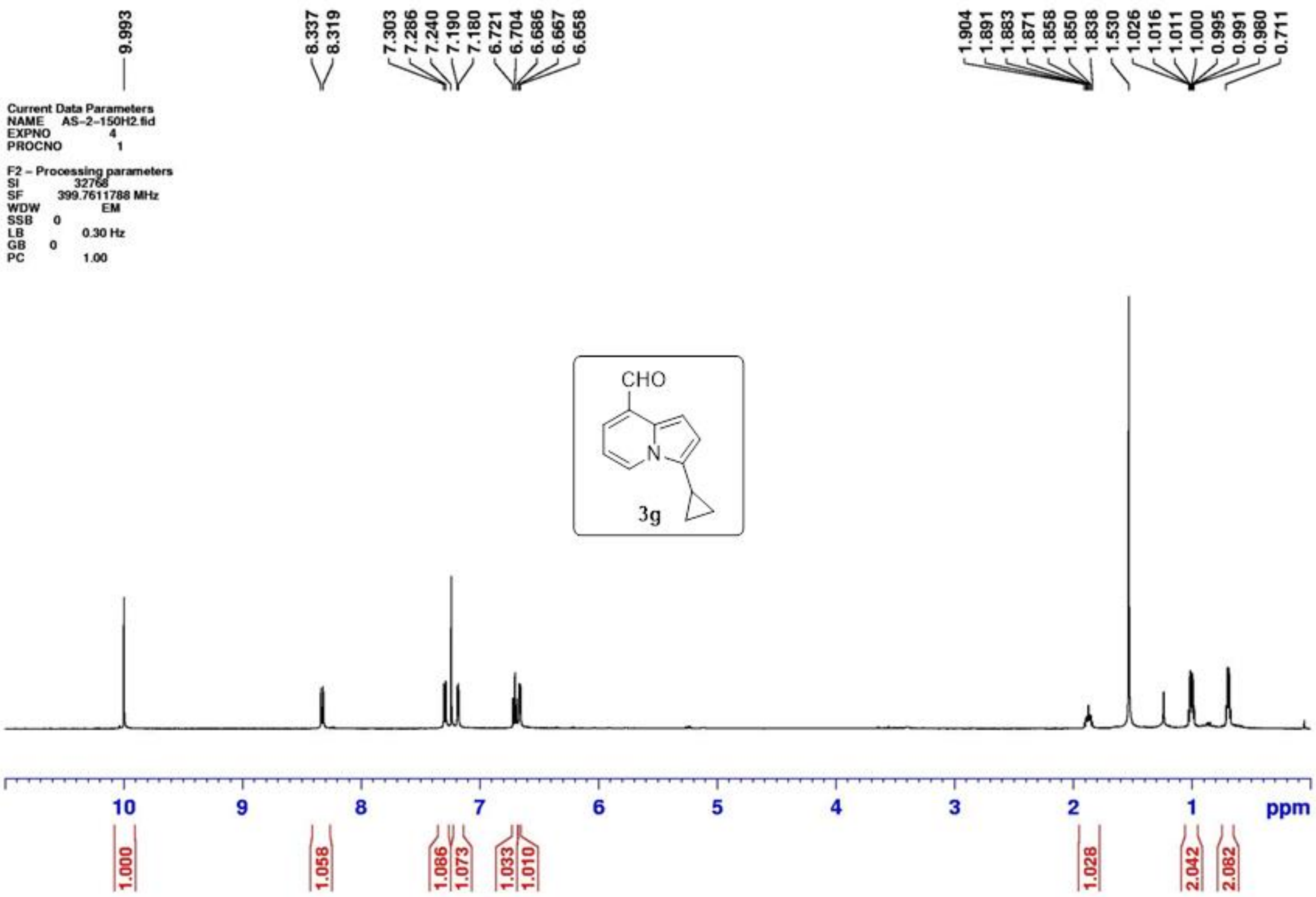
Current Data Parameters
NAME AS-2-150H2.fid
EXPNO 4
PROCNO 1
F2 - Processing parameters
SI 32768
SF 399.7611788 MHz
WDW EM
SSB 0
LB 0.30 Hz
GB 0
PC 1.00
9.993
8.337
8.319
7.303
7.286
7.240
7.190
7.180
6.721
6.704
6.686
6.667
6.658
1.904
1.891
1.883
1.871
1.858
1.850
1.838
1.530
1.026
1.016
1.011
1.000
0.995
0.991
0.980
0.711
CHO
N
3g
10
9
8
7
6
5
4
3
2
1
ppm
1.000
1.058
1.066
1.073
1.033
1.010
1.028
2.042
2.082

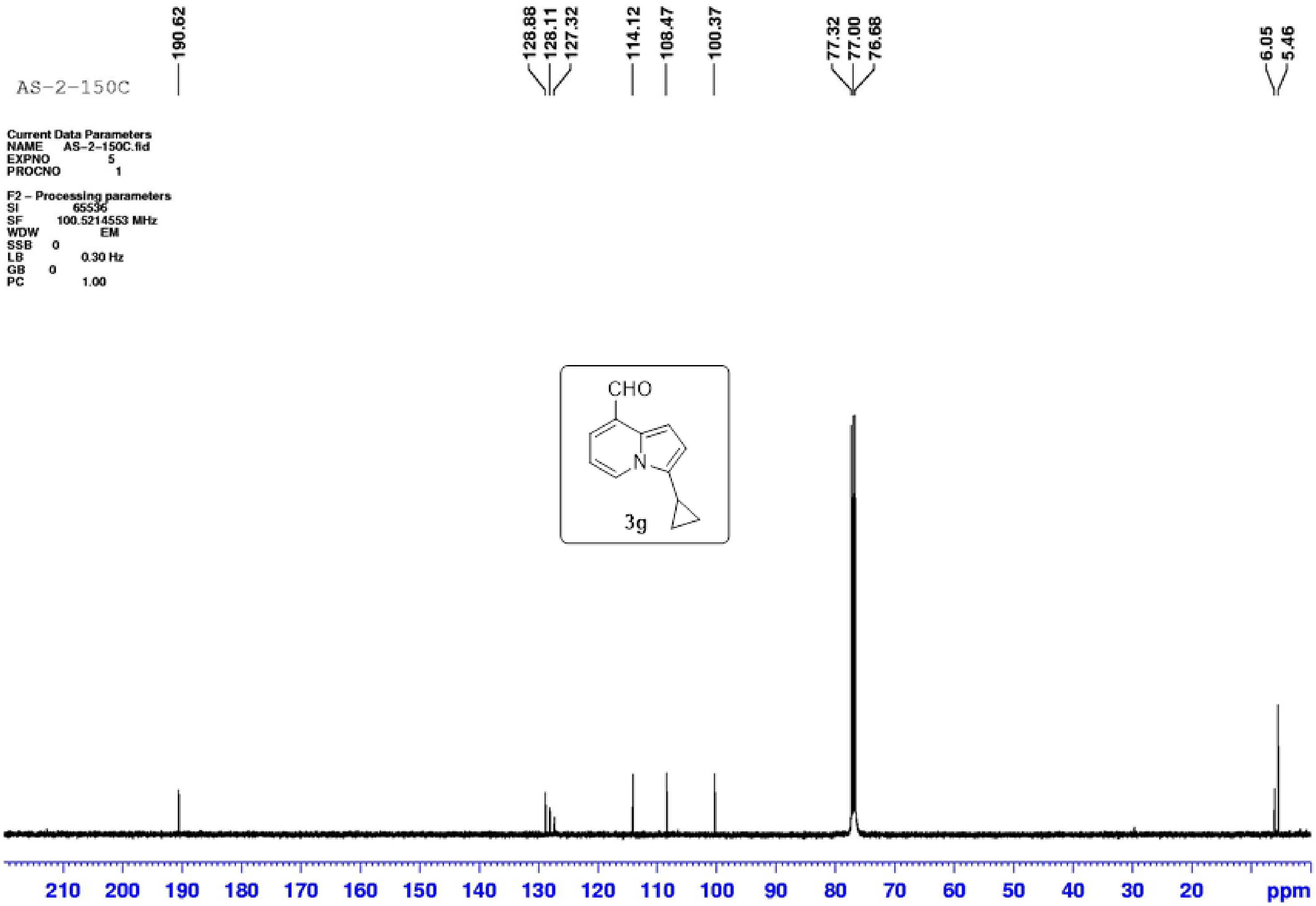

190.62
128.88
128.11
127.32
114.12
108.47
100.37
77.32
77.00
76.68
6.05
5.46
AS-2-150C
Current Data Parameters
NAME AS-2-150C.fid
EXPNO 5
PROCNO 1
F2 - Processing parameters
SI 65536
SF 100.5214553 MHz
WDW EM
SSB 0
LB 0.30 Hz
GB 0
PC 1.00
CHO
N
3g
210 200 190 180 170 160 150 140 130 120 110 100 90 80 70 60 50 40 30 20 ppm

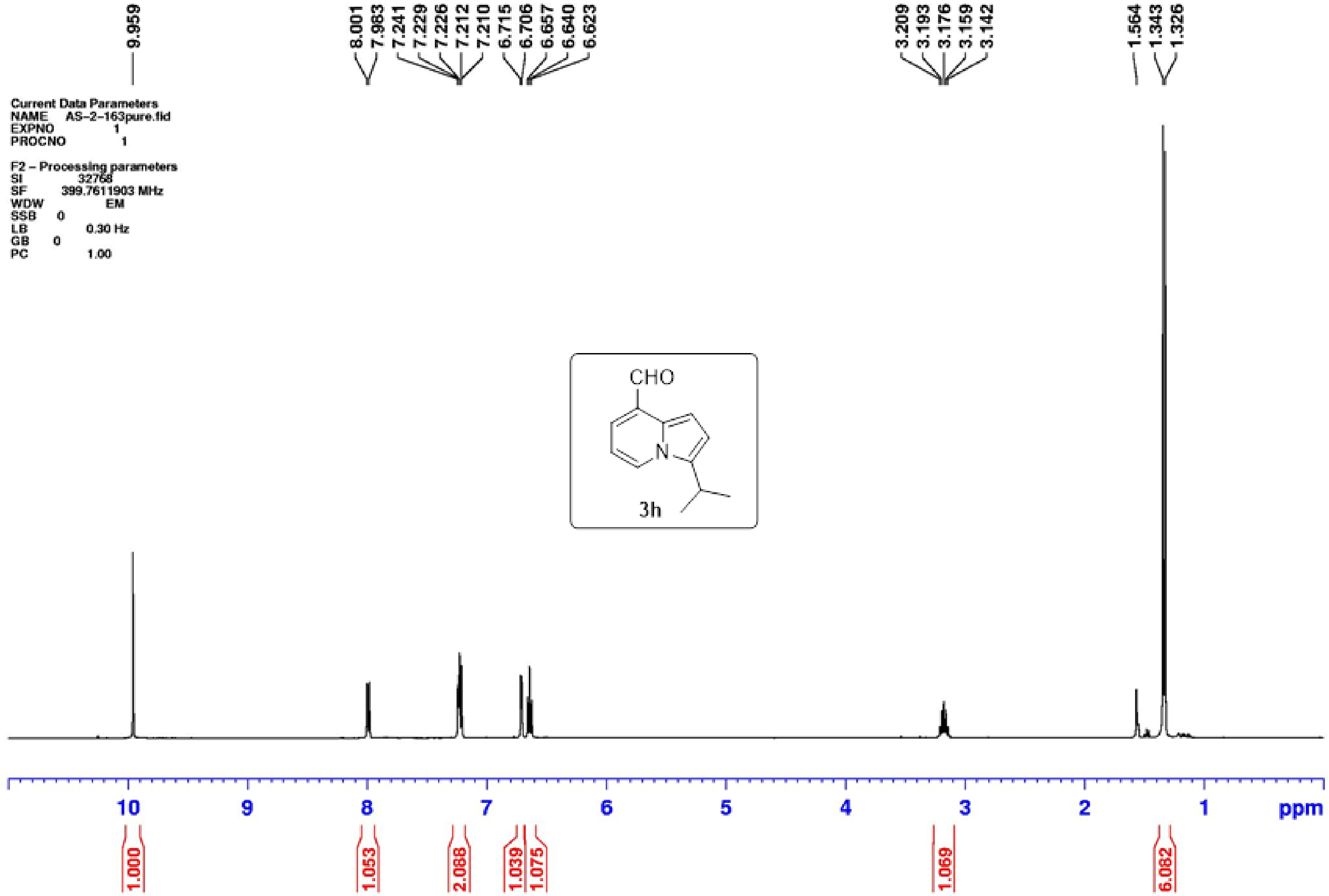
9.959
8.001
7.983
7.241
7.229
7.226
7.212
7.210
6.715
6.706
6.657
6.640
6.623
3.209
3.193
3.176
3.159
3.142
1.564
1.343
1.326
Current Data Parameters
NAME AS-2-163pure.fid
EXPNO 1
PROCNO 1
F2 - Processing parameters
SI 32768
SF 399.7611903 MHz
WDW EM
SSB 0
LB 0.30 Hz
GB 0
PC 1.00
CHO
N
3h
10
9
8
7
6
5
4
3
2
1
ppm
1.000
1.053
2.088
1.039
1.075
1.069
6.082

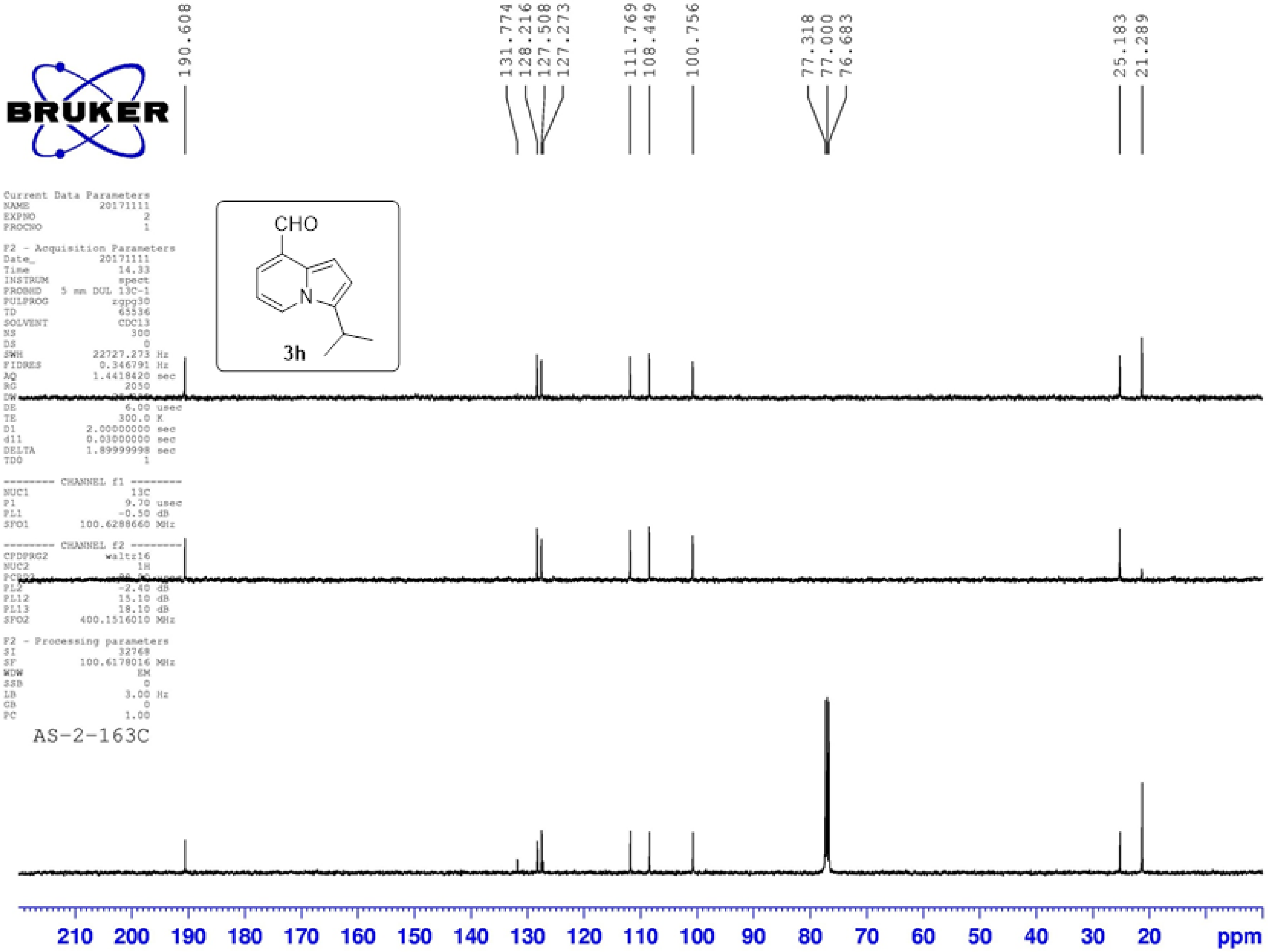
190.608
131.774
128.216
127.508
127.273
111.769
108.449
100.756
77.318
77.000
76.683
25.183
21.289
Current Data Parameters
NAME 20171111
EXPNO 2
PROCNO 1
F2 - Acquisition Parameters
Date_ 20171111
Time 14.33
INSTRUM spect
PROBHD 5 mm DUL 13C-1
PULPROG zgpg30
TD 65536
SOLVENT CDCl3
NS 300
DS 0
SWH 22727.273 Hz
FIDRES 0.346791 Hz
AQ 1.4418420 sec
RG 2050
DE 6.00 usec
TE 300.0 K
D1 2.00000000 sec
d11 0.03000000 sec
DELTA 1.89999998 sec
TD0 1
======== CHANNEL f1 ========
NUC1 13C
P1 9.70 usec
PL1 -0.50 dB
SFO1 100.6288660 MHz
======== CHANNEL f2 ========
CPDPRG2 waltz16
NUC2 1H
PL2 -2.40 dB
PL12 15.10 dB
PL13 18.10 dB
SFO2 400.1516010 MHz
F2 - Processing parameters
SI 32768
SF 100.6178016 MHz
WDW EM
SSB 0
LB 3.00 Hz
GB 0
PC 1.00
AS-2-163C
CHO
N
3h
210 200 190 180 170 160 150 140 130 120 110 100 90 80 70 60 50 40 30 20 ppm

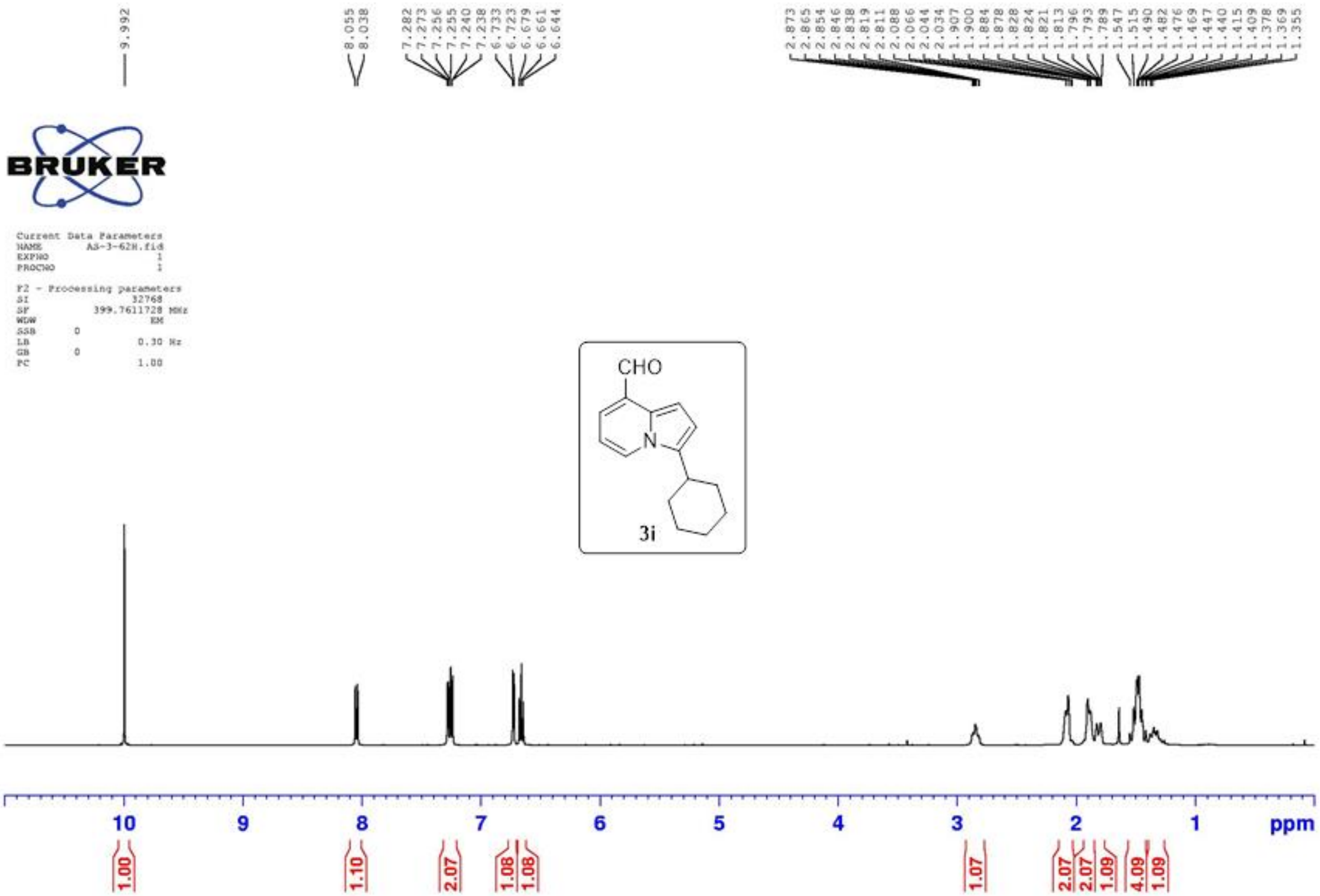
9.992
8.055
8.038
7.282
7.273
7.256
7.255
7.240
7.238
6.733
6.723
6.679
6.661
6.644
2.873
2.865
2.854
2.846
2.838
2.819
2.811
2.088
2.066
2.044
2.034
1.907
1.900
1.884
1.878
1.828
1.824
1.821
1.813
1.796
1.793
1.789
1.547
1.515
1.490
1.482
1.476
1.469
1.447
1.440
1.415
1.409
1.378
1.369
1.355
BRUKER
Current Data Parameters
NAME AS-3-62H.fid
EXPNO 1
PROCNO 1
F2 - Processing parameters
SI 32768
SF 399.7611728 MHz
WDW EM
SSB 0
LB 0.30 Hz
GB 0
PC 1.00
CHO
N
3i
10
9
8
7
6
5
4
3
2
1
ppm
1.00
1.10
2.07
1.08
1.08
1.07
2.07
2.07
1.09
4.09
1.09

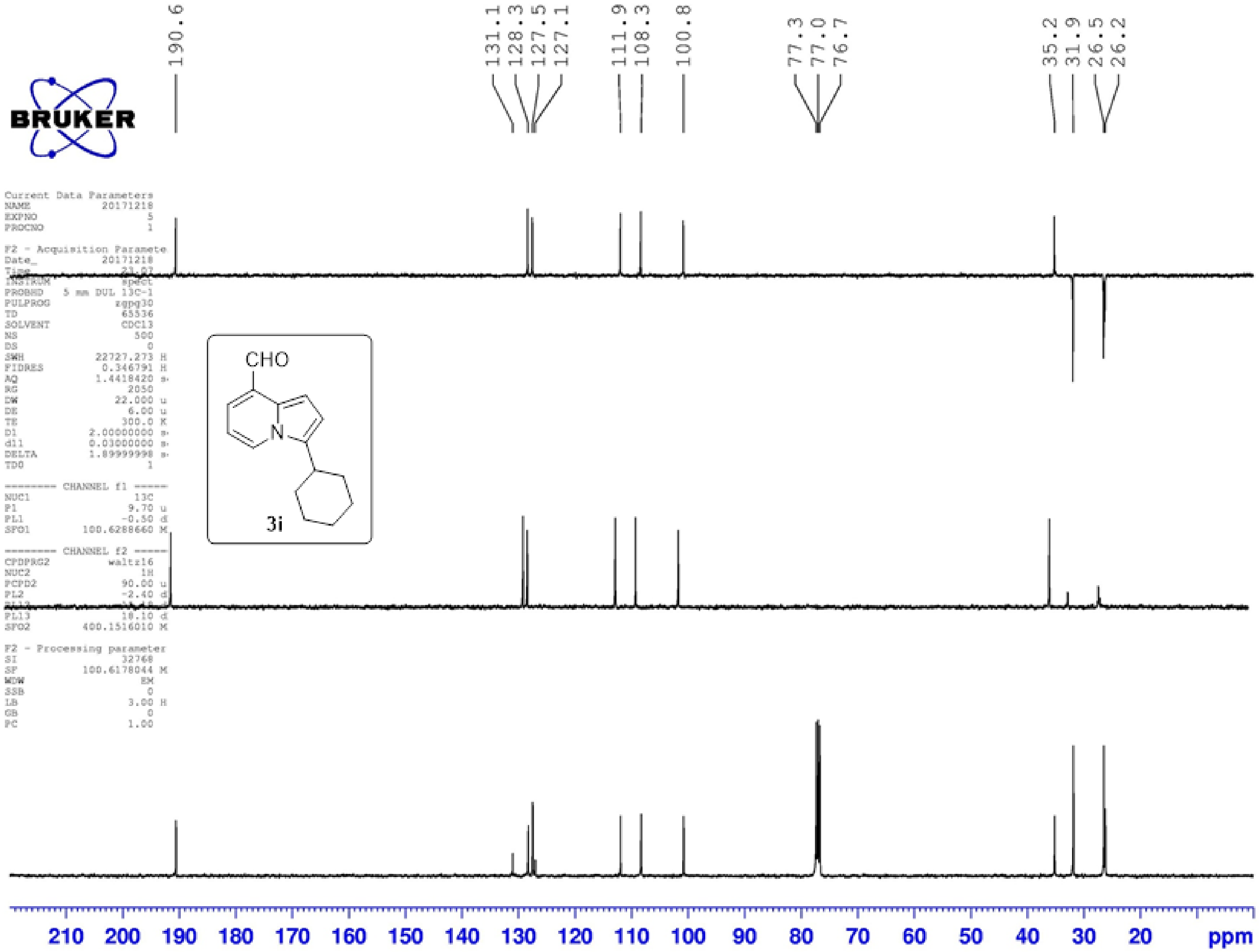
190.6
131.1
128.3
127.5
127.1
111.9
108.3
100.8
77.3
77.0
76.7
35.2
31.9
26.5
26.2
BRUKER
Current Data Parameters
NAME 20171218
EXPNO 5
PROCNO 1
F2 - Acquisition Paramete
Date_ 20171218
PROBHD 5 mm DUL 13C-1
PULPROG zgpg30
TD 65536
SOLVENT CDCl3
NS 500
DS 0
SWH 22727.273 H
FIDRES 0.346791 H
AQ 1.4418420 s
RG 2050
DW 22.000 u
DE 6.00 u
TE 300.0 K
D1 2.00000000 s
d11 0.03000000 s
DELTA 1.89999998 s
TD0 1
======== CHANNEL f1 =====
NUC1 13C
P1 9.70 u
PL1 -0.50 d
SFO1 100.6288660 M
======== CHANNEL f2 =====
CPDPRG2 waltz16
NUC2 1H
PCPD2 90.00 u
PL2 -2.40 d
PL13 18.10 d
SFO2 400.1516010 M
F2 - Processing parameter
SI 32768
SF 100.6178044 M
WDW EM
SSB 0
LB 3.00 H
GB 0
PC 1.00
CHO
N
3i
210 200 190 180 170 160 150 140 130 120 110 100 90 80 70 60 50 40 30 20 ppm

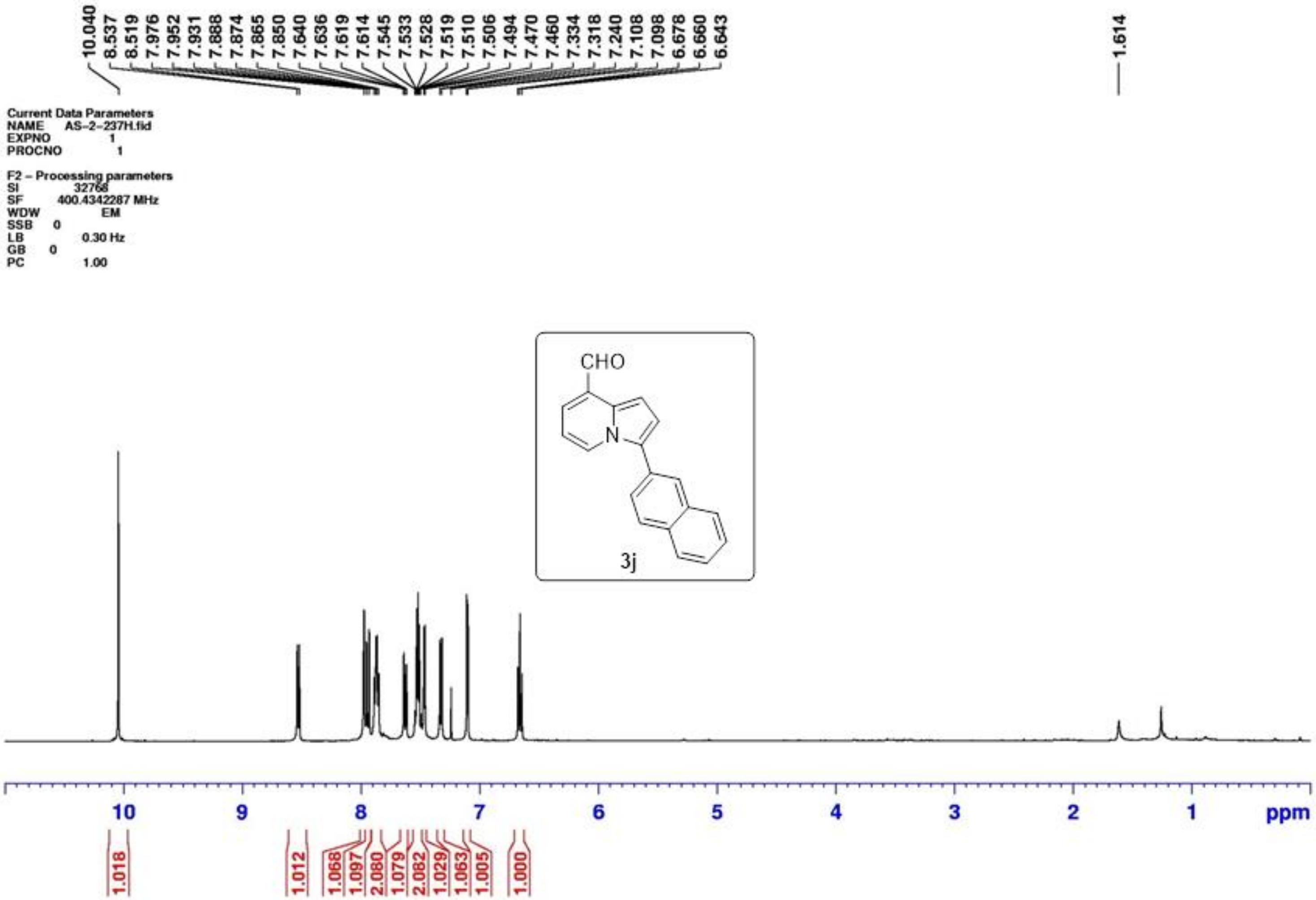
10.040
8.537
8.519
7.976
7.952
7.931
7.888
7.874
7.865
7.850
7.640
7.636
7.619
7.614
7.545
7.533
7.528
7.519
7.510
7.506
7.494
7.470
7.460
7.334
7.318
7.240
7.108
7.098
6.678
6.660
6.643
1.614
Current Data Parameters
NAME AS-2-237H.fid
EXPNO 1
PROCNO 1
F2 - Processing parameters
SI 32768
SF 400.4342287 MHz
WDW EM
SSB 0
LB 0.30 Hz
GB 0
PC 1.00
CHO
N
3j
10
9
8
7
6
5
4
3
2
1
ppm
1.018
1.012
1.068
1.097
2.080
1.079
2.082
1.029
1.063
1.005
1.000

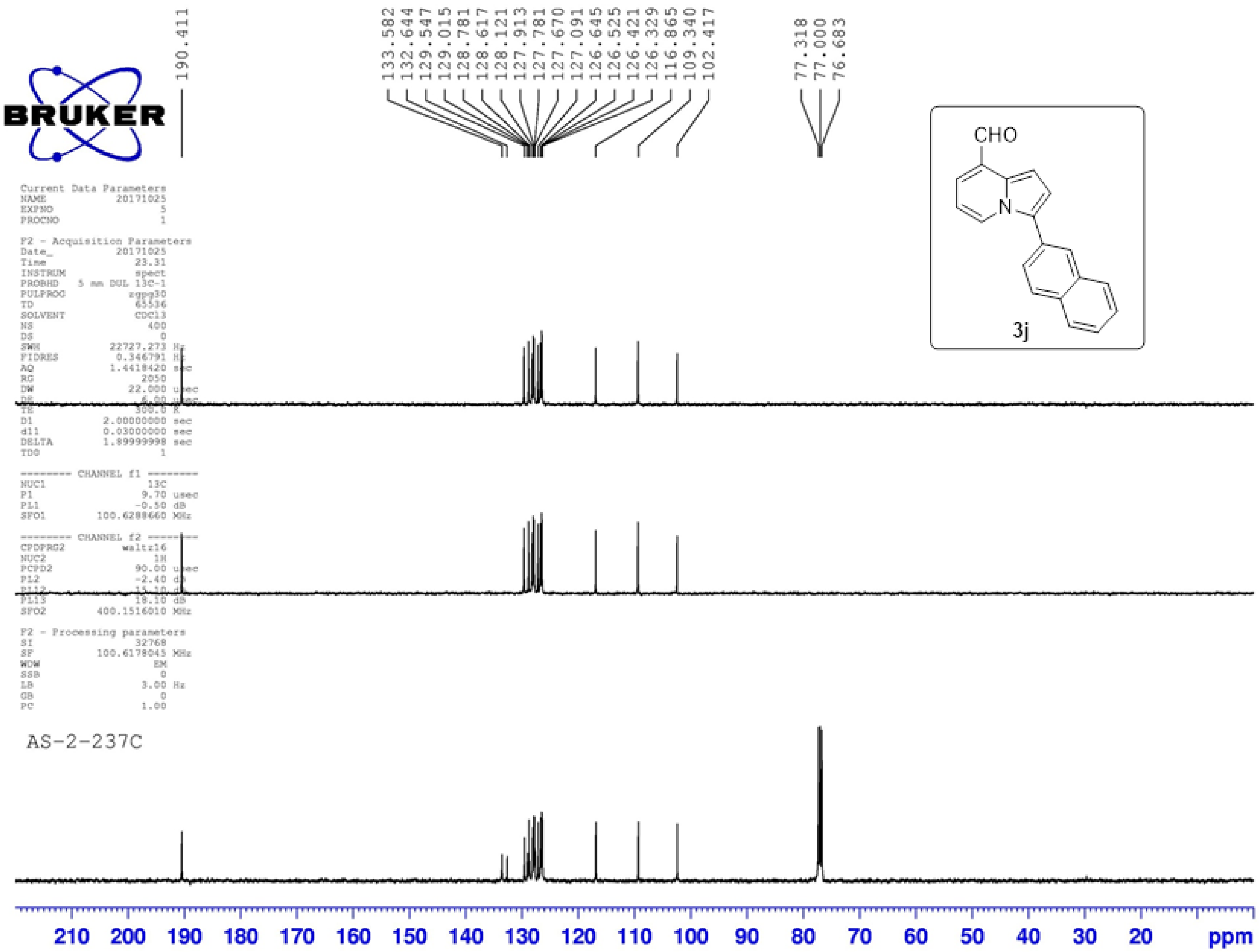
190.411
133.582
132.644
129.547
129.015
128.781
128.617
128.121
127.913
127.781
127.670
127.091
126.645
126.525
126.421
126.329
116.865
109.340
102.417
77.318
77.000
76.683
BRUKER
Current Data Parameters
NAME 20171025
EXPNO 5
PROCNO 1
F2 - Acquisition Parameters
Date_ 20171025
Time 23.31
INSTRUM spect
PROBHD 5 mm DUL 13C-1
PULPROG zgpg30
TD 65536
SOLVENT CDCl3
NS 400
DS 0
SWH 22727.273 Hz
FIDRES 0.346791 Hz
AQ 1.4418420 sec
RG 2050
DW 22.000 usec
DE 6.00 usec
TE 300.0 K
D1 2.00000000 sec
d11 0.03000000 sec
DELTA 1.89999998 sec
TD0 1
======== CHANNEL f1 ========
NUC1 13C
P1 9.70 usec
PL1 -0.50 dB
SFO1 100.6288660 MHz
======== CHANNEL f2 ========
CPDPRG2 waltz16
NUC2 1H
PCPD2 90.00 usec
PL2 -2.40 dB
PL12 15.10 dB
PL13 18.10 dB
SFO2 400.1516010 MHz
F2 - Processing parameters
SI 32768
SF 100.6178045 MHz
WDW EM
SSB 0
LB 3.00 Hz
GB 0
PC 1.00
AS-2-237C
CHO
N
3j
210 200 190 180 170 160 150 140 130 120 110 100 90 80 70 60 50 40 30 20 ppm

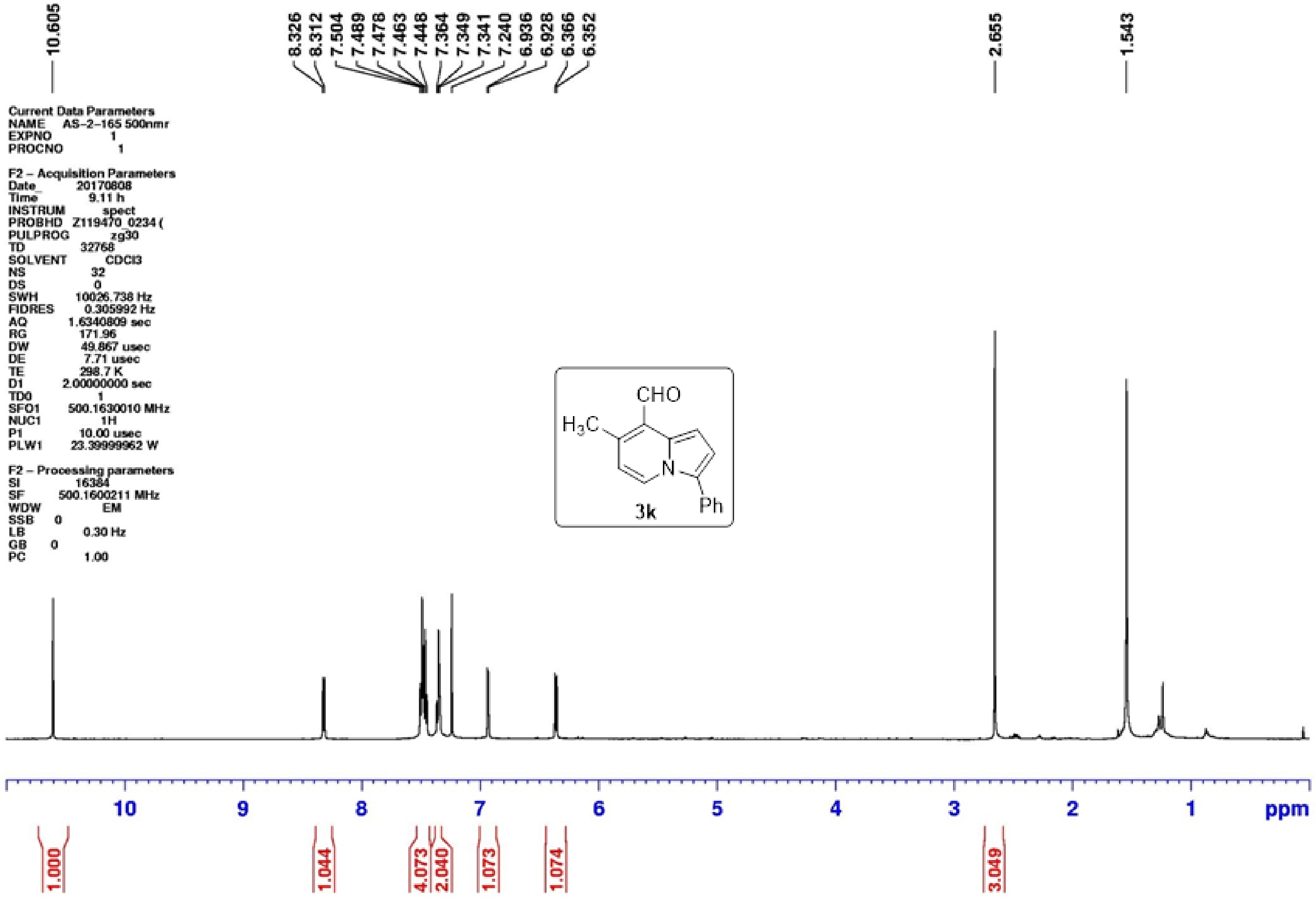
10.605
8.326
8.312
7.504
7.489
7.476
7.463
7.448
7.364
7.349
7.341
7.240
6.936
6.928
6.366
6.352
2.655
1.543
Current Data Parameters
NAME AS-2-165 500nmr
EXPNO 1
PROCNO 1
F2 - Acquisition Parameters
Date_ 20170808
Time 9.11 h
INSTRUM spect
PROBHD Z119470_0234 (
PULPROG zg30
TD 32768
SOLVENT CDCl3
NS 32
DS 0
SWH 10026.738 Hz
FIDRES 0.305992 Hz
AQ 1.6340809 sec
RG 171.96
DW 49.867 usec
DE 7.71 usec
TE 298.7 K
D1 2.00000000 sec
TD0 1
SFO1 500.1630010 MHz
NUC1 1H
P1 10.00 usec
PLW1 23.39999962 W
F2 - Processing parameters
SI 16384
SF 500.1600211 MHz
WDW EM
SSB 0
LB 0.30 Hz
GB 0
PC 1.00
CHO
$H_3C$
N
Ph
3k
10
9
8
7
6
5
4
3
2
1
ppm
1.000
1.044
4.073
2.040
1.073
1.074
3.049

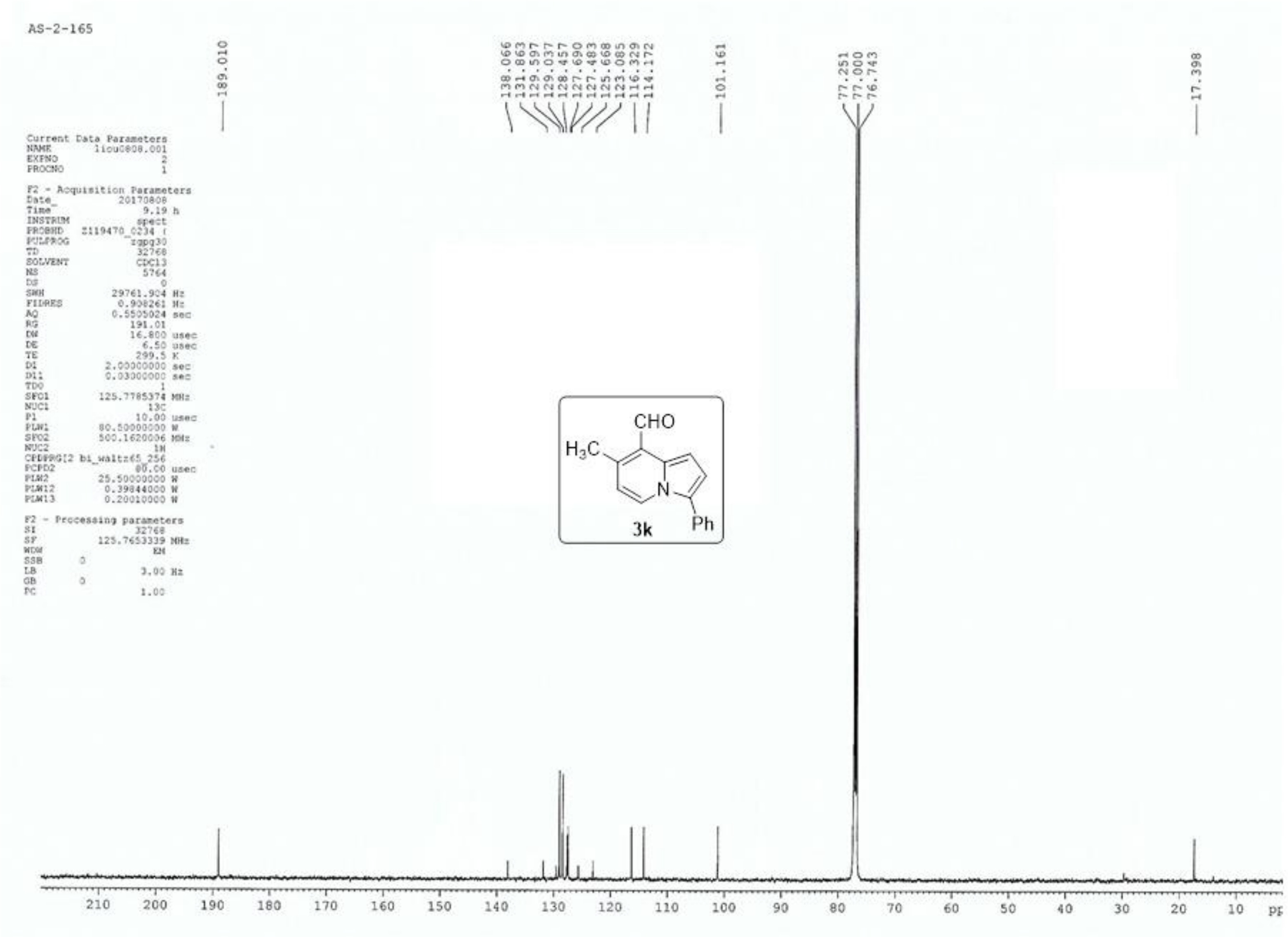
AS-2-165
189.010
138.066
131.863
129.597
129.037
128.457
127.690
127.483
125.668
123.085
116.329
114.172
101.161
77.251
77.000
76.743
17.398
Current Data Parameters
NAME liou0808.001
EXPNO 2
PROCNO 1
F2 - Acquisition Parameters
Date_ 20170808
Time 9.19 h
INSTRUM spect
PROBHD Z119470_0234 (
PULPROG zgpg30
TD 32768
SOLVENT CDCl3
NS 5764
DS 0
SWH 29761.904 Hz
FIDRES 0.908261 Hz
AQ 0.5505024 sec
RG 191.01
DW 16.800 usec
DE 6.50 usec
TE 299.5 K
D1 2.00000000 sec
D11 0.03000000 sec
TD0 1
SFO1 125.7785374 MHz
NUC1 13C
P1 10.00 usec
PLW1 80.50000000 W
SFO2 500.1620006 MHz
NUC2 1H
CPDPRG[2 bi_waltz65_256
PCPD2 80.00 usec
PLW2 25.50000000 W
PLW12 0.39844000 W
PLW13 0.20010000 W
F2 - Processing parameters
SI 32768
SF 125.7653339 MHz
WDW EM
SSB 0
LB 3.00 Hz
GB 0
PC 1.00
CHO
$H_3C$
N
Ph
3k
210 200 190 180 170 160 150 140 130 120 110 100 90 80 70 60 50 40 30 20 10 ppm

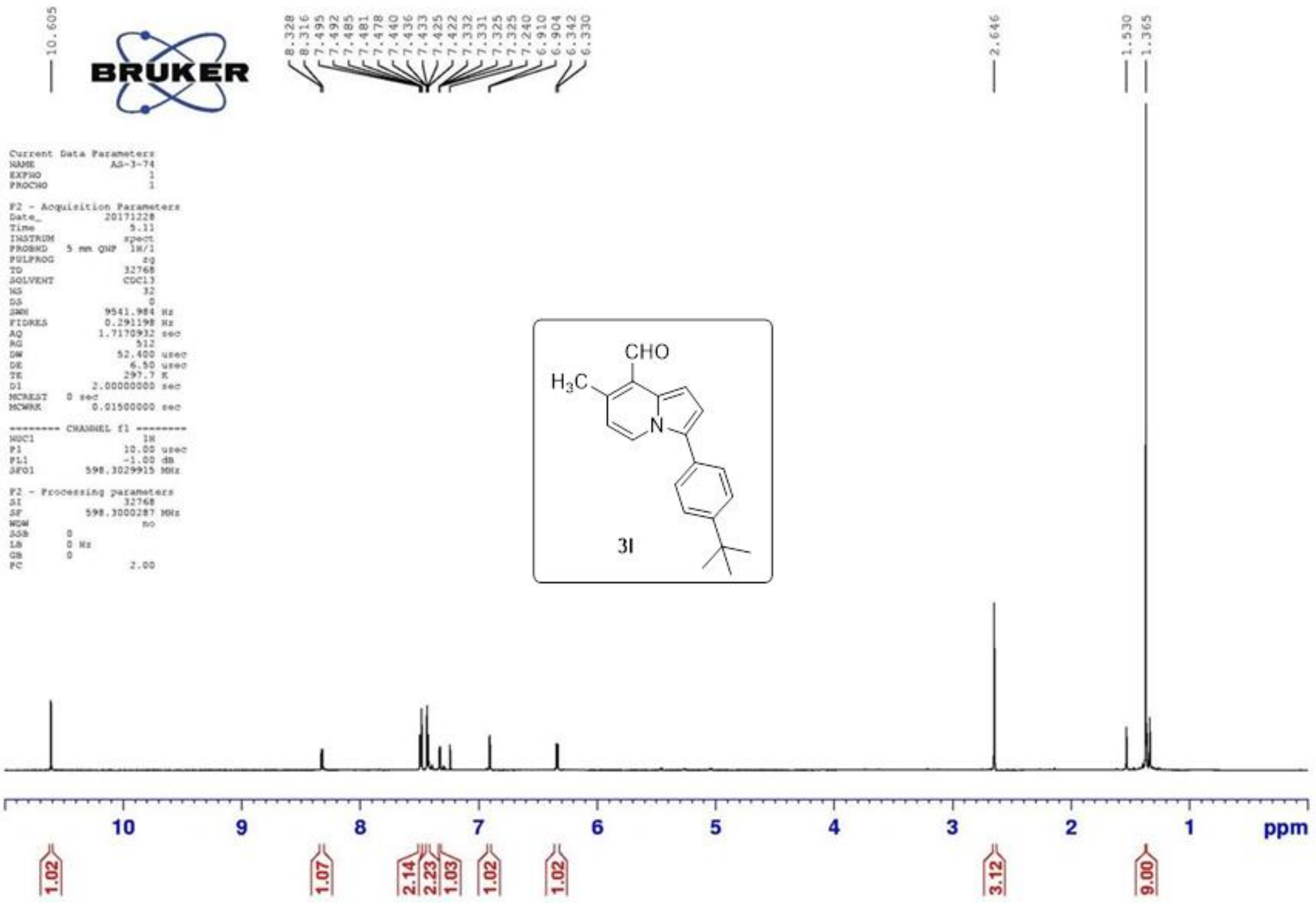
10.605
8.328
8.316
7.495
7.492
7.485
7.481
7.478
7.440
7.436
7.433
7.425
7.422
7.332
7.331
7.325
7.325
7.240
6.910
6.904
6.342
6.330
2.646
1.530
1.365
BRUKER
Current Data Parameters
NAME AS-3-74
EXPNO 1
PROCNO 1
F2 - Acquisition Parameters
Date_ 20171228
Time 5.11
INSTRUM spect
PROBHD 5 mm QNP 1H/1
PULPROG zg
TD 32768
SOLVENT CDCl3
NS 32
DS 0
SWH 9541.984 Hz
FIDRES 0.291198 Hz
AQ 1.7170932 sec
RG 512
DW 52.400 usec
DE 6.50 usec
TE 297.7 K
D1 2.00000000 sec
MCREST 0 sec
MCWRK 0.01500000 sec
======== CHANNEL f1 ========
NUC1 1H
P1 10.00 usec
PL1 -1.00 dB
SFO1 598.3029915 MHz
F2 - Processing parameters
SI 32768
SF 598.3000287 MHz
WDW no
SSB 0
LB 0 Hz
GB 0
PC 2.00
CHO
H3C
N
3l
10
9
8
7
6
5
4
3
2
1
ppm
1.02
1.07
2.14
2.23
1.03
1.02
1.02
3.12
9.00

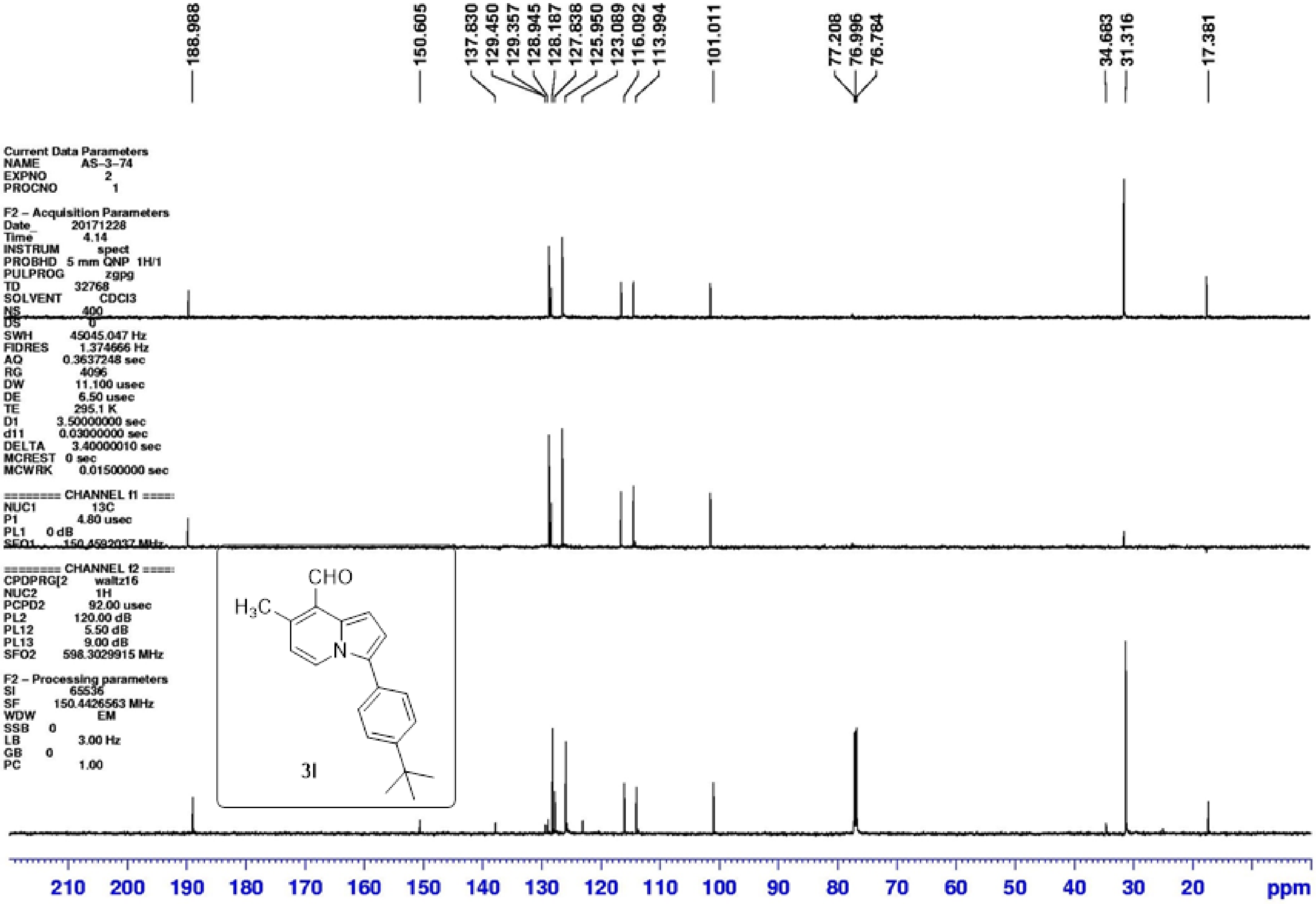

188.988
150.605
137.630
129.450
129.357
128.945
128.187
127.838
125.950
123.089
116.092
113.994
101.011
77.208
76.996
76.784
34.683
31.316
17.381
Current Data Parameters
NAME AS-3-74
EXPNO 2
PROCNO 1
F2 - Acquisition Parameters
Date_ 20171228
Time 4.14
INSTRUM spect
PROBHD 5 mm QNP 1H/1
PULPROG zgpg
TD 32768
SOLVENT CDCl3
NS 400
DS 0
SWH 45045.047 Hz
FIDRES 1.374666 Hz
AQ 0.3637248 sec
RG 4096
DW 11.100 usec
DE 6.50 usec
TE 295.1 K
D1 3.50000000 sec
d11 0.03000000 sec
DELTA 3.40000010 sec
MCREST 0 sec
MCWRK 0.01500000 sec
======== CHANNEL f1 ========
NUC1 13C
P1 4.80 usec
PL1 0 dB
SFO1 150.4592037 MHz
======== CHANNEL f2 ========
CPDPRG[2 waltz16
NUC2 1H
PCPD2 92.00 usec
PL2 120.00 dB
PL12 5.50 dB
PL13 9.00 dB
SFO2 598.3029915 MHz
F2 - Processing parameters
SI 65536
SF 150.4426563 MHz
WDW EM
SSB 0
LB 3.00 Hz
GB 0
PC 1.00
CHO
H3C
N
3l
210 200 190 180 170 160 150 140 130 120 110 100 90 80 70 60 50 40 30 20 ppm

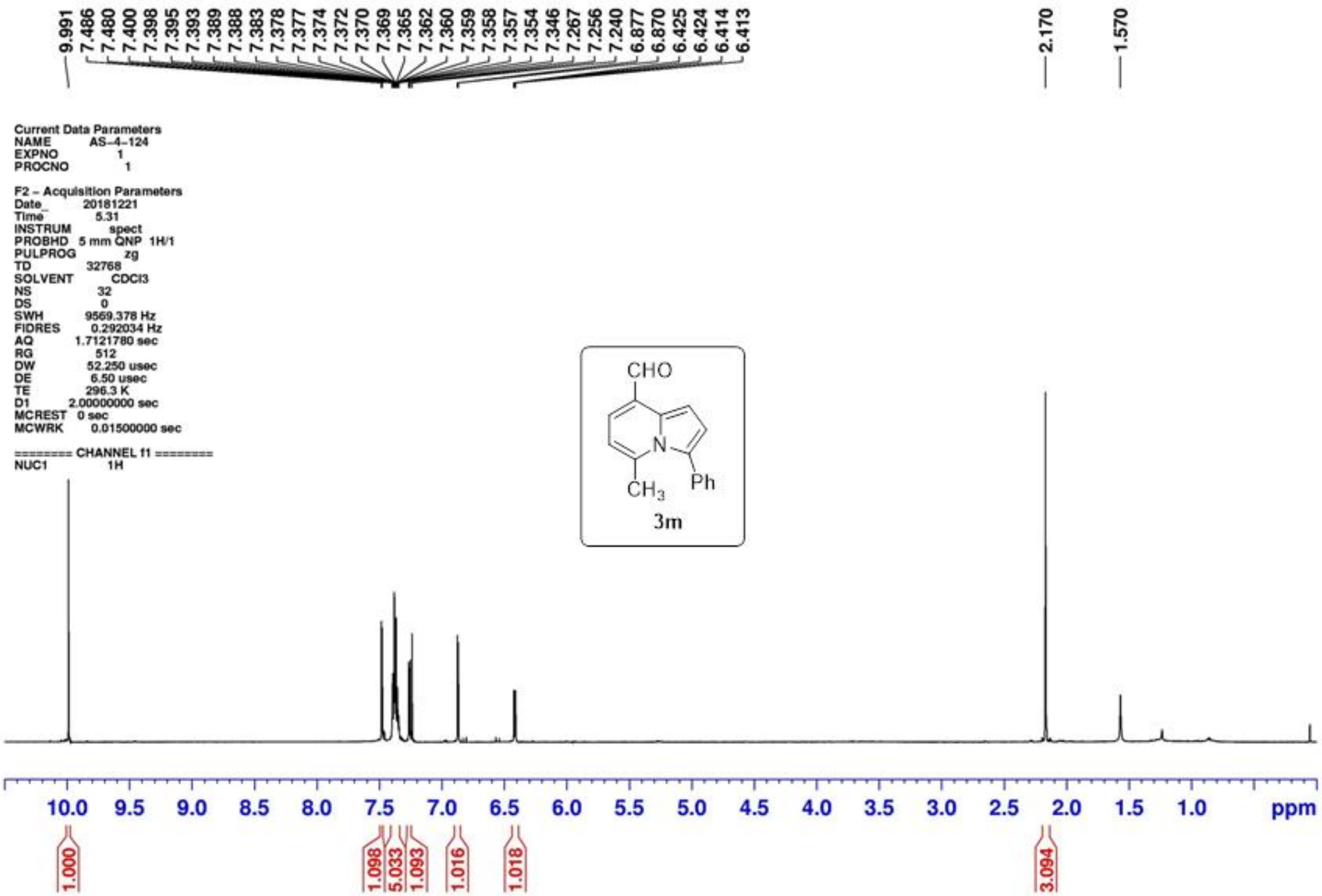
9.991
7.486
7.480
7.400
7.398
7.395
7.393
7.389
7.388
7.383
7.378
7.377
7.374
7.372
7.370
7.369
7.365
7.362
7.360
7.359
7.358
7.357
7.354
7.346
7.267
7.256
7.240
6.877
6.870
6.425
6.424
6.414
6.413
2.170
1.570
Current Data Parameters
NAME AS-4-124
EXPNO 1
PROCNO 1
F2 - Acquisition Parameters
Date_ 20181221
Time 5.31
INSTRUM spect
PROBHD 5 mm QNP 1H/1
PULPROG zg
TD 32768
SOLVENT CDCl3
NS 32
DS 0
SWH 9569.378 Hz
FIDRES 0.292034 Hz
AQ 1.7121780 sec
RG 512
DW 52.250 usec
DE 6.50 usec
TE 296.3 K
D1 2.00000000 sec
MCREST 0 sec
MCWRK 0.01500000 sec
======== CHANNEL f1 ========
NUC1 1H
CHO
N
CH3
Ph
3m
10.0 9.5 9.0 8.5 8.0 7.5 7.0 6.5 6.0 5.5 5.0 4.5 4.0 3.5 3.0 2.5 2.0 1.5 1.0 ppm
1.000
1.098
5.033
1.093
1.016
1.018
3.094

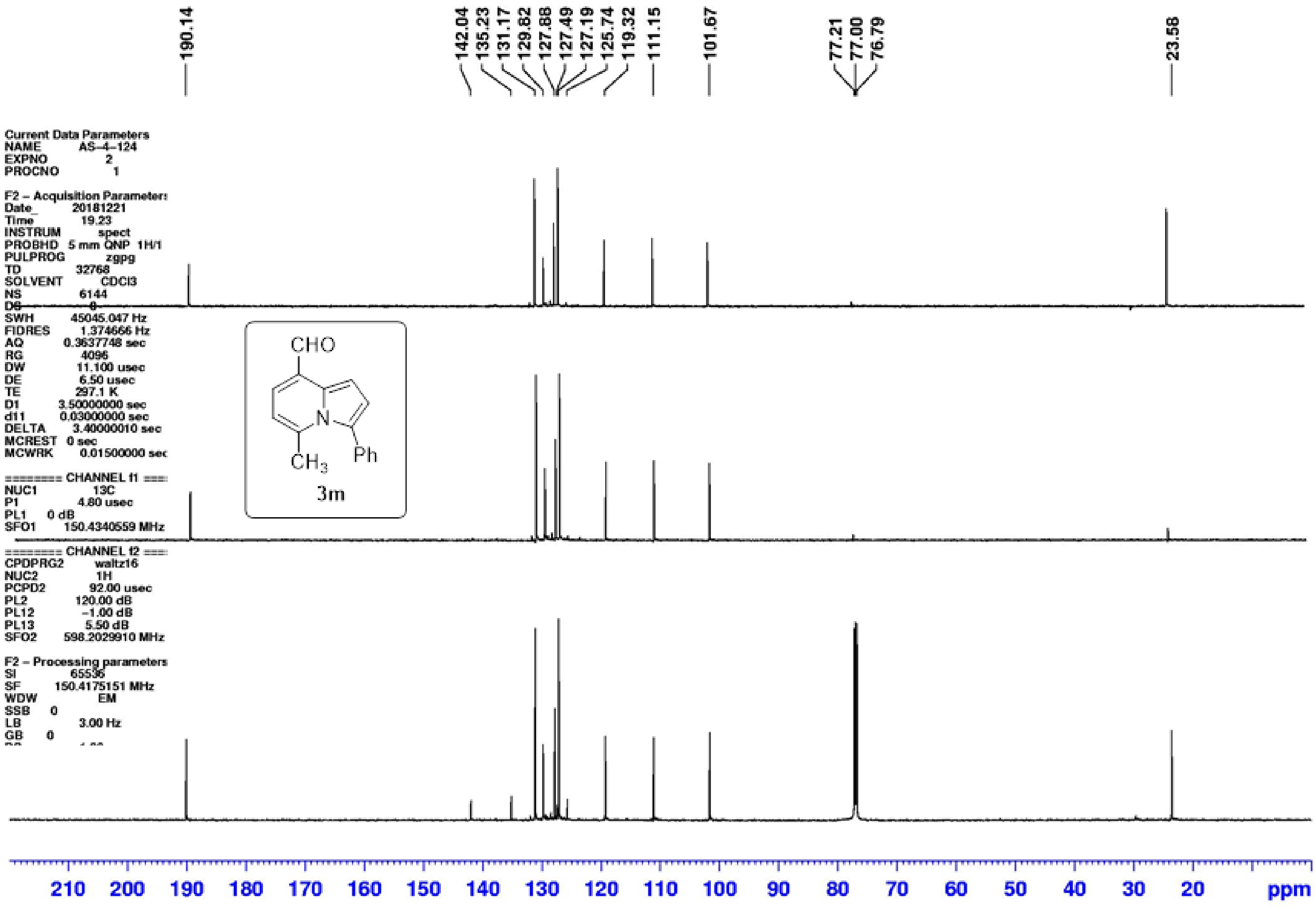
190.14
142.04
135.23
131.17
129.82
127.88
127.49
127.19
125.74
119.32
111.15
101.67
77.21
77.00
76.79
23.58
Current Data Parameters
NAME AS-4-124
EXPNO 2
PROCNO 1
F2 - Acquisition Parameters
Date_ 20181221
Time 19.23
INSTRUM spect
PROBHD 5 mm QNP 1H/1
PULPROG zgpg
TD 32768
SOLVENT CDCl3
NS 6144
SWH 45045.047 Hz
FIDRES 1.374666 Hz
AQ 0.3637748 sec
RG 4096
DW 11.100 usec
DE 6.50 usec
TE 297.1 K
D1 3.50000000 sec
d11 0.03000000 sec
DELTA 3.40000010 sec
MCREST 0 sec
MCWRK 0.01500000 sec
======== CHANNEL f1 ========
NUC1 13C
P1 4.80 usec
PL1 0 dB
SFO1 150.4340559 MHz
======== CHANNEL f2 ========
CPDPRG2 waltz16
NUC2 1H
PCPD2 92.00 usec
PL2 120.00 dB
PL12 -1.00 dB
PL13 5.50 dB
SFO2 598.2029910 MHz
F2 - Processing parameters
SI 65536
SF 150.4175151 MHz
WDW EM
SSB 0
LB 3.00 Hz
GB 0
CHO
N
CH3
Ph
3m
210 200 190 180 170 160 150 140 130 120 110 100 90 80 70 60 50 40 30 20 ppm

<sup></sup>

$^{1}$H NOE of compound **3m**

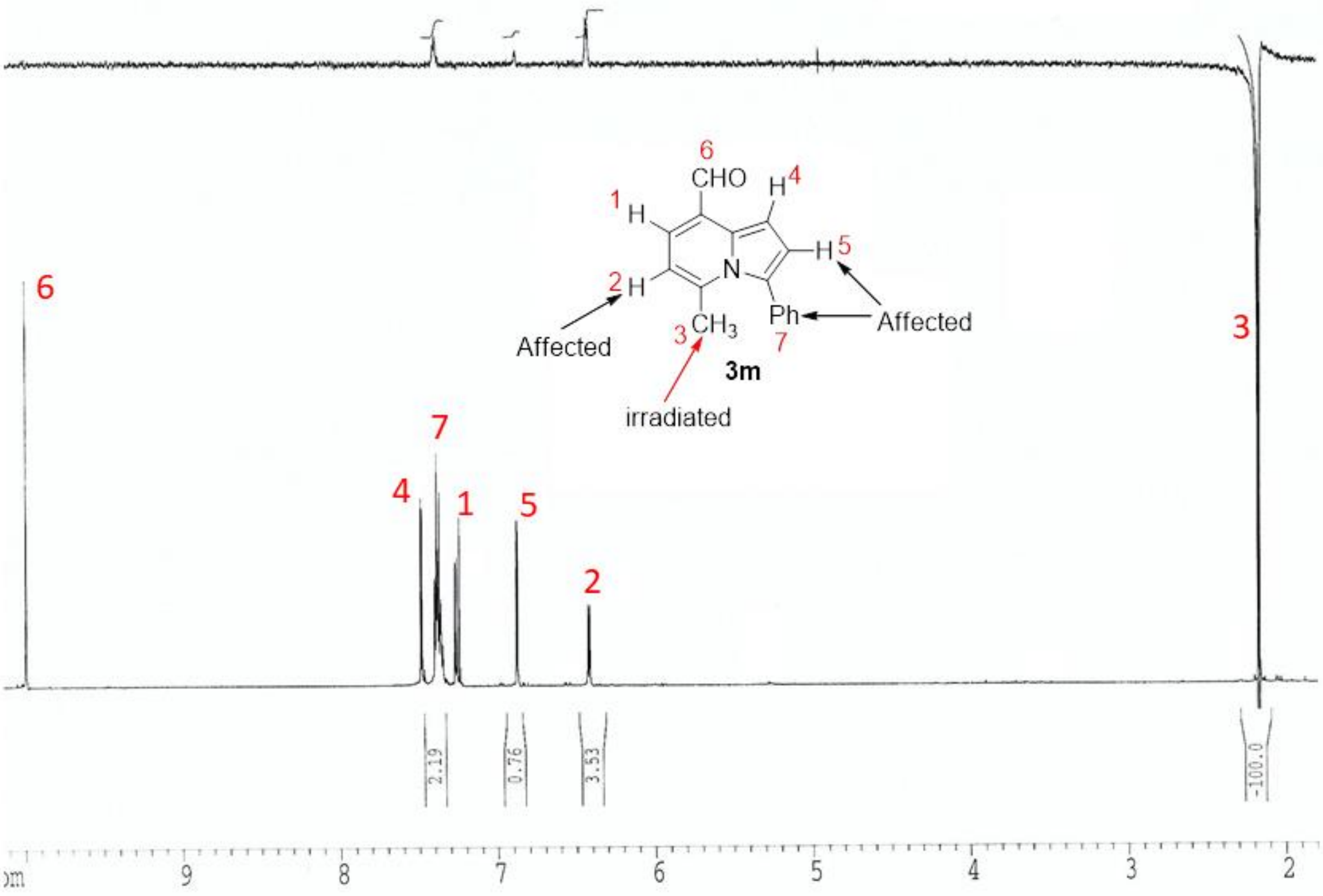

$^1H$ NOE of compound **3m**

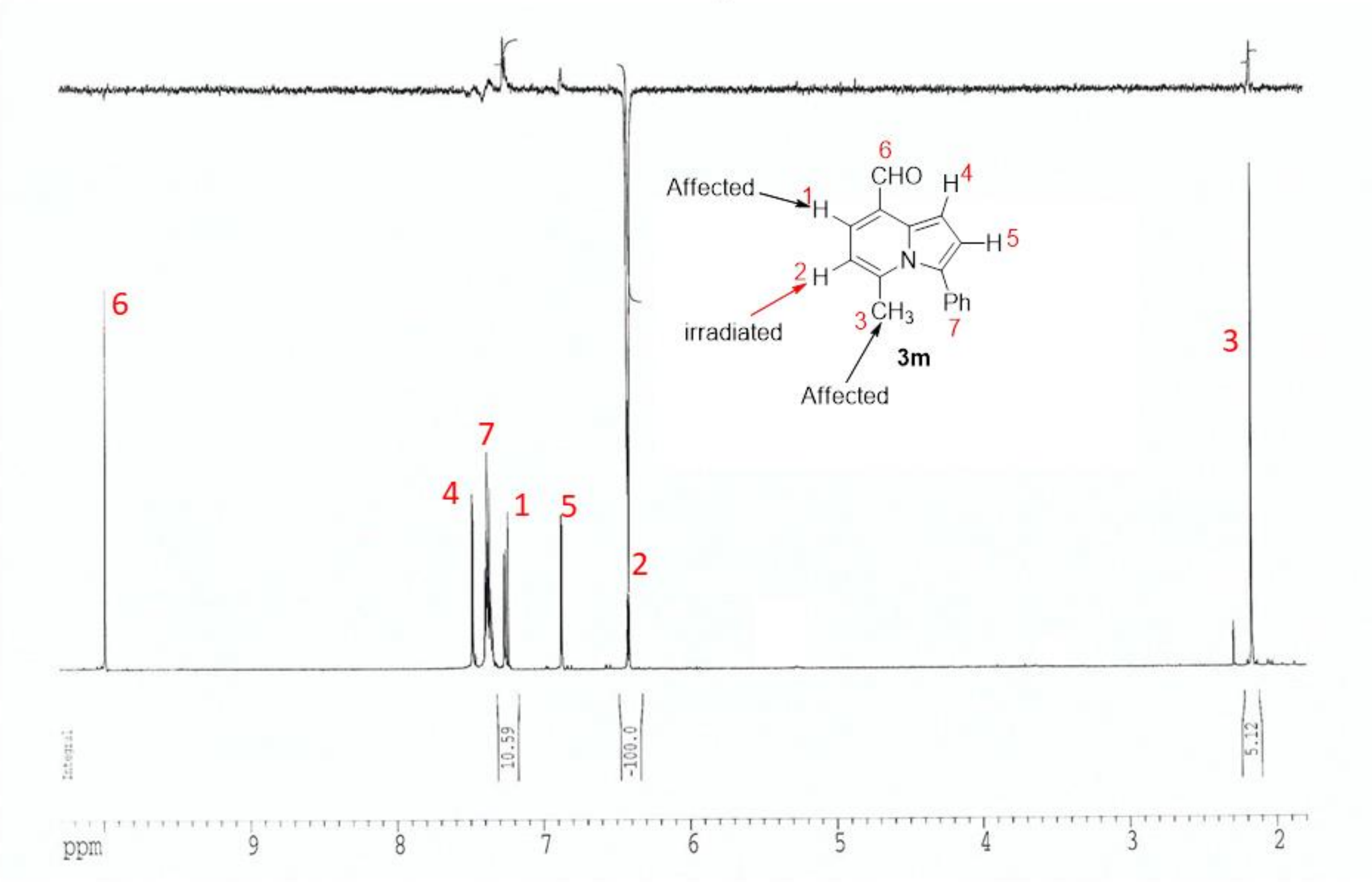

¹H NOE of compound **3m**

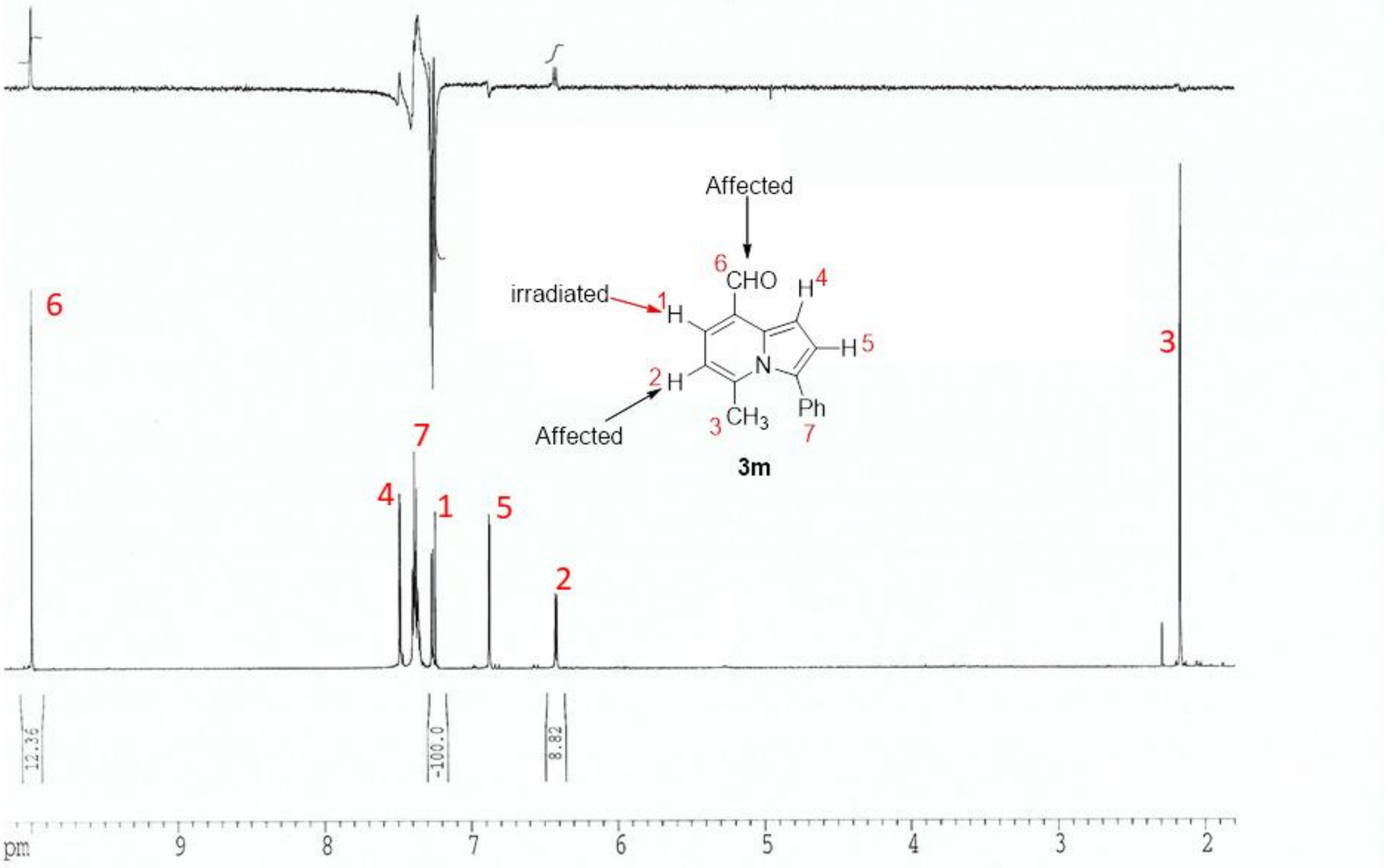

$^{1}$H NOE of compound **3m**

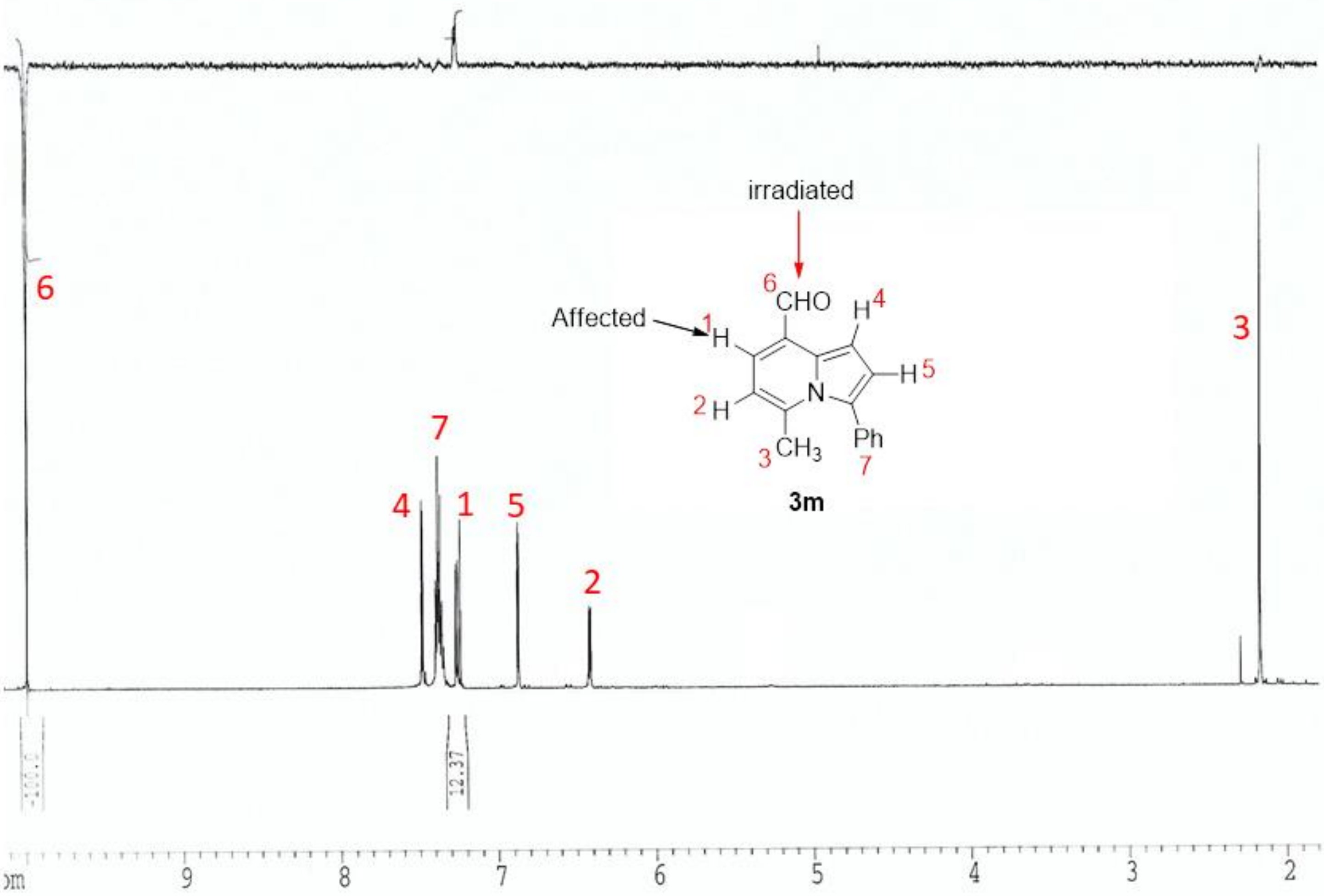

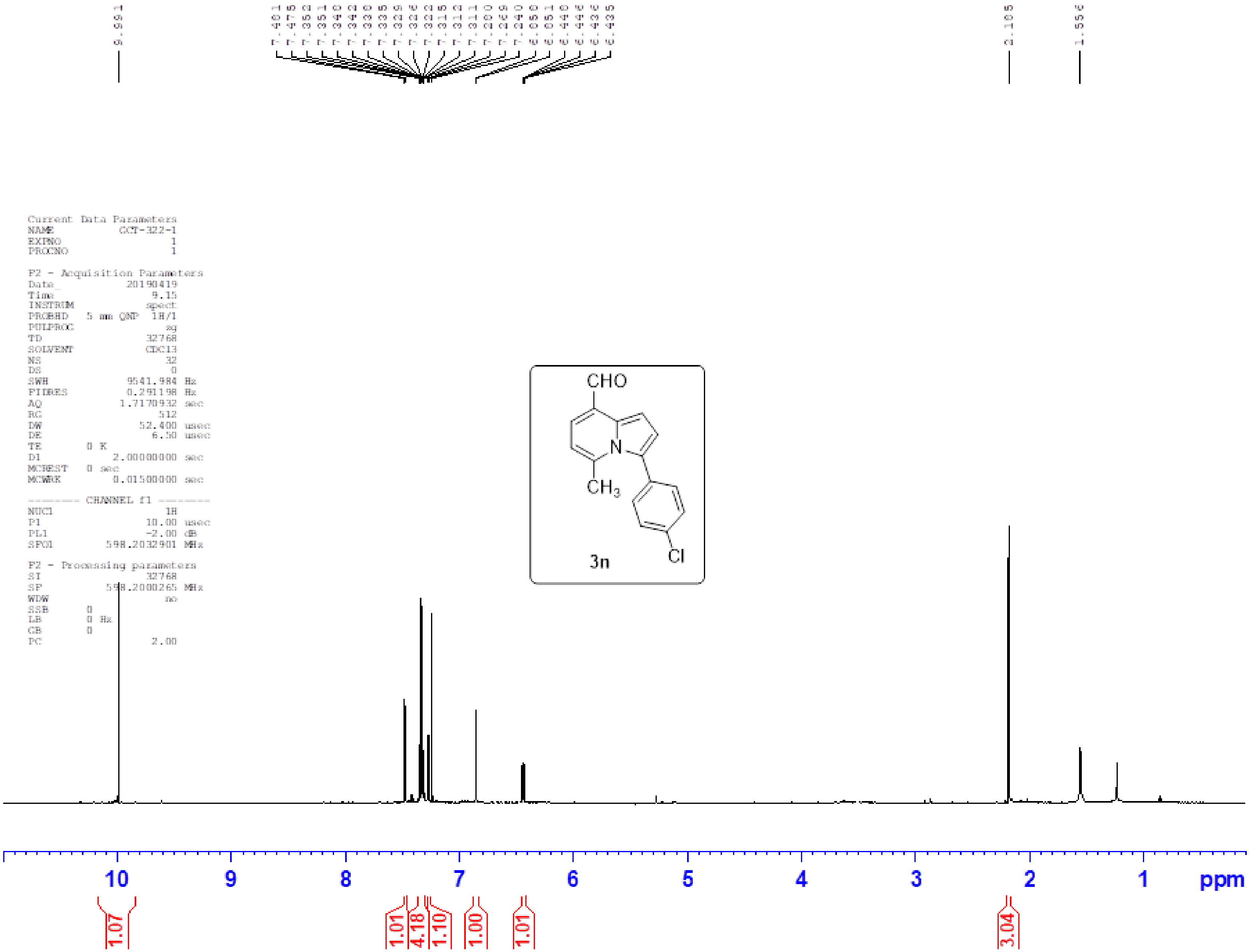
9.991
2.185
1.556
Current Data Parameters
NAME GCT-322-1
EXPNO 1
PROCNO 1
F2 - Acquisition Parameters
Date_ 20190419
Time 9.15
INSTRUM spect
PROBHD 5 mm QNP 1H/1
PULPROG zg
TD 32768
SOLVENT CDCl3
NS 32
DS 0
SWH 9541.984 Hz
FIDRES 0.291198 Hz
AQ 1.7170932 sec
RG 512
DW 52.400 usec
DE 6.50 usec
TE 0 K
D1 2.00000000 sec
MCREST 0 sec
MCWRK 0.01500000 sec
======== CHANNEL f1 ========
NUC1 1H
P1 10.00 usec
PL1 -2.00 dB
SFO1 598.2032901 MHz
F2 - Processing parameters
SI 32768
SF 598.2000265 MHz
WDW no
SSB 0
LB 0 Hz
GB 0
PC 2.00
CHO
N
CH3
Cl
3n
10
9
8
7
6
5
4
3
2
1
ppm
1.07
1.01
4.18
1.10
1.00
1.01
3.04

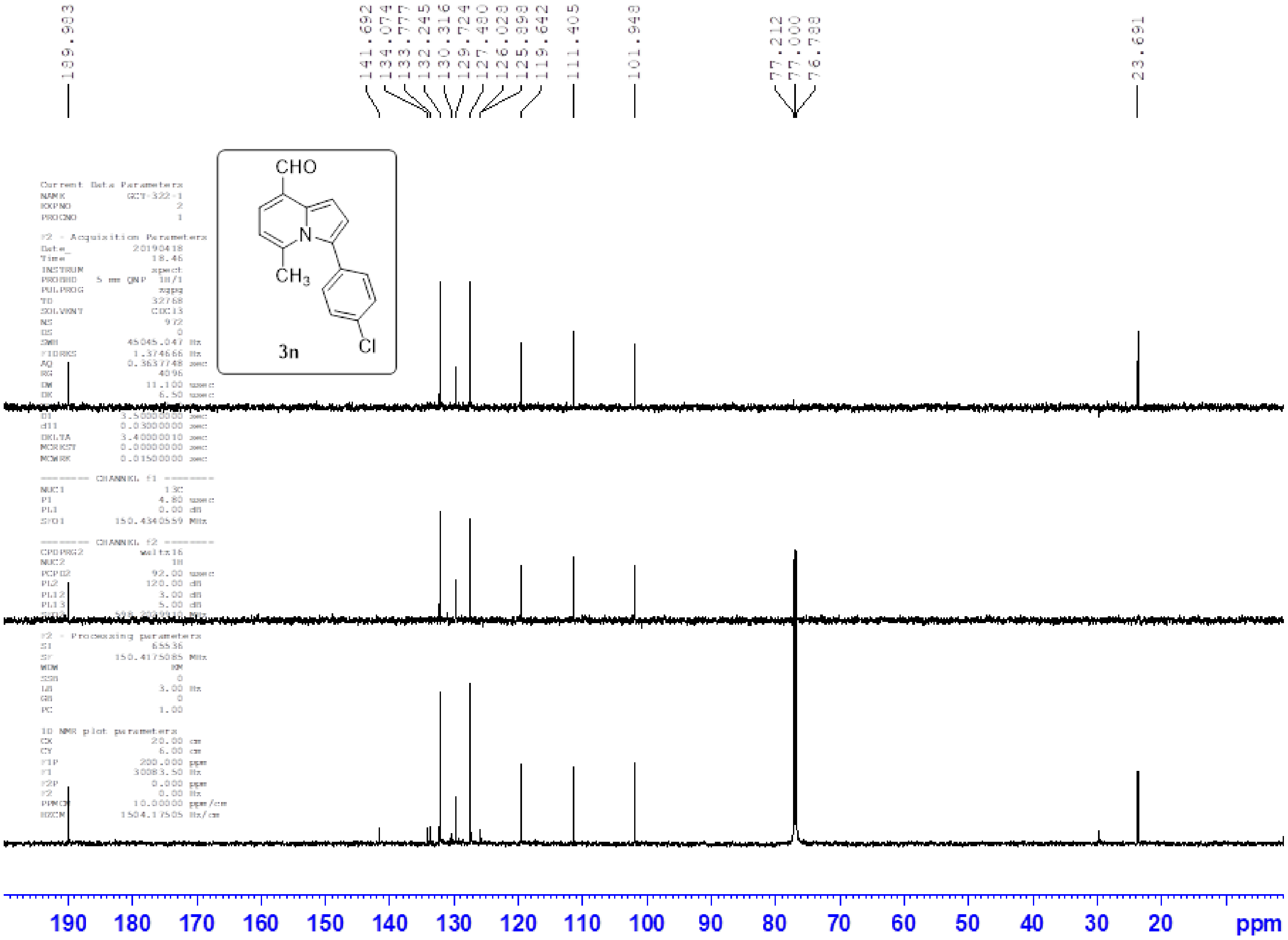
189.983
141.692
134.074
133.777
132.245
130.316
129.724
127.480
126.028
125.898
119.642
111.405
101.949
77.212
77.000
76.788
23.691
CHO
N
CH3
Cl
3n
190 180 170 160 150 140 130 120 110 100 90 80 70 60 50 40 30 20 ppm

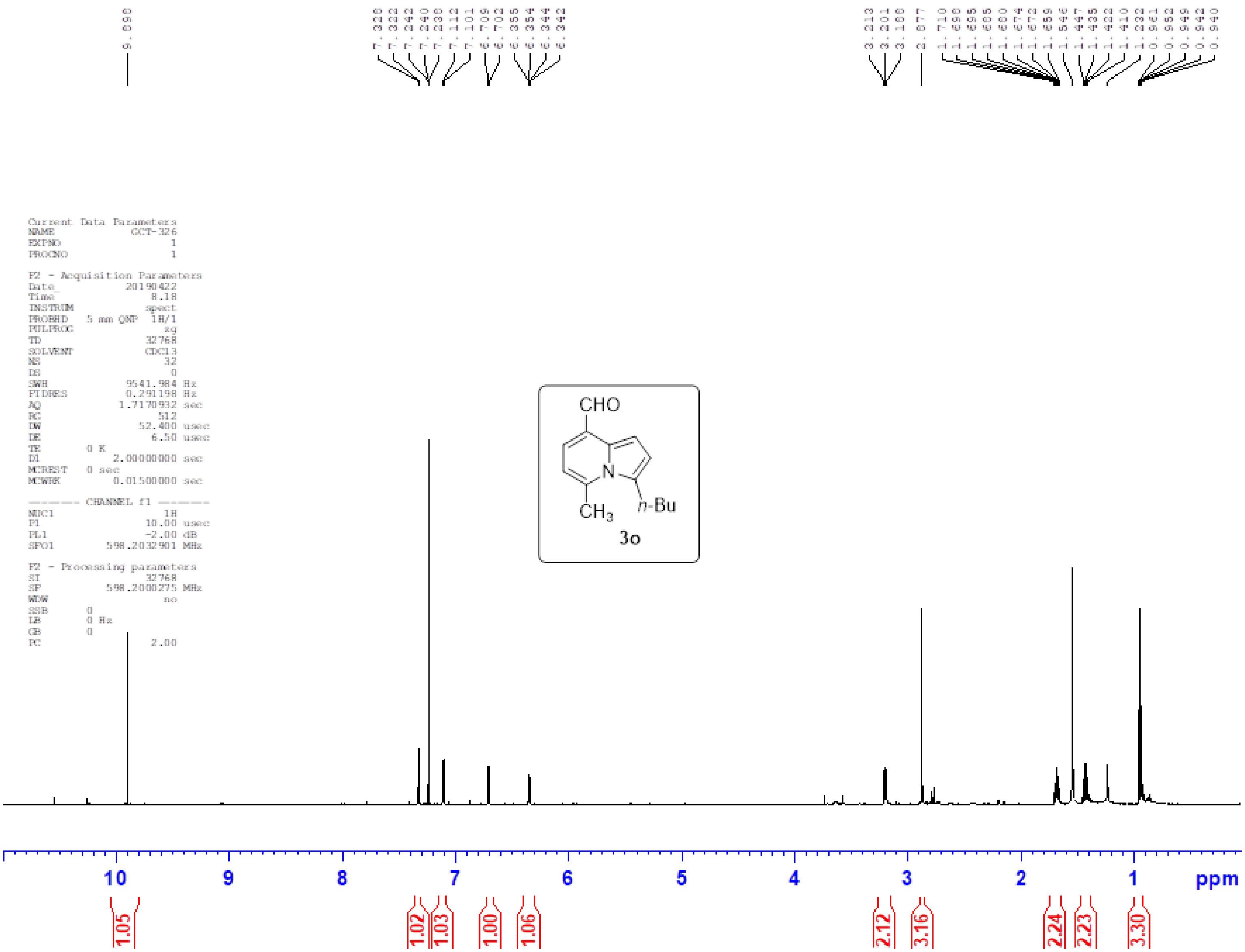
9.890
7.320
7.322
7.242
7.240
7.230
7.112
7.101
6.709
6.702
6.355
6.354
6.344
6.342
3.213
3.201
3.188
2.877
1.710
1.698
1.685
1.685
1.600
1.674
1.672
1.659
1.546
1.447
1.435
1.422
1.410
1.232
0.961
0.952
0.949
0.942
0.940
Current Data Parameters
NAME GCT-326
EXPNO 1
PROCNO 1
F2 - Acquisition Parameters
Date_ 20190422
Time 8.18
INSTRUM spect
PROBHD 5 mm QNP 1H/1
PULPROG zg
TD 32768
SOLVENT CDCl3
NS 32
DS 0
SWH 9541.984 Hz
FIDRES 0.291198 Hz
AQ 1.7170932 sec
RG 512
DW 52.400 usec
DE 6.50 usec
TE 0 K
D1 2.00000000 sec
MCREST 0 sec
MCWRK 0.01500000 sec
======== CHANNEL f1 ========
NUC1 1H
P1 10.00 usec
PL1 -2.00 dB
SFO1 598.2032901 MHz
F2 - Processing parameters
SI 32768
SF 598.2000275 MHz
WDW no
SSB 0
LB 0 Hz
GB 0
PC 2.00
CHO
N
CH3
n-Bu
3o
10
9
8
7
6
5
4
3
2
1
ppm
1.05
1.02
1.03
1.00
1.06
2.12
3.16
2.24
2.23
3.30

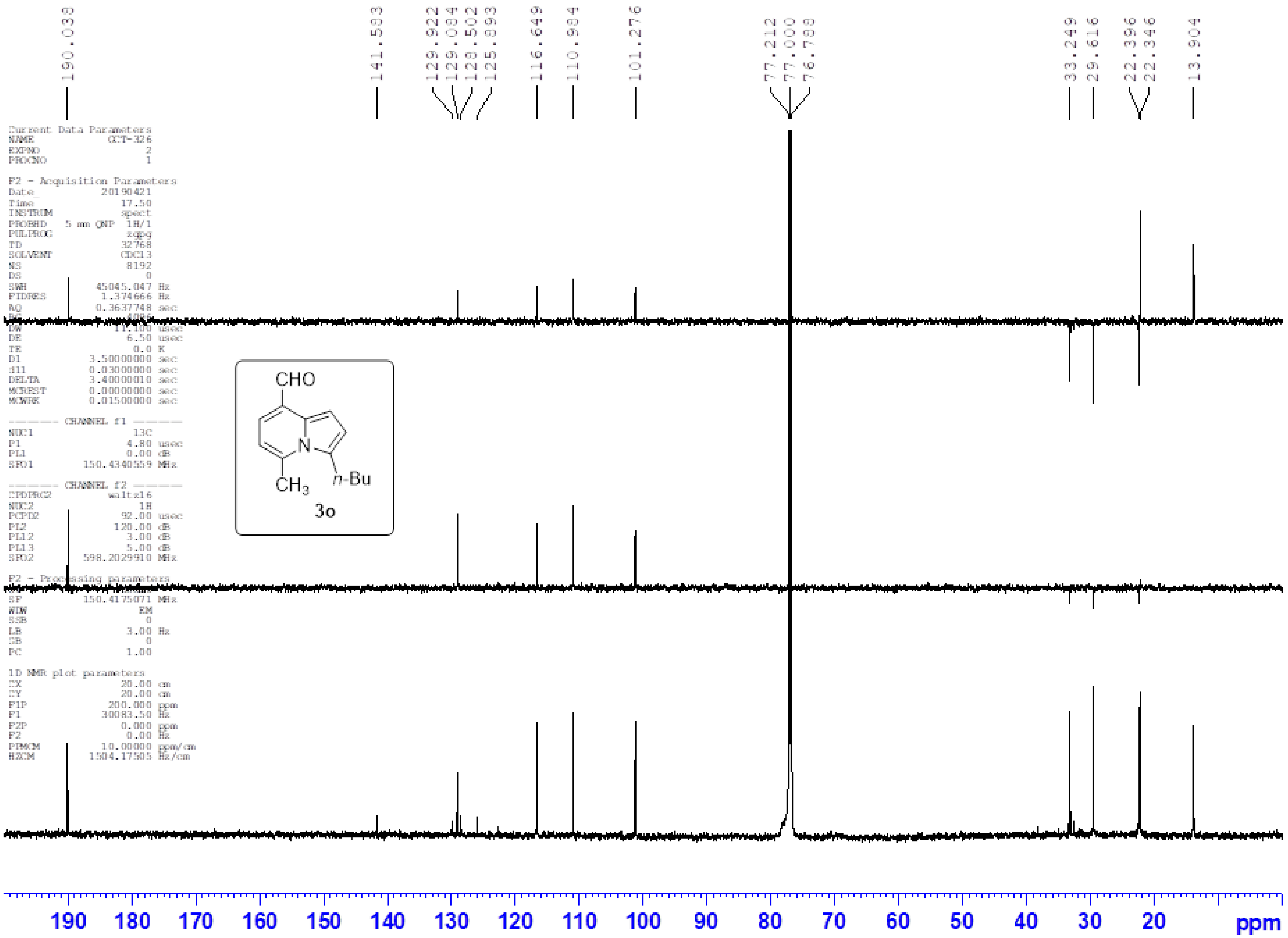
190.038
141.583
129.922
129.084
128.502
125.893
116.649
110.984
101.276
77.212
77.000
76.788
33.249
29.616
22.396
22.346
13.904
Current Data Parameters
NAME GCT-326
EXPNO 2
PROCNO 1
F2 - Acquisition Parameters
Date 20190421
Time 17.50
INSTRUM spect
PROBHD 5 mm QNP 1H/1
PULPROG zgpg
TD 32768
SOLVENT CDCl3
NS 8192
DS 0
SWH 45045.047 Hz
FIDRES 1.374666 Hz
AQ 0.3637748 sec
DE 6.50 usec
TE 0.0 K
D1 3.50000000 sec
d11 0.03000000 sec
DELTA 3.40000010 sec
MCREST 0.00000000 sec
MCWRK 0.01500000 sec
======== CHANNEL f1 ========
NUC1 13C
P1 4.80 usec
PL1 0.00 dB
SFO1 150.4340559 MHz
======== CHANNEL f2 ========
CPDPRG2 waltz16
NUC2 1H
PCPD2 92.00 usec
PL2 120.00 dB
PL12 3.00 dB
PL13 5.00 dB
SFO2 598.2029910 MHz
F2 - Processing parameters
SF 150.4175071 MHz
WDW EM
SSB 0
LB 3.00 Hz
GB 0
PC 1.00
1D NMR plot parameters
CX 20.00 cm
CY 20.00 cm
F1P 200.000 ppm
F1 30083.50 Hz
F2P 0.000 ppm
F2 0.00 Hz
PPMCM 10.00000 ppm/cm
HZCM 1504.17505 Hz/cm
CHO
N
CH3
n-Bu
3o
190
180
170
160
150
140
130
120
110
100
90
80
70
60
50
40
30
20
ppm

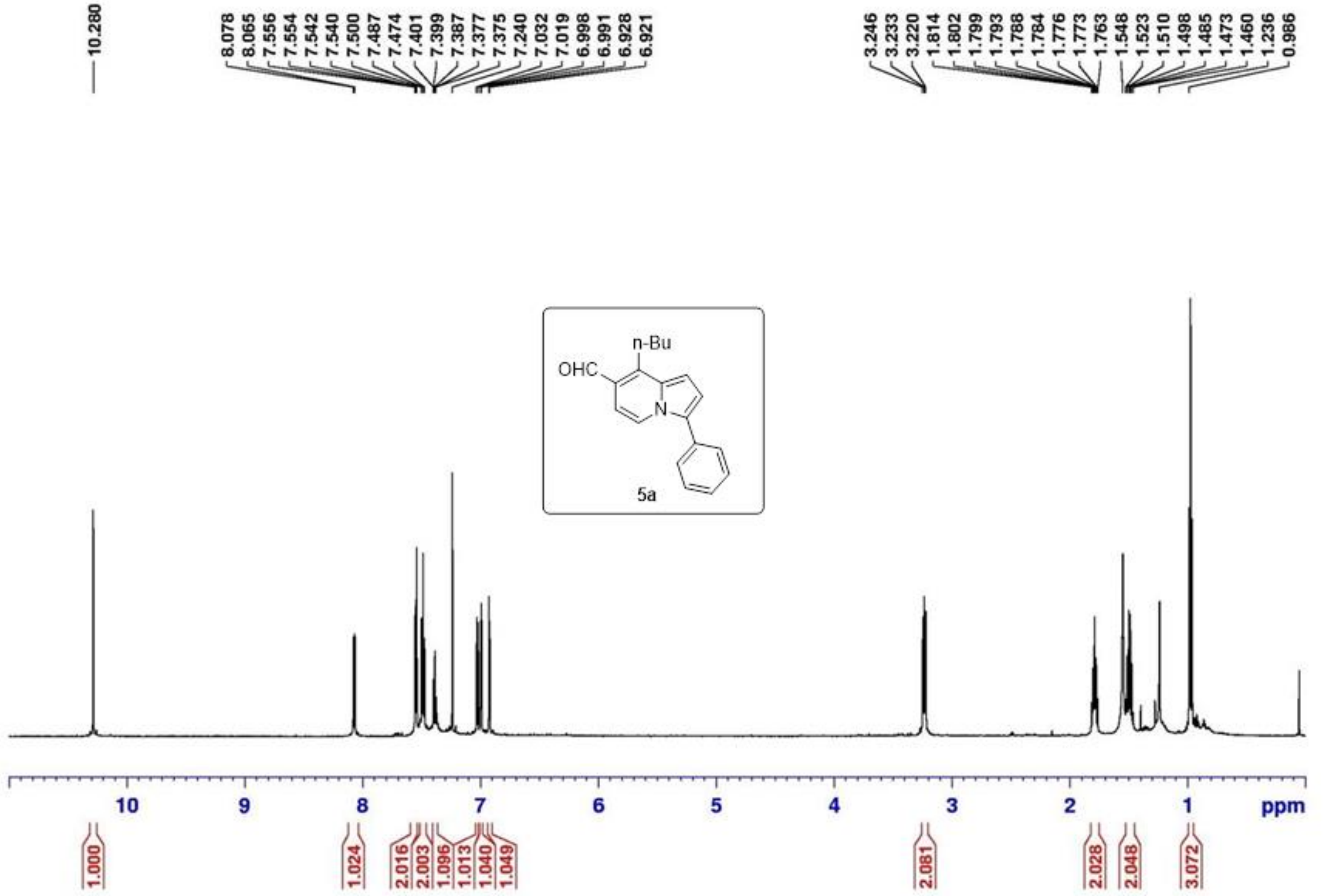
n-Bu
OHC
N
5a
ppm

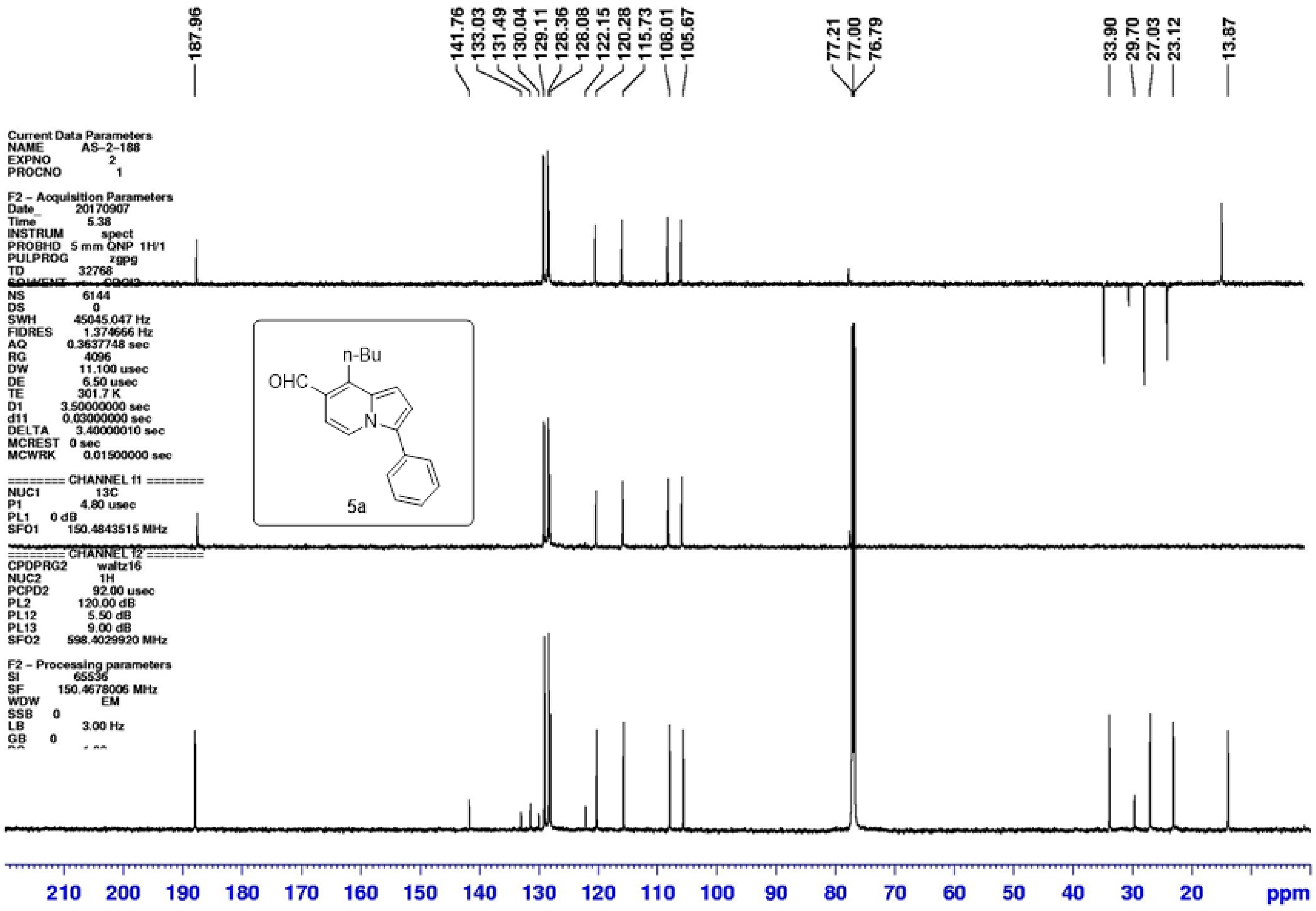
187.96
141.76
133.03
131.49
130.04
129.11
128.36
128.08
122.15
120.28
115.73
108.01
105.67
77.21
77.00
76.79
33.90
29.70
27.03
23.12
13.87
Current Data Parameters
NAME AS-2-188
EXPNO 2
PROCNO 1
F2 - Acquisition Parameters
Date_ 20170907
Time 5.38
INSTRUM spect
PROBHD 5 mm QNP 1H/1
PULPROG zgpg
TD 32768
NS 6144
DS 0
SWH 45045.047 Hz
FIDRES 1.374666 Hz
AQ 0.3637748 sec
RG 4096
DW 11.100 usec
DE 6.50 usec
TE 301.7 K
D1 3.50000000 sec
d11 0.03000000 sec
DELTA 3.40000010 sec
MCREST 0 sec
MCWRK 0.01500000 sec
======== CHANNEL f1 ========
NUC1 13C
P1 4.80 usec
PL1 0 dB
SFO1 150.4843515 MHz
======== CHANNEL f2 ========
CPDPRG2 waltz16
NUC2 1H
PCPD2 92.00 usec
PL2 120.00 dB
PL12 5.50 dB
PL13 9.00 dB
SFO2 598.4029920 MHz
F2 - Processing parameters
SI 65536
SF 150.4678006 MHz
WDW EM
SSB 0
LB 3.00 Hz
GB 0
n-Bu
OHC
N
5a
210 200 190 180 170 160 150 140 130 120 110 100 90 80 70 60 50 40 30 20 ppm

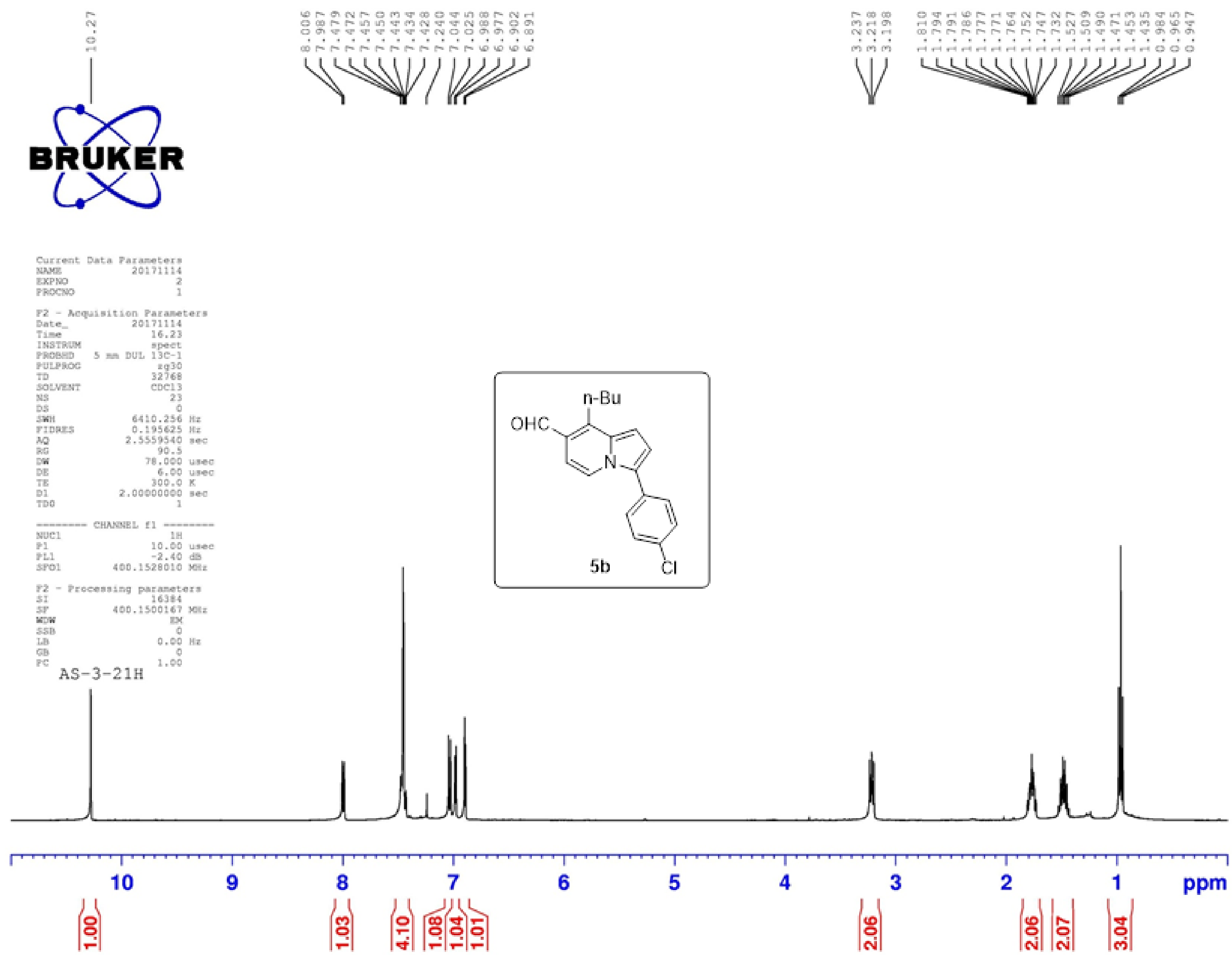
BRUKER
Current Data Parameters
NAME 20171114
EXPNO 2
PROCNO 1
F2 - Acquisition Parameters
Date_ 20171114
Time 16.23
INSTRUM spect
PROBHD 5 mm DUL 13C-1
PULPROG zg30
TD 32768
SOLVENT CDCl3
NS 23
DS 0
SWH 6410.256 Hz
FIDRES 0.195625 Hz
AQ 2.5559540 sec
RG 90.5
DW 78.000 usec
DE 6.00 usec
TE 300.0 K
D1 2.00000000 sec
TD0 1
======== CHANNEL f1 ========
NUC1 1H
P1 10.00 usec
PL1 -2.40 dB
SFO1 400.1528010 MHz
F2 - Processing parameters
SI 16384
SF 400.1500167 MHz
WDW EM
SSB 0
LB 0.00 Hz
GB 0
PC 1.00
AS-3-21H
n-Bu
OHC
N
Cl
5b
10.27
8.006
7.987
7.479
7.472
7.457
7.450
7.443
7.434
7.428
7.240
7.044
7.025
6.988
6.977
6.902
6.891
3.237
3.218
3.198
1.810
1.794
1.791
1.786
1.777
1.771
1.764
1.752
1.747
1.732
1.527
1.509
1.490
1.471
1.453
1.435
0.984
0.965
0.947
10
9
8
7
6
5
4
3
2
1
ppm
1.00
1.03
4.10
1.08
1.04
1.01
2.06
2.06
2.07
3.04

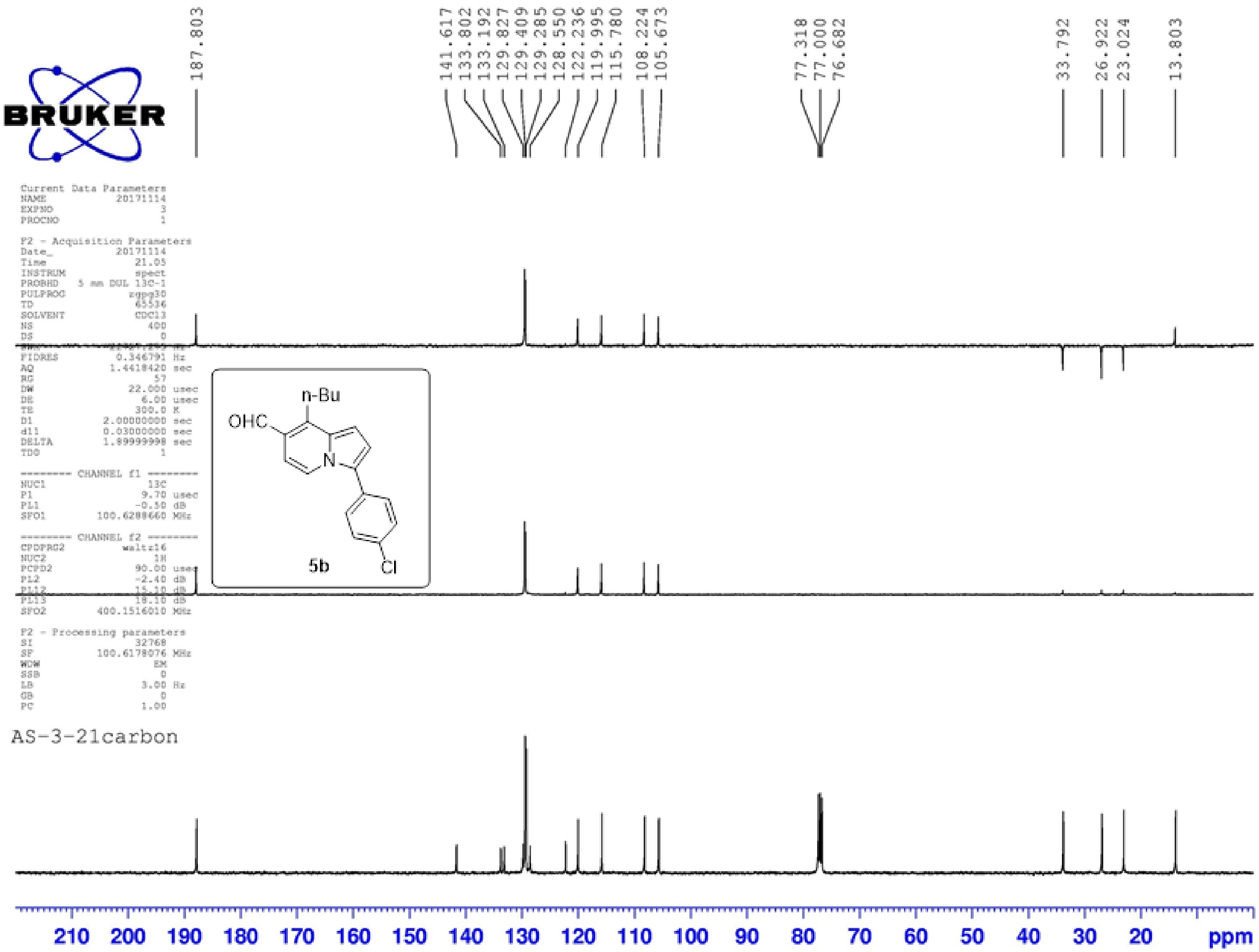
BRUKER
187.803
141.617
133.802
133.192
129.827
129.409
129.285
128.550
122.236
119.995
115.780
108.224
105.673
77.318
77.000
76.682
33.792
26.922
23.024
13.803
Current Data Parameters
NAME 20171114
EXPNO 3
PROCNO 1
F2 - Acquisition Parameters
Date_ 20171114
Time 21.05
INSTRUM spect
PROBHD 5 mm DUL 13C-1
PULPROG zgpg30
TD 65536
SOLVENT CDCl3
NS 400
DS 0
FIDRES 0.346791 Hz
AQ 1.4418420 sec
RG 57
DW 22.000 usec
DE 6.00 usec
TE 300.0 K
D1 2.00000000 sec
d11 0.03000000 sec
DELTA 1.89999998 sec
TD0 1
======== CHANNEL f1 ========
NUC1 13C
P1 9.70 usec
PL1 -0.50 dB
SFO1 100.6288660 MHz
======== CHANNEL f2 ========
CPDPRG2 waltz16
NUC2 1H
PCPD2 90.00 usec
PL2 -2.40 dB
PL13 18.10 dB
SFO2 400.1516010 MHz
F2 - Processing parameters
SI 32768
SF 100.6178076 MHz
WDW EM
SSB 0
LB 3.00 Hz
GB 0
PC 1.00
n-Bu
OHC
N
Cl
5b
AS-3-21carbon
210 200 190 180 170 160 150 140 130 120 110 100 90 80 70 60 50 40 30 20 ppm

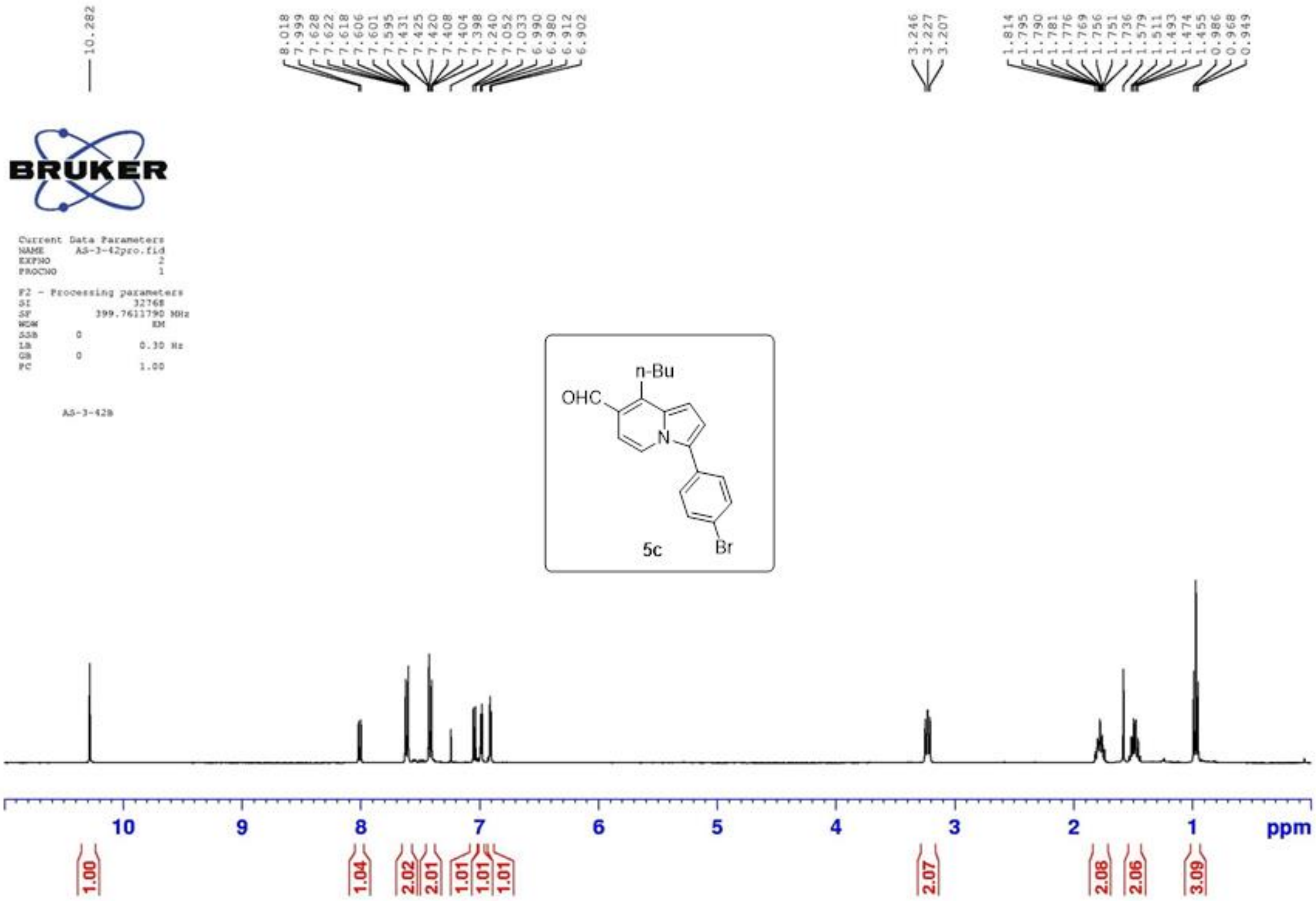

10.282
8.018
7.999
7.628
7.622
7.618
7.606
7.601
7.595
7.431
7.425
7.420
7.408
7.404
7.398
7.240
7.052
7.033
6.990
6.980
6.912
6.902
3.246
3.227
3.207
1.814
1.795
1.790
1.781
1.776
1.769
1.756
1.751
1.736
1.579
1.511
1.493
1.474
1.455
0.986
0.968
0.949
BRUKER
Current Data Parameters
NAME AS-3-42pro.fid
EXPNO 2
PROCNO 1
F2 - Processing parameters
SI 32768
SF 399.7611790 MHz
WDW EM
SSB 0
LB 0.30 Hz
GB 0
PC 1.00
AS-3-42B
n-Bu
OHC
N
Br
5c
10
9
8
7
6
5
4
3
2
1
ppm
1.00
1.04
2.02
2.01
1.01
1.01
1.01
2.07
2.08
2.06
3.09

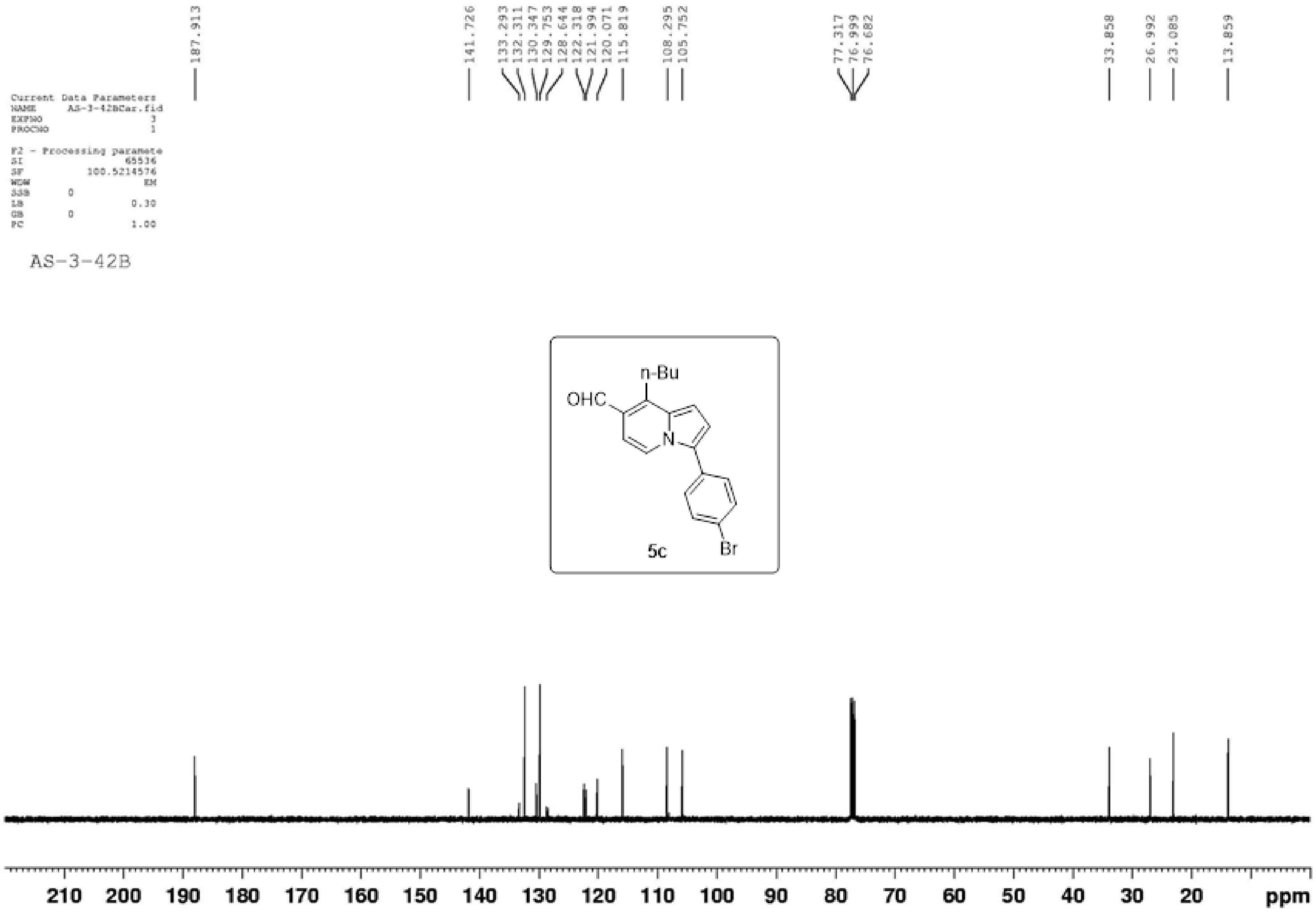
187.913
141.726
133.293
132.311
130.347
129.753
128.644
122.318
121.994
120.071
115.819
108.295
105.752
77.317
76.999
76.682
33.858
26.992
23.085
13.859
Current Data Parameters
NAME AS-3-42BCar.fid
EXPNO 3
PROCNO 1
F2 - Processing paramete
SI 65536
SF 100.5214576
WDW EM
SSB 0
LB 0.30
GB 0
PC 1.00
AS-3-42B
n-Bu
OHC
N
5c
Br
210 200 190 180 170 160 150 140 130 120 110 100 90 80 70 60 50 40 30 20 ppm

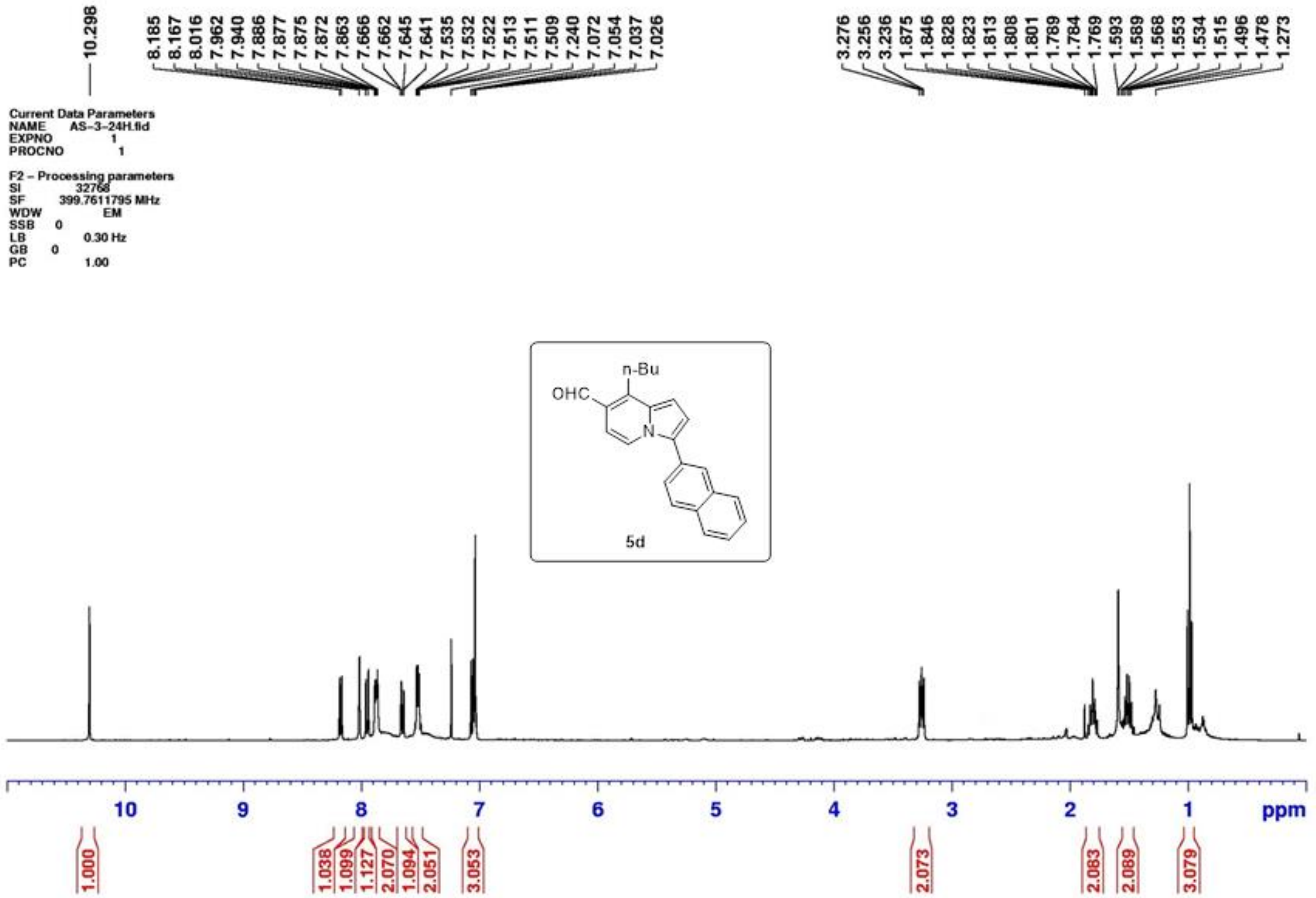
10.298
8.185
8.167
8.016
7.962
7.940
7.886
7.877
7.875
7.872
7.863
7.666
7.662
7.645
7.641
7.535
7.532
7.522
7.513
7.511
7.509
7.240
7.072
7.054
7.037
7.026
3.276
3.256
3.236
1.875
1.846
1.828
1.823
1.813
1.808
1.801
1.789
1.784
1.769
1.593
1.589
1.568
1.553
1.534
1.515
1.496
1.478
1.273
Current Data Parameters
NAME AS-3-24H.fid
EXPNO 1
PROCNO 1
F2 - Processing parameters
SI 32768
SF 399.7611795 MHz
WDW EM
SSB 0
LB 0.30 Hz
GB 0
PC 1.00
n-Bu
OHC
N
5d
10
9
8
7
6
5
4
3
2
1
ppm
1.000
1.038
1.099
1.127
2.070
1.094
2.051
3.053
2.073
2.083
2.089
3.079

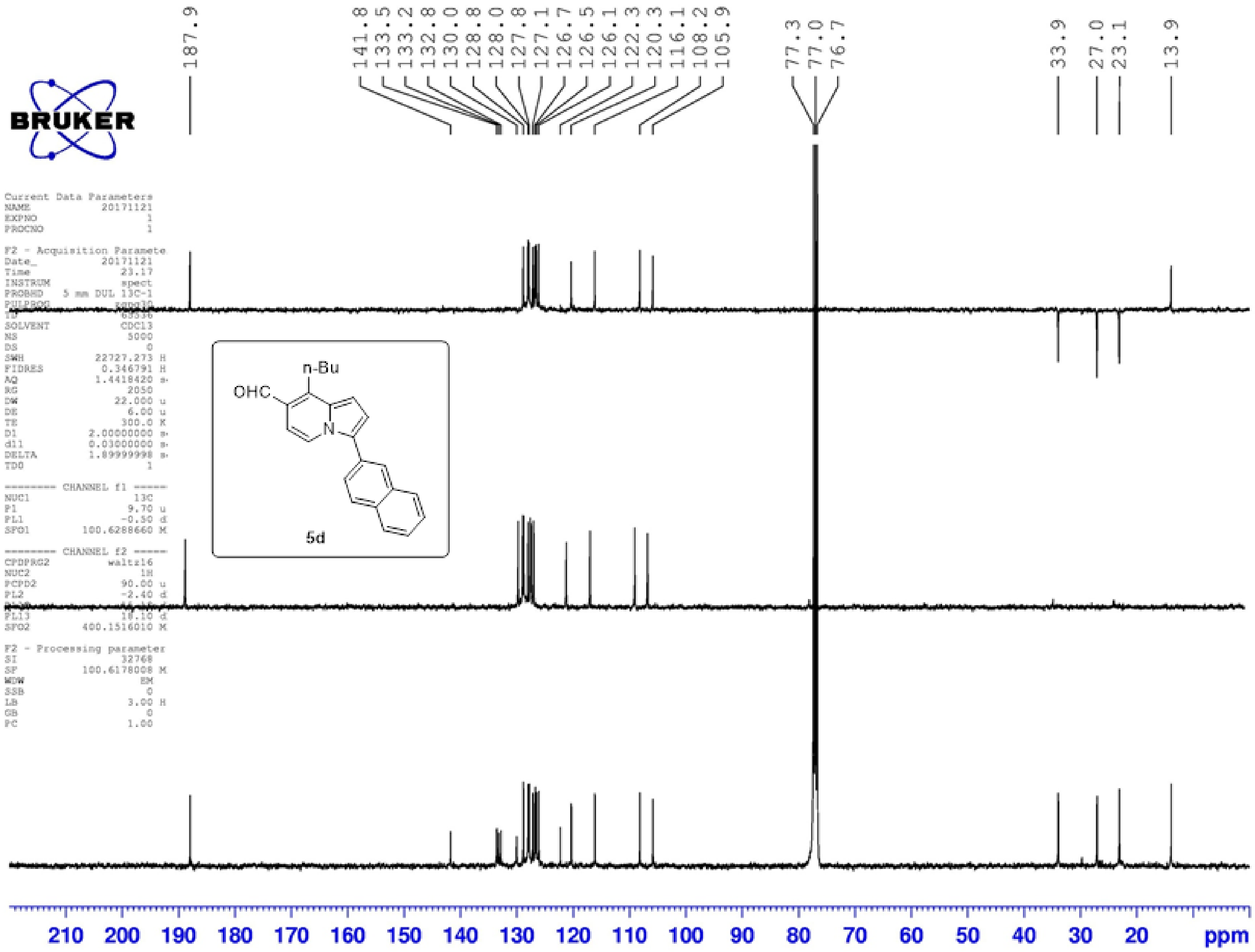

187.9
141.8
133.5
133.2
132.8
130.0
128.8
128.0
127.8
127.1
126.7
126.5
126.1
122.3
120.3
116.1
108.2
105.9
77.3
77.0
76.7
33.9
27.0
23.1
13.9
BRUKER
Current Data Parameters
NAME 20171121
EXPNO 1
PROCNO 1
F2 - Acquisition Paramete
Date_ 20171121
Time 23.17
INSTRUM spect
PROBHD 5 mm DUL 13C-1
SOLVENT CDCl3
NS 5000
DS 0
SWH 22727.273 H
FIDRES 0.346791 H
AQ 1.4418420 s
RG 2050
DW 22.000 u
DE 6.00 u
TE 300.0 K
D1 2.00000000 s
d11 0.03000000 s
DELTA 1.89999998 s
TD0 1
======== CHANNEL f1 =====
NUC1 13C
P1 9.70 u
PL1 -0.50 d
SFO1 100.6288660 M
======== CHANNEL f2 =====
CPDPRG2 waltz16
NUC2 1H
PCPD2 90.00 u
PL2 -2.40 d
PL13 18.10 d
SFO2 400.1516010 M
F2 - Processing parameter
SI 32768
SF 100.6178008 M
WDW EM
SSB 0
LB 3.00 H
GB 0
PC 1.00
n-Bu
OHC
N
5d
210 200 190 180 170 160 150 140 130 120 110 100 90 80 70 60 50 40 30 20 ppm

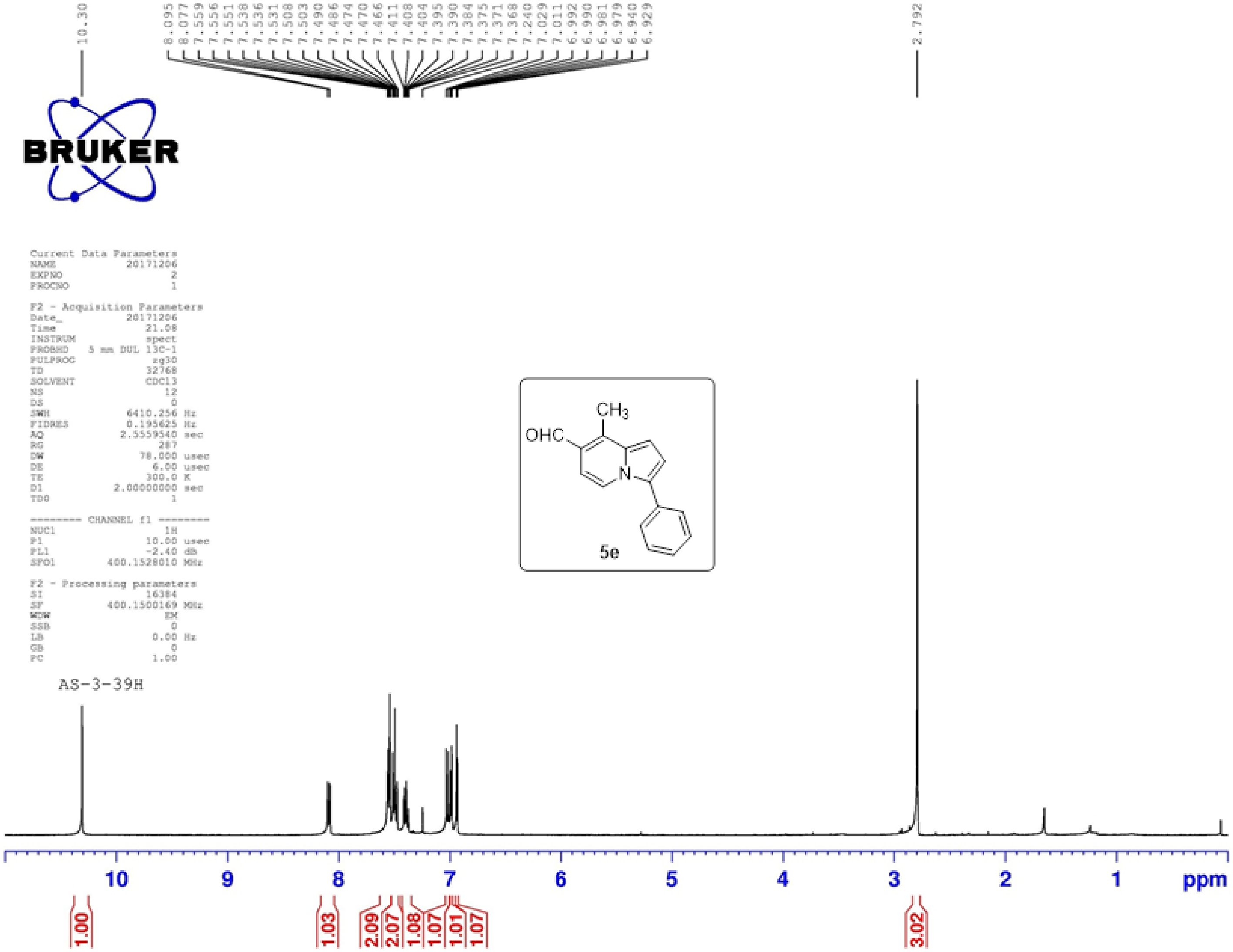
10.30
8.095
8.077
7.559
7.556
7.551
7.538
7.536
7.531
7.508
7.503
7.490
7.486
7.474
7.470
7.466
7.411
7.408
7.404
7.395
7.390
7.384
7.375
7.371
7.368
7.240
7.029
7.011
6.992
6.990
6.981
6.979
6.940
6.929
2.792
BRUKER
Current Data Parameters
NAME 20171206
EXPNO 2
PROCNO 1
F2 - Acquisition Parameters
Date_ 20171206
Time 21.08
INSTRUM spect
PROBHD 5 mm DUL 13C-1
PULPROG zg30
TD 32768
SOLVENT CDCl3
NS 12
DS 0
SWH 6410.256 Hz
FIDRES 0.195625 Hz
AQ 2.5559540 sec
RG 287
DW 78.000 usec
DE 6.00 usec
TE 300.0 K
D1 2.00000000 sec
TD0 1
======== CHANNEL f1 ========
NUC1 1H
P1 10.00 usec
PL1 -2.40 dB
SFO1 400.1528010 MHz
F2 - Processing parameters
SI 16384
SF 400.1500169 MHz
WDW EM
SSB 0
LB 0.00 Hz
GB 0
PC 1.00
AS-3-39H
CH3
OHC
N
5e
10
9
8
7
6
5
4
3
2
1
ppm
1.00
1.03
2.09
2.07
1.08
1.07
1.01
1.07
3.02

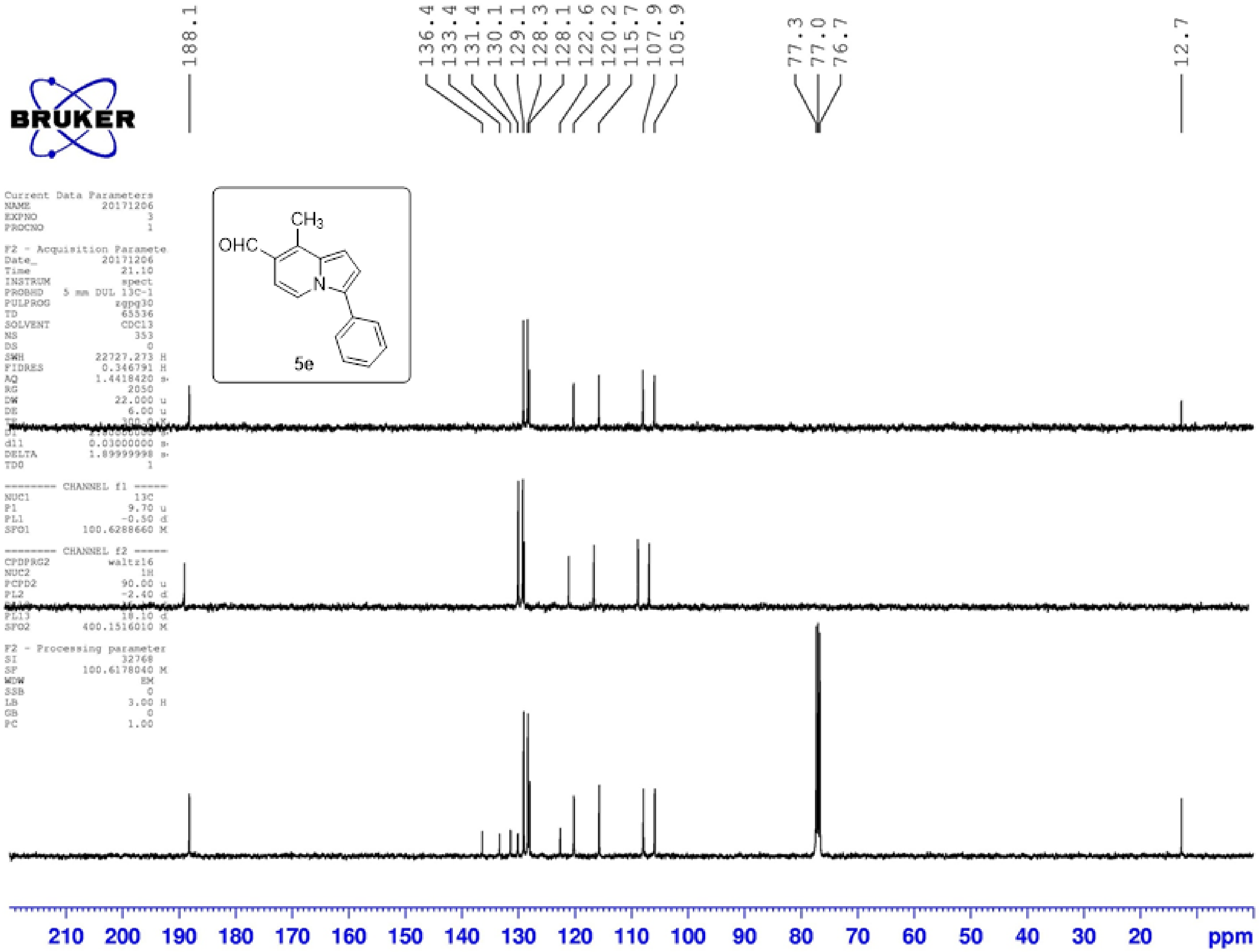
188.1
136.4
133.4
131.4
130.1
129.1
128.3
128.1
122.6
120.2
115.7
107.9
105.9
77.3
77.0
76.7
12.7
BRUKER
OHC
CH3
N
5e
Current Data Parameters
NAME 20171206
EXPNO 3
PROCNO 1
F2 - Acquisition Paramete
Date_ 20171206
Time 21.10
INSTRUM spect
PROBHD 5 mm DUL 13C-1
PULPROG zgpg30
TD 65536
SOLVENT CDCl3
NS 353
DS 0
SWH 22727.273 H
FIDRES 0.346791 H
AQ 1.4418420 s
RG 2050
DW 22.000 u
DE 6.00 u
d11 0.03000000 s
DELTA 1.89999998 s
TD0 1
======== CHANNEL f1 =====
NUC1 13C
P1 9.70 u
PL1 -0.50 d
SFO1 100.6288660 M
======== CHANNEL f2 =====
CPDPRG2 waltz16
NUC2 1H
PCPD2 90.00 u
PL2 -2.40 d
PL13 18.10 d
SFO2 400.1516010 M
F2 - Processing parameter
SI 32768
SF 100.6178040 M
WDW EM
SSB 0
LB 3.00 H
GB 0
PC 1.00
210
200
190
180
170
160
150
140
130
120
110
100
90
80
70
60
50
40
30
20
ppm

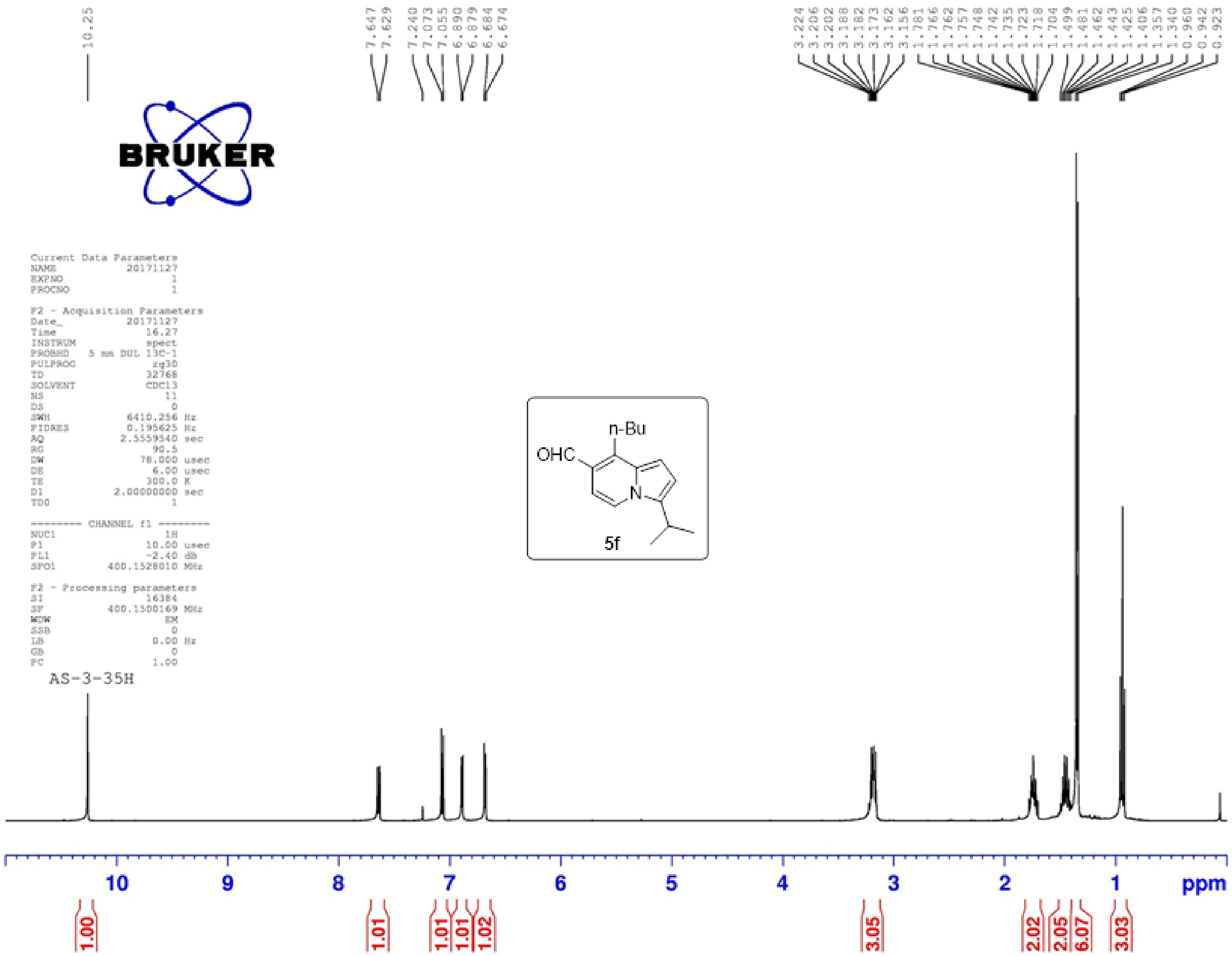

10.25
7.647
7.629
7.240
7.073
7.055
6.890
6.879
6.684
6.674
3.224
3.206
3.202
3.188
3.182
3.173
3.162
3.156
1.781
1.766
1.762
1.757
1.748
1.742
1.735
1.723
1.718
1.704
1.499
1.481
1.462
1.443
1.425
1.406
1.357
1.340
0.960
0.942
0.923
Current Data Parameters
NAME 20171127
EXPNO 1
PROCNO 1
F2 - Acquisition Parameters
Date_ 20171127
Time 16.27
INSTRUM spect
PROBHD 5 mm DUL 13C-1
PULPROG zg30
TD 32768
SOLVENT CDCl3
NS 11
DS 0
SWH 6410.256 Hz
FIDRES 0.195625 Hz
AQ 2.5559540 sec
RG 90.5
DW 78.000 usec
DE 6.00 usec
TE 300.0 K
D1 2.00000000 sec
TD0 1
======== CHANNEL f1 ========
NUC1 1H
P1 10.00 usec
PL1 -2.40 dB
SFO1 400.1528010 MHz
F2 - Processing parameters
SI 16384
SF 400.1500169 MHz
WDW EM
SSB 0
LB 0.00 Hz
GB 0
PC 1.00
AS-3-35H
n-Bu
OHC
N
5f
10
9
8
7
6
5
4
3
2
1
ppm
1.00
1.01
1.01
1.01
1.02
3.05
2.02
2.05
6.07
3.03

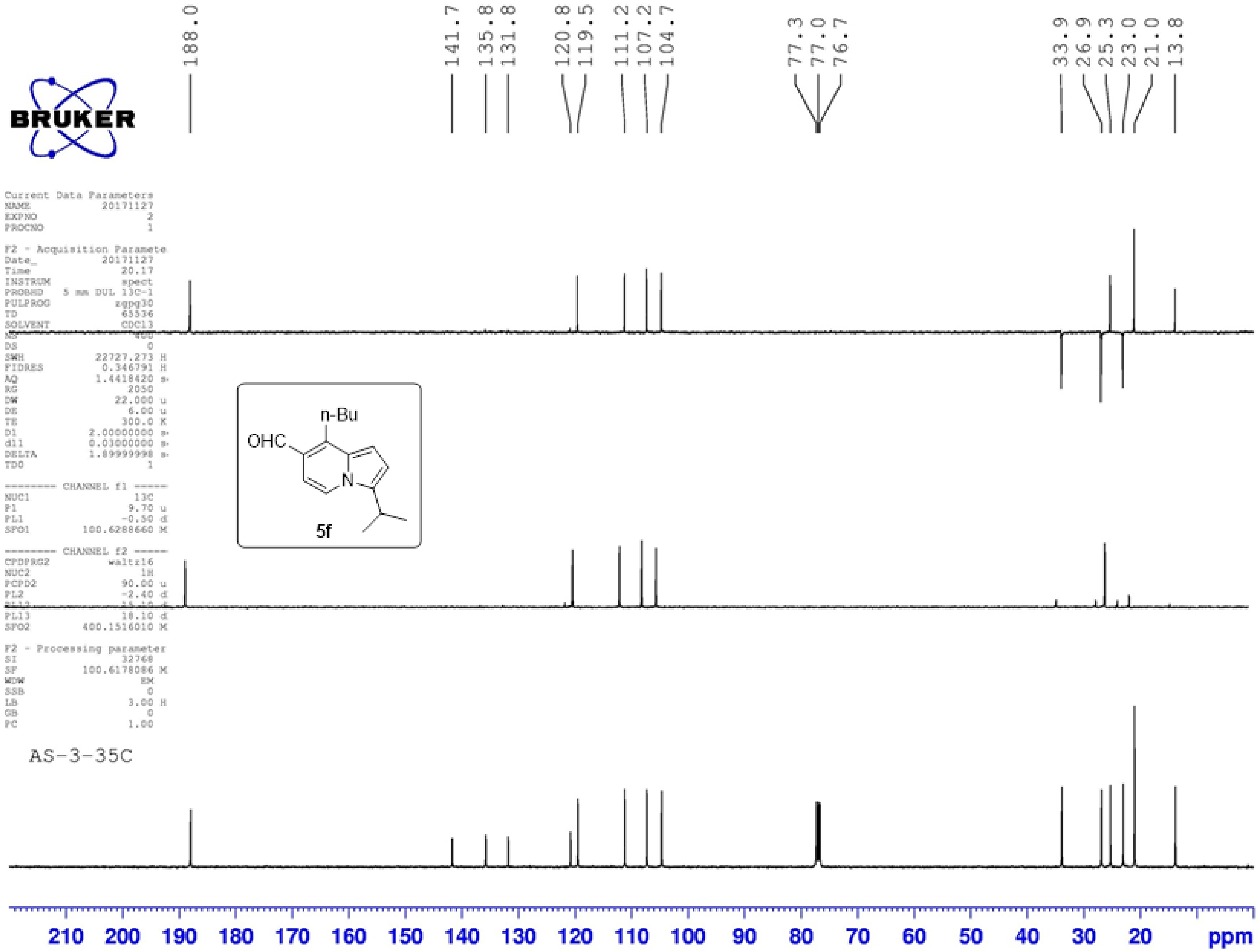
188.0
141.7
135.8
131.8
120.8
119.5
111.2
107.2
104.7
77.3
77.0
76.7
33.9
26.9
25.3
23.0
21.0
13.8
n-Bu
OHC
N
5f
AS-3-35C
210 200 190 180 170 160 150 140 130 120 110 100 90 80 70 60 50 40 30 20 ppm

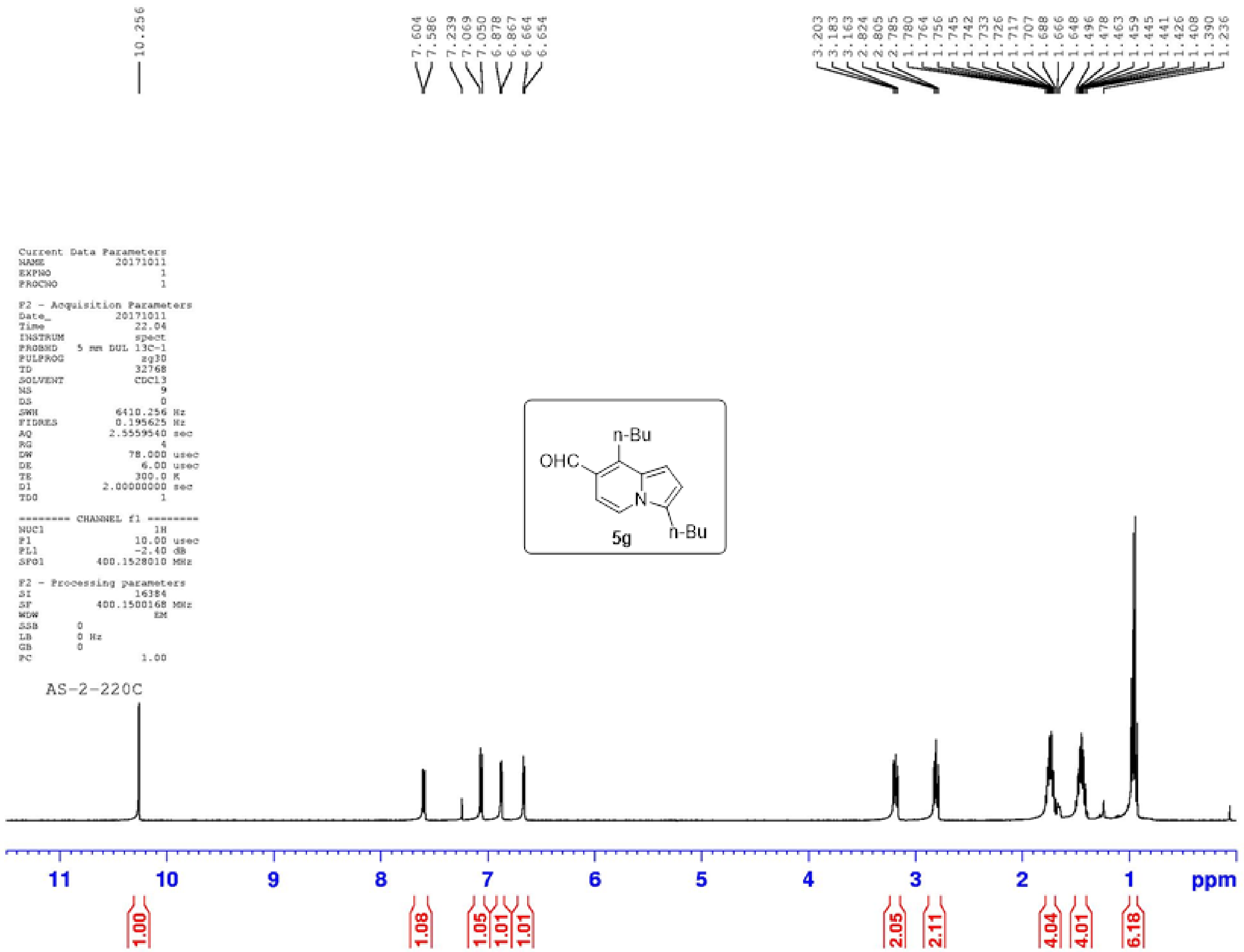

10.256
7.604
7.586
7.239
7.069
7.050
6.878
6.867
6.664
6.654
3.203
3.183
3.163
2.824
2.805
2.785
1.780
1.764
1.756
1.745
1.742
1.733
1.726
1.717
1.707
1.688
1.666
1.648
1.496
1.478
1.463
1.459
1.445
1.441
1.426
1.408
1.390
1.236
Current Data Parameters
NAME 20171011
EXPNO 1
PROCNO 1
F2 - Acquisition Parameters
Date_ 20171011
Time 22.04
INSTRUM spect
PROBHD 5 mm DUL 13C-1
PULPROG zg30
TD 32768
SOLVENT CDCl3
NS 9
DS 0
SWH 6410.256 Hz
FIDRES 0.195625 Hz
AQ 2.5559540 sec
RG 4
DW 78.000 usec
DE 6.00 usec
TE 300.0 K
D1 2.00000000 sec
TD0 1
======== CHANNEL f1 ========
NUC1 1H
P1 10.00 usec
PL1 -2.40 dB
SFO1 400.1528010 MHz
F2 - Processing parameters
SI 16384
SF 400.1500168 MHz
WDW EM
SSB 0
LB 0 Hz
GB 0
PC 1.00
AS-2-220C
n-Bu
OHC
N
n-Bu
5g
11
10
9
8
7
6
5
4
3
2
1
ppm
1.00
1.08
1.05
1.01
1.01
2.05
2.11
4.04
4.01
6.18

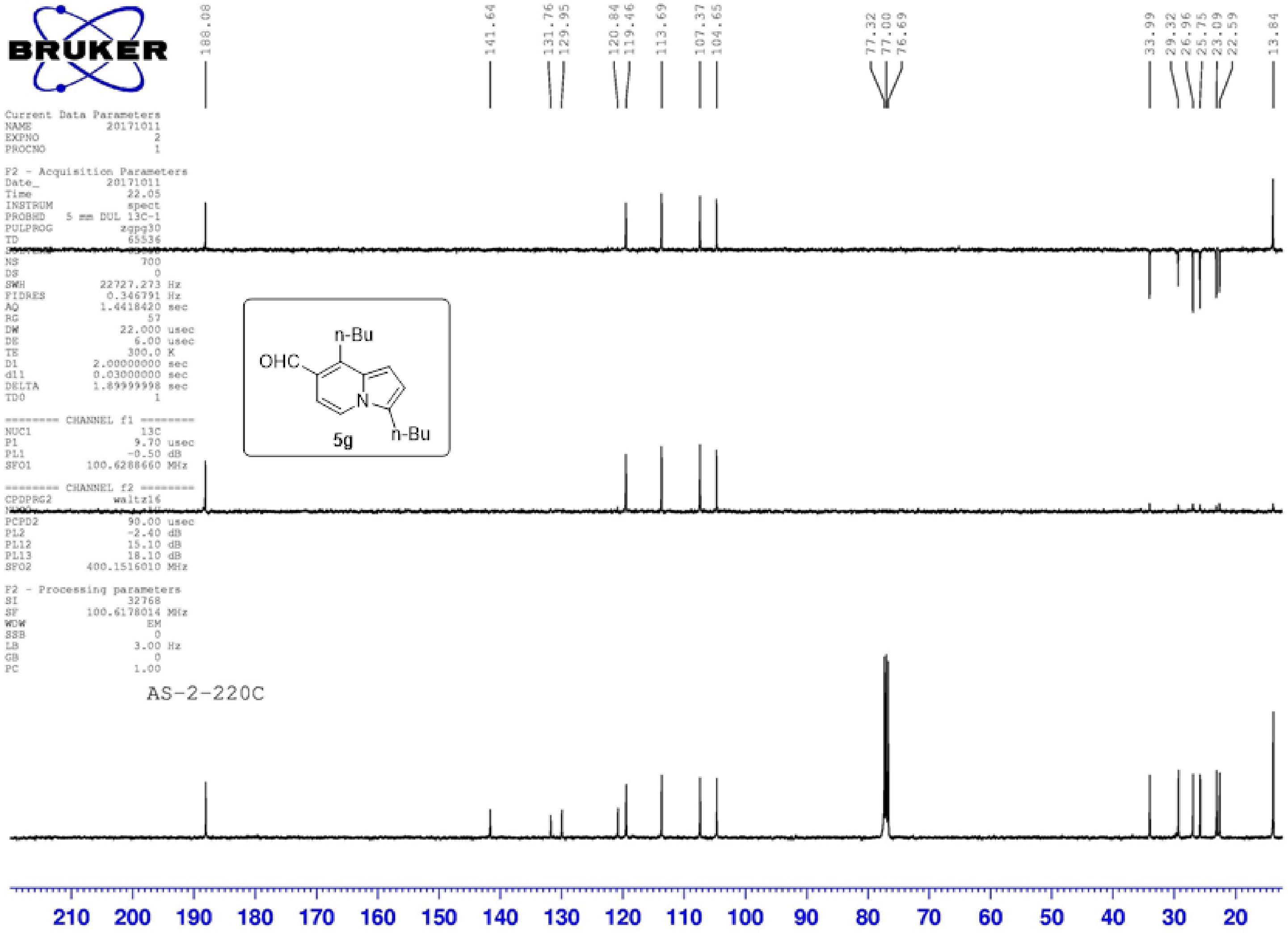

BRUKER
188.08
141.64
131.76
129.95
120.84
119.46
113.69
107.37
104.65
77.32
77.00
76.69
33.99
29.32
26.96
25.75
23.09
22.59
13.84
Current Data Parameters
NAME 20171011
EXPNO 2
PROCNO 1
F2 - Acquisition Parameters
Date_ 20171011
Time 22.05
INSTRUM spect
PROBHD 5 mm DUL 13C-1
PULPROG zgpg30
TD 65536
NS 700
DS 0
SWH 22727.273 Hz
FIDRES 0.346791 Hz
AQ 1.4418420 sec
RG 57
DW 22.000 usec
DE 6.00 usec
TE 300.0 K
D1 2.00000000 sec
d11 0.03000000 sec
DELTA 1.89999998 sec
TD0 1
======== CHANNEL f1 ========
NUC1 13C
P1 9.70 usec
PL1 -0.50 dB
SFO1 100.6288660 MHz
======== CHANNEL f2 ========
CPDPRG2 waltz16
PCPD2 90.00 usec
PL2 -2.40 dB
PL12 15.10 dB
PL13 18.10 dB
SFO2 400.1516010 MHz
F2 - Processing parameters
SI 32768
SF 100.6178014 MHz
WDW EM
SSB 0
LB 3.00 Hz
GB 0
PC 1.00
AS-2-220C
n-Bu
OHC
N
n-Bu
5g
210 200 190 180 170 160 150 140 130 120 110 100 90 80 70 60 50 40 30 20

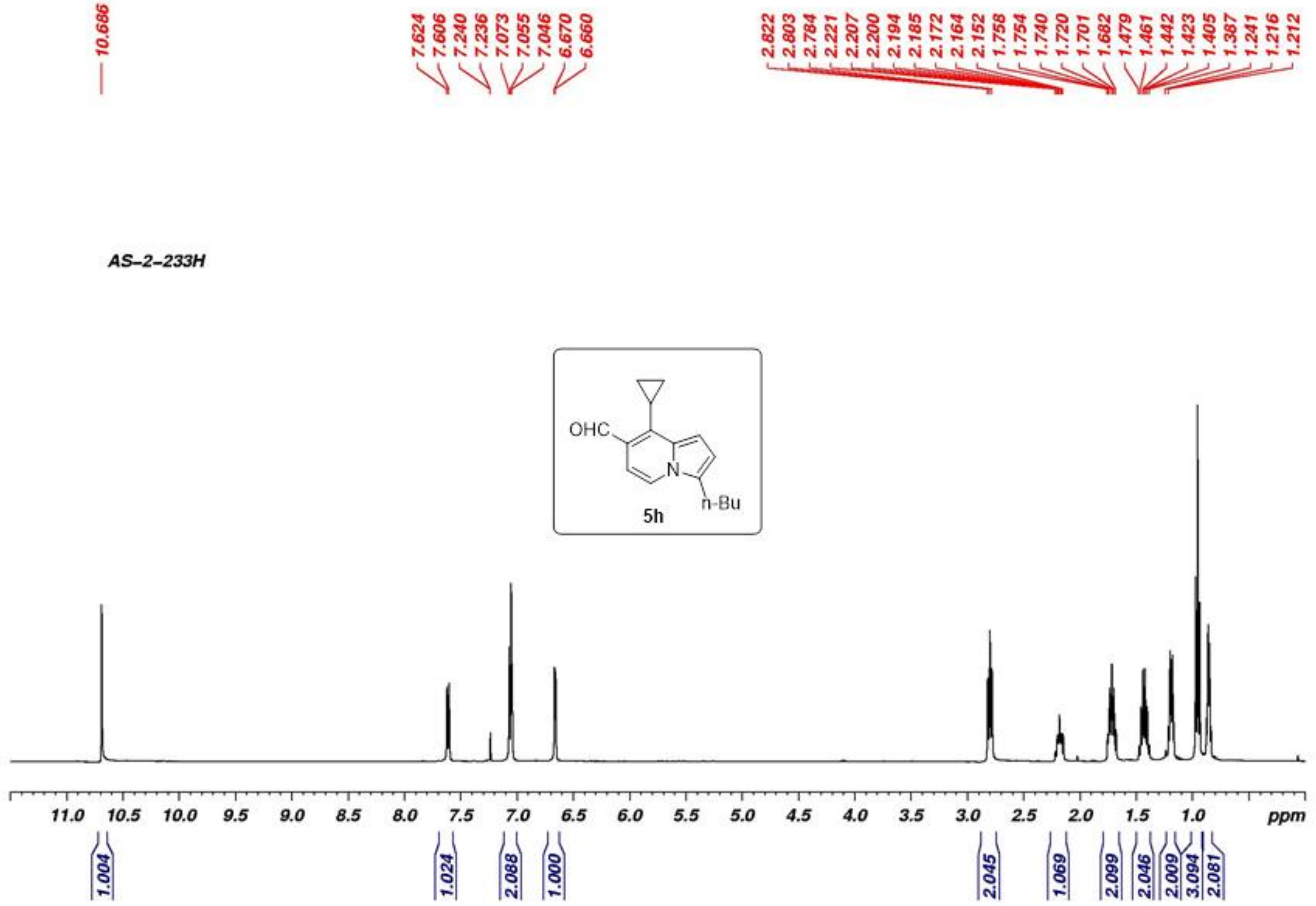

10.686
7.624
7.606
7.240
7.236
7.073
7.055
7.046
6.670
6.660
2.822
2.803
2.784
2.221
2.207
2.200
2.194
2.185
2.172
2.164
2.152
1.758
1.754
1.740
1.720
1.701
1.682
1.479
1.461
1.442
1.423
1.405
1.387
1.241
1.216
1.212
AS-2-233H
OHC
N
n-Bu
5h
11.0 10.5 10.0 9.5 9.0 8.5 8.0 7.5 7.0 6.5 6.0 5.5 5.0 4.5 4.0 3.5 3.0 2.5 2.0 1.5 1.0 ppm
1.004
1.024
2.088
1.000
2.045
1.069
2.099
2.046
2.009
3.094
2.081

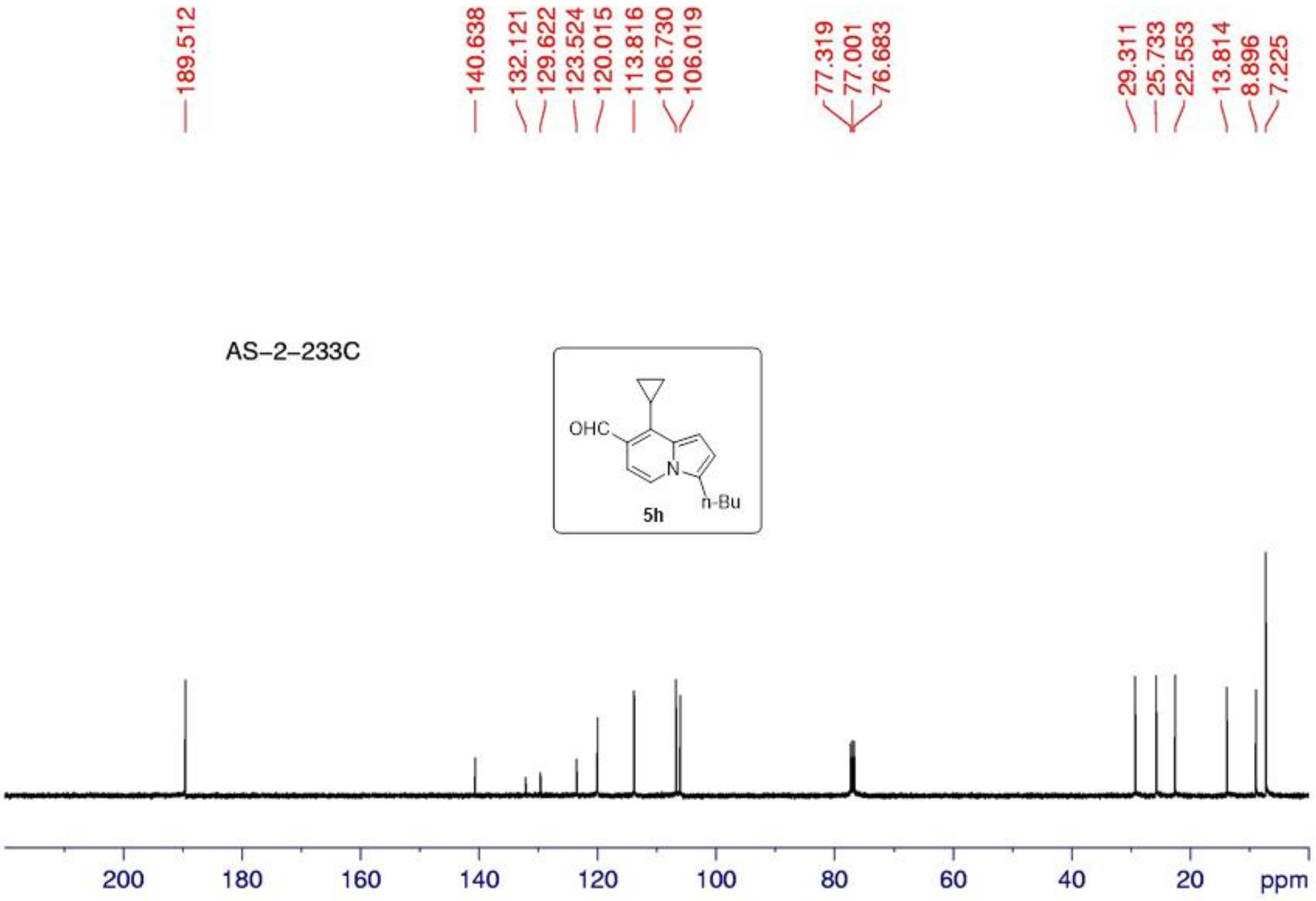
189.512
140.638
132.121
129.622
123.524
120.015
113.816
106.730
106.019
77.319
77.001
76.683
29.311
25.733
22.553
13.814
8.896
7.225
AS-2-233C
OHC
N
n-Bu
5h
200
180
160
140
120
100
80
60
40
20
ppm

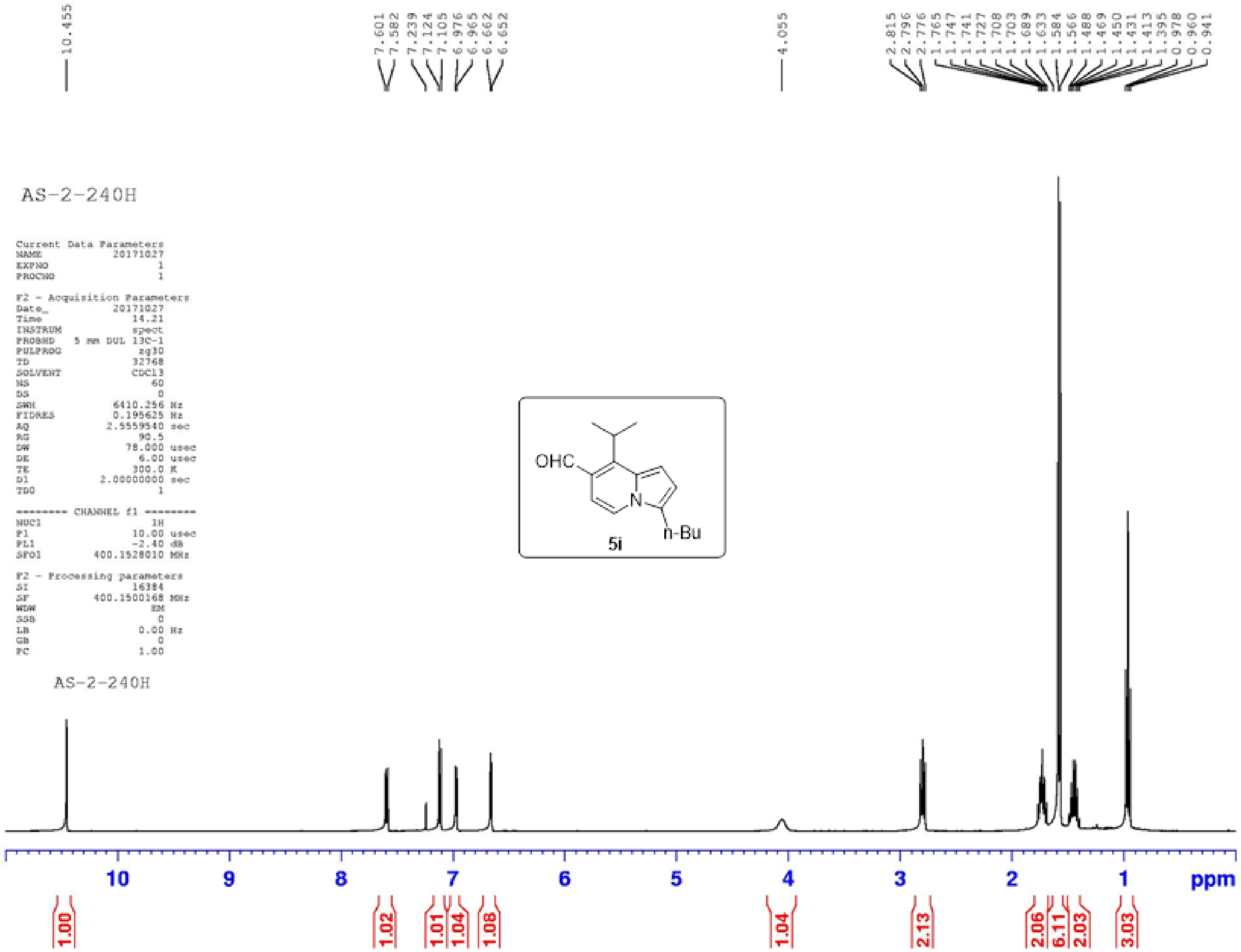

10.455
7.601
7.582
7.239
7.124
7.105
6.976
6.965
6.662
6.652
4.055
2.815
2.796
2.776
1.765
1.747
1.741
1.727
1.708
1.703
1.689
1.633
1.584
1.566
1.488
1.469
1.450
1.431
1.413
1.395
0.978
0.960
0.941
AS-2-240H
Current Data Parameters
NAME 20171027
EXPNO 1
PROCNO 1
F2 - Acquisition Parameters
Date_ 20171027
Time 14.21
INSTRUM spect
PROBHD 5 mm DUL 13C-1
PULPROG zg30
TD 32768
SOLVENT CDCl3
NS 60
DS 0
SWH 6410.256 Hz
FIDRES 0.195625 Hz
AQ 2.5559540 sec
RG 90.5
DW 78.000 usec
DE 6.00 usec
TE 300.0 K
D1 2.00000000 sec
TD0 1
======== CHANNEL f1 ========
NUC1 1H
P1 10.00 usec
PL1 -2.40 dB
SFO1 400.1528010 MHz
F2 - Processing parameters
SI 16384
SF 400.1500168 MHz
WDW EM
SSB 0
LB 0.00 Hz
GB 0
PC 1.00
AS-2-240H
OHC
N
n-Bu
5i
10
9
8
7
6
5
4
3
2
1
ppm
1.00
1.02
1.01
1.04
1.08
1.04
2.13
2.06
6.11
2.03
3.03

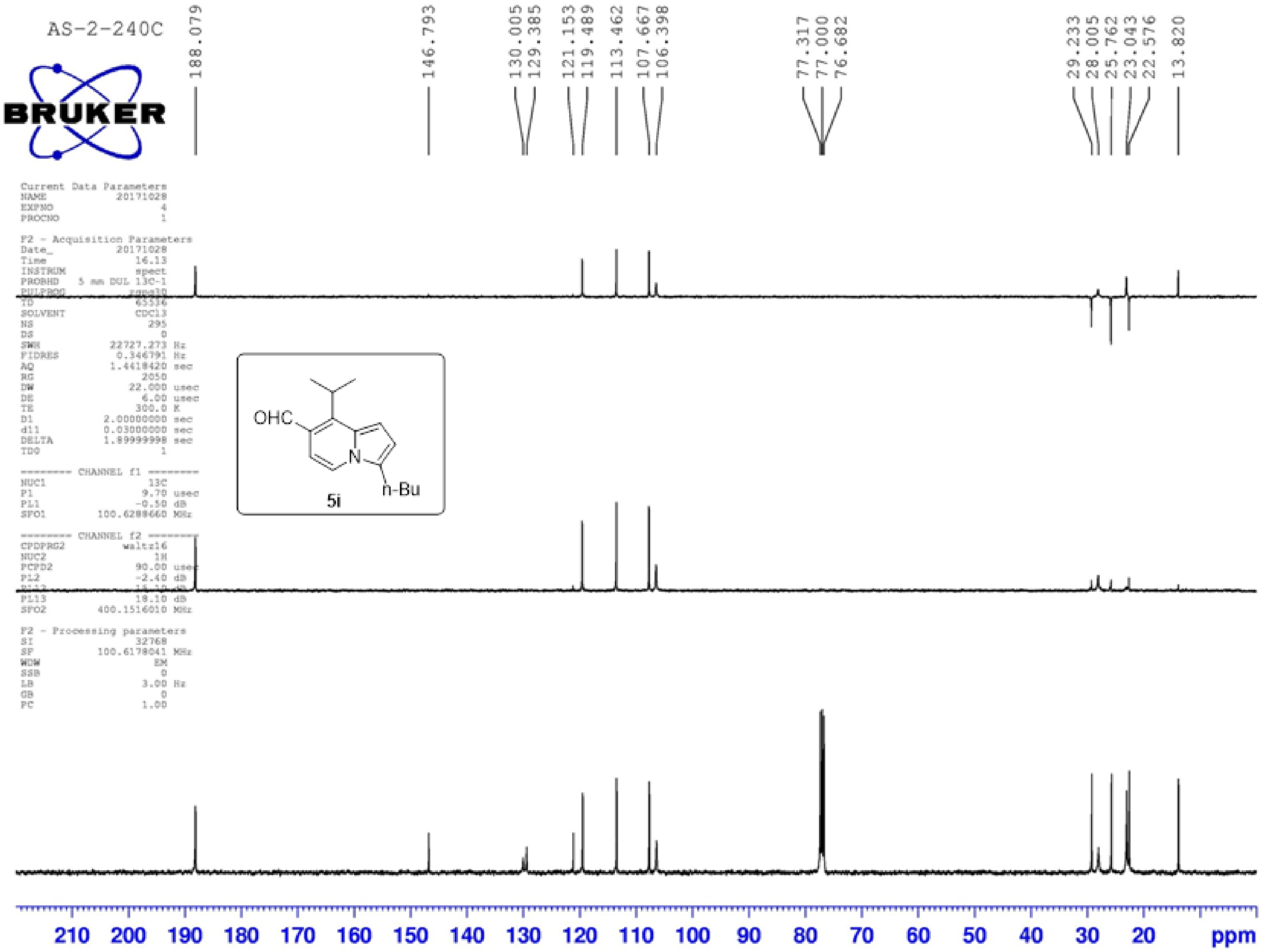
AS-2-240C
BRUKER
188.079
146.793
130.005
129.385
121.153
119.489
113.462
107.667
106.398
77.317
77.000
76.682
29.233
28.005
25.762
23.043
22.576
13.820
Current Data Parameters
NAME 20171028
EXPNO 4
PROCNO 1
F2 - Acquisition Parameters
Date_ 20171028
Time 16.13
INSTRUM spect
PROBHD 5 mm DUL 13C-1
TD 65536
SOLVENT CDCl3
NS 295
DS 0
SWH 22727.273 Hz
FIDRES 0.346791 Hz
AQ 1.4418420 sec
RG 2050
DW 22.000 usec
DE 6.00 usec
TE 300.0 K
D1 2.00000000 sec
d11 0.03000000 sec
DELTA 1.89999998 sec
TD0 1
======== CHANNEL f1 ========
NUC1 13C
P1 9.70 usec
PL1 -0.50 dB
SFO1 100.6288460 MHz
======== CHANNEL f2 ========
CPDPRG2 waltz16
NUC2 1H
PCPD2 90.00 usec
PL2 -2.40 dB
PL13 18.10 dB
SFO2 400.1516010 MHz
F2 - Processing parameters
SI 32768
SF 100.6178041 MHz
WDW EM
SSB 0
LB 3.00 Hz
GB 0
PC 1.00
OHC
N
n-Bu
5i
210 200 190 180 170 160 150 140 130 120 110 100 90 80 70 60 50 40 30 20 ppm

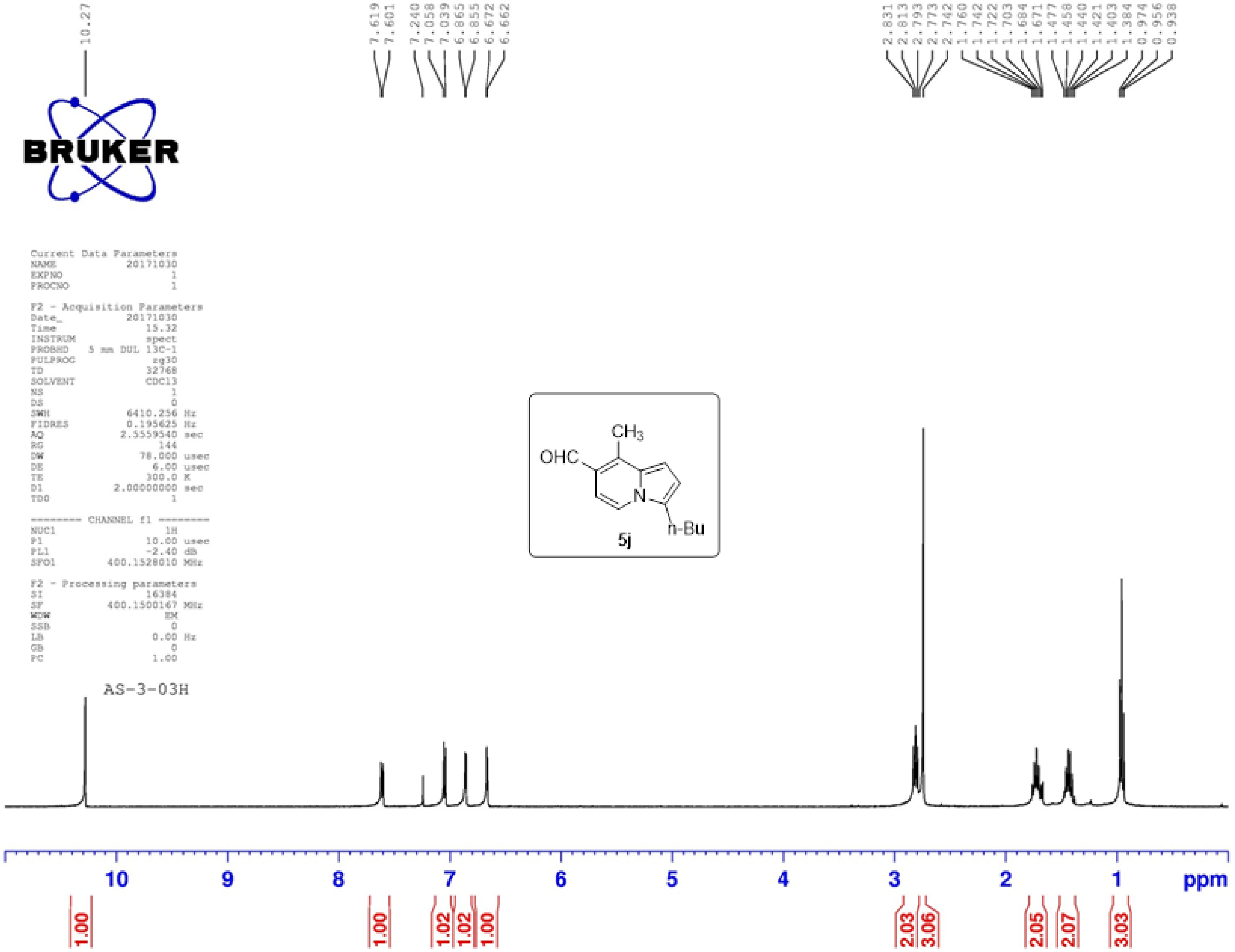
10.27
7.619
7.601
7.240
7.058
7.039
6.865
6.855
6.672
6.662
2.831
2.813
2.793
2.773
2.742
1.760
1.742
1.722
1.703
1.684
1.671
1.477
1.458
1.440
1.421
1.403
1.384
0.974
0.956
0.938
BRUKER
Current Data Parameters
NAME 20171030
EXPNO 1
PROCNO 1
F2 - Acquisition Parameters
Date_ 20171030
Time 15.32
INSTRUM spect
PROBHD 5 mm DUL 13C-1
PULPROG zg30
TD 32768
SOLVENT CDCl3
NS 1
DS 0
SWH 6410.256 Hz
FIDRES 0.195625 Hz
AQ 2.5559540 sec
RG 144
DW 78.000 usec
DE 6.00 usec
TE 300.0 K
D1 2.00000000 sec
TD0 1
======== CHANNEL f1 ========
NUC1 1H
P1 10.00 usec
PL1 -2.40 dB
SFO1 400.1528010 MHz
F2 - Processing parameters
SI 16384
SF 400.1500167 MHz
WDW EM
SSB 0
LB 0.00 Hz
GB 0
PC 1.00
AS-3-03H
OHC
CH3
N
n-Bu
5j
10
9
8
7
6
5
4
3
2
1
ppm
1.00
1.00
1.02
1.02
1.00
2.03
3.06
2.05
2.07
3.03

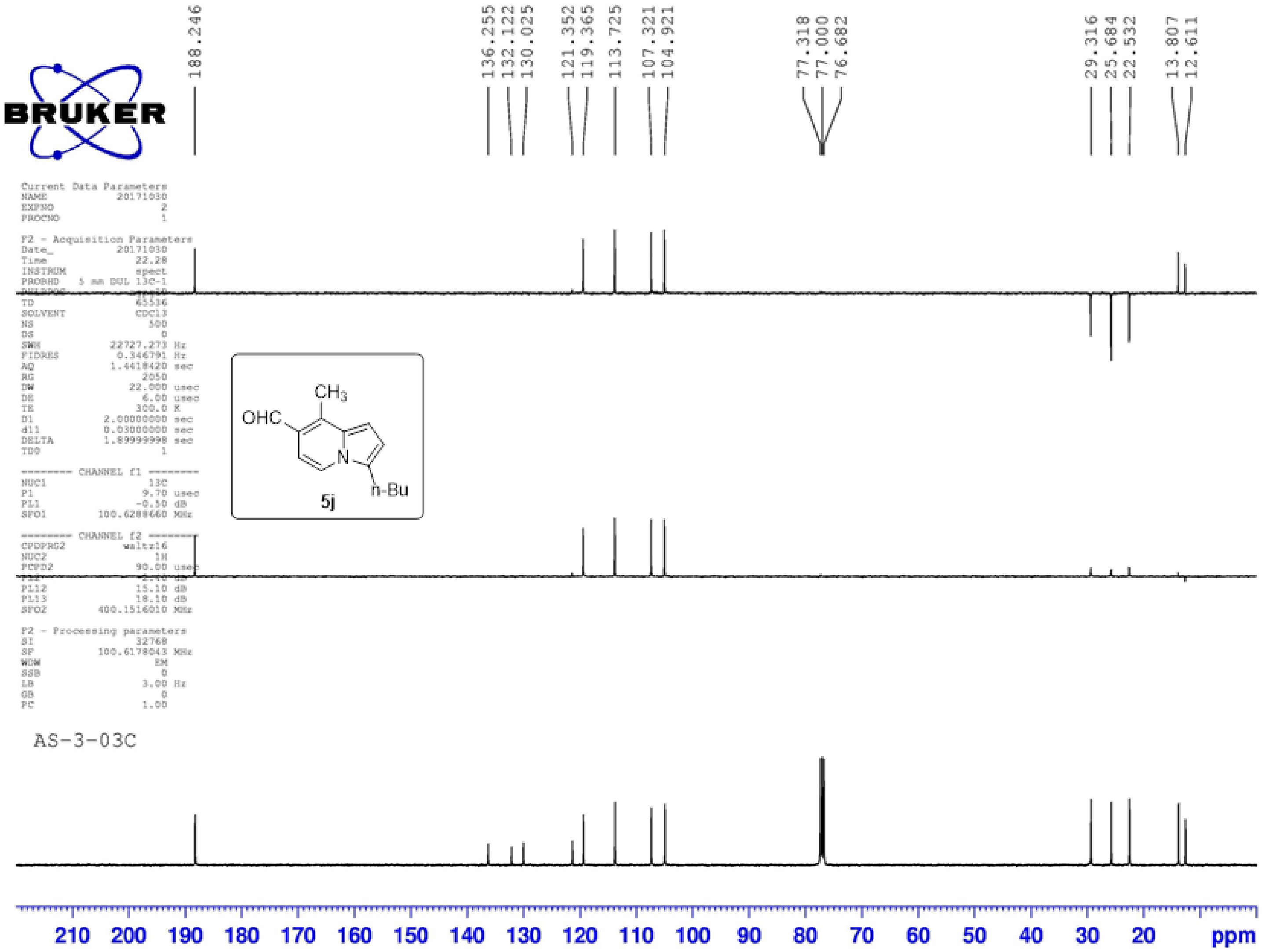
188.246
136.255
132.122
130.025
121.352
119.365
113.725
107.321
104.921
77.318
77.000
76.682
29.316
25.684
22.532
13.807
12.611
BRUKER
Current Data Parameters
NAME 20171030
EXPNO 2
PROCNO 1
F2 - Acquisition Parameters
Date_ 20171030
Time 22.28
INSTRUM spect
PROBHD 5 mm DUL 13C-1
TD 65536
SOLVENT CDCl3
NS 500
DS 0
SWH 22727.273 Hz
FIDRES 0.346791 Hz
AQ 1.4418420 sec
RG 2050
DW 22.000 usec
DE 6.00 usec
TE 300.0 K
D1 2.00000000 sec
d11 0.03000000 sec
DELTA 1.89999998 sec
TD0 1
======== CHANNEL f1 ========
NUC1 13C
P1 9.70 usec
PL1 -0.50 dB
SFO1 100.6288660 MHz
======== CHANNEL f2 ========
CPDPRG2 waltz16
NUC2 1H
PCPD2 90.00 usec
PL12 15.10 dB
PL13 18.10 dB
SFO2 400.1516010 MHz
F2 - Processing parameters
SI 32768
SF 100.6178043 MHz
WDW EM
SSB 0
LB 3.00 Hz
GB 0
PC 1.00
OHC
CH3
N
n-Bu
5j
AS-3-03C
210 200 190 180 170 160 150 140 130 120 110 100 90 80 70 60 50 40 30 20 ppm

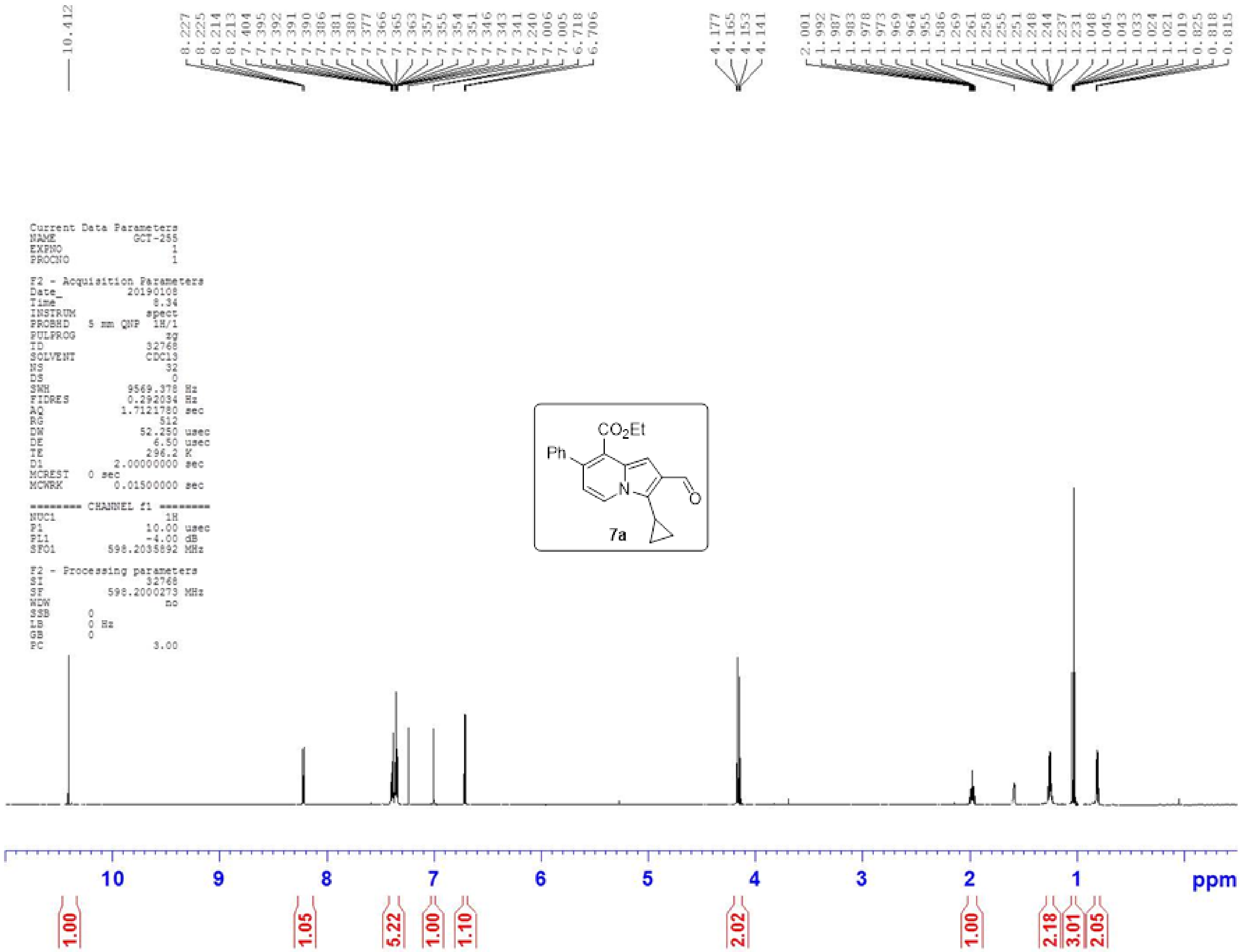
10.412
8.227 8.225 8.214 8.213 7.404 7.395 7.392 7.391 7.390 7.386 7.381 7.380 7.377 7.366 7.365 7.363 7.357 7.355 7.354 7.351 7.346 7.343 7.341 7.240 7.006 7.005 6.718 6.706
4.177 4.165 4.153 4.141
2.001 1.992 1.987 1.983 1.978 1.973 1.969 1.964 1.955 1.586 1.269 1.261 1.258 1.255 1.251 1.248 1.244 1.237 1.231 1.048 1.045 1.043 1.033 1.024 1.021 1.019 0.825 0.818 0.815
Current Data Parameters
NAME GCT-255
EXPNO 1
PROCNO 1
F2 - Acquisition Parameters
Date_ 20190108
Time 8.34
INSTRUM spect
PROBHD 5 mm QNP 1H/1
PULPROG zg
TD 32768
SOLVENT CDCl3
NS 32
DS 0
SWH 9569.378 Hz
FIDRES 0.292034 Hz
AQ 1.7121780 sec
RG 512
DW 52.250 usec
DE 6.50 usec
TE 296.2 K
D1 2.00000000 sec
MCREST 0 sec
MCWRK 0.01500000 sec
======== CHANNEL f1 ========
NUC1 1H
P1 10.00 usec
PL1 -4.00 dB
SFO1 598.2035892 MHz
F2 - Processing parameters
SI 32768
SF 598.2000273 MHz
WDW no
SSB 0
LB 0 Hz
GB 0
PC 3.00
CO2Et
Ph
N
O
7a
10 9 8 7 6 5 4 3 2 1 ppm
1.00 1.05 5.22 1.00 1.10 2.02 1.00 2.18 3.01 2.05

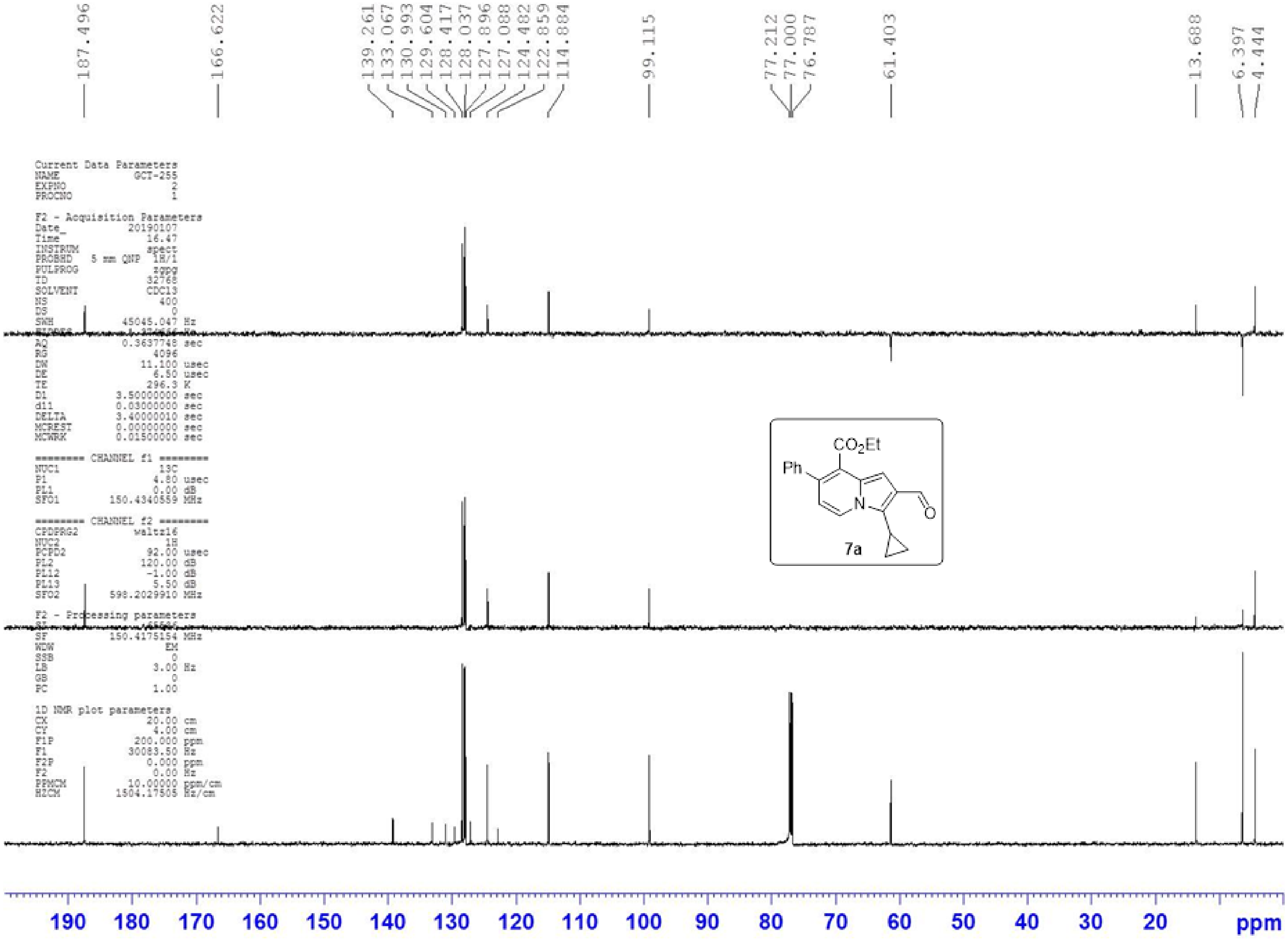

187.496
166.622
139.261
133.067
130.993
129.604
128.417
128.037
127.896
127.088
124.482
122.859
114.884
99.115
77.212
77.000
76.787
61.403
13.688
6.397
4.444
Current Data Parameters
NAME GCT-255
EXPNO 2
PROCNO 1
F2 - Acquisition Parameters
Date_ 20190107
Time 16.47
INSTRUM spect
PROBHD 5 mm QNP 1H/1
PULPROG zgpg
TD 32768
SOLVENT CDCl3
NS 400
DS 0
SWH 45045.047 Hz
AQ 0.3637748 sec
RG 4096
DW 11.100 usec
DE 6.50 usec
TE 296.3 K
D1 3.50000000 sec
d11 0.03000000 sec
DELTA 3.40000010 sec
MCREST 0.00000000 sec
MCWRK 0.01500000 sec
======== CHANNEL f1 ========
NUC1 13C
P1 4.80 usec
PL1 0.00 dB
SFO1 150.4340559 MHz
======== CHANNEL f2 ========
CPDPRG2 waltz16
NUC2 1H
PCPD2 92.00 usec
PL2 120.00 dB
PL12 -1.00 dB
PL13 5.50 dB
SFO2 598.2029910 MHz
F2 - Processing parameters
SF 150.4175154 MHz
WDW EM
SSB 0
LB 3.00 Hz
GB 0
PC 1.00
1D NMR plot parameters
CX 20.00 cm
CY 4.00 cm
F1P 200.000 ppm
F1 30083.50 Hz
F2P 0.000 ppm
F2 0.00 Hz
PPMCM 10.00000 ppm/cm
HZCM 1504.17505 Hz/cm
CO2Et
Ph
N
O
7a
190 180 170 160 150 140 130 120 110 100 90 80 70 60 50 40 30 20 ppm

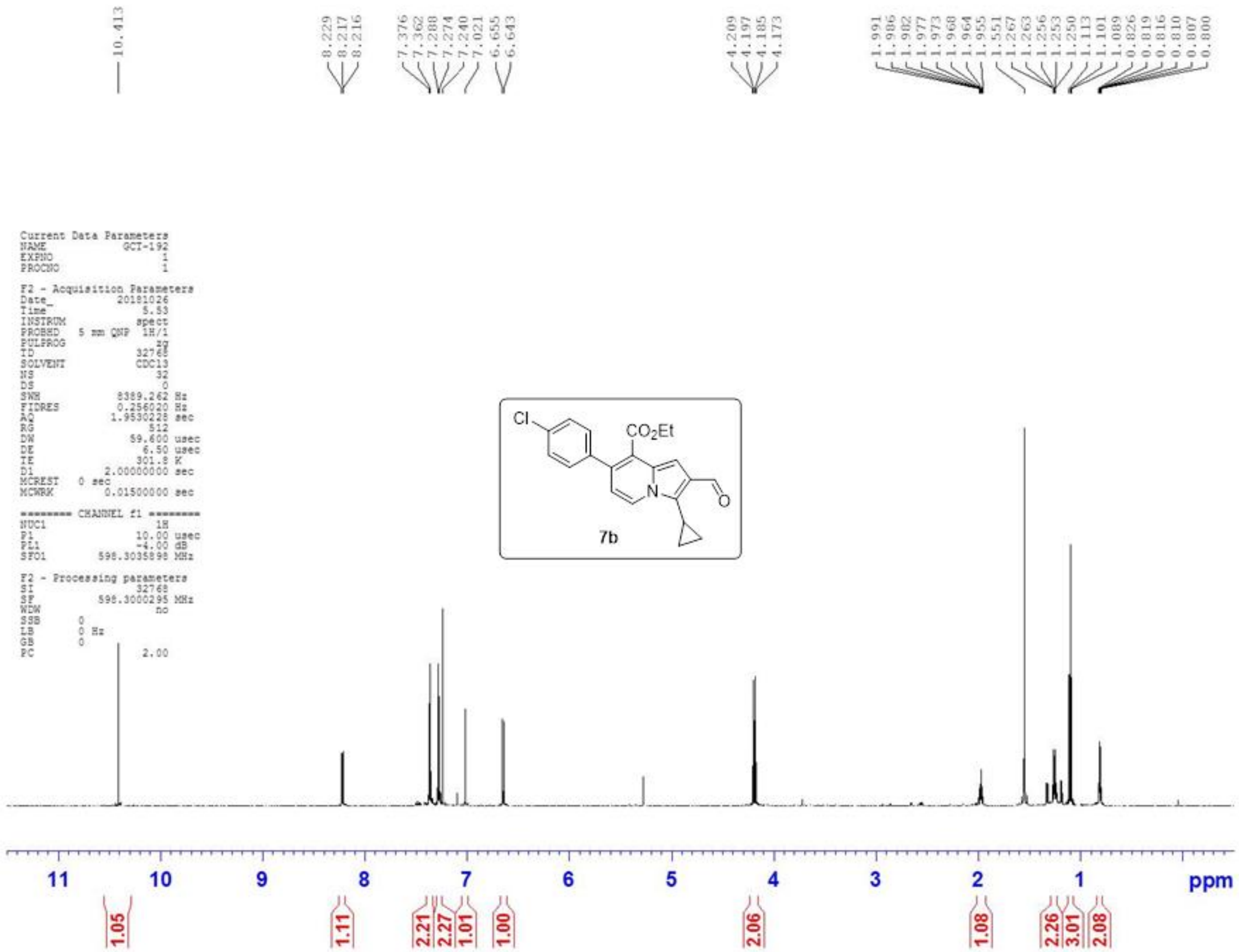
10.413
8.229
8.217
8.216
7.376
7.362
7.288
7.274
7.240
7.021
6.655
6.643
4.209
4.197
4.185
4.173
1.991
1.986
1.982
1.977
1.973
1.968
1.964
1.955
1.551
1.267
1.263
1.256
1.253
1.250
1.113
1.101
1.089
0.826
0.819
0.816
0.810
0.807
0.800
Current Data Parameters
NAME GCT-192
EXPNO 1
PROCNO 1
F2 - Acquisition Parameters
Date_ 20181026
Time 5.53
INSTRUM spect
PROBHD 5 mm QNP 1H/1
PULPROG zg
TD 32768
SOLVENT CDCl3
NS 32
DS 0
SWH 8389.262 Hz
FIDRES 0.256020 Hz
AQ 1.9530228 sec
RG 512
DW 59.600 usec
DE 6.50 usec
TE 301.8 K
D1 2.00000000 sec
MCREST 0 sec
MCWRK 0.01500000 sec
======== CHANNEL f1 ========
NUC1 1H
P1 10.00 usec
PL1 -4.00 dB
SFO1 598.3035898 MHz
F2 - Processing parameters
SI 32768
SF 598.3000295 MHz
WDW no
SSB 0
LB 0 Hz
GB 0
PC 2.00
Cl
CO2Et
N
O
7b
11
10
9
8
7
6
5
4
3
2
1
ppm
1.05
1.11
2.21
2.27
1.01
1.00
2.06
1.08
2.26
3.01
2.08

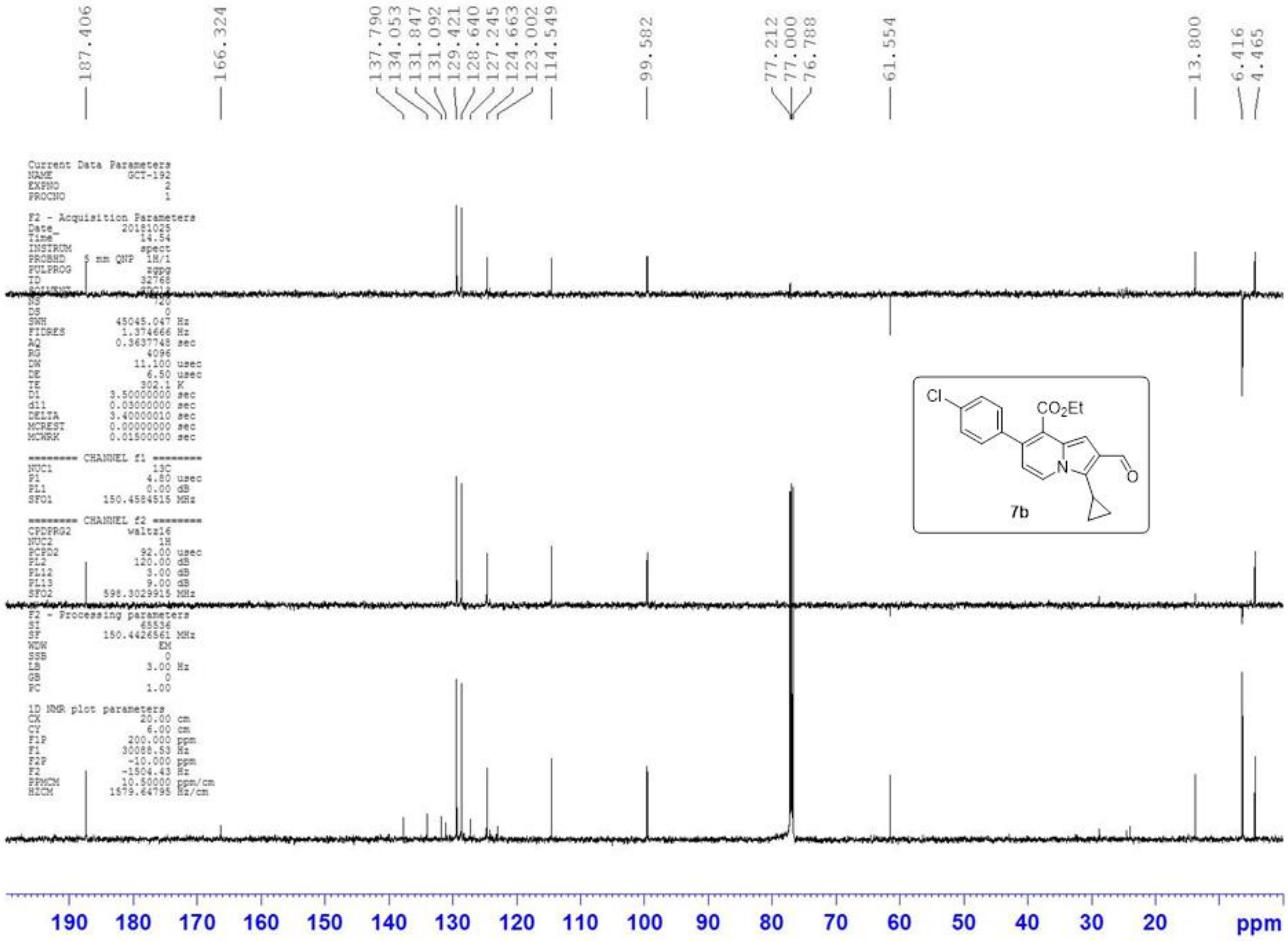
187.406
166.324
137.790
134.053
131.847
131.092
129.421
128.640
127.245
124.663
123.002
114.549
99.582
77.212
77.000
76.788
61.554
13.800
6.416
4.465
Current Data Parameters
NAME GCT-192
EXPNO 2
PROCNO 1
F2 - Acquisition Parameters
Date_ 20181025
Time 14.54
INSTRUM spect
PROBHD 5 mm QNP 1H/1
PULPROG zgpg
TD 32768
NS 120
DS 0
SWH 45045.047 Hz
FIDRES 1.374666 Hz
AQ 0.3637748 sec
RG 4096
DW 11.100 usec
DE 6.50 usec
TE 302.1 K
D1 3.50000000 sec
d11 0.03000000 sec
DELTA 3.40000010 sec
MCREST 0.00000000 sec
MCWRK 0.01500000 sec
======== CHANNEL f1 ========
NUC1 13C
P1 4.80 usec
PL1 0.00 dB
SFO1 150.4584515 MHz
======== CHANNEL f2 ========
CPDPRG2 waltz16
NUC2 1H
PCPD2 92.00 usec
PL2 120.00 dB
PL12 3.00 dB
PL13 9.00 dB
SFO2 598.3029915 MHz
F2 - Processing parameters
SI 65536
SF 150.4426561 MHz
WDW EM
SSB 0
LB 3.00 Hz
GB 0
PC 1.00
1D NMR plot parameters
CX 20.00 cm
CY 6.00 cm
F1P 200.000 ppm
F1 30088.53 Hz
F2P -10.000 ppm
F2 -1504.43 Hz
PPMCM 10.50000 ppm/cm
HZCM 1579.64795 Hz/cm
Cl
CO2Et
N
O
7b
190
180
170
160
150
140
130
120
110
100
90
80
70
60
50
40
30
20
ppm

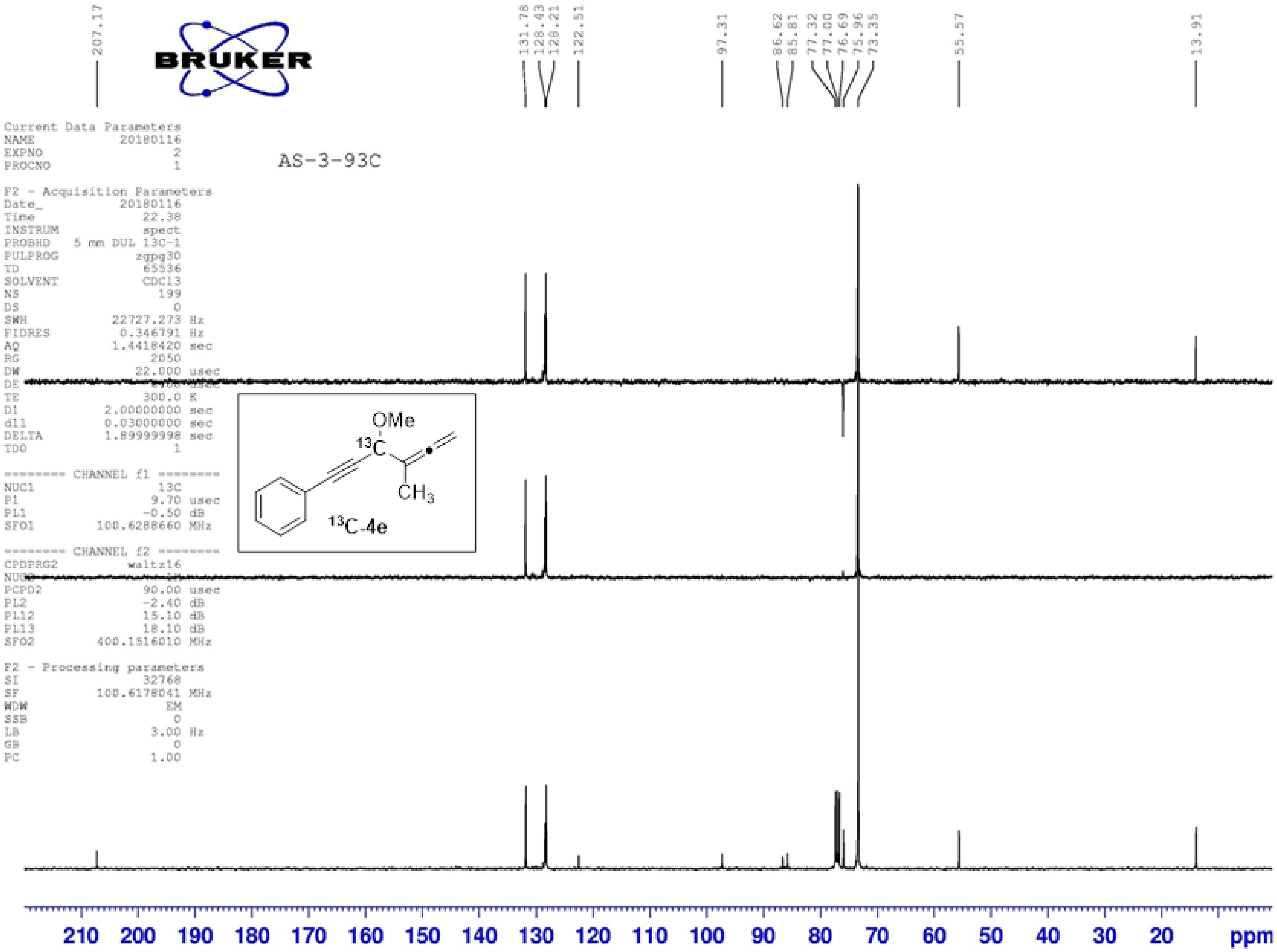
207.17
131.78
128.43
128.21
122.51
97.31
86.62
85.81
77.32
77.00
76.69
75.96
73.35
55.57
13.91
BRUKER
AS-3-93C
Current Data Parameters
NAME 20180116
EXPNO 2
PROCNO 1
F2 - Acquisition Parameters
Date_ 20180116
Time 22.38
INSTRUM spect
PROBHD 5 mm DUL 13C-1
PULPROG zgpg30
TD 65536
SOLVENT CDCl3
NS 199
DS 0
SWH 22727.273 Hz
FIDRES 0.346791 Hz
AQ 1.4418420 sec
RG 2050
DW 22.000 usec
TE 300.0 K
D1 2.00000000 sec
d11 0.03000000 sec
DELTA 1.89999998 sec
TD0 1
======== CHANNEL f1 ========
NUC1 13C
P1 9.70 usec
PL1 -0.50 dB
SFO1 100.6288660 MHz
======== CHANNEL f2 ========
CPDPRG2 waltz16
PCPD2 90.00 usec
PL2 -2.40 dB
PL12 15.10 dB
PL13 18.10 dB
SFO2 400.1516010 MHz
F2 - Processing parameters
SI 32768
SF 100.6178041 MHz
WDW EM
SSB 0
LB 3.00 Hz
GB 0
PC 1.00
OMe
13C
CH3
13C-4e
210 200 190 180 170 160 150 140 130 120 110 100 90 80 70 60 50 40 30 20 ppm

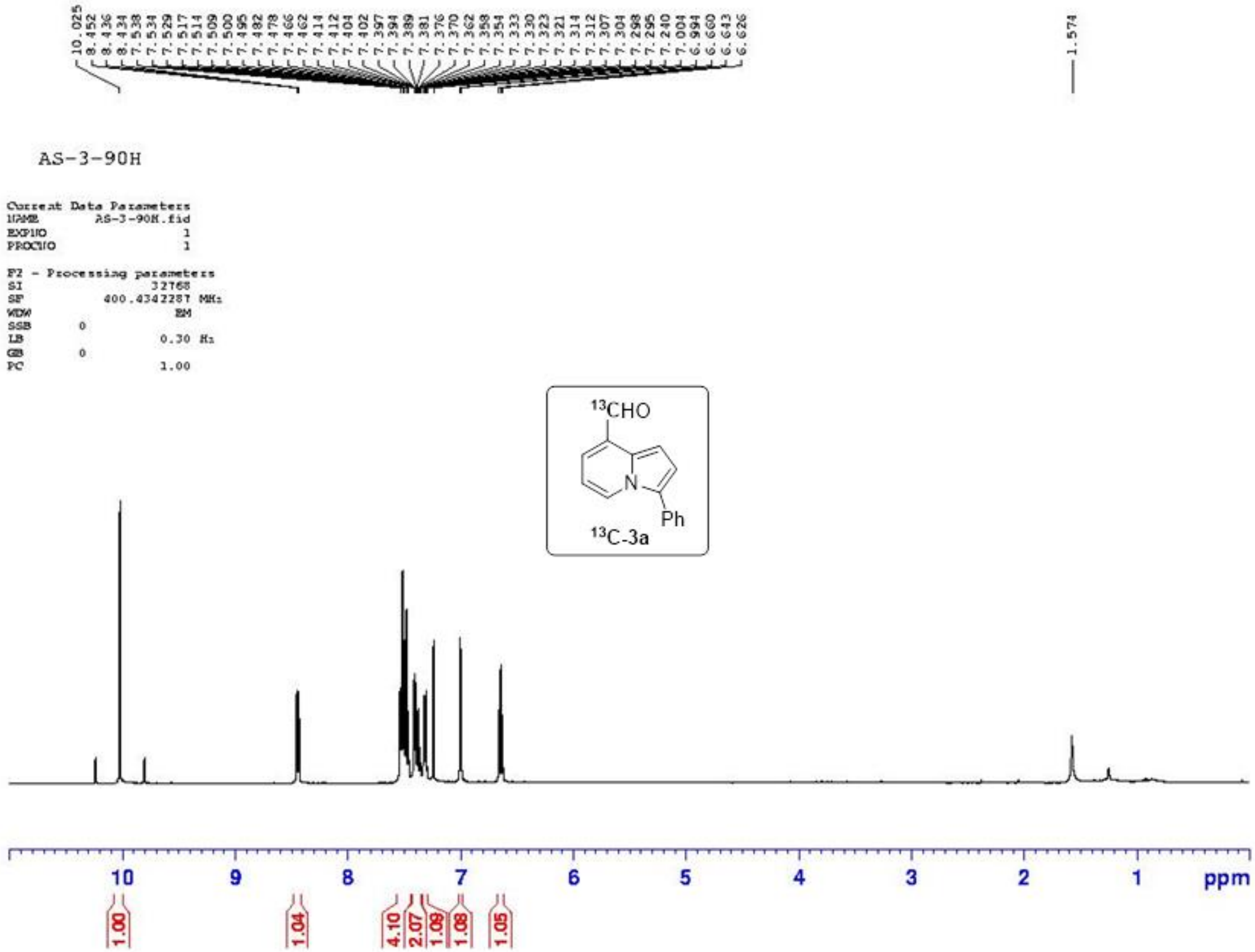
10.025
8.452
8.436
8.434
7.538
7.534
7.529
7.517
7.514
7.509
7.500
7.495
7.482
7.478
7.466
7.462
7.414
7.412
7.404
7.402
7.397
7.394
7.389
7.381
7.376
7.370
7.362
7.358
7.354
7.333
7.330
7.323
7.321
7.314
7.312
7.307
7.304
7.298
7.295
7.240
7.004
6.994
6.660
6.643
6.626
1.574
AS-3-90H
Current Data Parameters
NAME AS-3-90H.fid
EXPNO 1
PROCNO 1
F2 - Processing parameters
SI 32768
SF 400.4342287 MHz
WDW EM
SSB 0
LB 0.30 Hz
GB 0
PC 1.00
13CHO
N
Ph
13C-3a
10
9
8
7
6
5
4
3
2
1
ppm
1.00
1.04
4.10
2.07
1.09
1.08
1.05

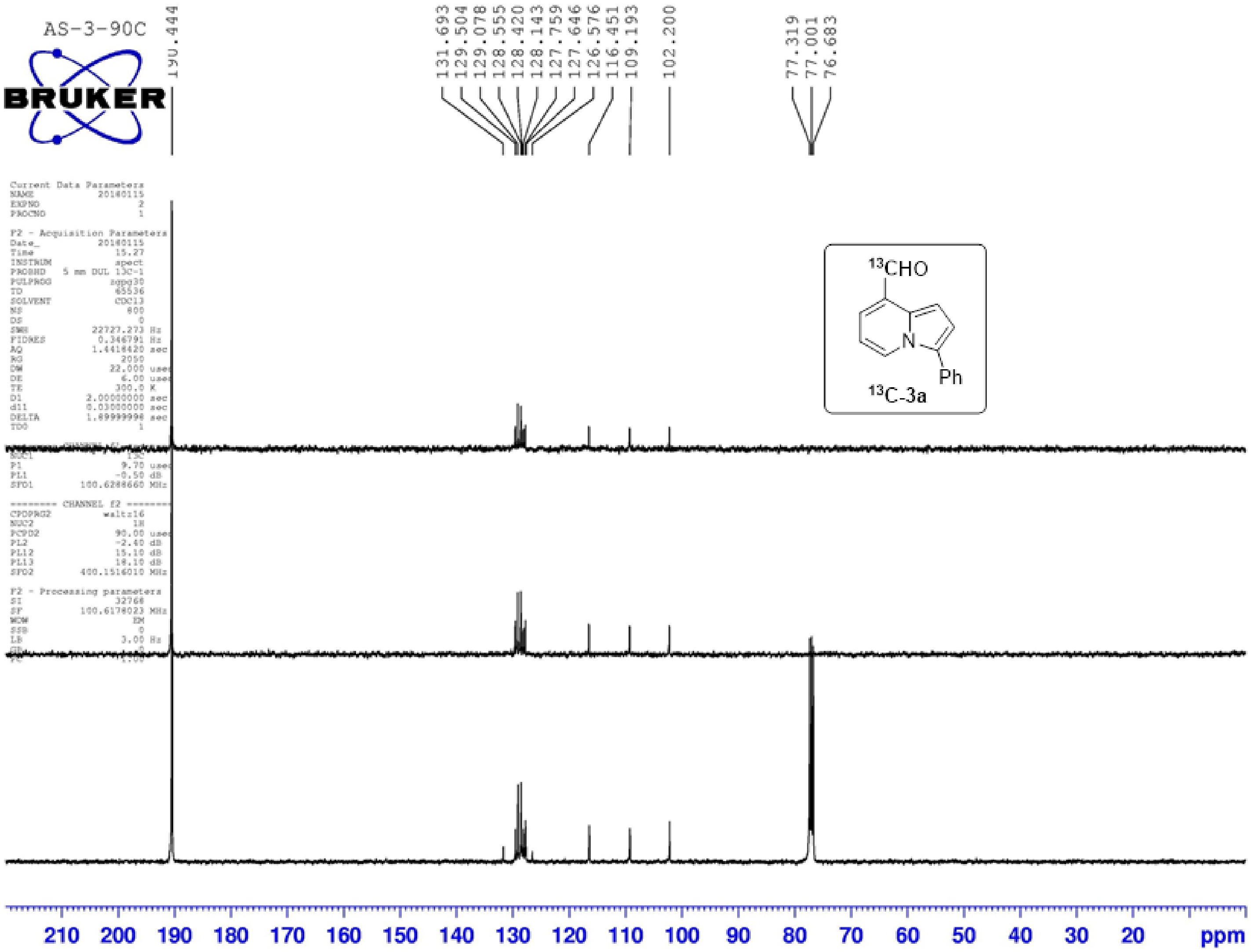
AS-3-90C
190.444
131.693
129.504
129.078
128.555
128.420
128.143
127.759
127.646
126.576
116.451
109.193
102.200
77.319
77.001
76.683
13CHO
N
Ph
13C-3a
210 200 190 180 170 160 150 140 130 120 110 100 90 80 70 60 50 40 30 20 ppm

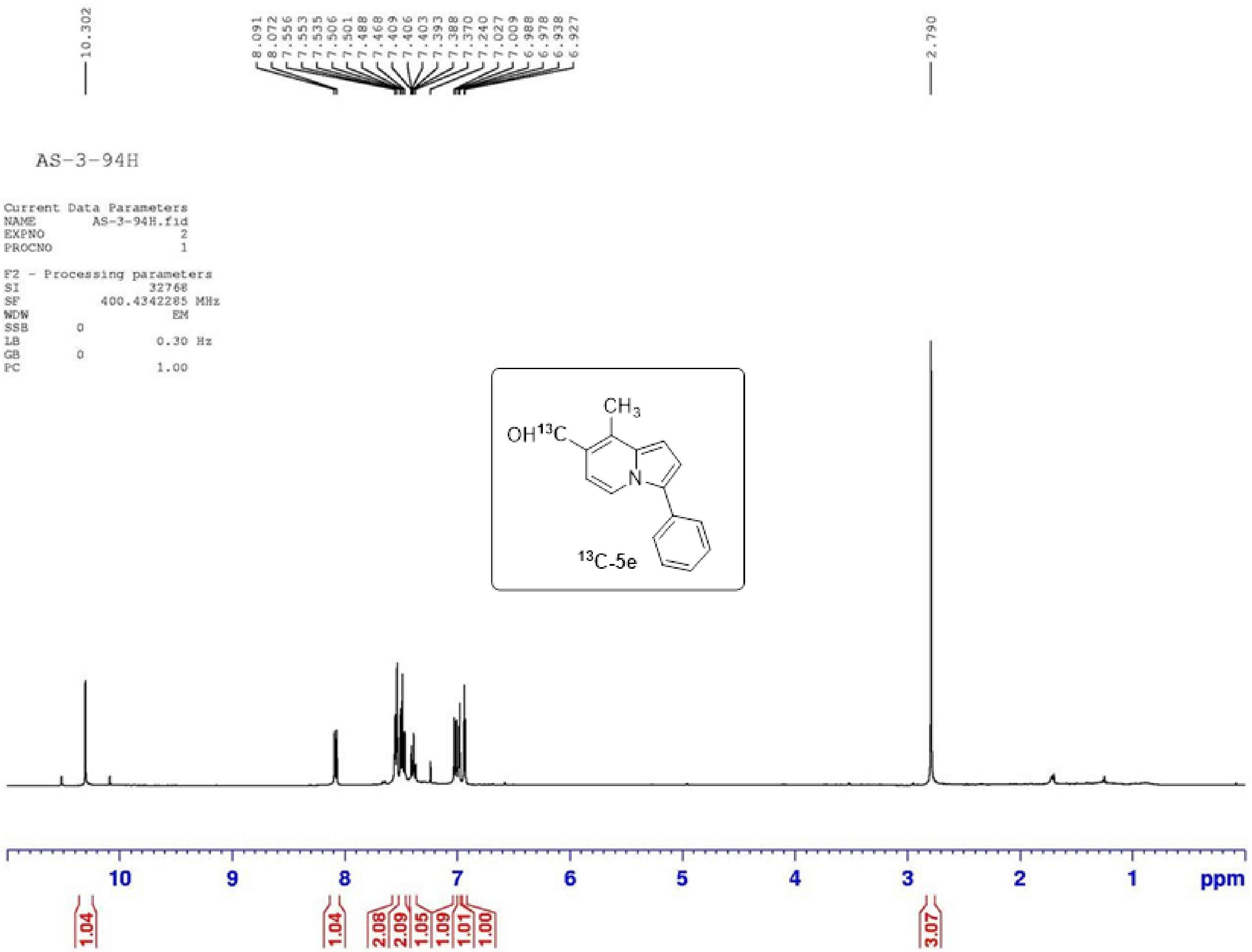
10.302
8.091
8.072
7.556
7.553
7.535
7.506
7.501
7.488
7.468
7.409
7.406
7.403
7.393
7.388
7.370
7.240
7.027
7.009
6.988
6.978
6.938
6.927
2.790
AS-3-94H
Current Data Parameters
NAME AS-3-94H.fid
EXPNO 2
PROCNO 1
F2 - Processing parameters
SI 32768
SF 400.4342285 MHz
WDW EM
SSB 0
LB 0.30 Hz
GB 0
PC 1.00
CH3
OH13C
N
13C-5e
10
9
8
7
6
5
4
3
2
1
ppm
1.04
1.04
2.08
2.09
1.05
1.09
1.01
1.00
3.07

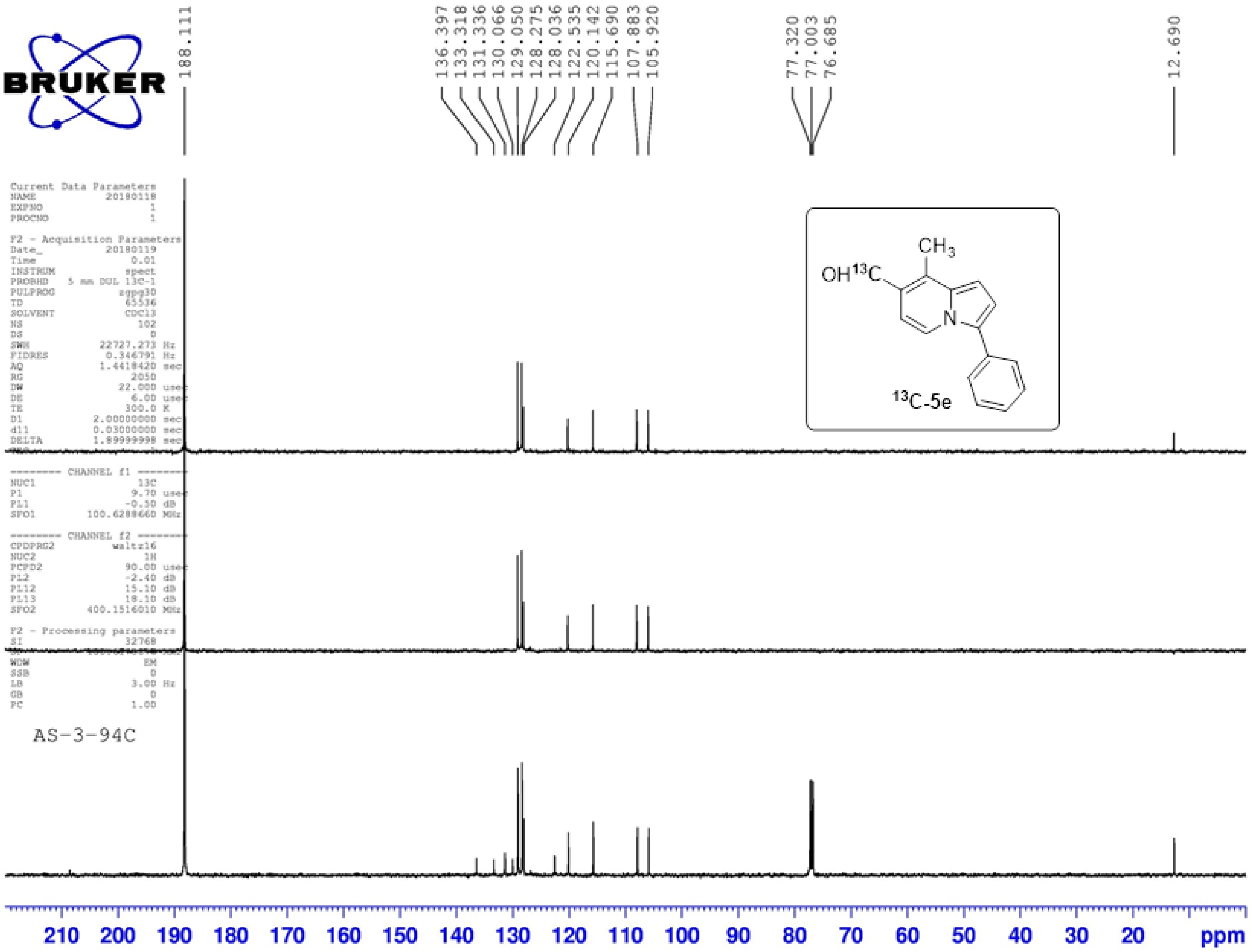
188.111
136.397
133.318
131.336
130.066
129.050
128.275
128.036
122.535
120.142
115.690
107.883
105.920
77.320
77.003
76.685
12.690
AS-3-94C
$^{13}$C-5e
ppm

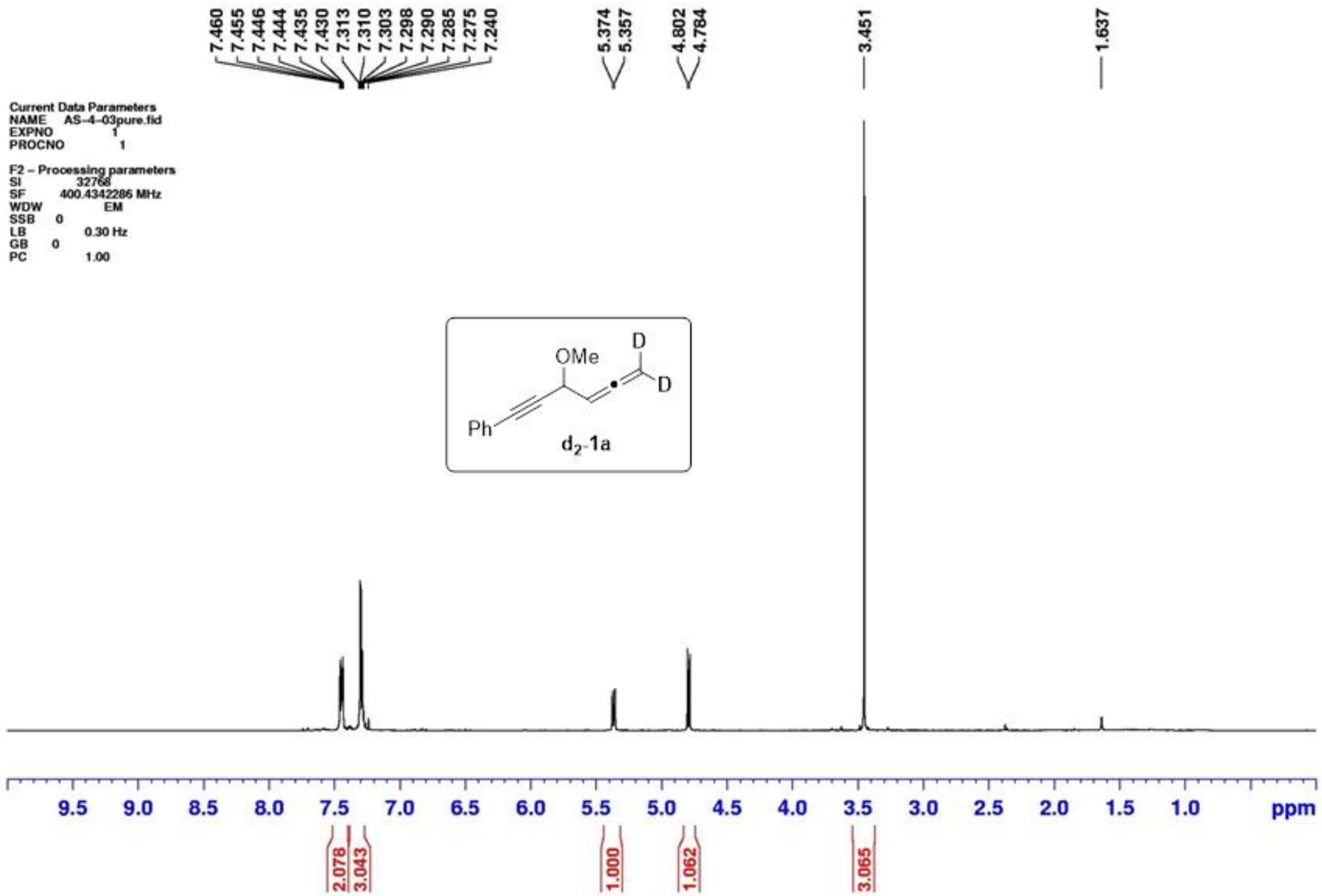

7.460
7.455
7.446
7.444
7.435
7.430
7.313
7.310
7.303
7.298
7.290
7.285
7.275
7.240
5.374
5.357
4.802
4.784
3.451
1.637
Current Data Parameters
NAME AS-4-03pure.fid
EXPNO 1
PROCNO 1
F2 - Processing parameters
SI 32768
SF 400.4342286 MHz
WDW EM
SSB 0
LB 0.30 Hz
GB 0
PC 1.00
OMe
D
D
Ph
d2-1a
9.5
9.0
8.5
8.0
7.5
7.0
6.5
6.0
5.5
5.0
4.5
4.0
3.5
3.0
2.5
2.0
1.5
1.0
ppm
2.078
3.043
1.000
1.062
3.065

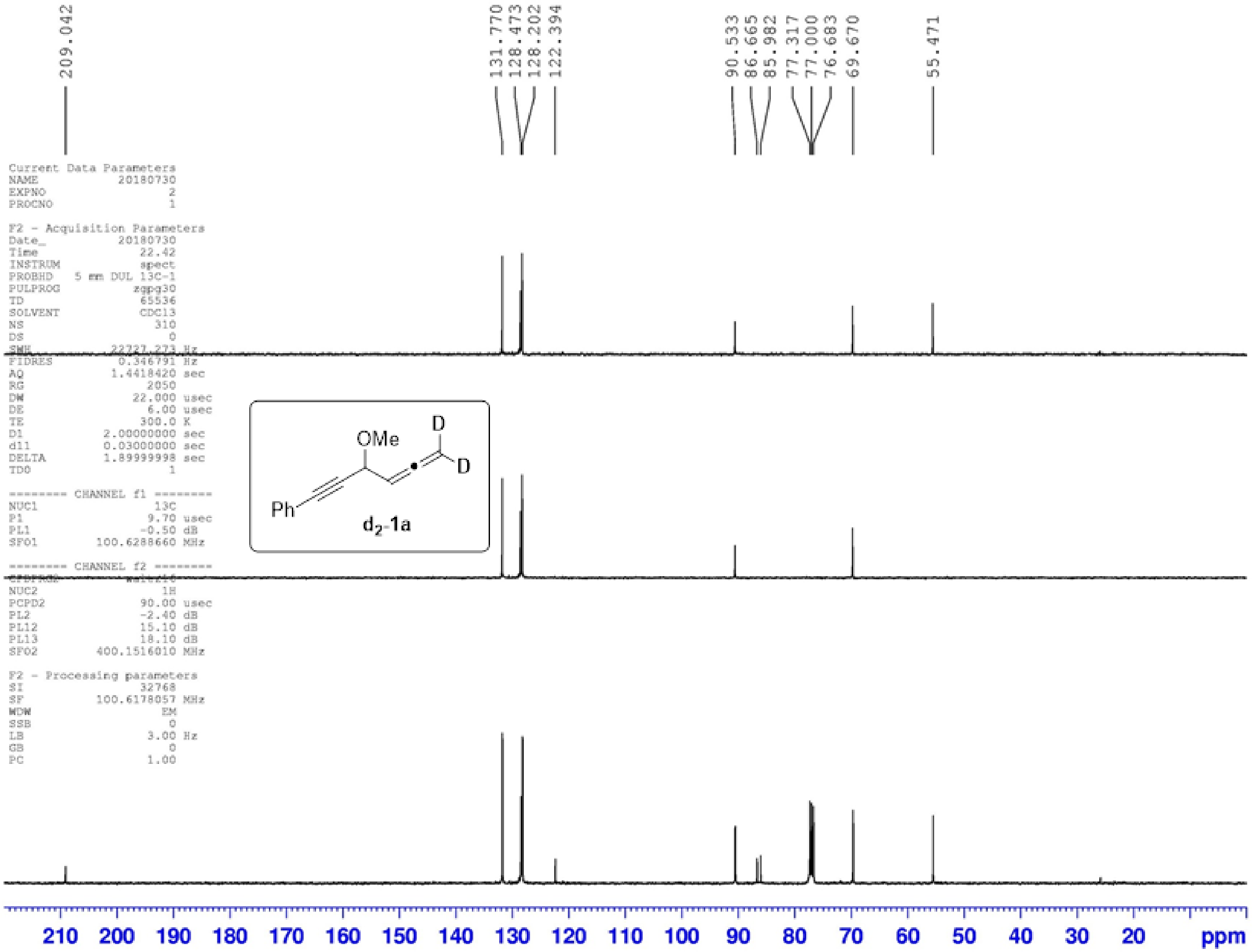
209.042
131.770
128.473
128.202
122.394
90.533
86.665
85.982
77.317
77.000
76.683
69.670
55.471
Current Data Parameters
NAME 20180730
EXPNO 2
PROCNO 1
F2 - Acquisition Parameters
Date_ 20180730
Time 22.42
INSTRUM spect
PROBHD 5 mm DUL 13C-1
PULPROG zgpg30
TD 65536
SOLVENT CDCl3
NS 310
DS 0
SWH 22727.273 Hz
FIDRES 0.346791 Hz
AQ 1.4418420 sec
RG 2050
DW 22.000 usec
DE 6.00 usec
TE 300.0 K
D1 2.00000000 sec
d11 0.03000000 sec
DELTA 1.89999998 sec
TD0 1
======== CHANNEL f1 ========
NUC1 13C
P1 9.70 usec
PL1 -0.50 dB
SFO1 100.6288660 MHz
======== CHANNEL f2 ========
NUC2 1H
PCPD2 90.00 usec
PL2 -2.40 dB
PL12 15.10 dB
PL13 18.10 dB
SFO2 400.1516010 MHz
F2 - Processing parameters
SI 32768
SF 100.6178057 MHz
WDW EM
SSB 0
LB 3.00 Hz
GB 0
PC 1.00
OMe
D
D
Ph
d2-1a
210 200 190 180 170 160 150 140 130 120 110 100 90 80 70 60 50 40 30 20 ppm

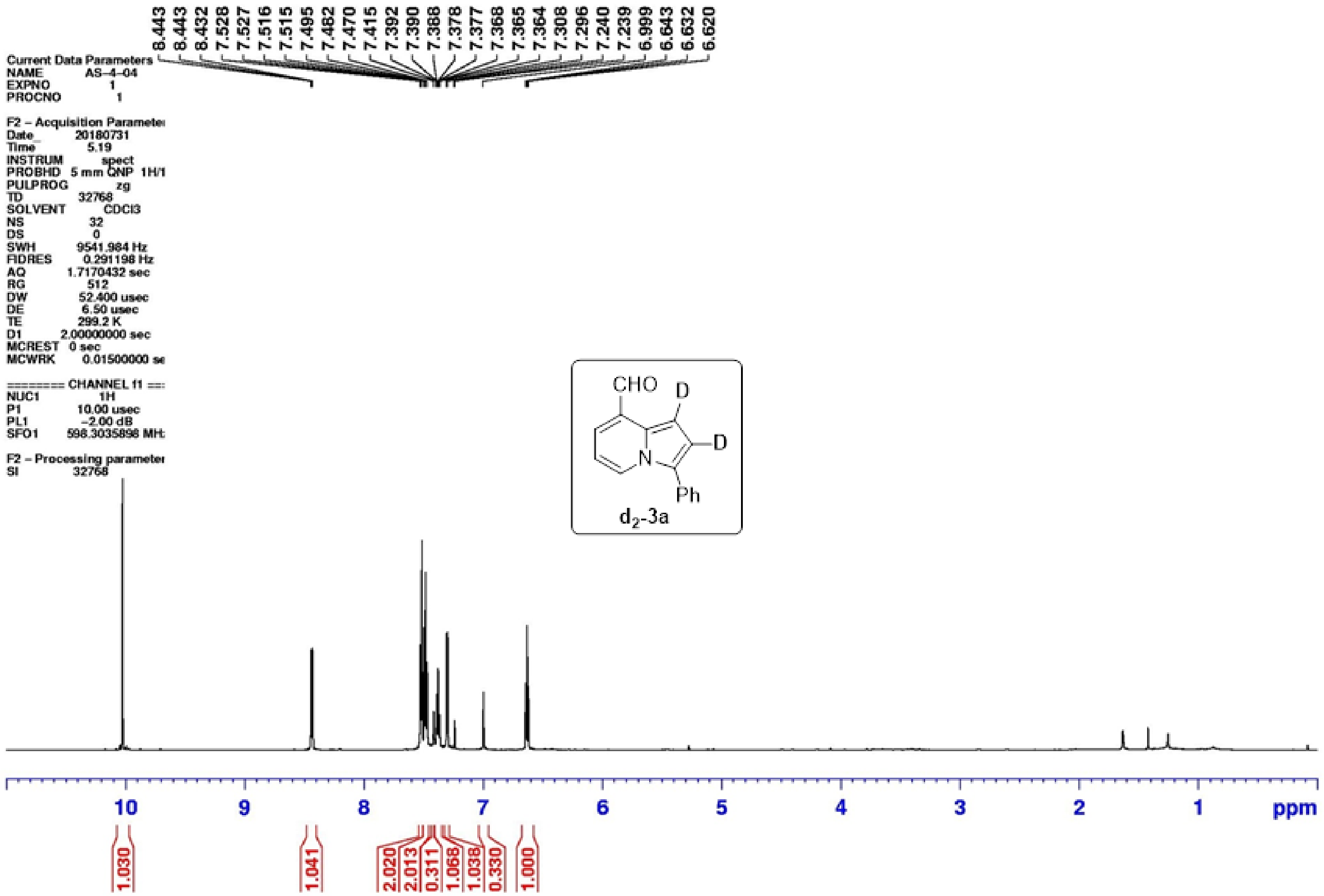
8.443
8.443
8.432
7.528
7.527
7.516
7.515
7.495
7.482
7.470
7.415
7.392
7.390
7.388
7.378
7.377
7.368
7.365
7.364
7.308
7.296
7.240
7.239
6.999
6.643
6.632
6.620
Current Data Parameters
NAME AS-4-04
EXPNO 1
PROCNO 1
F2 - Acquisition Parameters
Date_ 20180731
Time 5.19
INSTRUM spect
PROBHD 5 mm QNP 1H/1
PULPROG zg
TD 32768
SOLVENT CDCl3
NS 32
DS 0
SWH 9541.984 Hz
FIDRES 0.291198 Hz
AQ 1.7170432 sec
RG 512
DW 52.400 usec
DE 6.50 usec
TE 299.2 K
D1 2.00000000 sec
MCREST 0 sec
MCWRK 0.01500000 se
======== CHANNEL f1 ===
NUC1 1H
P1 10.00 usec
PL1 -2.00 dB
SFO1 598.3035898 MH
F2 - Processing parameters
SI 32768
CHO
D
D
N
Ph
d2-3a
10
9
8
7
6
5
4
3
2
1
ppm
1.030
1.041
2.020
2.013
0.311
1.068
1.038
0.330
1.000

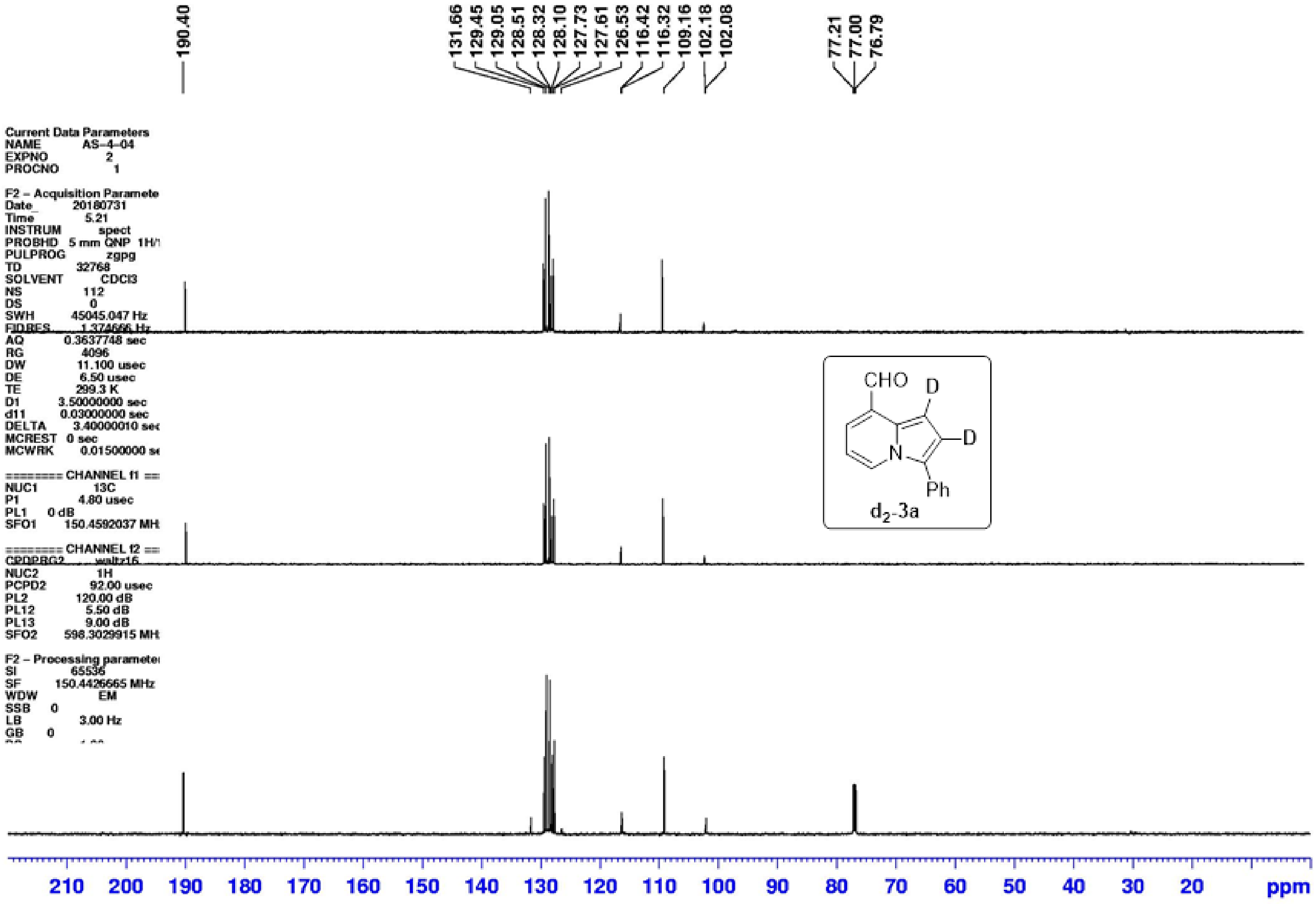
190.40
131.66
129.45
129.05
128.51
128.32
128.10
127.73
127.61
126.53
116.42
116.32
109.16
102.18
102.08
77.21
77.00
76.79
Current Data Parameters
NAME AS-4-04
EXPNO 2
PROCNO 1
F2 - Acquisition Paramete
Date_ 20180731
Time 5.21
INSTRUM spect
PROBHD 5 mm QNP 1H/1
PULPROG zgpg
TD 32768
SOLVENT CDCl3
NS 112
DS 0
SWH 45045.047 Hz
FIDRES 1.374666 Hz
AQ 0.3637748 sec
RG 4096
DW 11.100 usec
DE 6.50 usec
TE 299.3 K
D1 3.50000000 sec
d11 0.03000000 sec
DELTA 3.40000010 sec
MCREST 0 sec
MCWRK 0.01500000 sec
======== CHANNEL f1 ==
NUC1 13C
P1 4.80 usec
PL1 0 dB
SFO1 150.4592037 MH
======== CHANNEL f2 ==
CPDPRG2 waltz16
NUC2 1H
PCPD2 92.00 usec
PL2 120.00 dB
PL12 5.50 dB
PL13 9.00 dB
SFO2 598.3029915 MH
F2 - Processing paramete
SI 65536
SF 150.4426665 MHz
WDW EM
SSB 0
LB 3.00 Hz
GB 0
CHO
D
D
N
Ph
d2-3a
210 200 190 180 170 160 150 140 130 120 110 100 90 80 70 60 50 40 30 20 ppm
